# Deconstructive Functionalization of Unstrained Cycloalkanols via Electrochemically Generated Aromatic Radical Cations

**DOI:** 10.1021/acs.orglett.3c00219

**Published:** 2023-02-27

**Authors:** James Harnedy, Hussain A. Maashi, Albara A. M. A. El Gehani, Matthew Burns, Louis C. Morrill

**Affiliations:** † Cardiff Catalysis Institute, School of Chemistry, Cardiff University, Main Building, Park Place, Cardiff CF10 3AT, U.K.; ‡ Chemical Development, Pharmaceutical Technology & Development, Operations, AstraZeneca, Macclesfield SK10 2NA, U.K.

## Abstract

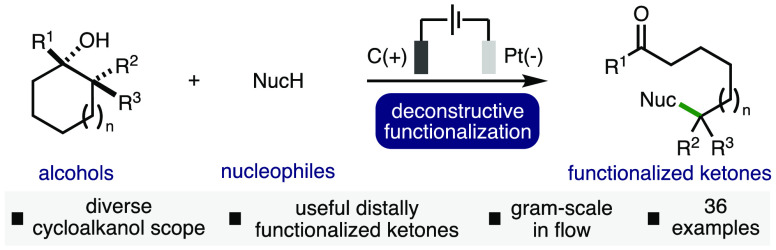

Herein we report an electrochemical approach for the deconstructive functionalization of cycloalkanols, where various alcohols, carboxylic acids, and *N*-heterocycles are employed as nucleophiles. The method has been demonstrated across a broad range of cycloalkanol substrates, including various ring sizes and substituents, to access useful remotely functionalized ketone products (36 examples). The method was demonstrated on a gram scale via single-pass continuous flow, which exhibited increased productivity in relation to the batch process.

In synthetic chemistry, molecular complexity is most commonly established through the sequential construction of new bonds over multiple steps. However, an alternative approach, deconstructive functionalization,^1^ provides a complementary method to rapidly access structurally complex and functional molecular fragments that would often be challenging, or impossible, to access using existing synthetic methods.^2^ As a representative example of this strategy, in 2018, Sarpong and co-workers reported the deconstructive fluorination of readily accessible unstrained cyclic amines, which provided convenient access to valuable remotely fluorinated amides in one step (Scheme 1A).^3^ Within this domain, the deconstructive functionalization of cycloalkanols has received considerable attention due to the prevalence of the alcohol functional group in naturally occurring compounds, pharmaceuticals, agrochemicals, dyes, fragrances, polymers, functional materials, and catalysts, combined with its ease of installation within molecules (Scheme 1B).^4^ Such transformations often proceed via the generation of highly reactive electrophilic alkoxy radical intermediates, which can undergo β-scission of strong β–C–C σ-bonds to form a carbonyl functionality in addition to nucleophilic carbon-centered radicals, which subsequently participate in various bond-forming processes.^5^ Despite providing access to a broad range of useful remotely functionalized carbonyl compounds, many of these approaches require the use of stoichiometric oxidants (e.g., K_2_S_2_O_8_ or hypervalent iodine reagents) and/or precious metal (photo)catalysts.^6^ However, building upon pioneering work by Nikishin and co-workers,^7^ several research groups have reported electrochemical approaches for the deconstructive functionalization of cycloalkanols,^8^ which typically employ acid as the terminal oxidant and generate hydrogen gas as a byproduct. Despite these advances, most existing electrochemical approaches are characterized by limitations relating to the cycloalkanol ring size and/or the types of functionalizations possible, which impacts negatively the breadth of accessible products. For example, our previously developed Mn-catalyzed deconstructive chlorination method was limited to 3- and 4-membered cycloalkanols,^9^ whereas we recently described an alternative approach enabled by proton-coupled electron transfer, which tolerated cycloalkanols of various ring sizes, but was predominately limited to deconstructive bromination.^10^ As part of our ongoing interest in the development of new electrosynthetic methodologies^11^ and with a view to addressing the aforementioned limitations, herein we report an alternative electrochemical method for the deconstructive functionalization of cycloalkanols via the formation of aromatic radical cations and the associated weakening/breaking of β–C-C σ-bonds. This approach tolerates a broad range of ring sizes and employs nucleophiles, including alcohols, carboxylic acids, and *N*-heterocycles, to generate a more diverse array of valuable remotely functionalized ketones (Scheme 1C).

**Scheme 1:**
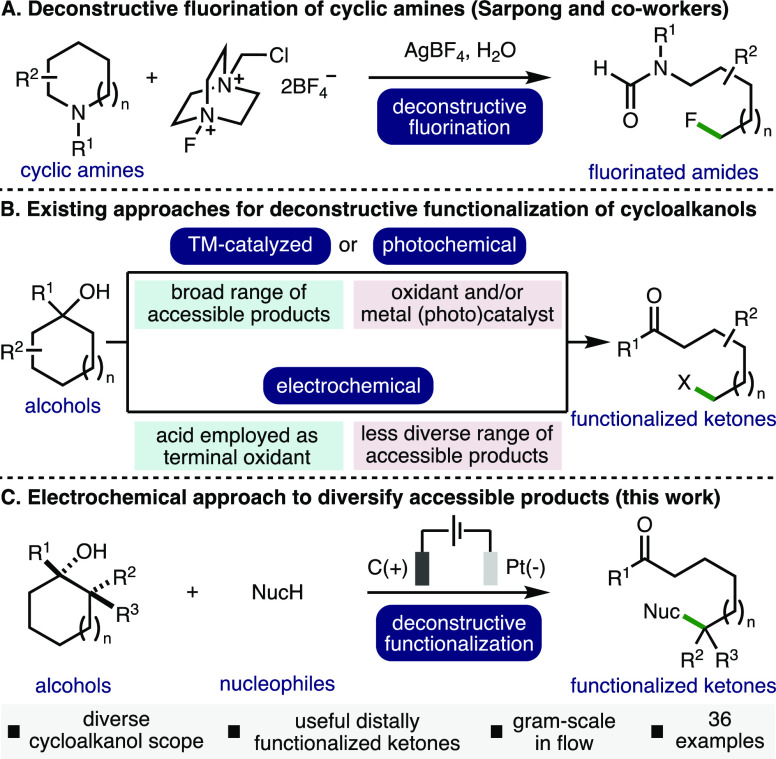
Context

The optimized electrochemical reaction conditions for the deconstructive methoxylation of cyclohexanol **1** (*E*_p/2_ = 1.08 V vs Fc/Fc^+^) employed *n*-Bu_4_NPF_6_ as the supporting electrolyte in CH_2_Cl_2_/MeOH (3:1, [**1**] = 0.05 M), galvanostatic conditions (*i* = 10 mA, *j*_anode_ = 7.8 mA/cm^2^, 3 *F*), and graphite electrodes in an undivided cell at 25 °C under N_2_, which gave ε-methoxy ketone **2** in 67% NMR yield (Table 1, entry 1).^12^ No product formation and quantitative recovery of **1** was observed in the absence of electricity (entry 2). Employing a constant cell potential (*E*_cell_ = 4.5 V) resulted in only 54% conversion to **2** after 3 *F* of charge was passed (entry 3). Alterations to the current applied (*i* = 7.5 or 12.5 mA) lowered the yield of **2** (entry 4), as did variation of electrode materials (entries 5 and 6), electrolyte (entry 7), electrolyte/substrate concentration (entries 8 and 9), solvent mixture (entries 10 and 11), and the amount of charge passed (entry 12).

**Table 1:**
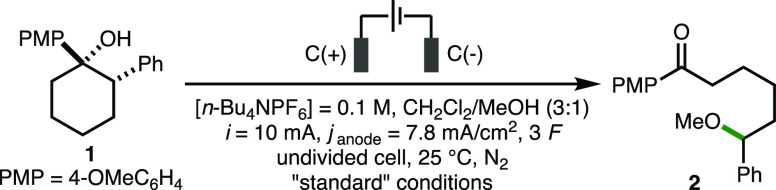
Optimization of the Electrochemical Process^a^

entry	variation from “standard” conditions	yield^b^ (%)
**1**	**none**	**67 (61)**
2	no electricity	<2
3	*E*_cell_ = 4.5 V	54
4	*i* = 7.5 mA or 12.5 mA	66, 62
5	Pt foil cathode	60
6	Pt foil or RVC as anode	56, 49
7	[*n*-Bu_4_ClO_4_] = 0.1 M as supporting electrolyte	61
8	[*n*-Bu_4_NPF_6_] = 0.05 M	45
9	[**1**] = 0.033 or 0.1 M	63,^c^ 54^d^
10	CH_2_Cl_2_/MeOH (1:1) or CH_2_Cl_2_/MeOH (5:1)	55, 41
11	MeCN/MeOH (3:1) as solvent	37
12	2 *F* or 4 *F*	49, 54

a Reactions performed with 0.3 mmol of **1** using the ElectraSyn 2.0 batch electrochemical reactor. [**1**] = 0.05 M.

b As determined by ^1^H NMR analysis of the crude reaction mixture with 1,3,5-trimethylbenzene as the internal standard. Isolated yield given in parentheses.

c **1** (0.2 mmol).

d **1** (0.6 mmol).

With the optimized electrochemical reaction conditions in hand, the substrate scope of the deconstructive methoxylation process was investigated (Scheme 2). For this purpose, the cathode material was switched from graphite to Pt foil, which was found to give consistently higher product yields across a range of substrates. Initially, it was found that a selection of substituents and functional groups were tolerated on the aromatic rings present at both the 1- and 2-positions of the cyclohexanol substrates, which gave products **2**–**14** in good yields. These included electron-releasing groups (e.g., 4-OMe), electron-withdrawing groups (e.g., 4-CF_3_, 4-CO_2_Me, and 4-CO_2_H), and halogens (e.g., 4-F, 4-Cl, and 4-Br). Cyclohexanol substrates bearing extended aromatic systems (2-naphthyl, 9-phenanthrenyl) and heteroaromatics (3-pyridyl, 2-benzothiazolyl) were also converted into the corresponding ε-methoxy ketones **15**–**18**. In addition to cyclohexanols, it was found that 4-, 5-, and 7-membered cycloalkanols also participated in deconstructive methoxylation to give products **19**–**21** in 53–77% yields. The aromatic rings at both the 1- and 2-positions of the cyclohexanol scaffold could be replaced by alkyl groups, which provided access to products **22**–**25**. Furthermore, the formation of products **24**–**27** in good yields (53–73%) illustrated that a benzylic C–H bond is not required for the electrochemical deconstructive methoxylation process to occur. Acyclic tertiary and primary homobenzylic alcohols also underwent deconstructive methoxylation to give ethers **28** and **29** in good yields, which generated acetone and formaldehyde as byproducts, respectively. Substitution of the methanol cosolvent for other alcohols, namely, *n*-butanol, benzyl alcohol, and isopropanol, enabled the formation of ether products **30**–**32** in 58–70% yields. When CH_2_Cl_2_/AcOH (23:1) was employed as a solvent mixture in combination with *n*-Bu_4_NOAc as the supporting electrolyte, 68% conversion to acetate ester **33** was observed. Similarly, benzoate ester **34** was accessed by using benzoic acid in combination with *n*-Bu_4_NOBz. Finally, pyrazole and 1,2,3-triazole could be employed as nucleophiles in combination with a MeCN/TFE (19:1) solvent mixture to access products **35** and **36** in 54% and 53% yields, respectively.

**Scheme 2:**
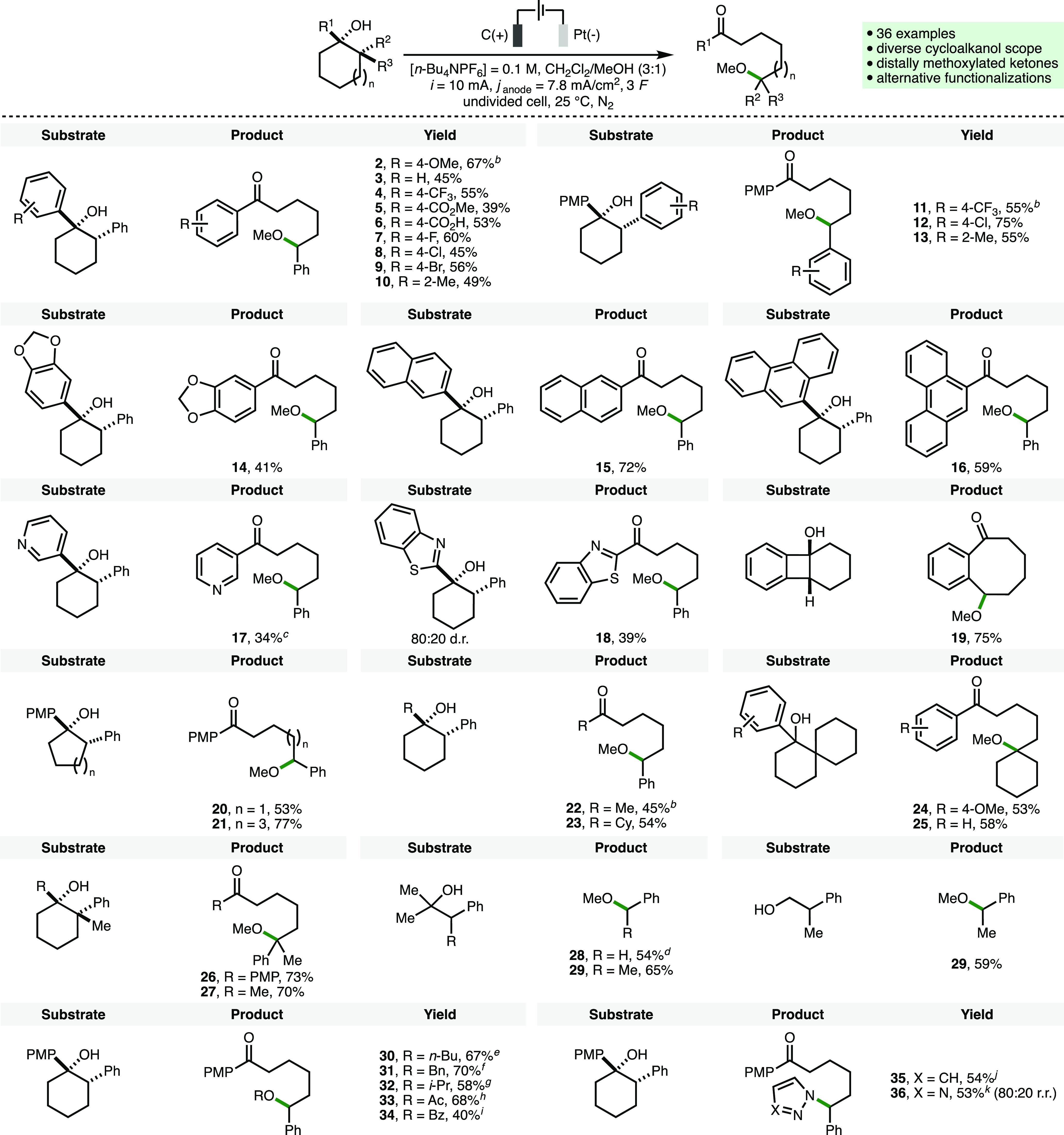
Substrate Scope of Electrochemical Deconstructive Functionalization Reactions performed with 0.3 mmol of substrate using the ElectraSyn 2.0 batch electrochemical reactor. [Substrate] = 0.05 M. All substrates are diastereomerically pure (>95:<5 d.r.), where relevant, unless otherwise noted. Yields as determined by ^1^H NMR analysis of the crude reaction mixture with 1,3,5-trimethylbenzene as the internal standard. Graphite cathode. 4.5 *F*. 0.6 mmol of substrate. [substrate] = 0.1 M. CH_2_Cl_2_/*n*-BuOH (3:1). CH_2_Cl_2_/*n*-BnOH (3:1). CH_2_Cl_2_/*i*-PrOH (3:1). CH_2_Cl_2_/AcOH (23:1), [*n*-Bu_4_NOAc] = 0.1 M. CH_2_Cl_2_, benzoic acid (6.7 equiv), [*n*-Bu_4_NOBz] = 0.1 M, 2 *F*. MeCN/TFE (19:1), pyrazole (2 equiv). MeCN/TFE (19:1), triazole (2 equiv).

To demonstrate scalability, the electrochemical deconstructive methoxylation process was performed in flow employing a syringe pump (flow rate = 2 mL/min) in combination with the commercially available Ammonite8 flow electroreactor (volume = 1 mL, *i* = 500 mA) equipped with a graphite anode and platinum plate cathode (Scheme 3).^13^ Cyclohexanol **1** (5.1 mmol) was converted to **2** in a 74% isolated yield (1.18 g) in a continuous single pass. In comparison to batch, the flow process was performed using a lower electrolyte concentration ([*n*-Bu_4_NPF_6_] = 0.05 M vs [*n*-Bu_4_NPF_6_] = 0.1 M) and increased current density (*j*_anode_ = 22 mA/cm^2^ vs *j*_anode_ = 7.8 mA/cm^2^), which resulted in higher productivity (4.1 mmol/h vs 0.08 mmol/h). The 4-methoxyphenyl ketone functionality within **2** can be readily converted to the corresponding ester or amide via Baeyer–Villiger^10^ or Beckmann^14^ rearrangements, respectively.

**Scheme 3:**
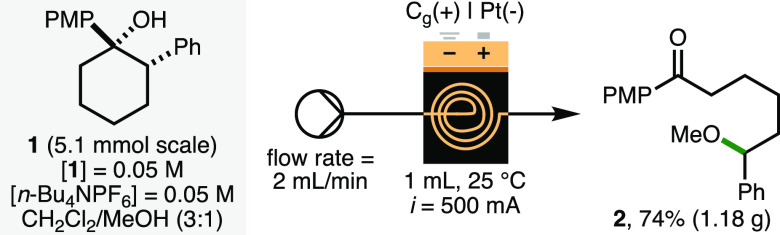
Electrochemical Scale-up in Flow

A selection of experiments was performed to gain insight into the reaction mechanism (Scheme 4A). First, using the optimized electrochemical reaction conditions, 1-(4-methoxyphenyl)cyclohexan-1-ol **37** (*E*_p/2_ = 1.03 V vs Fc/Fc^+^) did not undergo deconstructive methoxylation to give **38**, which indicated that aryl/dialkyl substitution at the 2-position of the cyclohexanol ring is required for the desired reactivity (cf., Scheme 2, products **2** and **24**). 2-Methyl substituted cyclohexanol **39** produced a complex mixture of ring-opened products in a combined 64% NMR yield, from which **40** was isolated in 16% yield. Aliphatic cyclohexanol **41** was recovered in 89% yield when subjected to the same reaction conditions, which implied that the reaction proceeds via an initial oxidation of the aromatic ring to form an aromatic radical cation at either the 1- or 2-position (cf. Scheme 2, products **22** and **25**). Methyl ether substrate **42** underwent deconstructive methoxylation to give **2** in 55% yield, which revealed that alkoxy radical intermediates are not involved in the reaction mechanism. As such, taking the formation of product **27** as a representative example, a plausible reaction mechanism initiates with single-electron anodic oxidation of the phenyl ring within the cyclohexanol substrate to give the corresponding aromatic radical cation (Scheme 4B).^15^ This species can be converted to the corresponding benzylic carbocation via hydroxyl-assisted ring opening of the unstrained six-membered ring with concomitant proton loss and single-electron anodic oxidation.^16^ Subsequent nucleophilic attack by methanol will form **27**, with the counter cathodic reaction being hydrogen gas production via proton reduction.

**Scheme 4:**
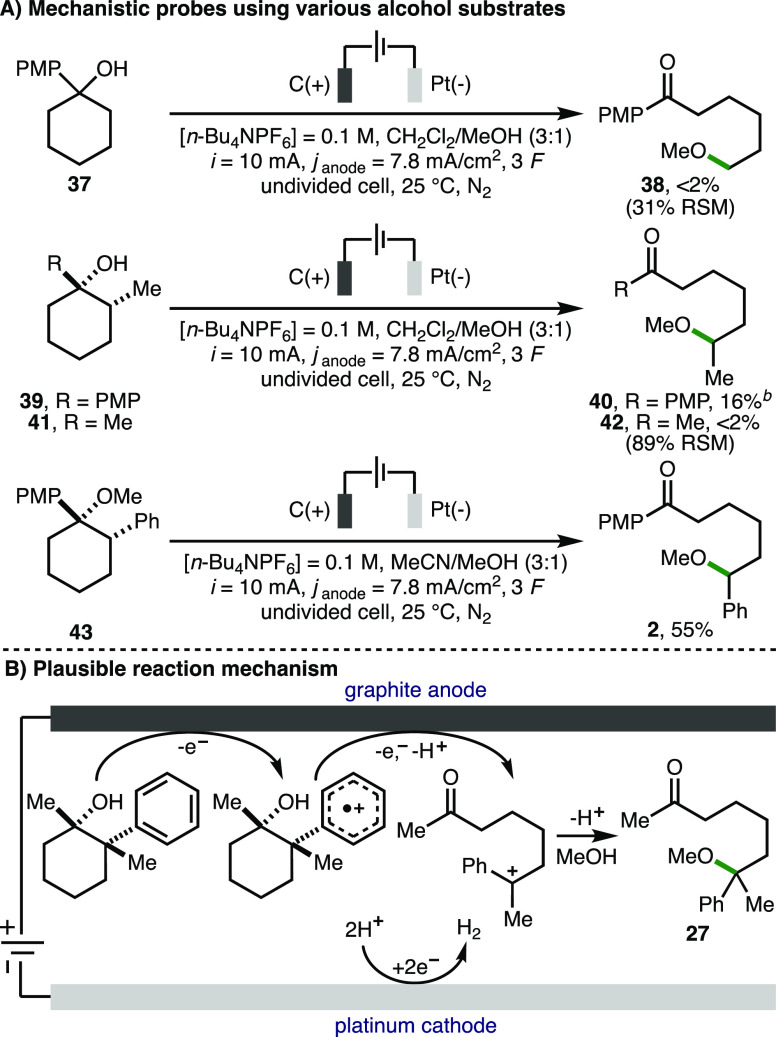
Reaction Mechanism Reactions performed with 0.3 mmol of substrate using the ElectraSyn 2.0 batch electrochemical reactor. [Substrate] = 0.05 M. All substrates are diastereomerically pure (>95:<5 d.r.), where relevant, unless otherwise noted. Yields as determined by ^1^H NMR analysis of the crude reaction mixture with 1,3,5-trimethylbenzene as the internal standard. Isolated yield.

In conclusion, an electrochemical approach for the deconstructive functionalization of cycloalkanols has been developed, where various alcohols, carboxylic acids, and *N*-heterocycles have been employed as nucleophiles. The method was demonstrated across a broad range of cycloalkanol substrates, including various ring sizes and substituents, to access useful remotely functionalized ketone products (36 examples). The method was demonstrated on a gram scale via single-pass continuous flow, which exhibited increased productivity in relation to the batch process. Ongoing work in our laboratory is focused on further applications of electrochemical deconstructive functionalization in organic synthesis.

## Data Availability

The data underlying this study are openly available in the Cardiff University data catalogue at: 10.17035/d.2022.0233339387.

## Supporting Information Available

Optimization data, experimental procedures, characterization of new compounds, and spectral data (PDF)
