# Supplementary material for: Deconstructive Functionalization of Unstrained Cycloalkanols via Electrochemically Generated Aromatic Radical Cations
Source: Org Lett. 2023 Feb 27;25(9):1486–90. doi: 10.1021/acs.orglett.3c00219 (PMC10012273; doi:10.1021/acs.orglett.3c00219)
Supplement: Supplementary file 1 — ol3c00219_si_001.pdf [file ol3c00219_si_001.pdf]

SUPPORTING INFORMATION

Deconstructive Functionalization of Unstrained Cycloalkanols *via* Electrochemically-Generated Aromatic Radical Cations

James Harnedy,[†] Hussain A. Maashi,[†] Albara A. M. A. El Gehani,[†] Matthew Burns[‡] and Louis C. Morrill*,[†]

† Cardiff Catalysis Institute, School of Chemistry, Cardiff University, Main Building, Park Place, Cardiff, CF10 3AT, U.K.

‡ Chemical Development, Pharmaceutical Technology & Development, Operations, AstraZeneca, Macclesfield, SK10 2NA, U.K.

*Email: MorrillLC@cardiff.ac.uk

Contents

General Information 3

General Procedures 5

Substrate Synthesis and Characterisation 8

Characterisation of Products 66

Electrochemical General Procedure X: 66

Mechanistic Studies: 140

Electrochemical Flow Scale Up 149

General Information 149

Gram Scale Flow Experimental 150

Proposed Mechanism through 1-aryl Ring Oxidation 152

Cyclic Voltammetry Studies of Selected Substrates 153

References 157

General Information

Unless otherwise stated, all non-electrochemical reactions were conducted in flame-dried glassware under an atmosphere of dry nitrogen or argon, sealed with septum seals and were stirred with Teflon coated magnetic stirrer bars. Unless stated otherwise, all electrochemical reactions were performed using oven-dried 10 mL ElectraSyn vials under an atmosphere of dry nitrogen, sealed with an ElectraSyn Teflon cap fitted with a graphite anode and platinum cathode and were stirred with Teflon coated magnetic stirrer bars. Dry tetrahydrofuran (THF), diethyl ether (Et_2O) and acetonitrile (MeCN) and dichloromethane (CH_2Cl_2) were obtained after passing these previously degassed solvents through activated alumina columns (Mbraun, SPS-800). Tetra-*n*-butylammonium hexafluorophosphate (n-Bu_4NPF_6) and tetra-*n*-butylammonium perchlorate (n-Bu_4NClO_4) were recrystallised from ethanol or water, respectively, and dried in the oven before use. Spectroscopic grade anhydrous methanol was purchased and used for the electrochemical methoxylation. Other alcohol solvents were dried over 3 Å sieves for 24 hours before use. All other solvents and commercial reagents were used as supplied without further purification unless stated otherwise.

All electrochemical reactions were conducted using an ElectraSyn 2.0 apparatus, purchased from IKA. Graphite, reticulated vitreous carbon (RVC) and glassy carbon (GC) electrodes were purchased from IKA and are of uniform dimensions. Graphite electrodes were used as supplied from IKA or were cut from a sheet of carbon foil (2 mm thickness) purchased from Goodfellow. The electrodes were cut to the dimension of 8 mm × 52 mm using a Startrite Bandsaw (model 18-T-5) with a Starrett, Durate SFB high carbon steel blade (2870 mm x 10 mm x 0.65 mm, 3 mm pitch, regular tooth). Graphite electrodes could be used several times by renewing the top surface of the graphite. This was achieved by scraping away the top layer with a razor blade, sonicating in MeCN for 5 minutes, followed by oven drying for 30 mins. Platinum electrodes were cut from a sheet of platinum foil (0.05 mm thickness) purchased from Goodfellow using scissors to a width of 5 mm and to a standard length of an IKA supplied electrode (52 mm). Platinum electrodes were washed with water and acetone, then burned over a Bunsen burner before every reaction. The electrodes were set up in the standard IKA supplied Electrasyn vial cap with an electrode distance of 7 mm. Reactions were stirred at 400 rpm at room temperature.

Cyclic voltammetry (CV) experiments were conducted at room temperature using an Autolab PGSTAT204, controlled using Nova 2.1 software. The working electrode was a GC disc (3 mm dia., BASi part number MF-2012), the counter electrode was a Pt-wire (BASi part number MW-4130) and a Ag/AgCl reference electrode was used (BASi part number – MF-2052). The working electrode was polished on a water wet alumina pad in a figure of 8 motion for 30 seconds before being rinsed with

deionised water and acetone. The counter electrode was rinsed with deionised water and acetone before being burned over a Bunsen burner. The scans were oxidative starting at 0.0 V, then referenced to Fc. The solvent was deoxygenated by bubbling through N_2 for 5 minutes before scan was initiated.

Room temperature (rt) refers to 20-25 °C. Ice/water and CO_2(s)/acetone baths were used to obtain temperatures of 0 °C and -78 °C respectively. All reactions involving heating were conducted using DrySyn blocks and a contact thermometer. In vacuo refers to reduced pressure through the use of a rotary evaporator.

Analytical thin layer chromatography was carried out using aluminium plates coated with silica (Kieselgel 60 F254 silica) and visualisation was achieved using ultraviolet light (254 nm), followed by 3 staining with a 1% aqueous $KMnO_4$ solution, or a 10% w/v solution of phosphomolybdic acid in ethanol. Flash column chromatography was performed using Kieselgel 60 silica in the solvent system stated using head-pressure by means of a compressed air line.

Melting points were recorded on an a Gallenkamp melting point apparatus and are reported corrected by linear calibration to benzophenone (47 - 49 °C) and benzoic acid (121 - 123 °C). Infrared spectra were recorded on a Shimadzu IRAffinity-1 Fourier Transform ATR spectrometer as thin films using a Pike MIRacle ATR accessory. The most intense peaks and structurally important peaks are quoted. Absorption maxima (vmax) are recorded in wavenumbers (cm^{-1}). ^1H, ^{13}C and ^{19}F NMR spectra were obtained on a Bruker Avance 300 (300 MHz ^1H, 75 MHz ^{13}C), Bruker Avance 400 (400 MHz ^1H, 101 MHz ^{13}C, 376 MHz ^{19}F) or a Bruker Avance 500 (500 MHz ^1H, 126 MHz ^{13}C, 471 MHz ^{19}F) spectrometer at rt in the solvent stated. Chemical shifts are reported in parts per million (ppm) relative to the residual solvent signal. All coupling constants, J, are quoted in Hz. Multiplicites are reported with the following symbols: br = broad, s = singlet, d = doublet, t = triplet, q = quartet, m = multiplet and combinations of these were used to denote higher order multiplicities. High resolution mass spectrometry (HRMS, m/z) data was acquired at Cardiff University. EI/CI HRMS data was collected on a Thermo Scientific Exactive GC machine with an orbitrap mass analyser. ES HRMS data was collected on a Walters Xevo G2XS machine with a TOF (Time of Flight) mass analyser.

“Petrol” and ‘’hexanes’’ refers to the fraction boiling in the range of 40-60 °C unless otherwise stated.

General Procedures

General Procedure A

Part A: Synthesis of 2-arylketone:

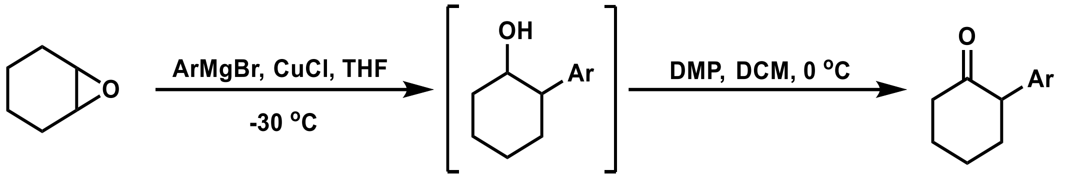

To a flame dried 3 necked flask was added magnesium (1.4 equiv.), iodine (1 crystal) and THF (0.5 M with respect to arylbromide). Arylbromide (1.2 equiv.) was added portionwise at room temperature, and then the mixture was heated to reflux for 1-2 h. The mixture was cooled to room temperature and Cu(I)Cl (0.1 equiv.) was added before cooling to -30 °C. Cyclohexene oxide (1 equiv.) was added dropwise and then the mixture was stirred at -30 °C for 2 h before being allowed to stir at room temperature overnight. The reaction was then quenched with a saturated solution of NH_4Cl. The mixture was extracted with EtOAc (x 2), and the organics combined and dried over $MgSO_4$, filtered and concentrated *in vacuo* yielding crude 2-arylcyclohexan-1-ol. The crude 2-arylcyclohexan-1-ol (1 equiv.) was diluted in CH_2Cl_2 (0.5 M with respect to 2-arylcyclohexan-1-ol) and cooled to 0 °C. Dess Martin Periodinane (DMP) (1.1 equiv.) was added and the mixture was stirred up to room temperature overnight. The reaction mixture was poured onto a cold solution of potassium carbonate and the suspension was stirred for 30 minutes and then filtered. The layers of the filtrate were separated and the aqueous was extracted with CH_2Cl_2 (x 2). The organics were combined and dried over $MgSO_4$, filtered and concentrated *in vacuo* affording crude 2-arylcyclohexanone. If required the crude residue was purified by flash column chromatography (eluent = 10 to 20% EtOAc in hexanes, silica gel) to afford product and used in the next step.

General Procedure A Part B(1)

Addition of Grignard reagent to 2-arylketone:

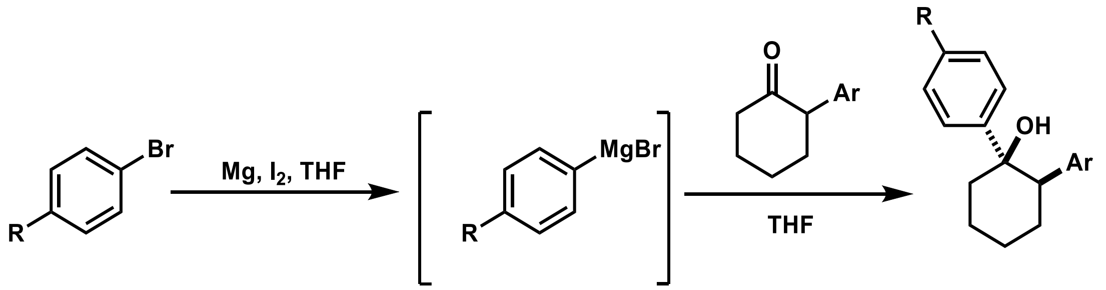

To a flame dried flask was added magnesium (1.8-2.4 equiv.), iodine (1 crystal) and THF (0.5 M with respect to arylbromide). Arylbromide (1.5-2.0 equiv.) was added portionwise and the resultant mixture was heated at reflux for 1-2 h. The solution was cooled to room temperature and 2-arylcyclohexanone (1 equiv.) in THF (1 M with respect to 2-arylcyclohexanone) was added and the mixture was stirred overnight. The mixture was quenched with H_2O/saturated NH_4Cl solution (4:1) and then extracted with EtOAc (x 2). The layers were separated, and the organics were dried over $MgSO_4$, filtered, and concentrated *in vacuo* yielding crude product. The crude residue was purified by flash column chromatography (eluent = EtOAc in hexanes, silica gel) to afford product.

Part B(2)

of Aryllithium to 2-arylketone:

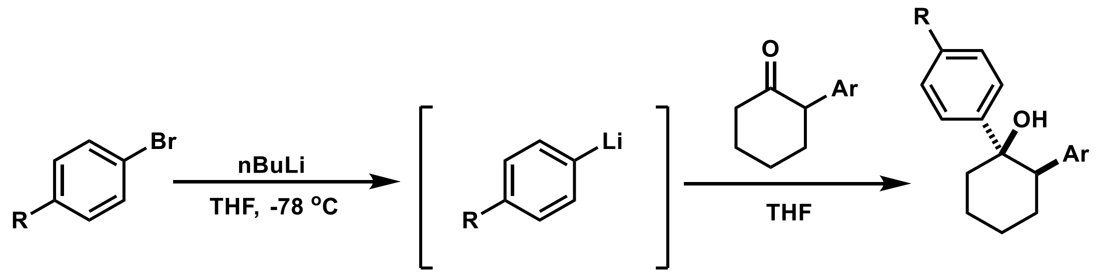

To a flame dried flask was added arylbromide (1.5 equiv.) and THF (0.3 M wrt arylbromide) and the solution was cooled to -78 °C with an acetone/dry ice bath, after which *n*-butyllithium (1.5 equiv., 2.2 M solution in hexanes) was added dropwise. The mixture was stirred at -78 °C for 1 h before a solution of 2-arylketone (1 equiv.) in THF (1M wrt to ketone) was added. The reaction mixture was allowed to stir up to room temperature and stirred for 3-4 h. The mixture was then quenched with water and extracted with EtOAc (x 2). The combined organics were dried over $MgSO_4$, filtered and concentrated

in vacuo yielding crude product. The crude residue was purified by flash column chromatography (eluent = EtOAc in hexanes, silica gel) to afford product.

General Procedure B

Addition of Commercial Grignard Reagents to Ketones:

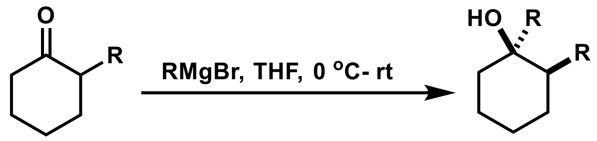

To cycloalkanone (1 equiv.) in THF (0.5 M with respect to cycloalkanone) at 0 °C was added commercial Grignard reagent (1.5-2.0 equiv.) dropwise. The reaction mixture was allowed to warm to room temperature and left to stir overnight. The mixture was quenched with H_2O/saturated NH_4Cl solution (4:1) and then extracted with EtOAc (x 2). The layers were separated, and the organics were dried over $MgSO_4$, filtered and concentrated *in vacuo* yielding crude product. The crude residue was purified by flash column chromatography (eluent = EtOAc in hexanes, silica gel) to afford product.

General Procedure C

Synthesis of spiro[5.5]undecan-1-ols:

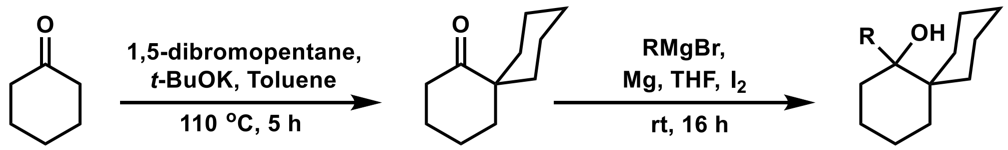

Part A:

To a solution of cyclohexanone (1 equiv.) in toluene (0.5 M) was added potassium tert-butoxide (2.1 equiv.) and 1,5-dibromopentane (1 equiv.). The mixture was heated at reflux for 5 h before being cooled to room temperature. The reaction was quenched with HCl (0.5 M) and extracted with EtOAc. The layers were separated and the aqueous extracted with EtOAc (x 2). The organics were combined,

dried over $MgSO_4$, filtered and concentrated *in vacuo* yielding crude product. The crude oil was passed through a silica plug (eluent = 20% EtOAc in hexanes) affording crude product as a colourless oil.

Part B:

To a flame dried flask was added magnesium (1.8 equiv.), iodine (1 crystal) and THF (0.5 M). Arylbromide (1.5 equiv.) was added portionwise and the resultant mixture was heated at reflux for 2 h. The solution was cooled to room temperature and spiro[5.5]undecan-1-one (1 equiv.) in THF (1 M wrt to ketone) was added and the mixture was stirred overnight. The mixture was quenched with H_2O/saturated NH_4Cl solution (4:1) and then extracted with EtOAc. The layers were separated, aqueous extracted with EtOAc (x 2), organics combined and dried over $MgSO_4$, filtered and concentrated *in vacuo* yielding crude product. The crude residue was purified by flash column chromatography (eluent = EtOAc in hexanes, silica gel) to afford product.

Substrate Synthesis and Characterisation

(1a)

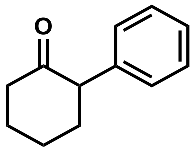

Prepared according to General Procedure A, Part A using magnesium (1.44 g, 60 mmol), iodine (1 crystal), THF (100 mL), bromobenzene (7.85 g, 50 mmol), CuCl (395 mg, 4.2 mmol) and cyclohexene oxide (4.12 g, 42 mmol). The crude secondary alcohol (42 mmol) was used directly in the next step with DMP (19.6 g, 46 mmol) and CH_2Cl_2 (85 mL). The crude residue was purified by flash column chromatography (eluent = 10 to 20% EtOAc in hexanes, silica gel) to afford product as a white solid (4.88 g, 67% yield).

Mp.: 41-43 °C **R_f** = 0.36 (eluent = 25% EtOAc in hexanes); **^{1}H NMR (500 MHz, $CDCl_3$)** δ_H: 1.75-1.91 (2H, m), 1.97-2.09 (2H, m), 2.09-2.20 (1H, m), 2.23-2.34 (1H, m), 2.40-2.59 (2H, m), 3.62 (1H, dd, *J* 12.3, 5.3 Hz), 7.11-7.19 (2H, m), 7.23-7.30 (1H, m), 7.30-7.38 (2H, m); **^{13}C NMR (126 MHz, $CDCl_3$)** δ_C: 25.5, 28.0, 35.2, 42.3, 57.5, 127.0, 128.5, 128.7, 138.9, 210.4.

Data consistent with the literature[1]

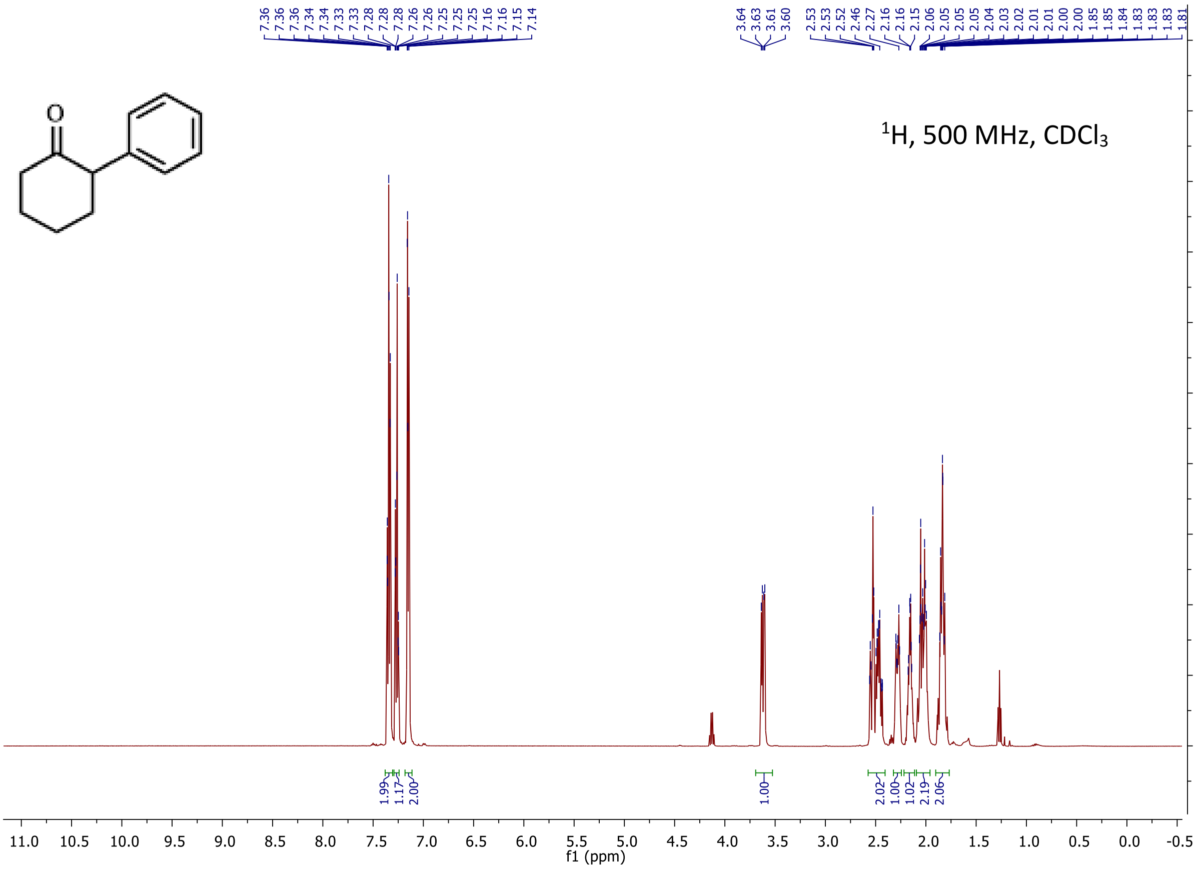

1H, 500 MHz, CDCl3
f1 (ppm)

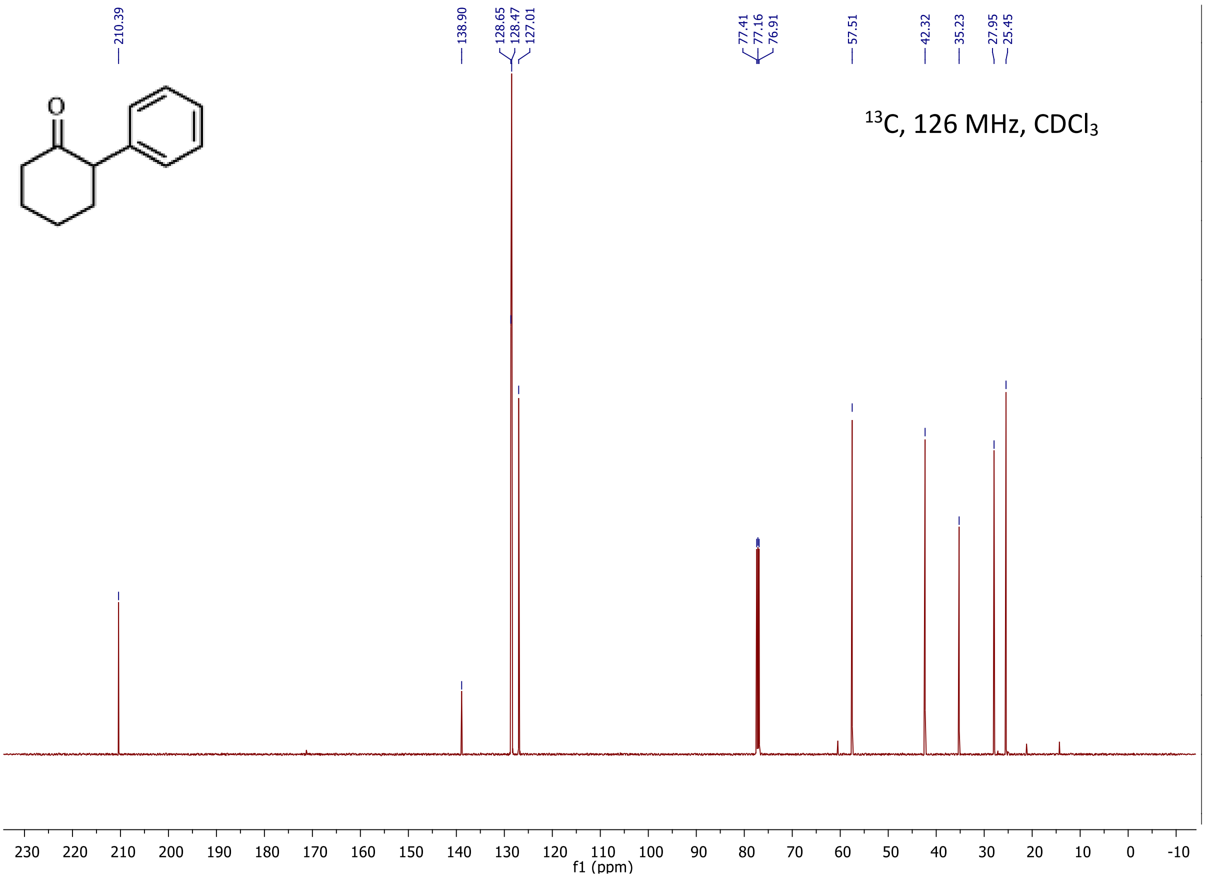

13C, 126 MHz, CDCl3
210.39
138.90
128.65
128.47
127.01
77.41
77.16
76.91
57.51
42.32
35.23
27.95
25.45
f1 (ppm)

(1)

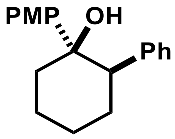

Prepared according to General Procedure A, part B(1) using magnesium (910 mg, 38 mmol), iodine (1 crystal), THF (65 mL), 4-bromoanisole (6.00 g, 30 mmol) and 2-phenylcyclohexanone (3.48 g, 20 mmol). The crude residue was purified by flash column chromatography (eluent = 5 to 15% EtOAc in hexanes, silica gel) to afford product as a white solid (4.61 g, 82% yield).

Mp.: 43-45 °C; $\mathbf{R_f}$ = 0.29 (eluent = 20% EtOAc in hexanes); $\mathbf{\nu_{max}}$ **/ cm^{-1}** (thin film) 3456, 2926, 1611, 1512, 1445, 1356, 1246, 1180; **^{1}H NMR (500 MHz, $CDCl_3$)** δ_H: 1.67-1.80 (3H, m), 1.81-1.89 (2H, m), 1.91-1.98 (1H, m), 2.00-2.08 (1H, m), 2.17-2.30 (1H, m), 3.00 (1H, dd, *J* 12.8, 3.6 Hz), 6.72 (2H, d, *J* 8.7 Hz), 6.89-6.94 (2H, m), 7.05-7.10 (3H, m), 7.11 (2H, d, *J* 8.7 Hz); **^{13}C NMR (126 MHz, $CDCl_3$)** δ_C: 22.2, 26.7, 28.6, 40.7, 53.1, 55.3, 75.6, 113.1, 126.0, 126.3, 127.8, 129.1, 140.3, 141.9, 158.0; **HRMS (ES$^+$)** [$C_{19}H_{22}O_2$] requires $[M]^+$ 282.1620, found 282.1617 (-1.1 ppm).

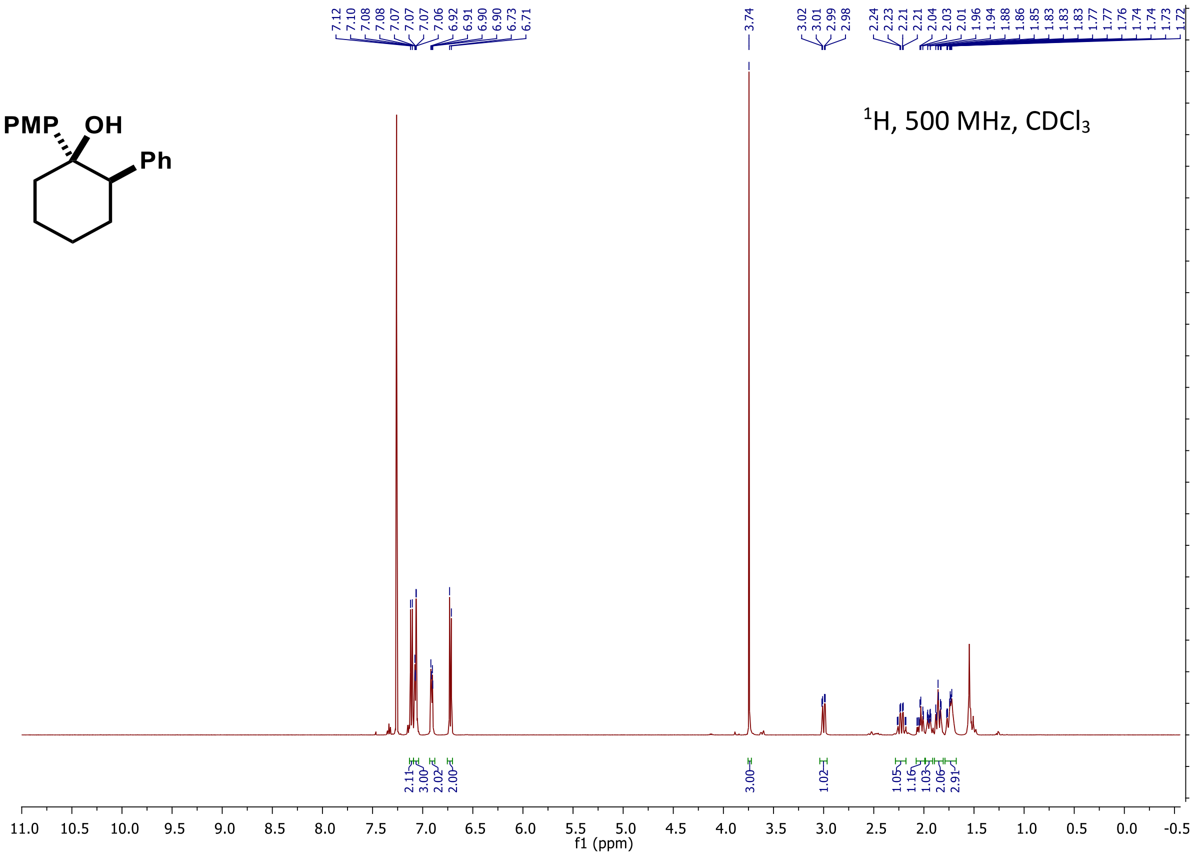
PMP
OH
Ph
1H, 500 MHz, CDCl3
f1 (ppm)

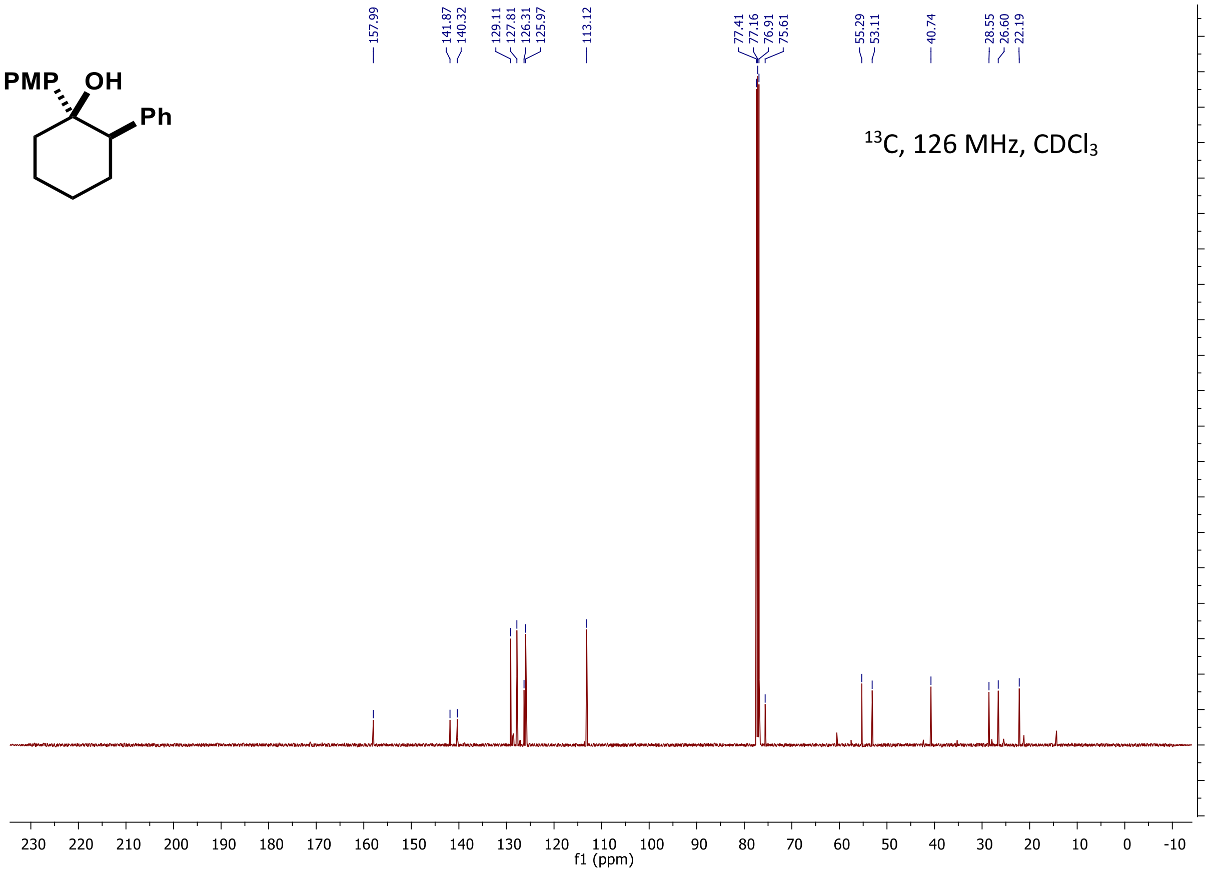
PMP
OH
Ph
13C, 126 MHz, CDCl3
f1 (ppm)

(S3)

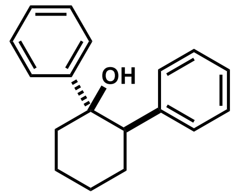

Prepared according to General Procedure A, part B(1) using magnesium turnings (219 mg, 9 mmol), iodine (1 crystal), bromobenzene (0.80 mL, 7.5 mmol), and 2- phenylcyclohexan-1-one (871 mg, 5 mmol). The crude residue was purified by flash column chromatography (5 to 10% EtOAc in hexanes, silica gel) to afford product as a white solid (0.92 g, 72%).

Mp.: 66-68 °C; $\mathbf{R_f}$ = 0.41 (eluent = 10% EtOAc in hexanes); $\mathbf{v_{max}}$ **/ cm**$^{-1}$ (thin film) 3561, 2940, 1601, 1490, 1443, 1180; **^{1}H NMR (500 MHz, CDCl$_3$)** δ_H: 1.53 – 1.57 (2H, m), 1.74 - 1.78 (1H, m), 1.85-1.91 (3H, m), 2.04-2.08 (1H, m), 2.23-2.26 (1H, m), 3.04-3.07 (1H, dd, *J* 12.8, 3.7 Hz), 6.89-6.91 (2H, m), 7.04-7.07 (3H, m), 7.07-7.15 (1H, m), 7.18-7.23 (4H, m); **^{13}C NMR (101 MHz, CDCl$_3$)** δ: 22.0, 26.4, 28.3, 40.4, 52.8, 75.8, 124.7, 126.2, 126.2, 127.6, 127.7, 128.9, 141.5, 147.8; **HRMS (CI$^+$)** [$C_{18}H_{20}O$] requires [M]$^+$ 252.1514, found 252.1511 (-1.2 ppm).

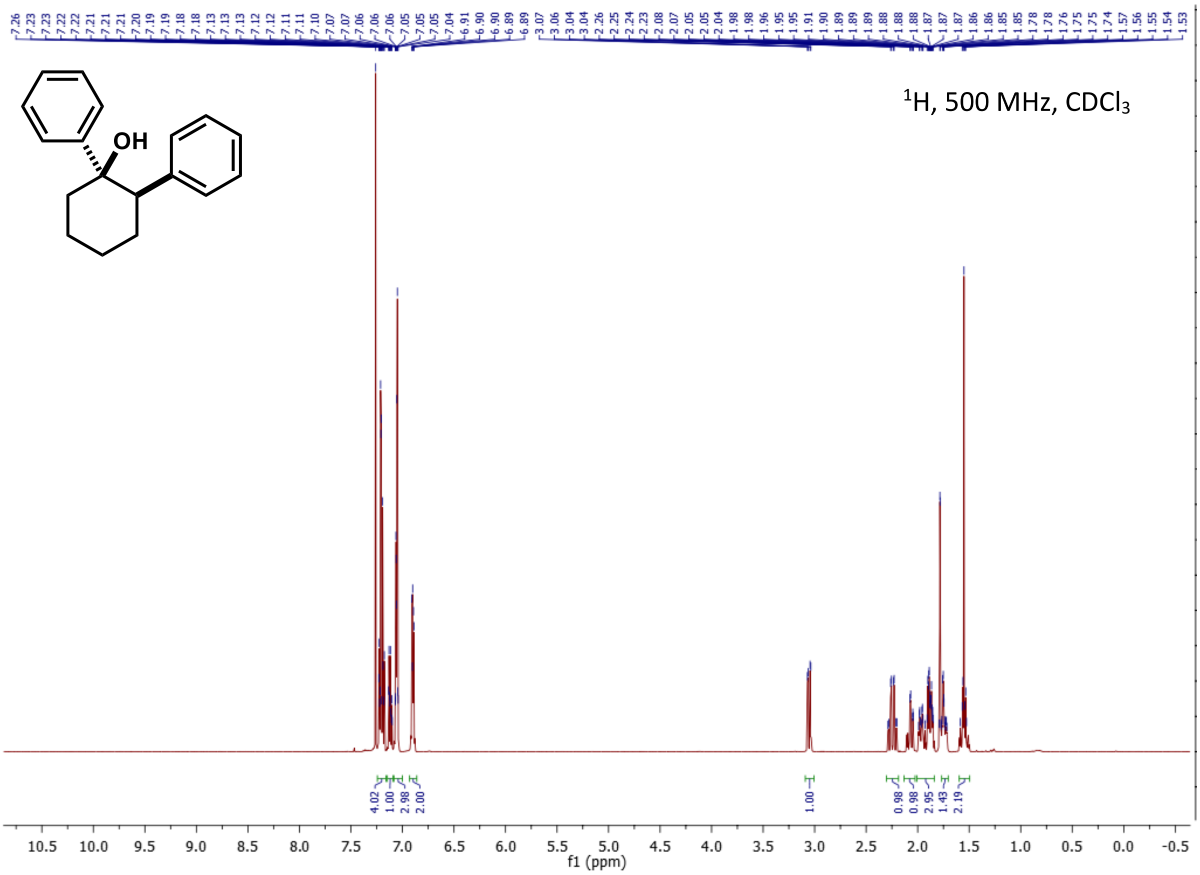

1H, 500 MHz, CDCl3
OH
4.02
1.00
2.98
2.00
1.00
0.98
0.98
2.95
1.43
2.19
f1 (ppm)

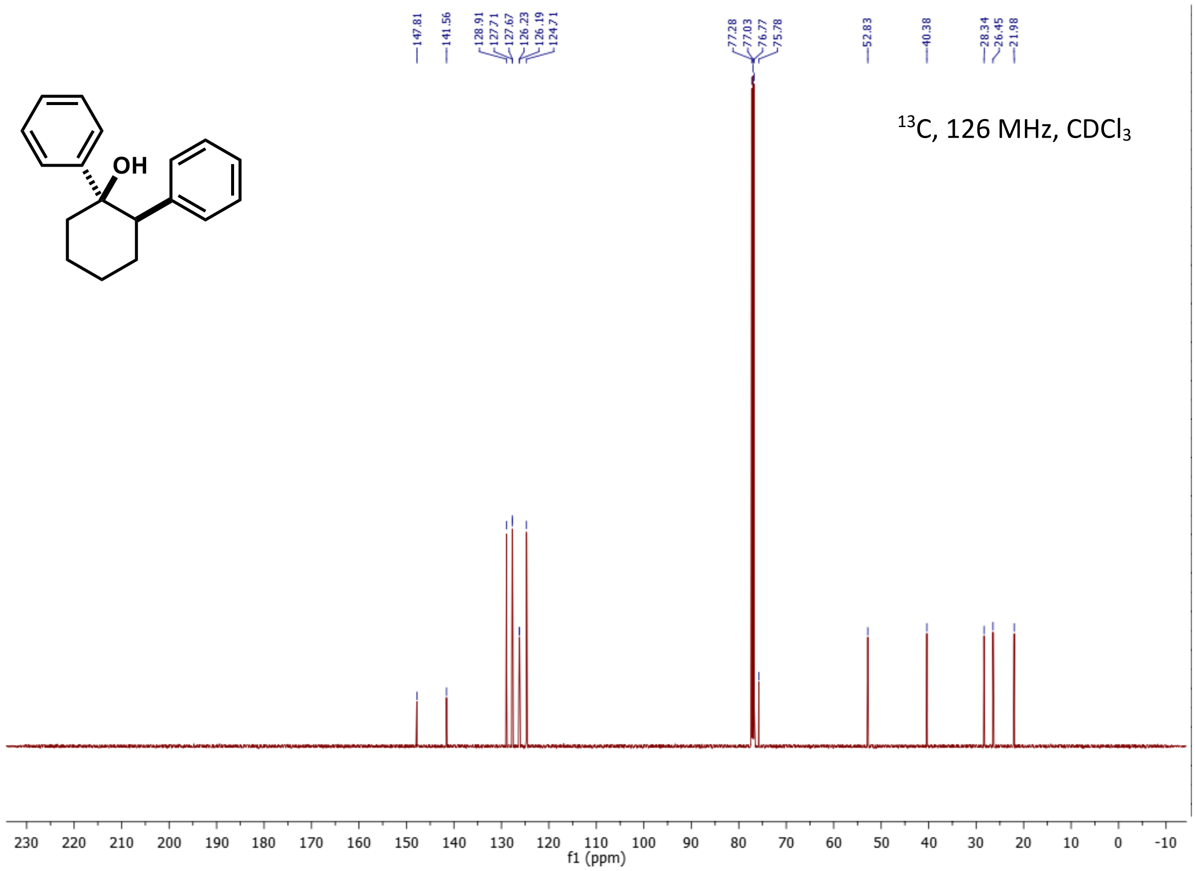

13C, 126 MHz, CDCl3
OH
147.81
141.56
128.91
127.71
127.67
126.23
126.19
124.71
77.28
77.03
76.77
75.78
52.83
40.38
28.34
26.45
21.98
f1 (ppm)

(S4)

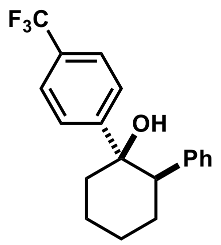

Prepared according to general procedure A, part B(1) using 2-phenylcyclohexanone (1.74 g, 10 mmol), 4-bromobenzotrifluoride (4.50 g, 20 mmol), magnesium turnings (583 mg, 24 mmol) and THF (40 mL). The crude residue was purified by flash column chromatography (eluent = 10 to 20% EtOAc in hexanes, silica gel) to afford product as an orange solid (690 mg, 22% yield).

Mp.: 50-52 °C; **R_f** = 0.44 (eluent = 25% EtOAc in hexanes); **v_{max} / cm^{-1}** (thin film) 3541, 2920, 1618, 1497, 1329, 1103, 1070; **^1H NMR (500 MHz, $CDCl_3$)** δ_H: 1.50-1.63 (1H, m), 1.73-1.92 (5H, m), 1.96-2.02 (1H, m), 2.01-2.10 (1H, m), 2.24 (1H, app qd, *J* 12.9, 3.6), 3.05 (1H, dd, *J* 12.8, 3.7 Hz), 6.83-6.93 (2H, m), 7.04-7.11 (3H, m), 7.35 (2H, d, *J* 8.2 Hz), 7.45 (2H, d, *J* 8.2 Hz); **^{13}C NMR (126 MHz, $CDCl_3$)** δ_C: 21.8, 26.3, 28.3, 40.2, 52.7, 75.8, 124.3 (q, *J* 265 Hz), 124.7 (q, *J* 3.8 Hz), 125.2, 126.5, 127.9, 128.8, 141.0, 151.9; **^{19}F NMR (471 MHz, $CDCl_3$)** δ_F: -62.3; **HRMS (CI^+)** [$C_{19}H_{19}OF_3$] requires $[M]^+$ 320.1388, found 320.1385 (- 0.9 ppm).

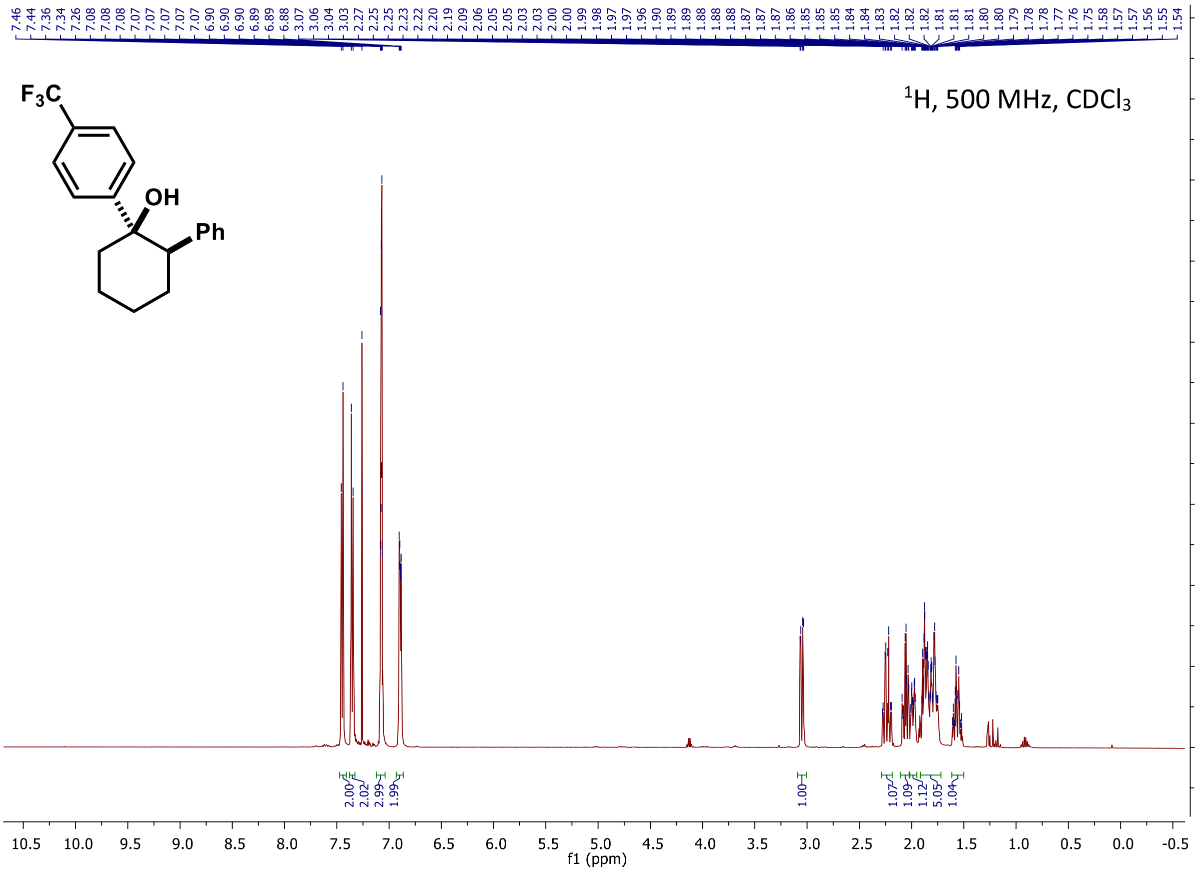

F3C
OH
Ph
1H, 500 MHz, CDCl3
f1 (ppm)

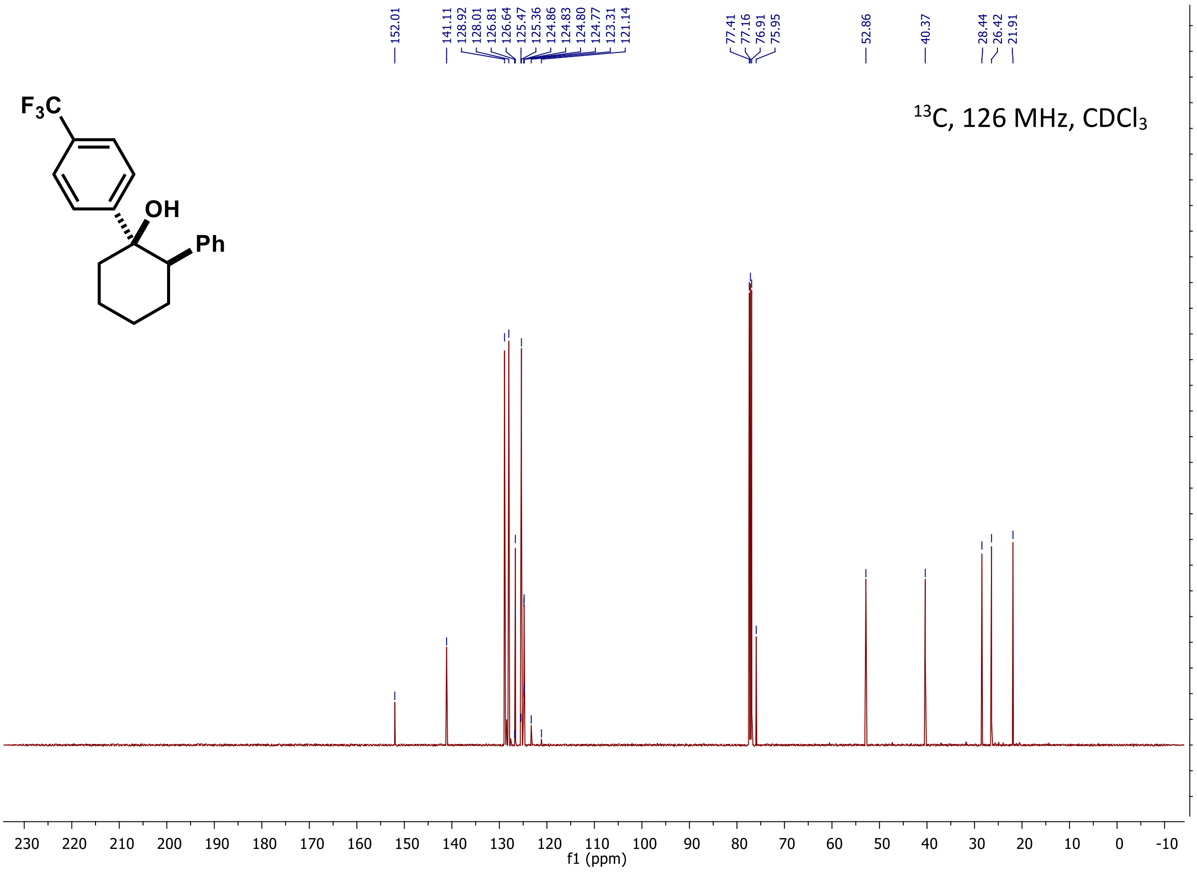

F3C
OH
Ph
13C, 126 MHz, CDCl3
f1 (ppm)

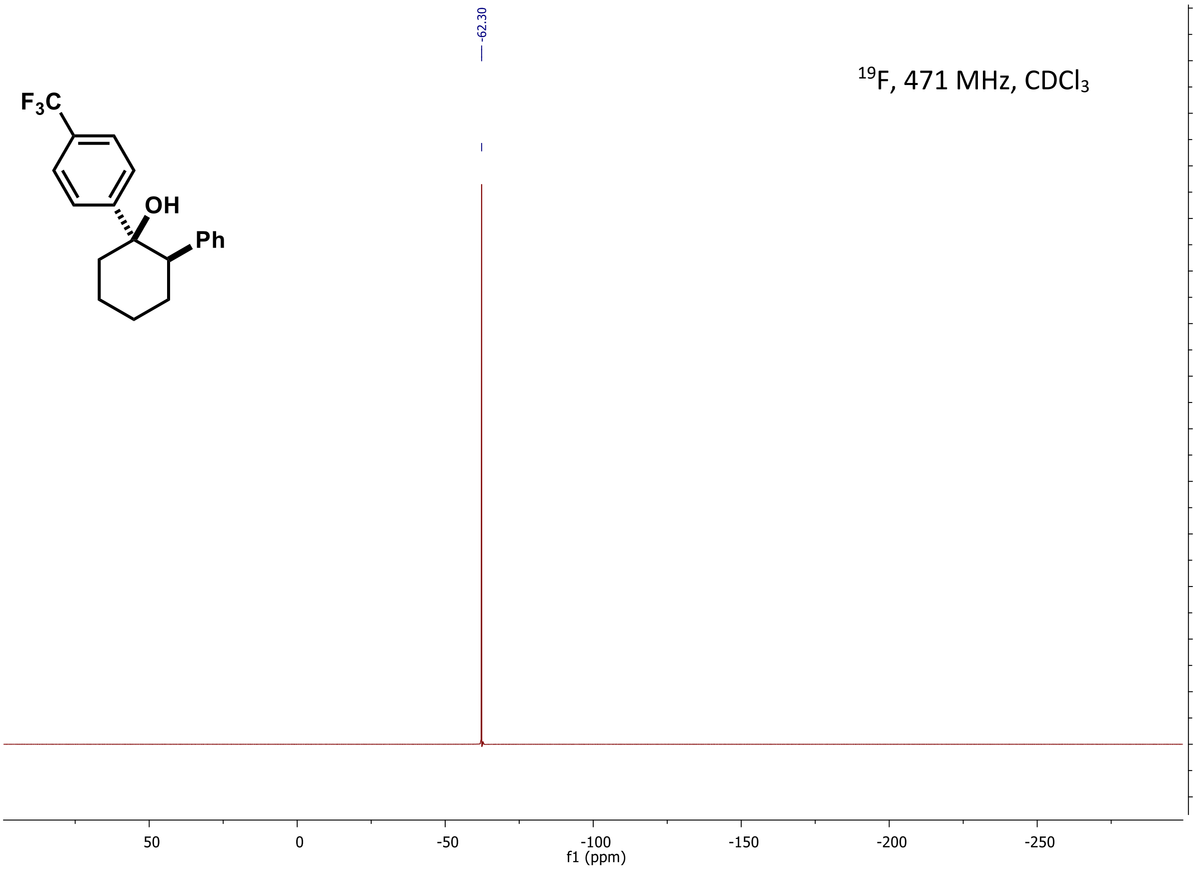

F3C
OH
Ph
-62.30
19F, 471 MHz, CDCl3
50
0
-50
-100
f1 (ppm)
-150
-200
-250

(S5)

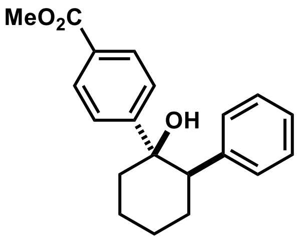

Substrate (**S6**) (296 mg, 1 mmol, 1 equiv.) was dissolved in DMF (5 mL) and potassium carbonate (166 mg, 1.2 mmol, 1.2 equiv.) was added. The suspension was stirred at room temperature for 15 minutes. Methyl iodide (150 mg, 1.1 mmol, 1.1 equiv.) was added and the mixture was stirred for 4 h. The reaction was quenched with H_2O (20 mL) and extracted with EtOAc (20 mL). The layers were separated and the aqueous was extracted with EtOAc (2 x 20 mL). The organics were combined, dried over $MgSO_4$, filtered and concentrated *in vacuo* yielding crude product. The crude residue was purified by flash column chromatography (eluent = 10% EtOAc in hexanes, silica gel) to afford product as a colourless oil (300 mg, 96% yield).

R_f = 0.49 (eluent = 20% EtOAc in toluene); **v_{max} / cm^{-1}** (thin film) 3505, 2934, 1705, 1608, 1435, 1277, 1186, 1105; **^{1}H NMR (500 MHz, $CDCl_3$)** δ_H: 1.49-1.63 (1H, m), 1.73-1.82 (2H, m), 1.83-1.91 (3H, m), 1.94-2.00 (1H, m), 2.02-2.10 (1H, m), 2.23 (1H, qd, *J* 13.1, 3.6 Hz), 3.06 (1H, dd, *J* 12.8, 3.7 Hz), 3.88 (3H, s), 6.86-6.90 (2H, m), 7.03-7.08 (3H, m), 7.31 (2H, d, *J* 8.4 Hz), 7.86 (2H, d, *J* 8.4 Hz); **^{13}C NMR (126 MHz, $CDCl_3$)** δ_C: 21.9, 26.5, 28.4, 40.3, 52.1, 52.8, 76.1, 125.0, 126.6, 128.0, 128.2, 128.9, 129.3, 141.2, 153.3, 167.2; **HRMS (EI$^+$)** [$C_{20}H_{22}O_3$] requires $[M+H]^+$ 311.1647, found 311.1652 (+ 1.6 ppm).

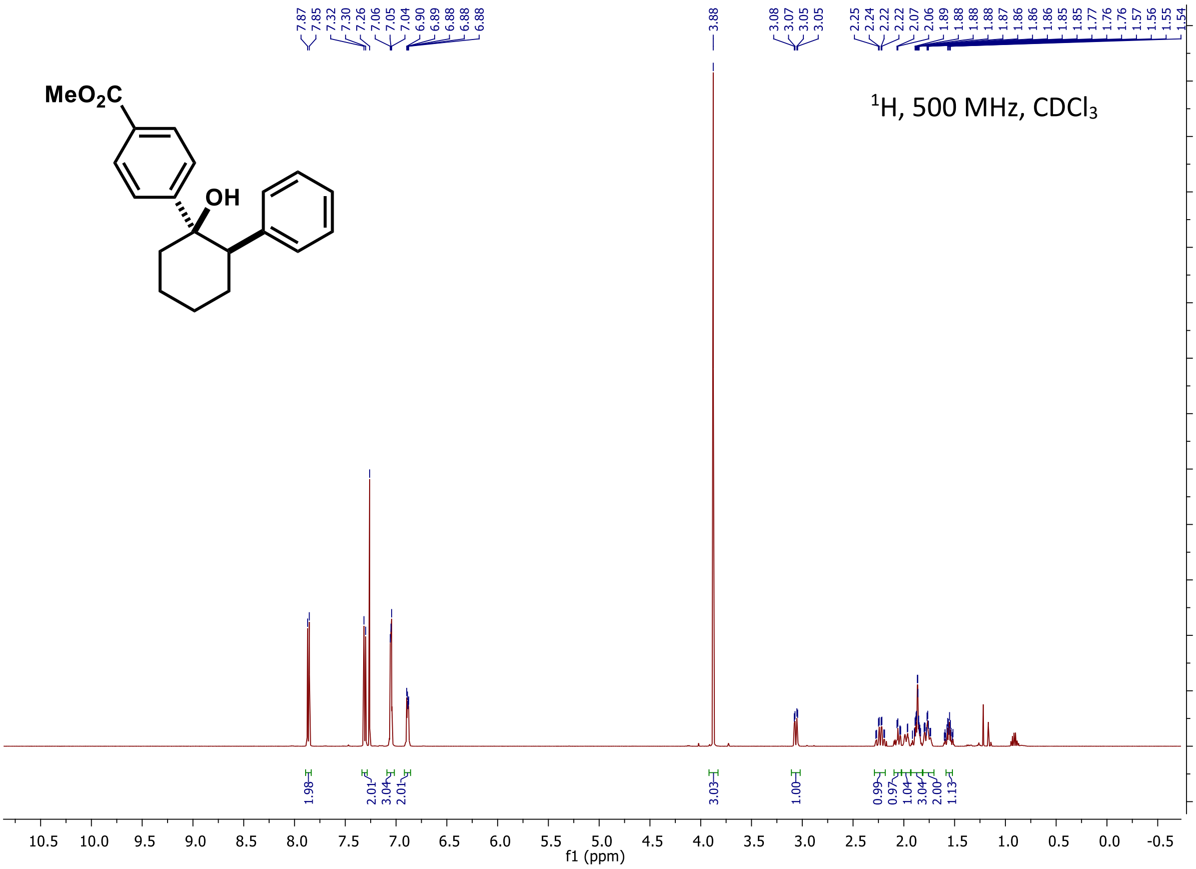
MeO2C
OH
1H, 500 MHz, CDCl3
f1 (ppm)

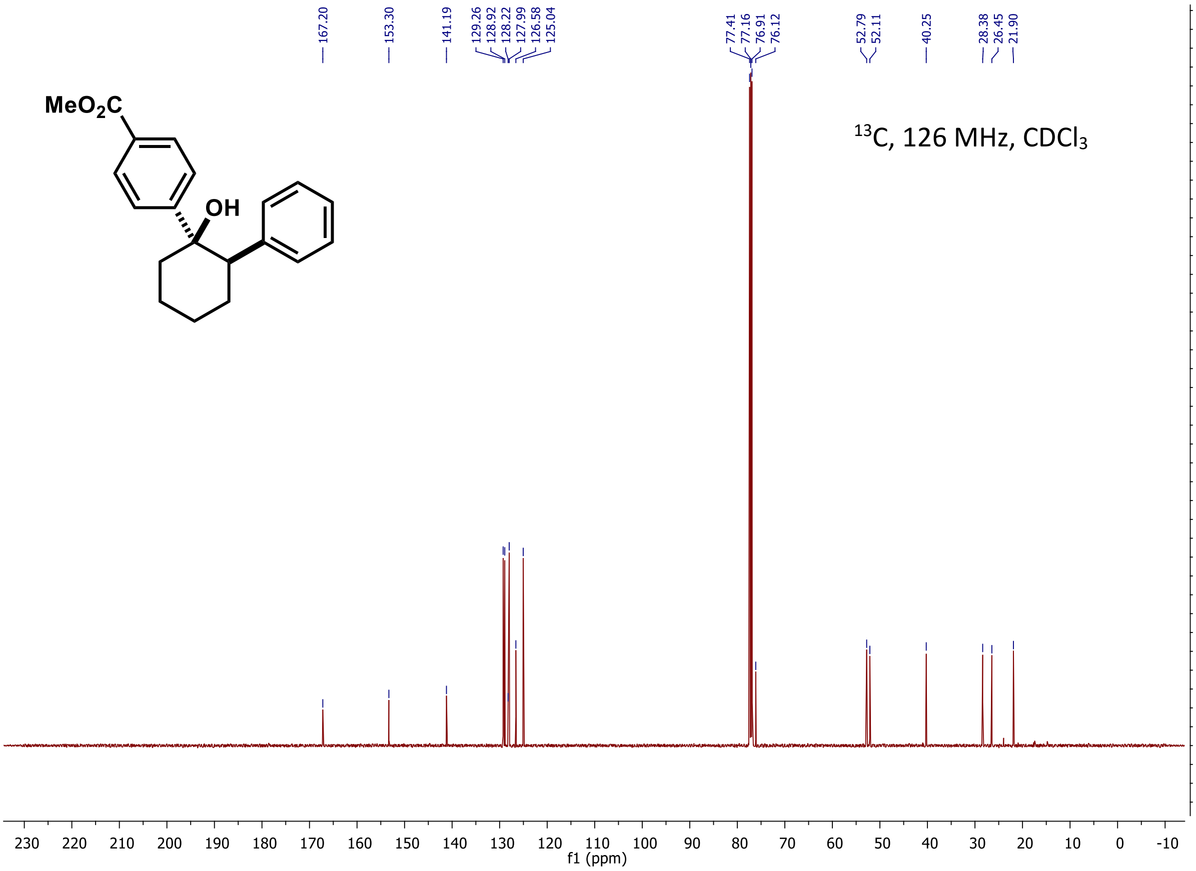
MeO2C
OH
13C, 126 MHz, CDCl3
f1 (ppm)

(S6)

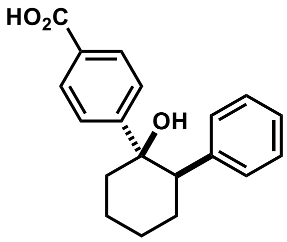

To a solution of 4-bromobenzoic acid (1.51 g, 7.5 mmol, 1 equiv.) in THF (25 mL) at -78 °C was added *n*-butyllithium (6.81 mL, 15 mmol, 2 equiv.) dropwise and the mixture was stirred at this temperature for 30 minutes. A solution of 2-phenylcyclohexanone (1.56 g, 9.0 mmol, 1.2 equiv.) in THF (9 mL) was added and the mixture was stirred up to room temperature and allowed to stir for 3 h. The reaction mixture was quenched with a saturated solution of NH_4Cl and then acidified to pH 2 with 1 M HCl. The mixture was extracted with CH_2Cl_2 (2 x 100 mL), organics combined and dried over $MgSO_4$, filtered and concentrated *in vacuo*. The crude residue was mobilised in a minimum volume of CH_2Cl_2 and precipitated with hexanes. The solid was collected by filtration, washed with hexane and dried under vacuum yielding pure product as a yellow solid (425 mg, 21% yield)

Mp.: 173-175 °C; **ν_{max} / cm^{-1}** (thin film) 3535, 2980, 1663, 1606, 1423, 1267; **^{1}H NMR (500 MHz, DMSO$_{d6}$)** δ_H: 1.51-1.63 (3H, m), 1.69 (1H, d, *J* 13.1 Hz), 1.77-1.95 (2H, m), 1.96-2.05 (1H, m), 2.17 (1H, qd, *J* 13.2, 3.3 Hz), 2.94 (1H, dd, *J* 12.6, 2.6 Hz), 4.94-4.97 (1H, m), 6.91-7.01 (5H, m), 7.41 (2H, d, *J* 8.6 Hz), 7.68 (2H, d, *J* 8.6 Hz), 12.68 (1H, s); **^{13}C NMR (126 MHz, DMSO$_{d6}$)** δ_C: 21.3, 25.9, 29.4, 52.5, 74.8, 125.3, 125.4, 127.0, 128.0, 128.3, 129.1, 143.1, 154.3, 167.3; **HRMS (ES$^+$)** [$C_{19}H_{20}O_3$] requires [M-H_2O+H]$^+$ 279.1385, found 279.1397 (+ 4.3 ppm).

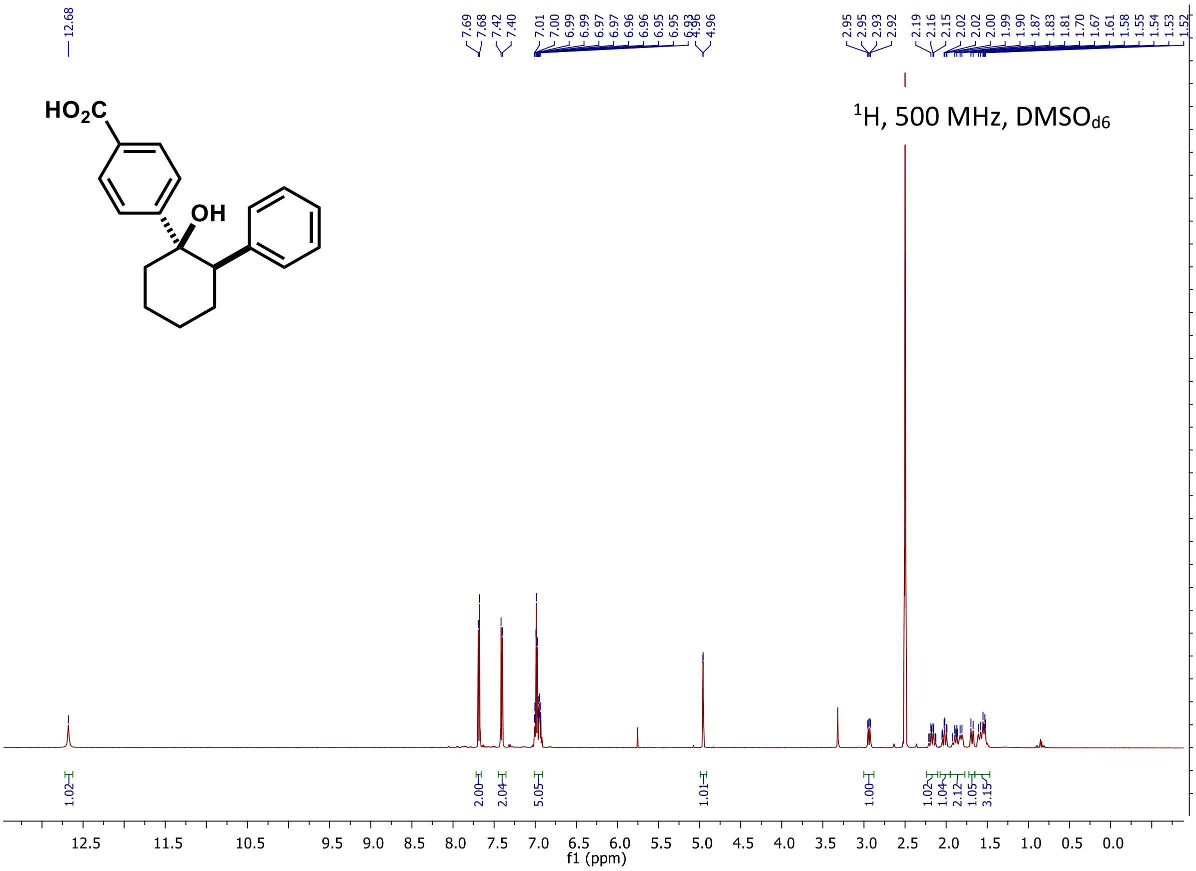

HO2C
OH
1H, 500 MHz, DMSOd6
f1 (ppm)

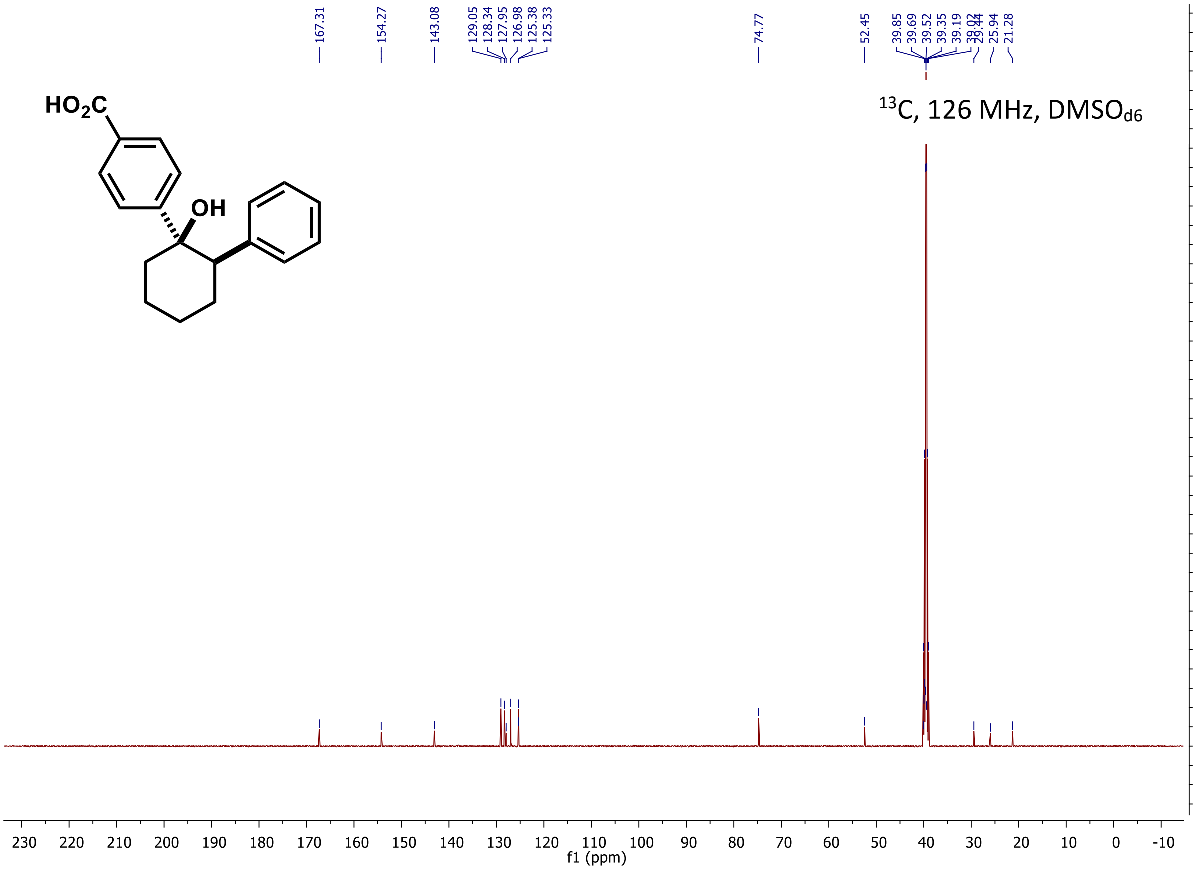

HO2C
OH
13C, 126 MHz, DMSOd6
f1 (ppm)

(S7)

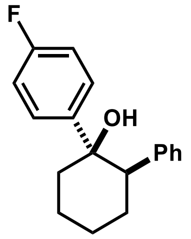

Prepared according to general procedure A, part B(1) using 2-phenylcyclohexanone (1.74 g, 10 mmol), 4-fluorobromobenzene (2.63 g, 15 mmol), magnesium turnings (432 mg, 18 mmol) and THF (30 mL). The crude residue was purified by flash column chromatography (eluent = 5 to 10% EtOAc in hexanes, silica gel) to afford product as a white solid (2.00 g, 74% yield).

Mp.: 60-61 °C; **R_f** = 0.48 (eluent = 20% EtOAc in hexanes); **ν_{max} / cm^{-1}** (thin film) 3547, 2924, 2850, 1604, 1506, 1442, 1224, 1161; **^{1}H NMR (500 MHz, $CDCl_3$)** δ_H: 1.47-1.60 (1H, m), 1.72-1.81 (3H, m), 1.84-1.92 (2H, m), 1.93-2.01 (1H, m), 2.01-2.09 (1H, m), 2.17-2.28 (1H, m), 2.99 (1H, dd, *J* 12.8, 3.5 Hz), 6.83-6.93 (4H, m), 7.02-7.12 (3H, m), 7.14-7.20 (2H, m); **^{13}C NMR (126 MHz, $CDCl_3$)** δ_C: 22.1, 26.5, 28.5, 40.6, 53.2, 75.7, 114.5 (d, *J* 21.4 Hz), 126.5, 126.6 (d, *J* 8.8 Hz), 127.9, 129.0, 141.5, 143.7 (d, *J* 2.5 Hz), 161.9 (d, *J* 244.4 Hz); **^{19}F NMR (471 MHz, $CDCl_3$)** δ_F: -117.37— -117.4 (m); **HRMS (CI$^+$)** [$C_{18}H_{19}OF$] requires $[M]^+$ 270.1420, found 270.1417 (-1.1 ppm).

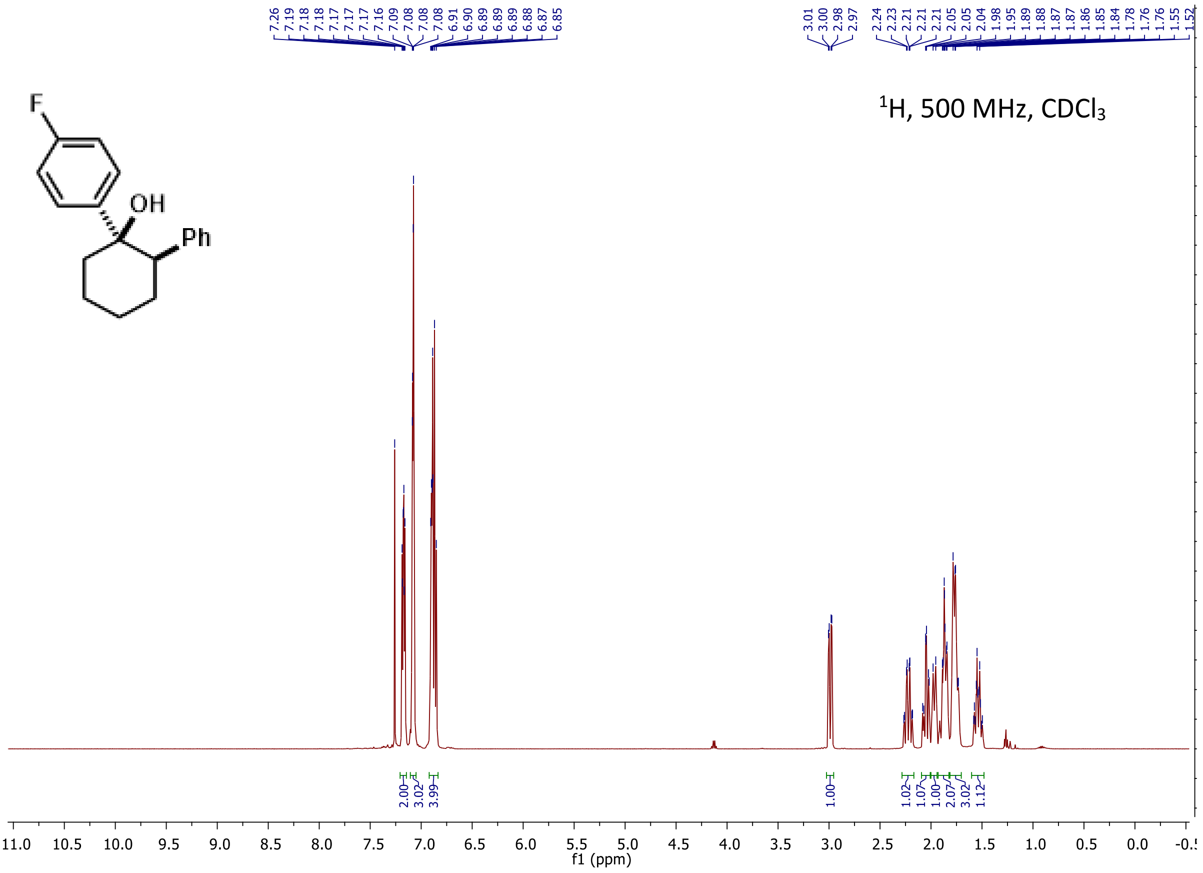

^{1}H, 500 MHz, CDCl$_3$
OH
Ph
F
f1 (ppm)

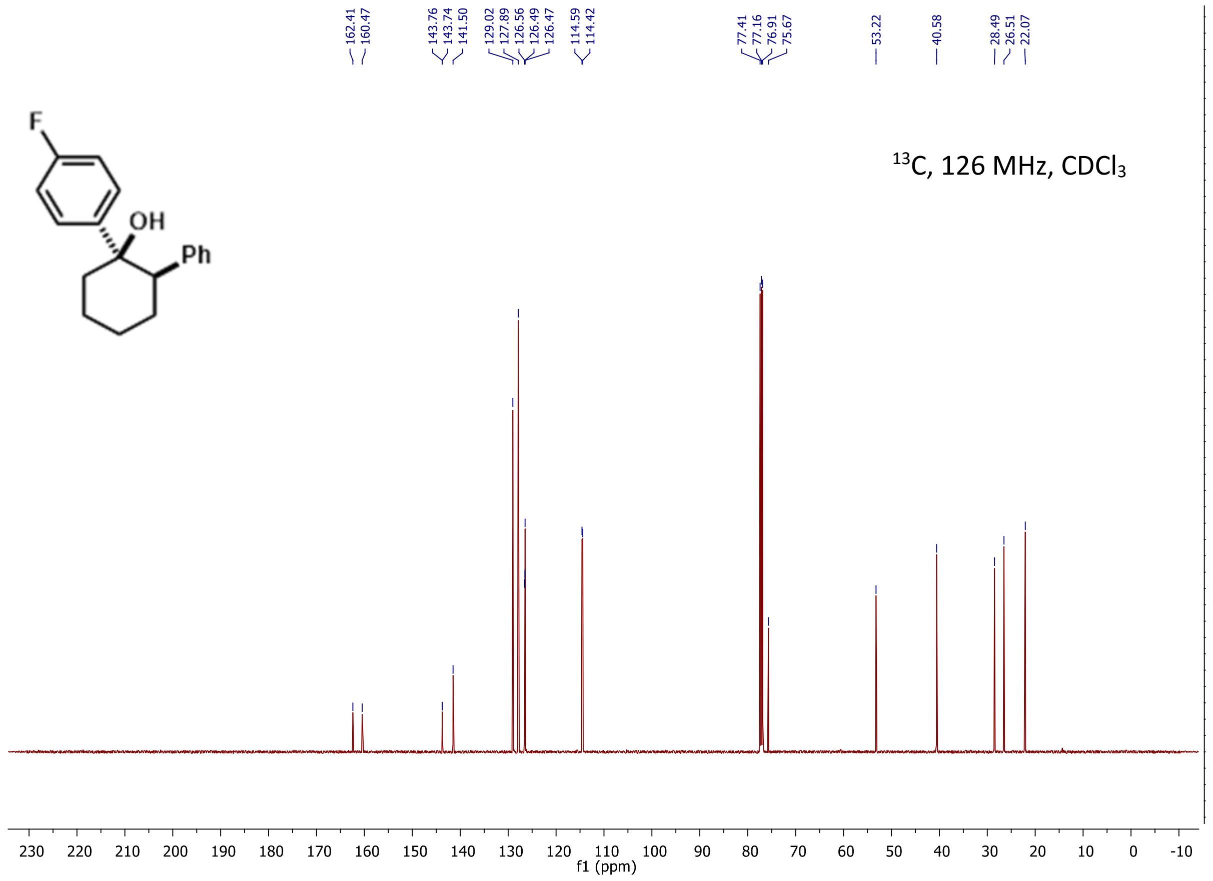

^{13}C, 126 MHz, CDCl$_3$
OH
Ph
F
f1 (ppm)

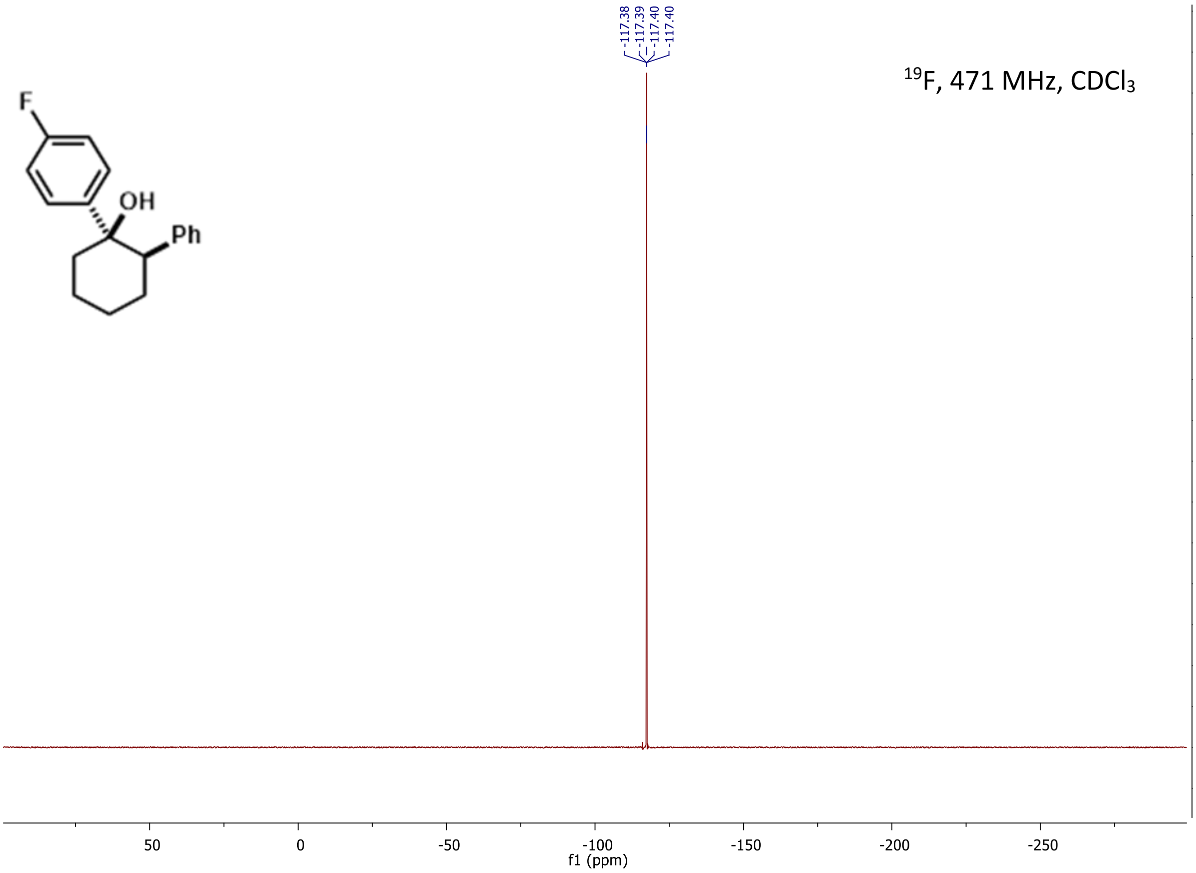
-117.38
-117.39
-117.40
-117.40
19F, 471 MHz, CDCl3
F
OH
Ph
50
0
-50
-100
-150
-200
-250
f1 (ppm)

(S8)

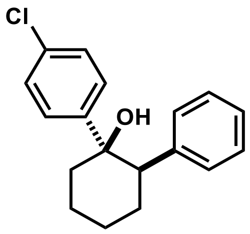

Prepared according to General Procedure A part B(2) using 4-chloroiodobenzene (2.15 g, 9 mmol), n-butyllithium (4.1 mL, 9 mmol, 2.2 M in hexanes), THF (30 mL) and 2-phenylcyclohexanone (1.05 g, 6 mmol). The crude residue was purified by flash column chromatography (eluent = 5 to 10% EtOAc in hexanes, silica gel) to afford product as a yellow solid (0.98 g, 57% yield).

Mp.: 86-88 °C; **R_f** = 0.43 (eluent = 10% EtOAc in hexanes); **ν_{max} / cm^{-1}** (thin film) 3456, 2932, 1601, 1489, 1445, 1269, 1090, 980; **^{1}H NMR (400 MHz, $CDCl_3$)** δ_H: 1.48- 1.61 (1H, m), 1.70-1.90 (5H, m), 1.90-2.09 (2H, m), 2.22 (1H, app qd, *J* 12.9, 3.4 Hz), 3.00 (1H, dd, *J* 12.8, 3.5 Hz), 6.87-6.94 (2H, m), 7.05-7.11 (3H, m), 7.13-7.19 (4H, m); **^{13}C NMR (101 MHz, $CDCl_3$)** δ_C: 22.0, 26.5, 28.5, 40.5, 53.0, 75.7, 126.4, 126.5, 127.9, 128.0, 129.0, 132.1, 141.4, 146.6; **HRMS (CI$^+$)** [$C_{18}H_{19}OCl$] requires [M]$^+$ 286.1124, found 286.1122 (- 0.7ppm).

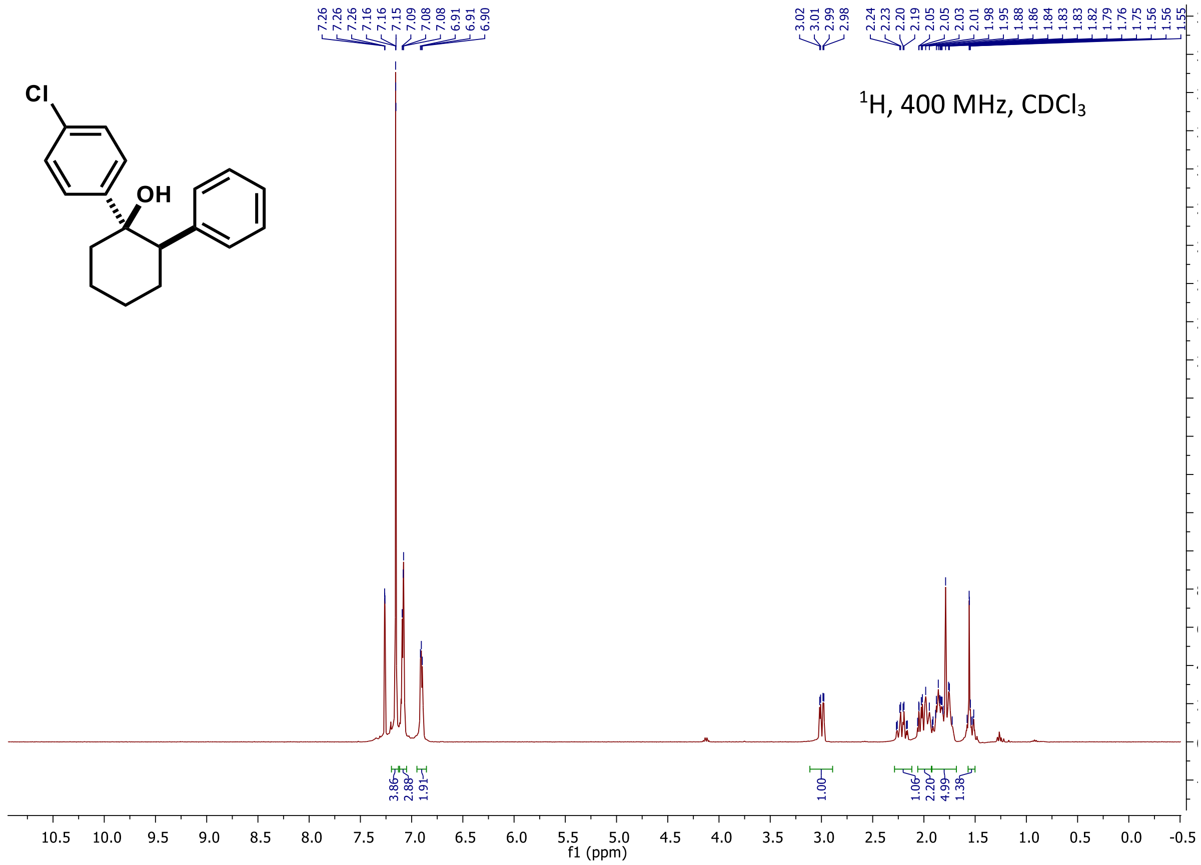
Cl
OH
1H, 400 MHz, CDCl3
7.26 7.26 7.26 7.16 7.15 7.09 7.08 7.08 6.91 6.91 6.90
3.02 3.01 2.99 2.98
2.24 2.23 2.20 2.19 2.05 2.05 2.03 2.01 1.98 1.95 1.88 1.86 1.84 1.83 1.83 1.82 1.79 1.76 1.75 1.56 1.56 1.55
3.86 2.88 1.91 1.00 1.06 2.20 4.99 1.38
f1 (ppm)

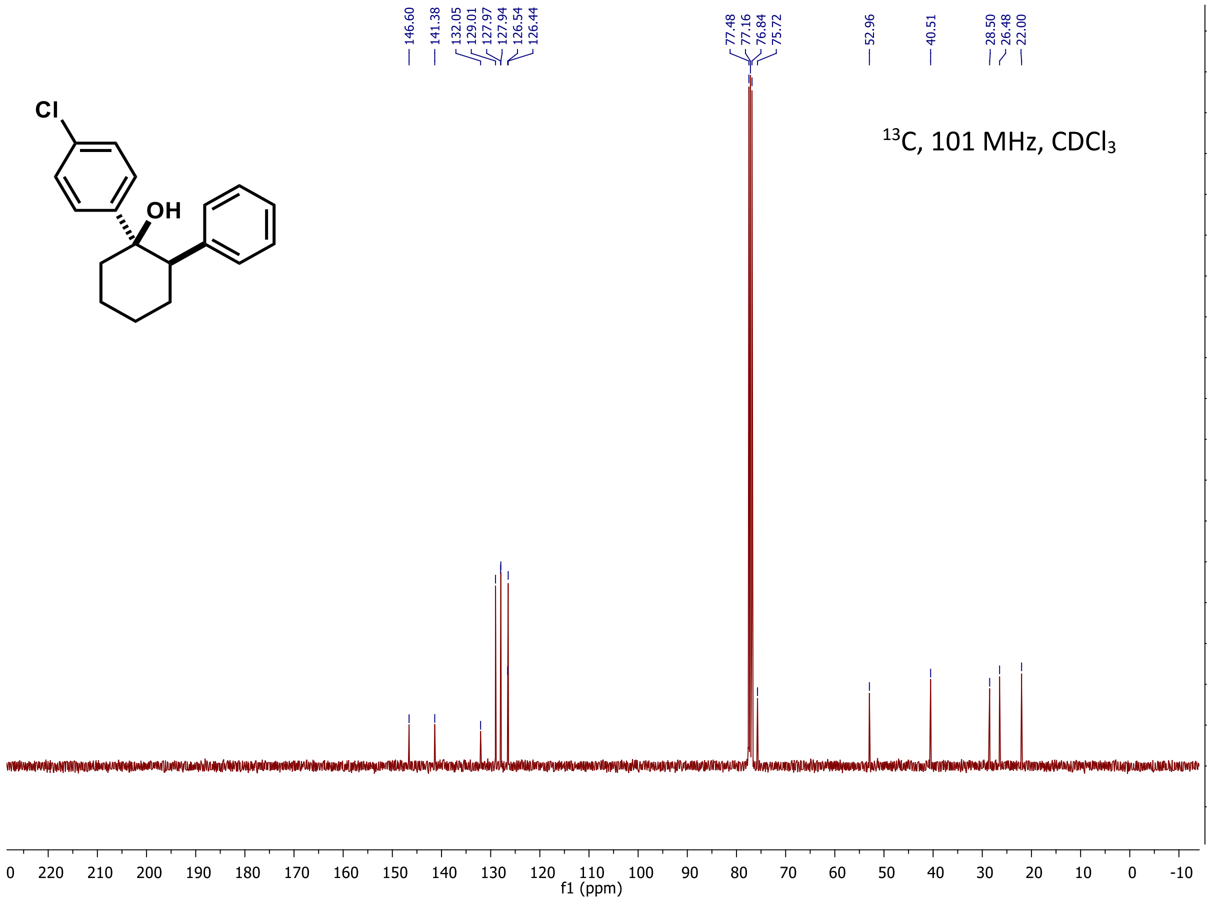
Cl
OH
13C, 101 MHz, CDCl3
146.60
141.38
132.05 129.01 127.97 127.94 126.54 126.44
77.48 77.16 76.84 75.72
52.96
40.51
28.50 26.48 22.00
f1 (ppm)

(S9)

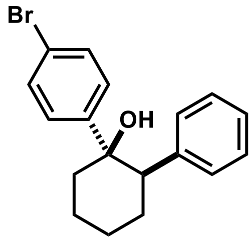

Prepared according to General Procedure A part B(2) using 4-bromoiodobenzene (2.12 g, 7.5 mmol), n-butyllithium (3.4 mL, 7.5 mmol, 2.2 M in hexanes), THF (25 mL) and 2-phenylcyclohexanone (0.87 g, 5 mmol). The crude residue was purified by flash column chromatography (eluent = 5 to 10% EtOAc in hexanes, silica gel) to afford product as a yellow oil (0.74 g, 45% yield).

Mp.: 107-109 °C; **R_f** = 0.35 (eluent = 10% EtOAc in hexanes); **ν_{max} / cm^{-1}** (thin film) 3347, 2950, 1487, 1443, 1269, 1059; **^{1}H NMR (400 MHz, $CDCl_3$)** δ_H: 1.46-1.60 (1H, m), 1.68-1.80 (3H, m), 1.81-1.90 (2H, m), 1.89-2.10 (2H, m), 2.21 (1H, app qd, *J* 13.1, 3.6 Hz), 2.99 (1H, dd, *J* 12.8, 3.6 Hz), 6.86-6.93 (2H, m), 7.02-7.13 (5H, m), 7.30 (2H, d, *J* 8.8 Hz); **^{13}C NMR (101 MHz, $CDCl_3$)** δ_C: 22.0, 26.5, 28.5, 40.5, 52.9, 75.8, 120.2, 126.6, 126.8, 128.0, 129.0, 130.9, 141.4, 147.2; **HRMS (CI^+)** [$C_{18}H_{19}OBr$] requires $[M]^+$ 330.0619, found 330.0615 (-1.2 ppm).

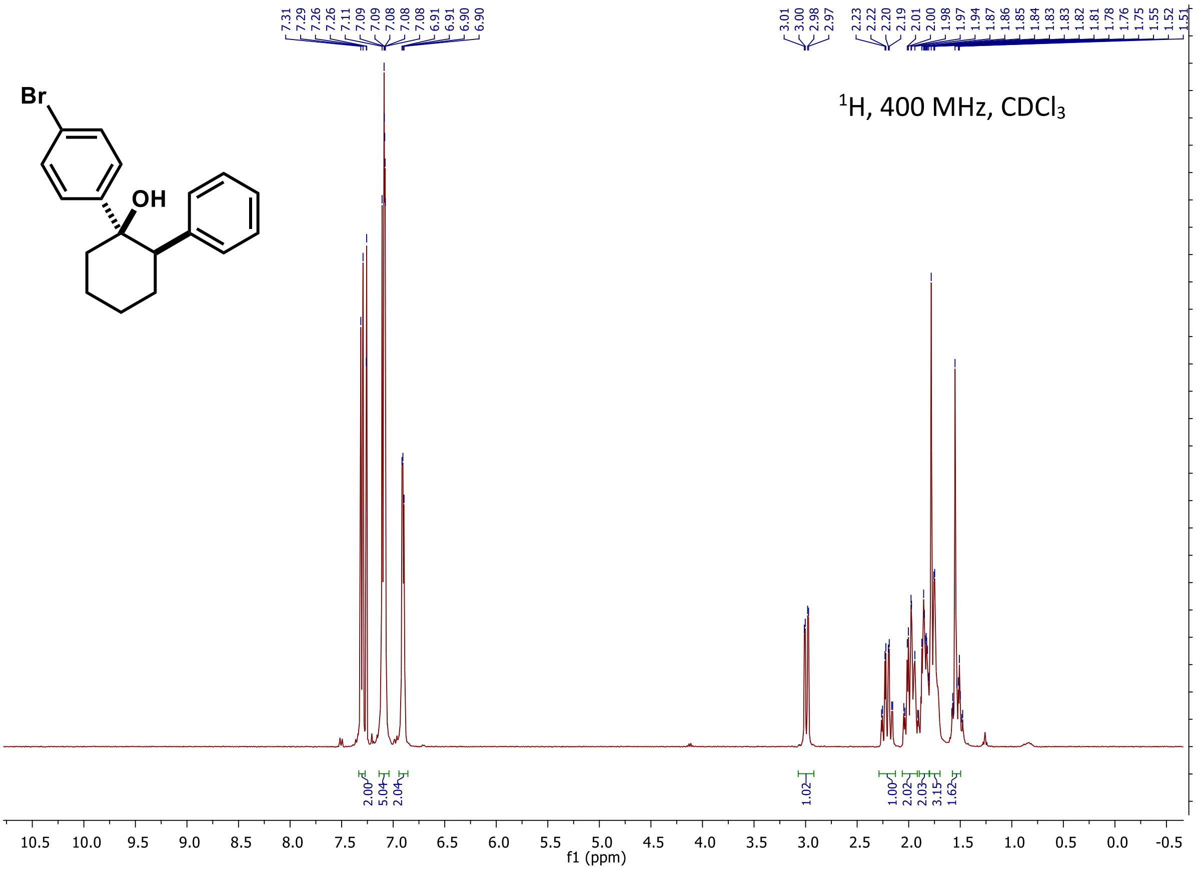

Br
OH
1H, 400 MHz, CDCl3
7.31 7.29 7.26 7.26 7.11 7.09 7.09 7.08 7.08 6.91 6.91 6.90 6.90
3.01 3.00 2.98 2.97
2.23 2.22 2.20 2.19 2.01 2.00 1.98 1.97 1.94 1.87 1.86 1.85 1.84 1.83 1.83 1.82 1.81 1.78 1.76 1.75 1.55 1.52 1.51
2.00 5.04 2.04 1.02 1.00 2.02 2.03 3.15 1.62
f1 (ppm)

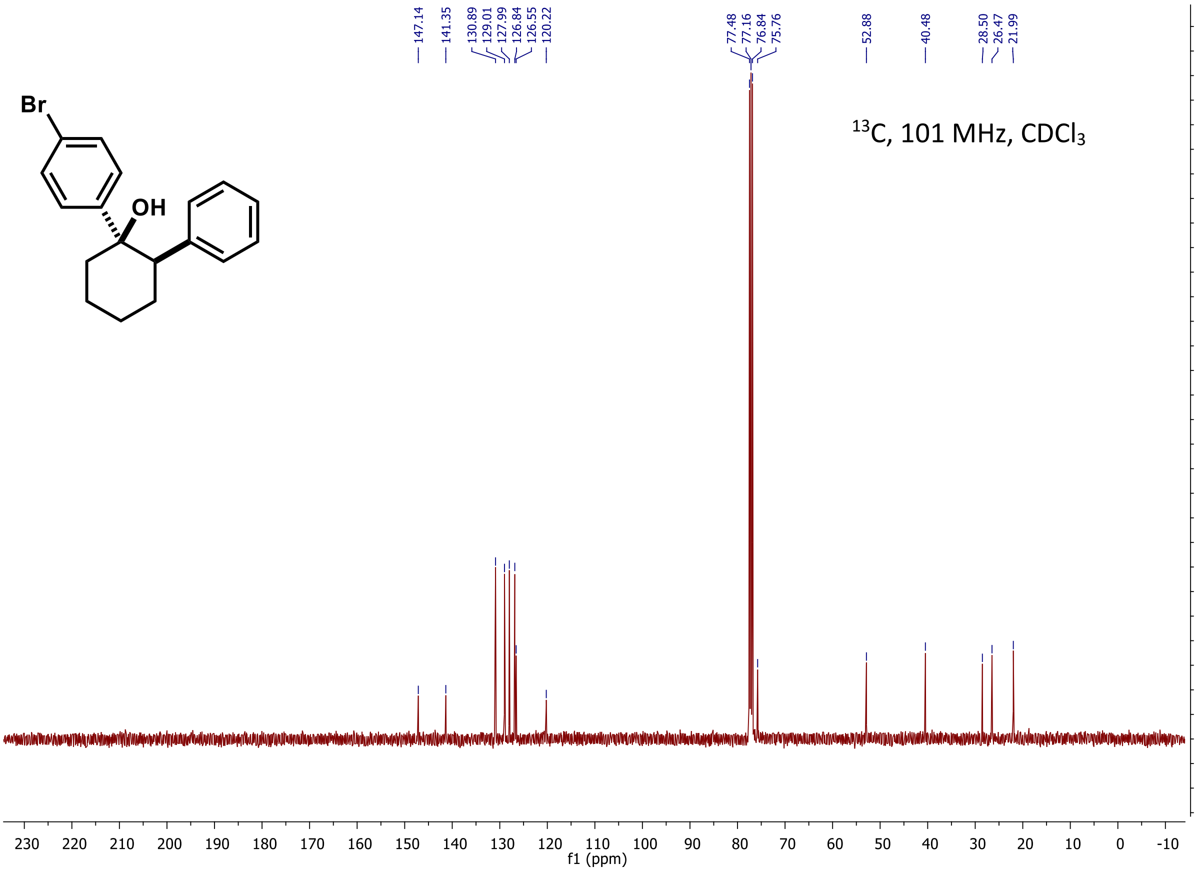

Br
OH
13C, 101 MHz, CDCl3
147.14 141.35 130.89 129.01 127.99 126.84 126.55 120.22
77.48 77.16 76.84 75.76
52.88 40.48 28.50 26.47 21.99
f1 (ppm)

(S10)

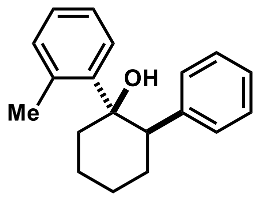

Prepared according to General Procedure A, part B(1) using magnesium turnings (219 mg, 9 mmol), iodine (1 crystal), 1-bromo-2-methylbenzene (0.92 mL, 7.5 mmol), THF (15 mL) and 2-phenylcyclohexan-1-one (871 mg, 5 mmol). The crude residue was purified by flash column chromatography (5 to 10% EtOAc in hexanes, silica gel) to afford product as a brown oil (0.65 g, 50% yield).

R_f = 0.80 (10% EtOAc/Petrol); **ν_{max} / cm^{-1}** (thin film); 3560, 2927, 2856, 1600, 1489, 1461, 750; **^{1}H NMR (500 MHz, $DMSO_{d6}$)** δ_H: 1.42-1.64 (3H, m), 1.65-1.75 (1H, m), 1.76-1.84 (1H, m), 1.84-1.95 (1H, m), 2.13-2.36 (2H, m), 4.62 (1H, s (br)), 6.75-7.11 (8H, m), 7.38 (1H, d, *J* 9.1 Hz); **^{13}C NMR (126 MHz, $CDCl_3$)** δ_C: 14.2, 21.0, 21.1, 22.0, 23.0, 26.3, 28.1, 60.4, 125.6, 126.3, 126.4, 127.7, 128.8, 132.5, 141.7, 145.1, 171.2; **HRMS (APCI$^-$)** [$C_{19}H_{22}O$] requires [M-H] 265.1592, found 265.1596 (+ 1.5 ppm).

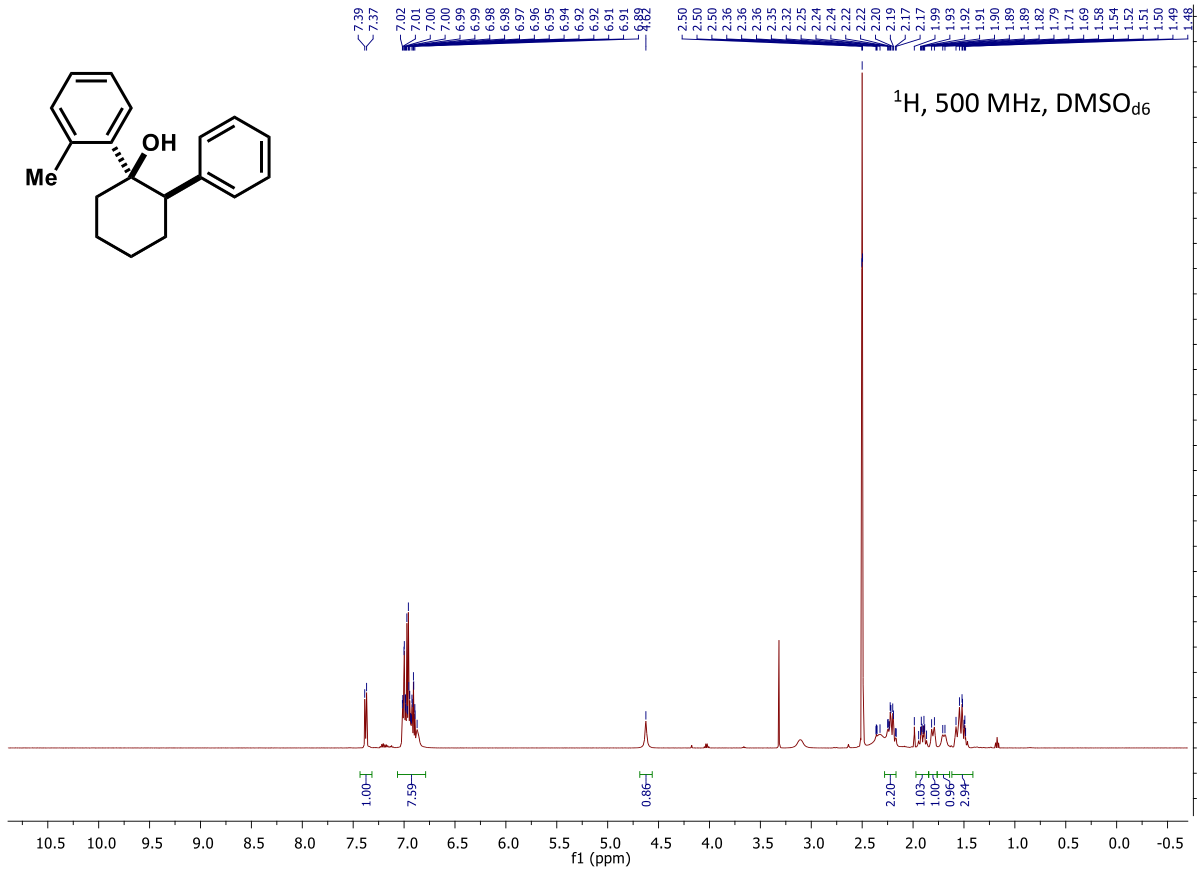
1H, 500 MHz, DMSOd6
OH
Me
f1 (ppm)

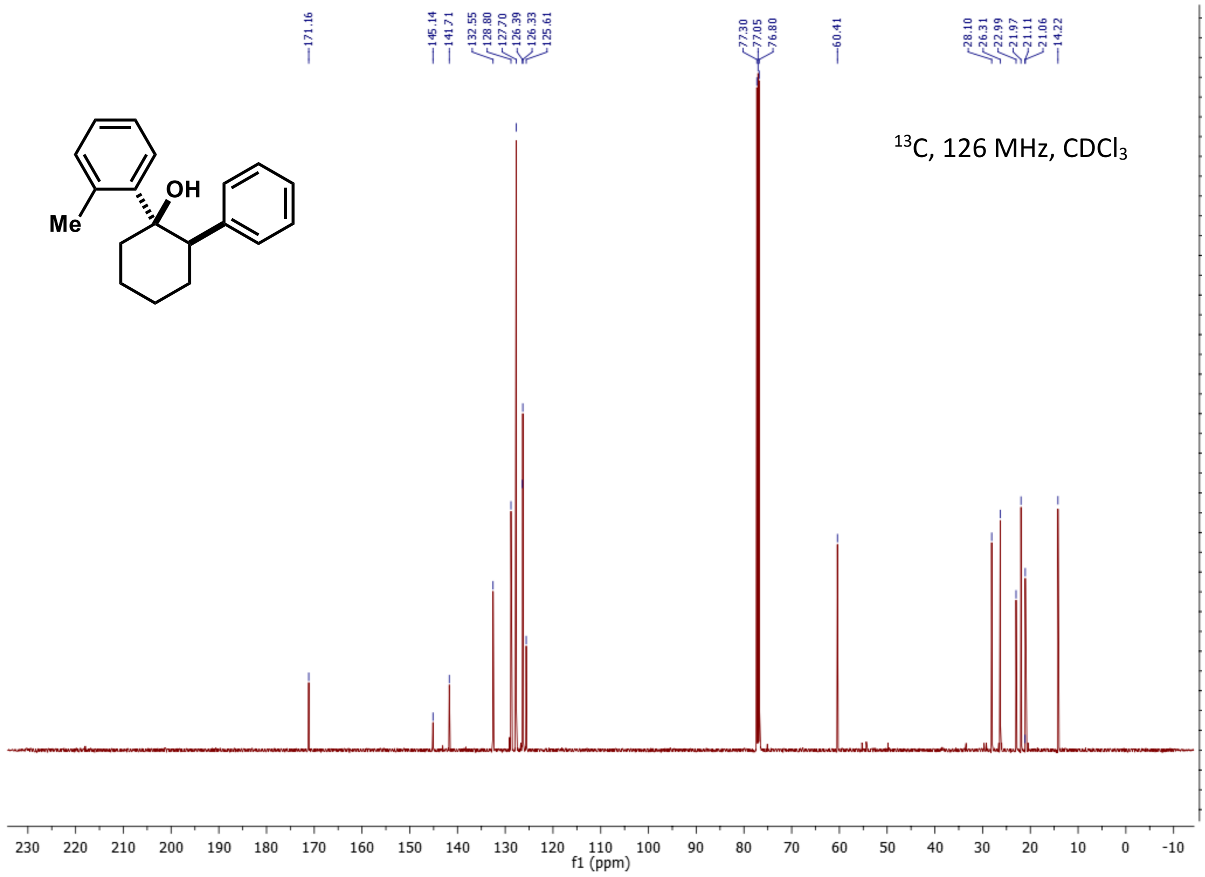
13C, 126 MHz, CDCl3
OH
Me
171.16
145.14
141.71
132.55
128.80
127.70
126.39
126.33
125.61
77.30
77.05
76.80
60.41
28.10
26.31
22.99
21.97
21.11
21.06
14.22
f1 (ppm)

(S11)

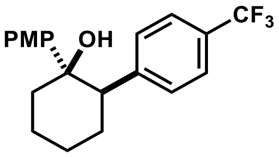

Prepared according to General Procedure A, part A using magnesium (1.44 g, 60 mmol), iodine (1 crystal), THF (100 mL), 4-Bromobenzotrifluoride (8.43 g, 37.5 mmol) cyclohexene oxide (2.45 g, 25 mmol) and CuCl (248 mg, 2.5 mmol). A portion of the crude residue (3.30 g, 13 mmol) was used directly in the next step with DMP (6.30 g, 15 mmol) and CH_2Cl_2 (30 mL). The crude residue was purified by flash column chromatography (eluent = 10 % EtOAc in hexanes, silica gel) to afford intermediate ketone as a white solid (1.05 g, 32% yield).

Prepared according to General Procedure A, part B(1) using magnesium (178 mg, 7.44 mmol), iodine (1 crystal), THF (12.5 mL), 4-bromoanisole (1.16 g, 6.20 mmol) 2-(4-(trifluoromethyl)phenyl)cyclohexan-1-one (1.00 g, 4.18 mmol). The crude residue was purified by flash column chromatography (eluent = 5 to 10% EtOAc in hexanes, silica gel) to afford product as a white solid (1.10 g, 75% yield).

Mp.: 85-87 °C; $\mathbf{R_f}$ = 0.41 (eluent = 20% EtOAc in hexanes); $\mathbf{\nu_{max}}$ **/ cm^{-1}** (thin film) 3350, 2916, 2358, 1618, 1510, 1419, 1170, 1112; **^{1}H NMR (500 MHz, CDCl$_3$)** δ_H: 1.53-1.61 (2H, m), 1.70-1.90 (4H, m), 1.93-2.00 (1H, m), 2.07-2.15 (1H, m), 2.22 (1H, app qd, *J* 13.1, 3.6 Hz), 2.97 (1H, dd, *J* 12.8, 3.5 Hz), 6.72 (2H, d, *J* 8.8 Hz), 7.05 (2H, d, *J* 8.2 Hz), 7.10 (2H, d, *J* 8.8 Hz), 7.31 (2H, d, *J* 8.2 Hz); **^{13}C NMR (126 MHz, CDCl$_3$)** δ_C: 22.0, 26.4, 28.8, 40.9, 53.5, 55.3, 75.5, 113.3, 124.5 (q, *J* 3.8 Hz), 124.5 (q, *J* 273 Hz), 125.9, 128.3 (q, *J* 32.8 Hz), 129.5, 139.7, 146.4, 158.2; **^{19}F NMR (471 MHz, CDCl$_3$)** δ_F: -62.4; **HRMS (CI$^+$)** [$C_{20}H_{21}O_2F_3$] requires [M]$^+$ 350.1494, found 350.1489 (-1.4 ppm).

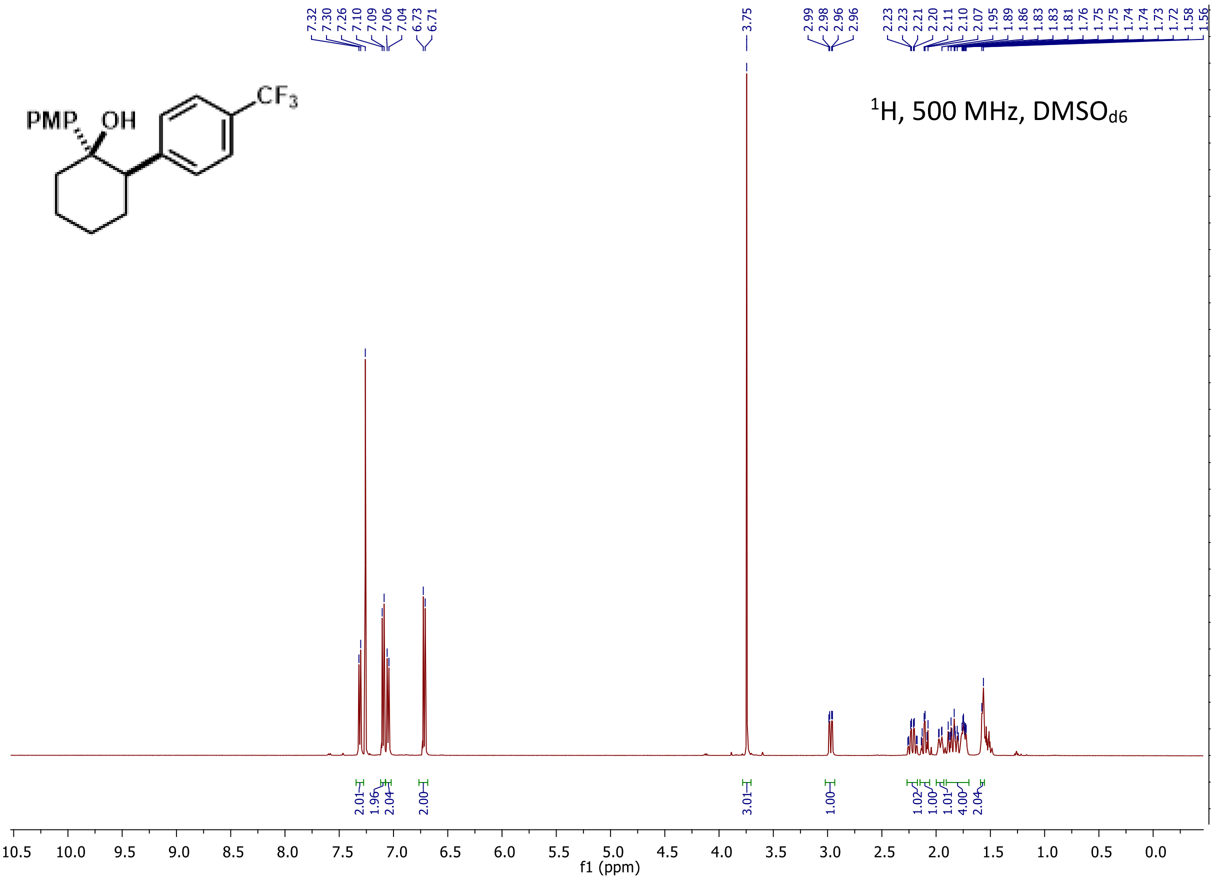
PMP OH
CF3
1H, 500 MHz, DMSOd6
7.32 7.30 7.26 7.10 7.09 7.06 7.04 6.73 6.71
3.75
2.99 2.98 2.96 2.96
2.23 2.23 2.21 2.20 2.11 2.10 2.07 1.95 1.89 1.86 1.83 1.83 1.81 1.76 1.75 1.75 1.74 1.73 1.72 1.58 1.56
2.01 1.96 2.04 2.00 3.01 1.00 1.02 1.00 1.01 4.00 2.04
f1 (ppm)

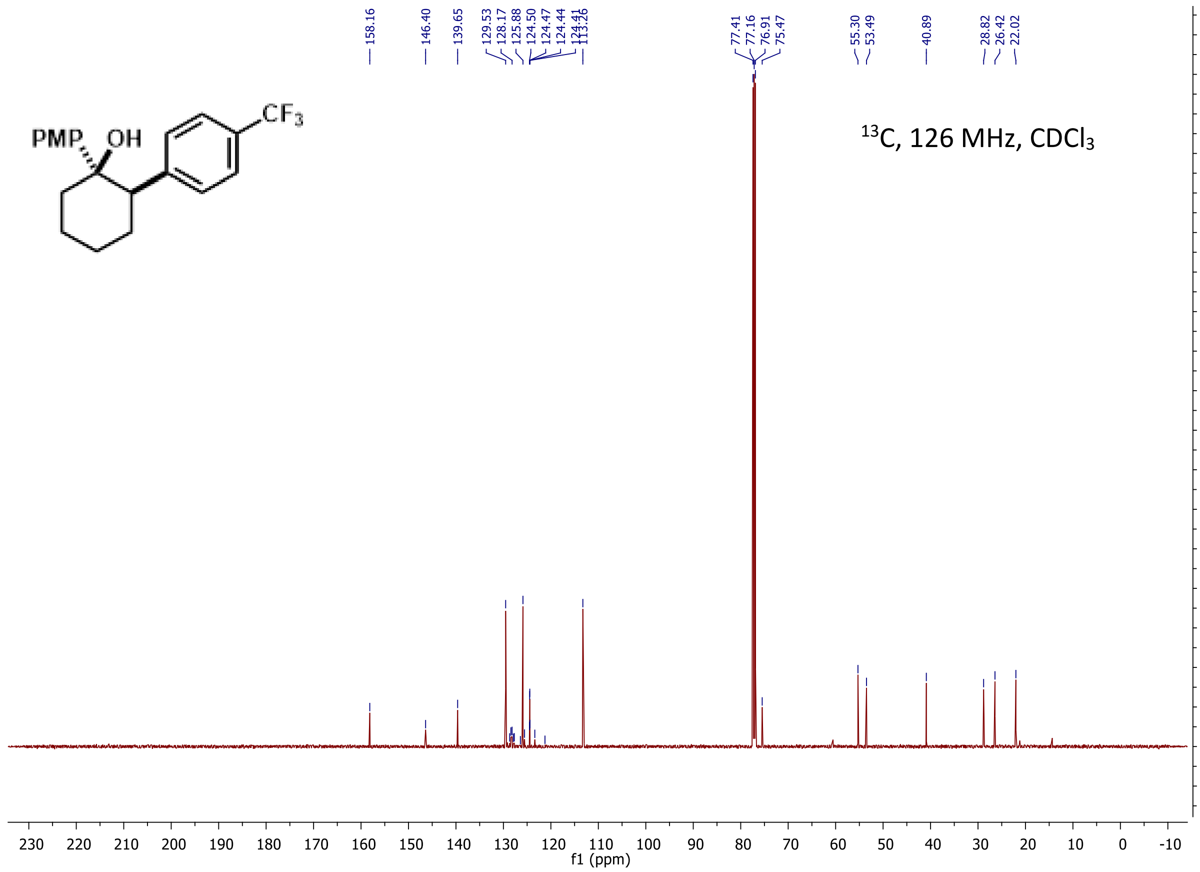
PMP OH
CF3
13C, 126 MHz, CDCl3
158.16 146.40 139.65 129.53 128.17 125.88 124.50 124.47 124.44 124.41 113.26
77.41 77.16 76.91 75.47
55.30 53.49 40.89 28.82 26.42 22.02
f1 (ppm)

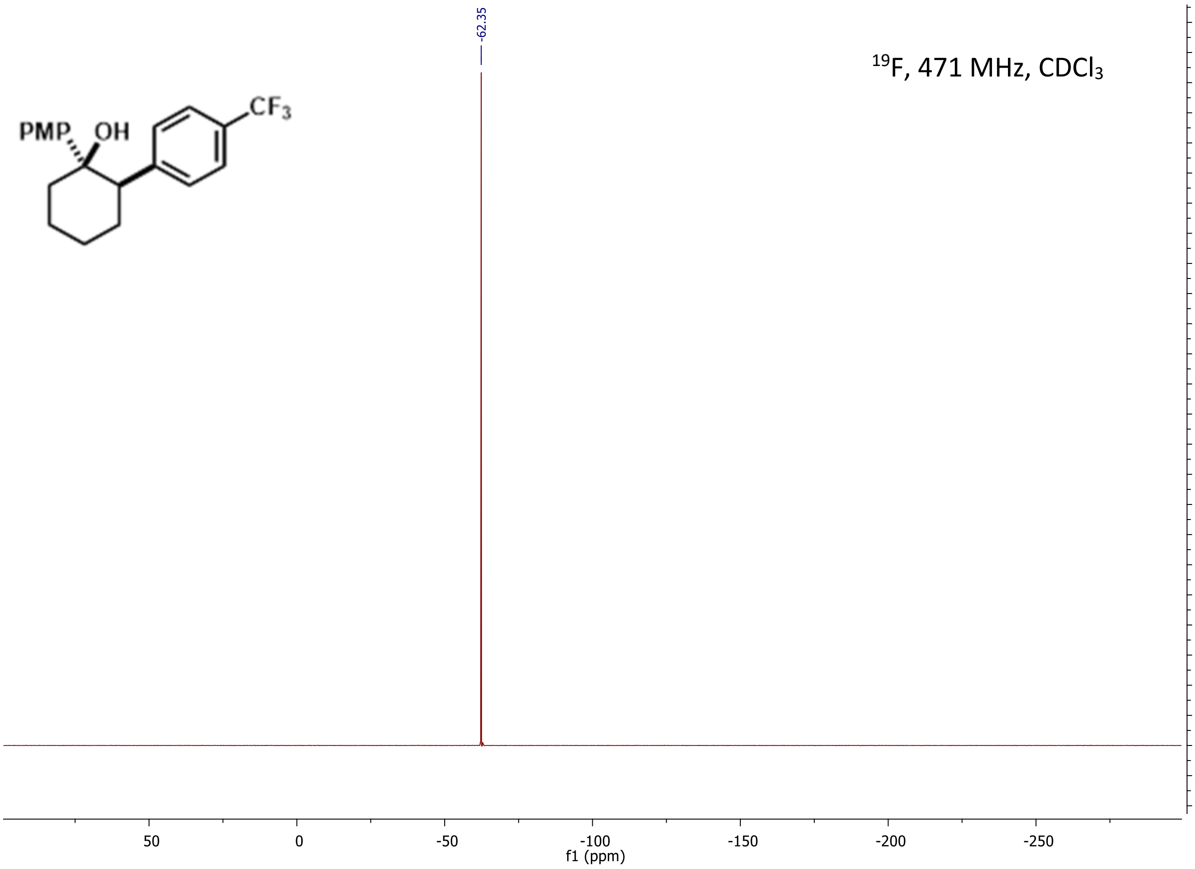

-62.35
^{19}F, 471 MHz, $CDCl_3$
PMP
OH
CF_3
50
0
-50
-100
-150
-200
-250
f1 (ppm)

(S12)

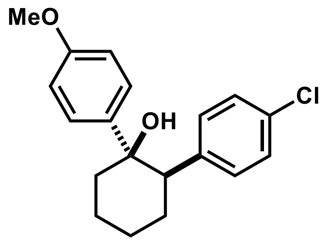

Prepared according to General Procedure A, part B(2) using 4-bromoanisole (0.94 mL, 7.5 mmol), *n*-butyllithium (3.0 mL, 7.5 mmol), THF (15 mL) and 2-(4-chlorophenyl)cyclohexan-1-one (1.04 g, 5 mmol). The crude residue was purified by flash column chromatography (5 to 10% EtOAc in hexanes, silica gel) to afford product as a yellow solid (1.22 g, 77% yield).

M.p.: 45-47 °C; $\mathbf{R_f}$ = 0.44 (10% EtOAc in hexanes); $\mathbf{\nu_{max}}$ **/ cm^{-1}** (thin film) 3460, 2926, 2845, 1610, 1508, 1244, 1168, 1087; **^{1}H NMR (300 MHz, CDCl$_3$)** δ_H: 1.53-1.58 (1H, m), 1.69-1.98 (5H, m), 2.06-2.24 (2H, m), 2.92 (1H, dd, *J* 12.8, 3.6 Hz), 3.75 (3H, s), 6.73 (2H, d, *J* 9.0 Hz), 6.86 (2H, d, *J* 8.5 Hz), 7.03 (2H, d, *J* 8.5 Hz), 7.10 (2H, d, *J* 9.0 Hz); **^{13}C NMR (126 MHz, CDCl$_3$)** δ_C: 22.0, 26.5, 28.8, 40.7, 52.8, 55.3, 75.5, 113.2, 125.9, 127.7, 130.5, 131.9, 139.9, 140.6, 158.1; **HRMS (EI$^+$)** [$C_{19}H_{21}O_2Cl$] requires [M-H_2O+H]$^+$ 299.1203, found 299.1200 (- 1.0 ppm).

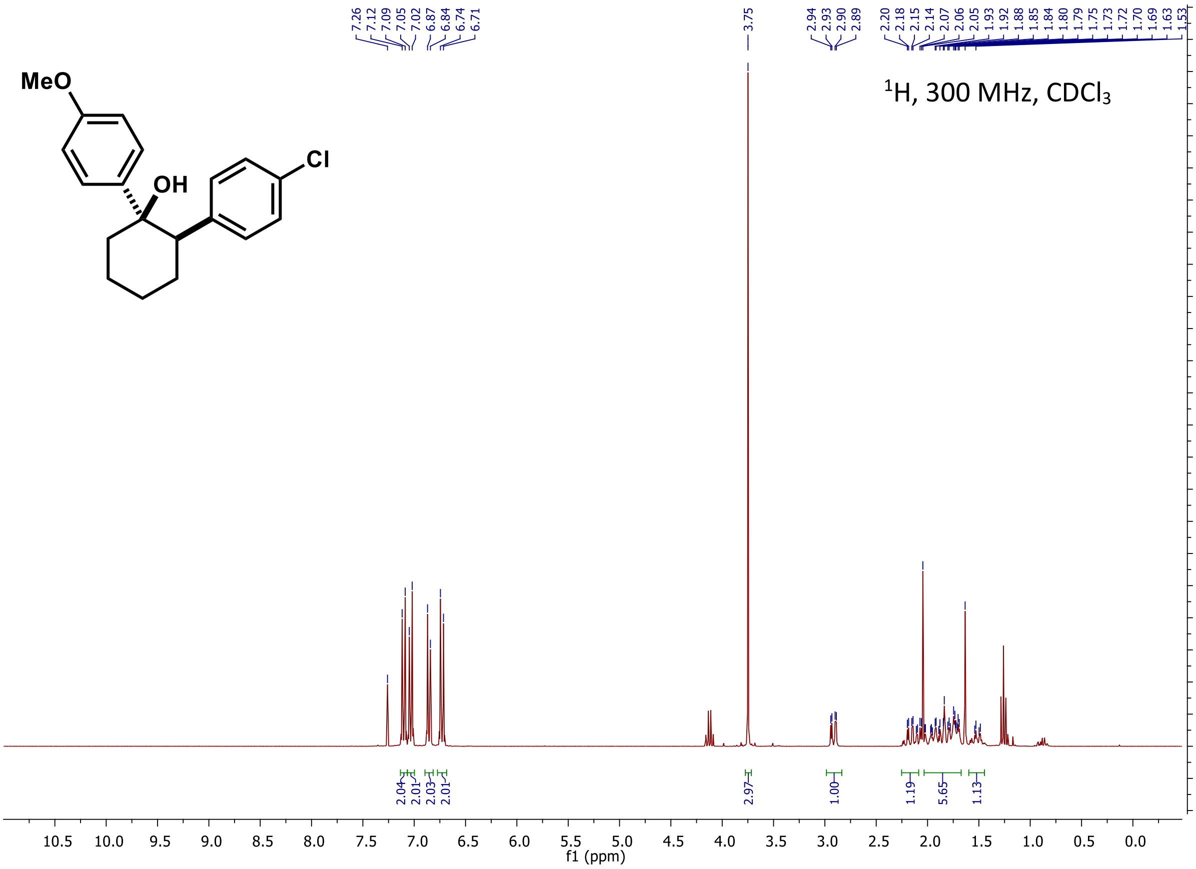

MeO
OH
Cl
¹H, 300 MHz, CDCl₃
f1 (ppm)

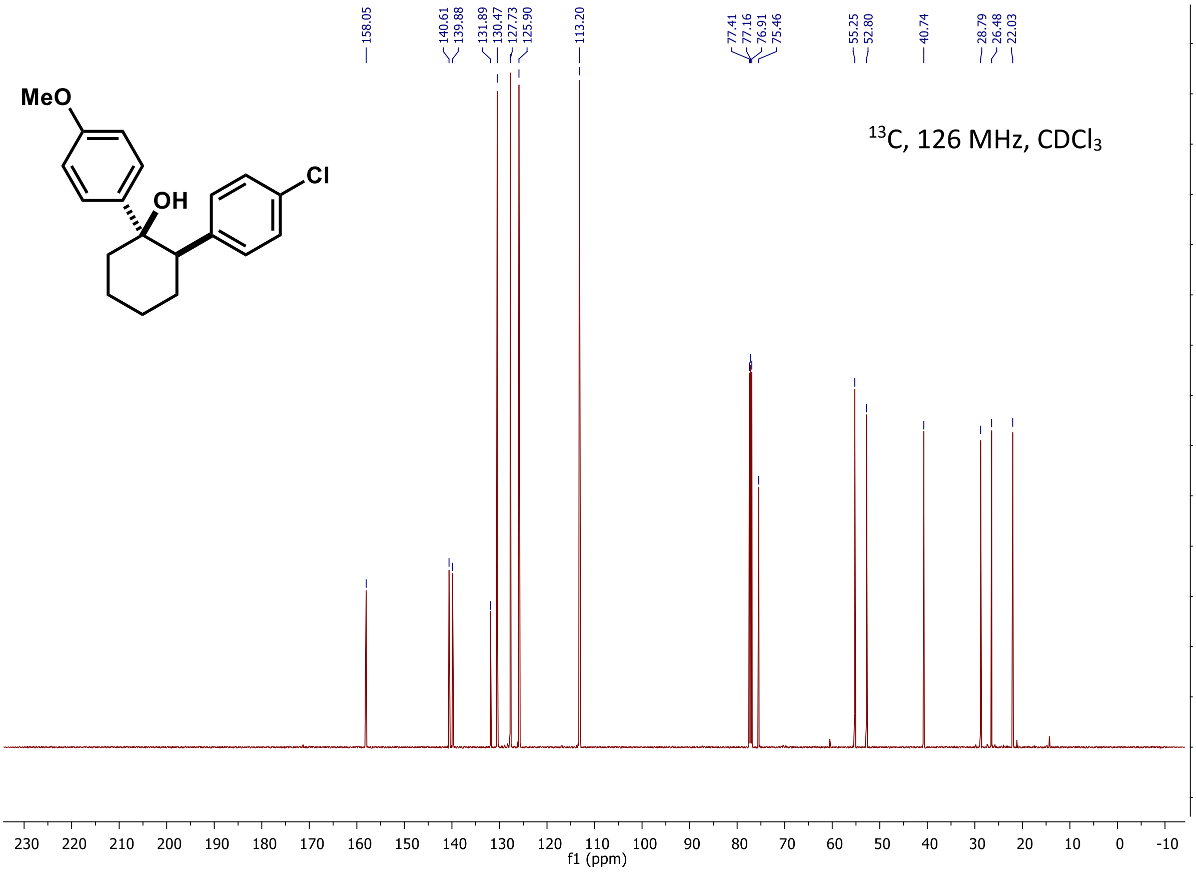

MeO
OH
Cl
¹³C, 126 MHz, CDCl₃
f1 (ppm)

(S13)

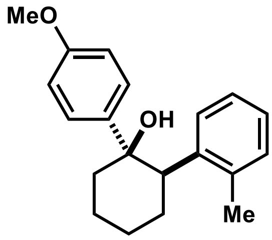

Prepared according to General Procedure A, part B(2) using 4-bromoanisole (0.94 mL, 7.5 mmol,), *n*-butyllithium (3.0 mL, 7.5 mmol), and 2-(*o*-tolyl)cyclohexan-1-one (0.94 g, 5 mmol). The crude residue was purified by flash column chromatography (5 to 10% EtOAc in hexanes, silica gel) to afford product as a yellow oil (0.93 g, 63% yield).

R_f = 0.44 (10% EtOAc in hexanes); **ν_{max} / cm^{-1}** (thin film) 3558, 2999, 2929, 1610, 1508, 1444, 1246, 1176, 1035, 977, 827, 758, 729, 574 ; **^{1}H NMR (500 MHz, $CDCl_3$)** δ_H: 1.47-1.57 (1H, m), 1.66-1.71 (1H, m), 1.71 (3H, s), 1.78-1.84 (2H, m), 1.85-1.89 (1H, m), 1.92-1.97 (2H, m), 2.11-2.20 (1H, m), 2.25-2.33 (1H, m), 3.08 (1H, dd, *J* 18.6, 8.2), 3.72 (3H, s), 6.63-6.65 (2H, m), 6.84 (1H, d, *J* 7.5 Hz), 6.98-7.03 (3H, m), 7.13 (1H, t, *J* 7.5 Hz), 7.61-7.63 (1H, m); **^{13}C NMR (126 MHz, $CDCl_3$)** δ_C: 19.9, 22.0, 26.8, 30.0, 40.2, 55.4, 75.2, 112.8, 125.4, 125.9, 126.4, 128.3, 129.9, 136.6, 141.2, 158.1; **HRMS (EI$^+$)** [$C_{20}H_{24}O_2$] requires [M]$^+$ 296.1776, found 296.1771 (-1.7 ppm).

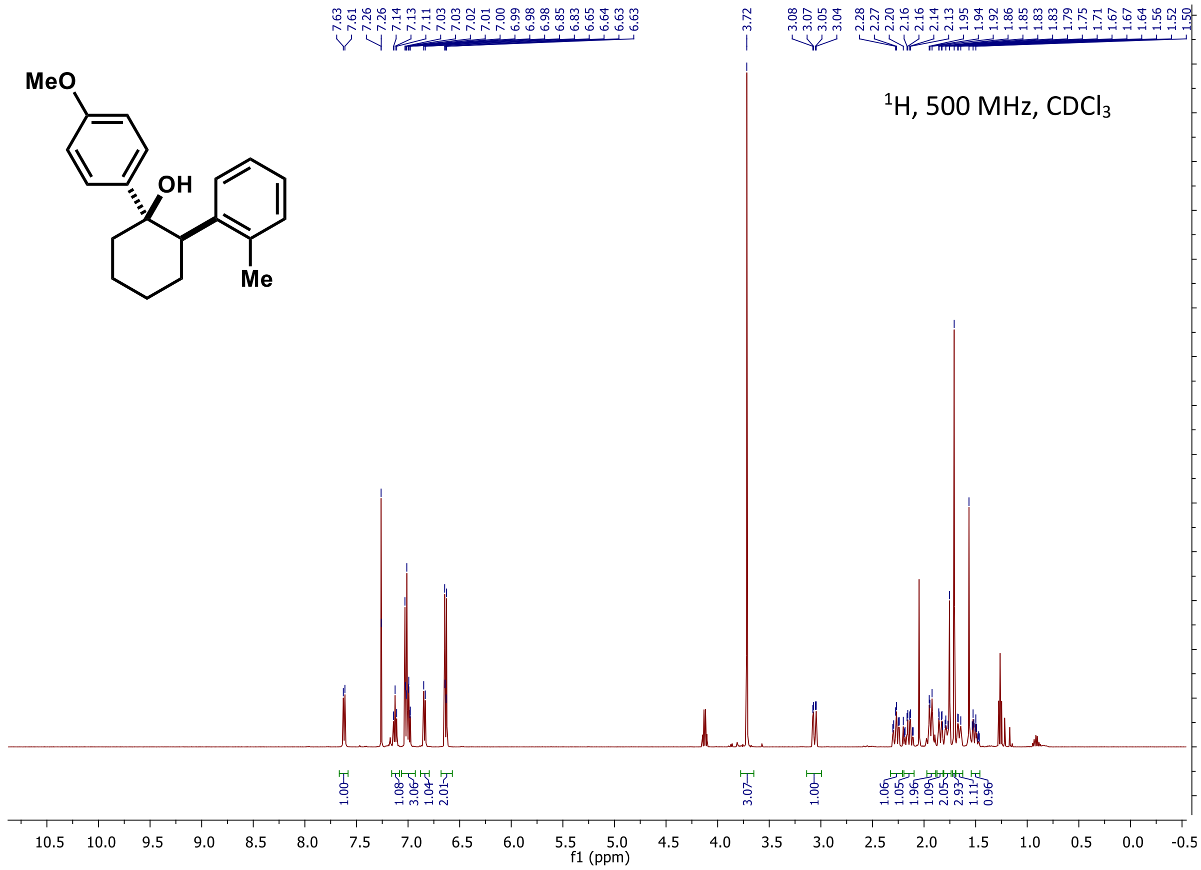
MeO
OH
Me
1H, 500 MHz, CDCl3
7.63 7.61 7.26 7.14 7.13 7.11 7.03 7.02 7.01 7.00 6.99 6.98 6.98 6.85 6.83 6.65 6.64 6.63 6.63
3.72
3.08 3.07 3.05 3.04
2.28 2.27 2.20 2.16 2.14 2.13 1.95 1.94 1.92 1.86 1.85 1.83 1.83 1.79 1.75 1.71 1.67 1.64 1.56 1.52 1.50
1.00 1.08 3.06 1.04 2.01 3.07 1.00 1.06 1.05 1.96 1.09 2.05 2.93 1.11 0.96
f1 (ppm)

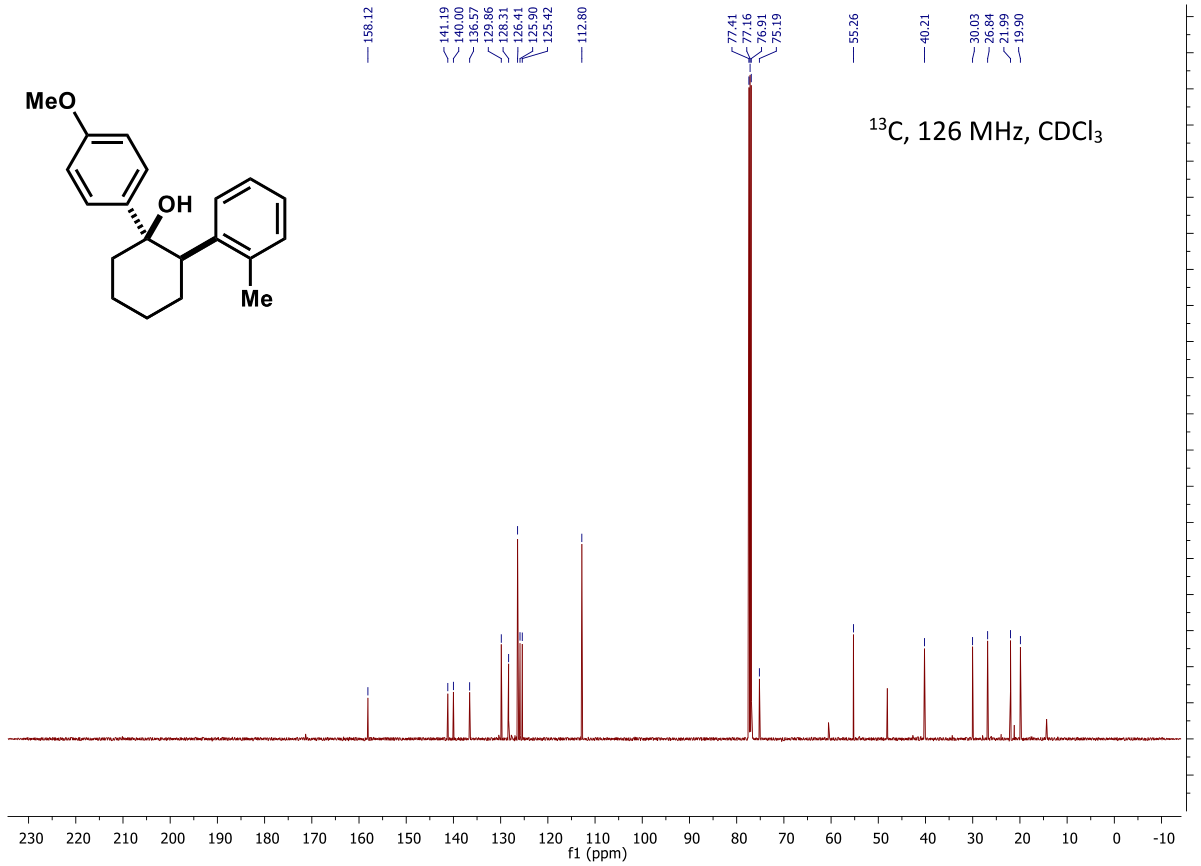
MeO
OH
Me
13C, 126 MHz, CDCl3
158.12
141.19 140.00 136.57 129.86 128.31 126.41 125.90 125.42
112.80
77.41 77.16 76.91 75.19
55.26
40.21
30.03 26.84 21.99 19.90
f1 (ppm)

(S14)

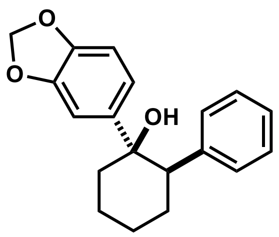

Prepared according to General Procedure A, part B(1) using magnesium turnings (219 mg, 9 mmol), iodine (1 crystal), 5-bromobenzo[*d*][1,3]dioxole (0.90 mL, 7.5 mmol), and 2- phenylcyclohexan-1-one (871 mg, 5 mmol). The crude residue was purified by flash column chromatography (5 to 10% EtOAc in hexanes, silica gel) to afford product as a yellow oil (1.00 g, 68% yield).

R_f = 0.33 (10% EtOAc/Petrol); **ν_{max} / cm^{-1}** (thin film) 3554, 2931, 1732, 1504, 1585, 1429, 1238, 1037, 698 ; **^{1}H NMR (500 MHz, $CDCl_3$)** δ_H: 1.47-1.56 (1H, m), 1.70 - 1.77 (3H, m), 1.81-1.87 (2H, m), 1.93-2.00 (2H, m), 2.21 (1H, qd, *J* 13.1, 3.6 Hz), 2.99 (1H, dd, *J* 12.8, 3.6 Hz), 5.88 (2H, dd, *J* 12.6, 1.5 Hz), 6.62-6.63 (1H, m), 6.67-6.69 (1H, m), 6.75 (1H, d, *J* 1.7 Hz), 6.93-6.97 (2H, m), 7.07-7.11 (3H, m); **^{13}C NMR (101 MHz, $CDCl_3$)** δ_C: 22.2, 26.5, 28.6, 40.9, 53.0, 75.8, 100.9, 106.0, 107.5, 118.0, 126.4, 127.9, 129.0, 141.7, 142.5, 145.8, 147.4; **HRMS (EI$^+$)** [$C_{19}H_{20}O_3$] requires [M-H_2O+H]$^+$ 279.1385, found 279.1386 (+ 0.4 ppm).

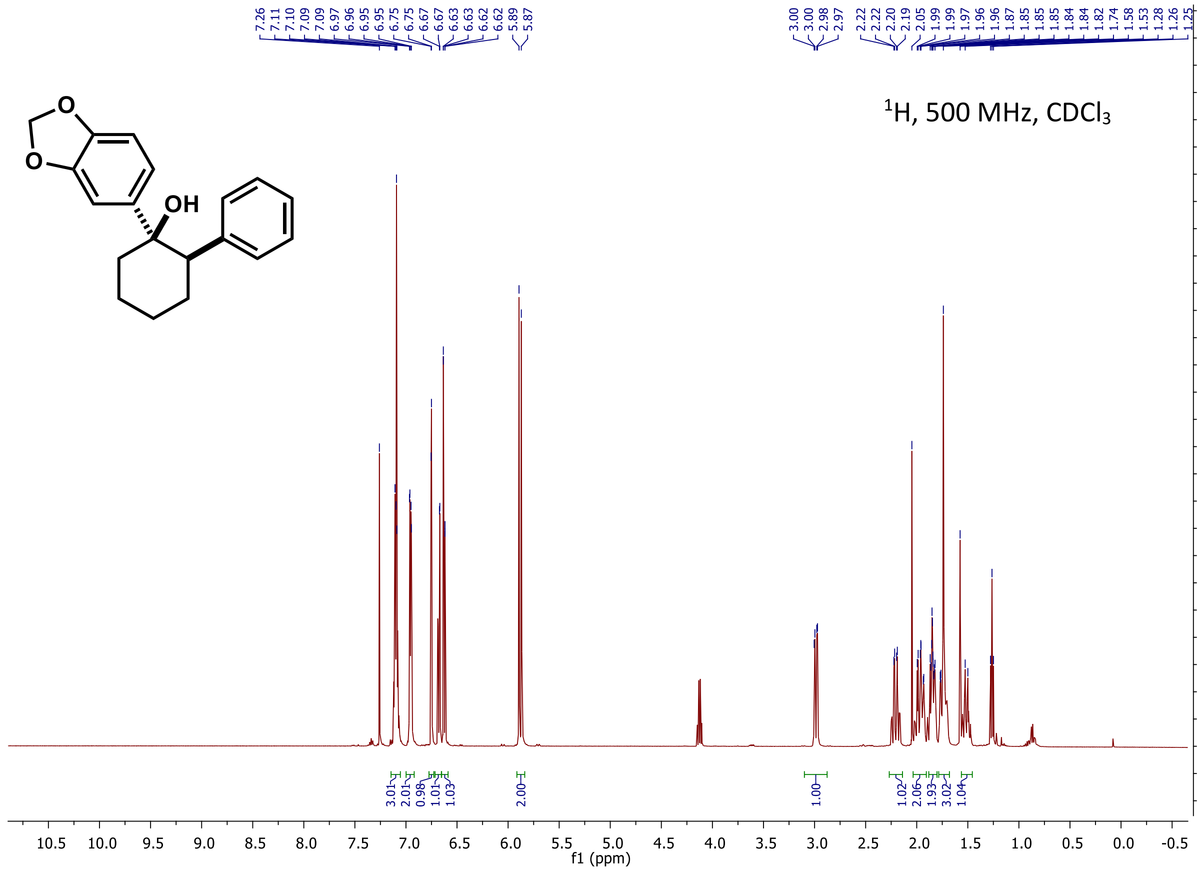
7.26 7.11 7.10 7.09 7.09 6.97 6.96 6.95 6.95 6.75 6.75 6.67 6.67 6.63 6.63 6.62 5.89 5.87
3.00 3.00 2.98 2.97 2.22 2.22 2.20 2.19 2.05 1.99 1.99 1.97 1.96 1.87 1.85 1.85 1.84 1.84 1.82 1.74 1.58 1.53 1.28 1.26 1.25
O
O
OH
^{1}H, 500 MHz, CDCl$_3$
3.01 2.01 0.98 1.01 1.03 2.00 1.00 1.02 2.06 1.93 3.02 1.04
10.5 10.0 9.5 9.0 8.5 8.0 7.5 7.0 6.5 6.0 5.5 5.0 4.5 4.0 3.5 3.0 2.5 2.0 1.5 1.0 0.5 0.0 -0.5
f1 (ppm)

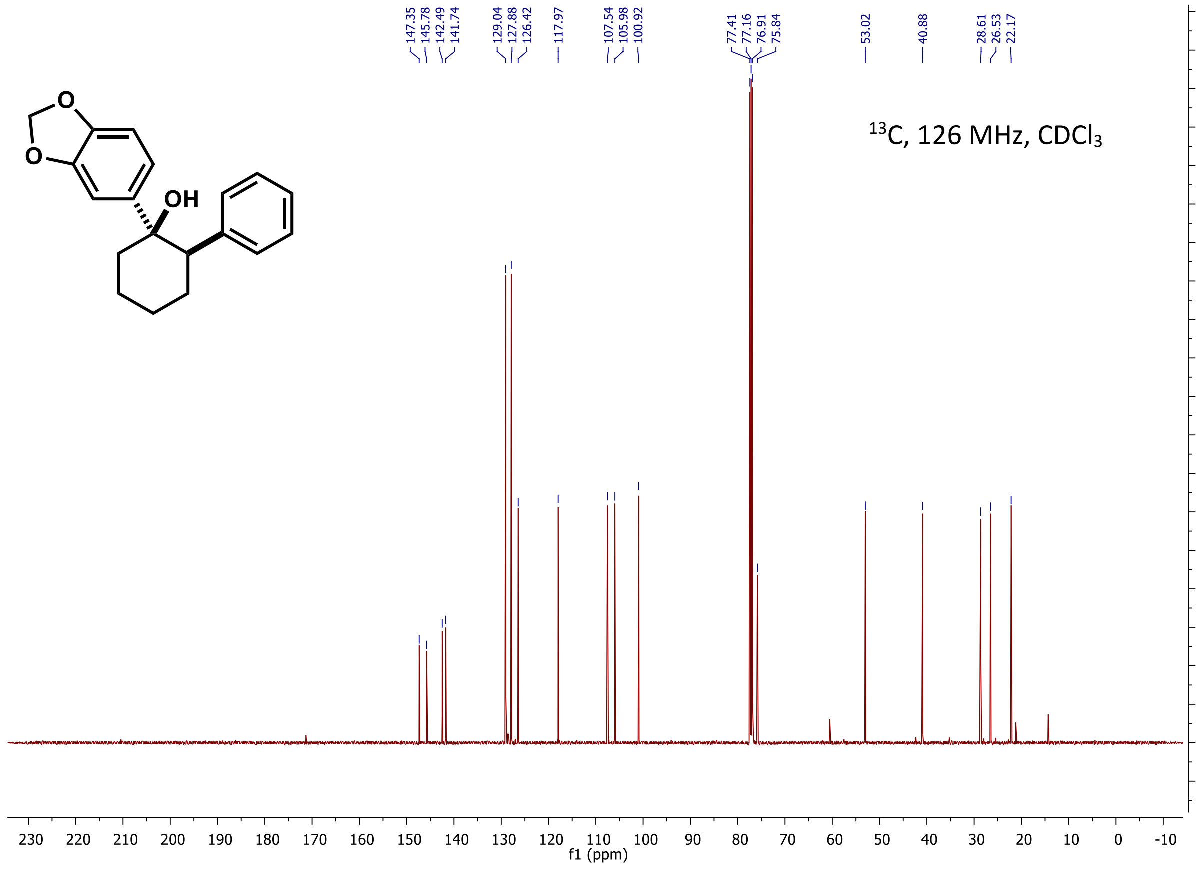
147.35 145.78 142.49 141.74 129.04 127.88 126.42 117.97 107.54 105.98 100.92 77.41 77.16 76.91 75.84 53.02 40.88 28.61 26.53 22.17
O
O
OH
^{13}C, 126 MHz, CDCl$_3$
230 220 210 200 190 180 170 160 150 140 130 120 110 100 90 80 70 60 50 40 30 20 10 0 -10
f1 (ppm)

(S15)

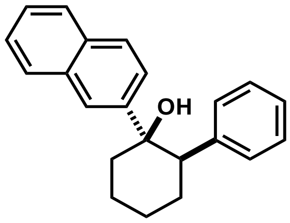

Prepared according to General Procedure A part B(2) using 2-bromonaphthalene (1.35 g, 6.5 mmol), n-butyllithium (2.6 mL, 6.5 mmol, 2.5 M in hexanes), THF (16 mL) and 2-phenylcyclohexanone (0.87 g, 5 mmol). The crude residue was purified by flash column chromatography (eluent = 2% EtOAc in hexanes, silica gel) to afford product as a white solid (1.01 g, 67% yield).

Mp.: 67-69 °C; **R_f** = 0.38 (eluent = 10% EtOAc in hexanes); **ν_{max} / cm^{-1}** (thin film) 3531, 3455, 3022, 2932, 2851, 1597, 1490, 1447, 1240; **^{1}H NMR (500 MHz, $CDCl_3$)** δ_H: 1.59 (1H, app. qt, *J* 12.8, 3.5 Hz), 1.72-2.06 (6H, m), 2.10-2.21 (1H, m), 2.30 (1H, app. qd, *J* 13.1, 3.6 Hz), 3.23 (1H, dd, *J* 12.8, 3.7 Hz), 6.90-6.97 (2H, m), 6.97-7.03 (3H, m), 7.37-7.43 (2H, m), 7.45 (1H, dd, *J* 8.6, 1.9 Hz), 7.63 (1H, d, *J* 1.6 Hz), 7.66-7.80 (3H, m); **^{13}C NMR (126 MHz, $CDCl_3$)** δ_C: 22.2, 26.6, 28.5, 40.7, 52.4, 76.1, 123.4, 123.9, 125.6, 125.9, 126.4, 127.4, 127.5, 127.9, 128.3, 129.0, 132.2, 133.2, 141.6; **HRMS (CI^+)** [$C_{22}H_{22}O$] requires $[M]^+$ 302.1671, found 302.1665 (- 2.0 ppm).

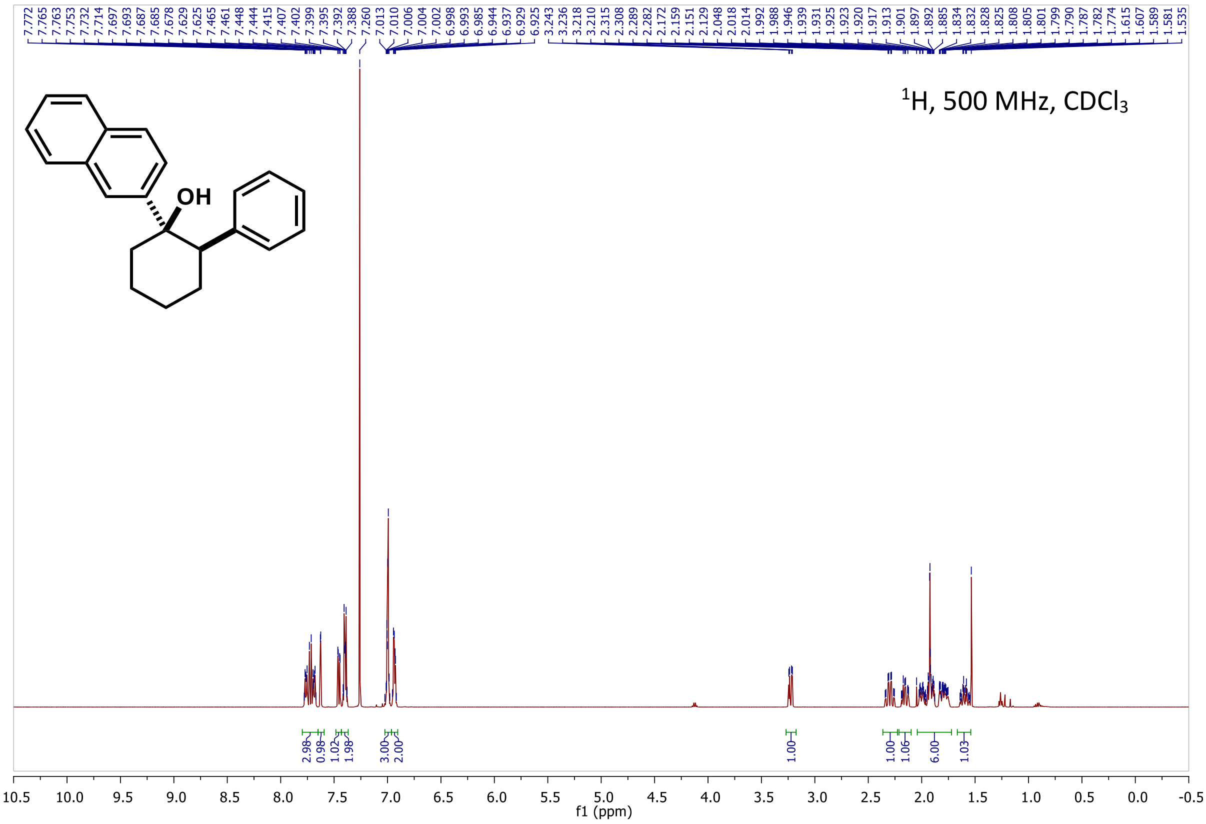
1H, 500 MHz, CDCl3
OH
f1 (ppm)

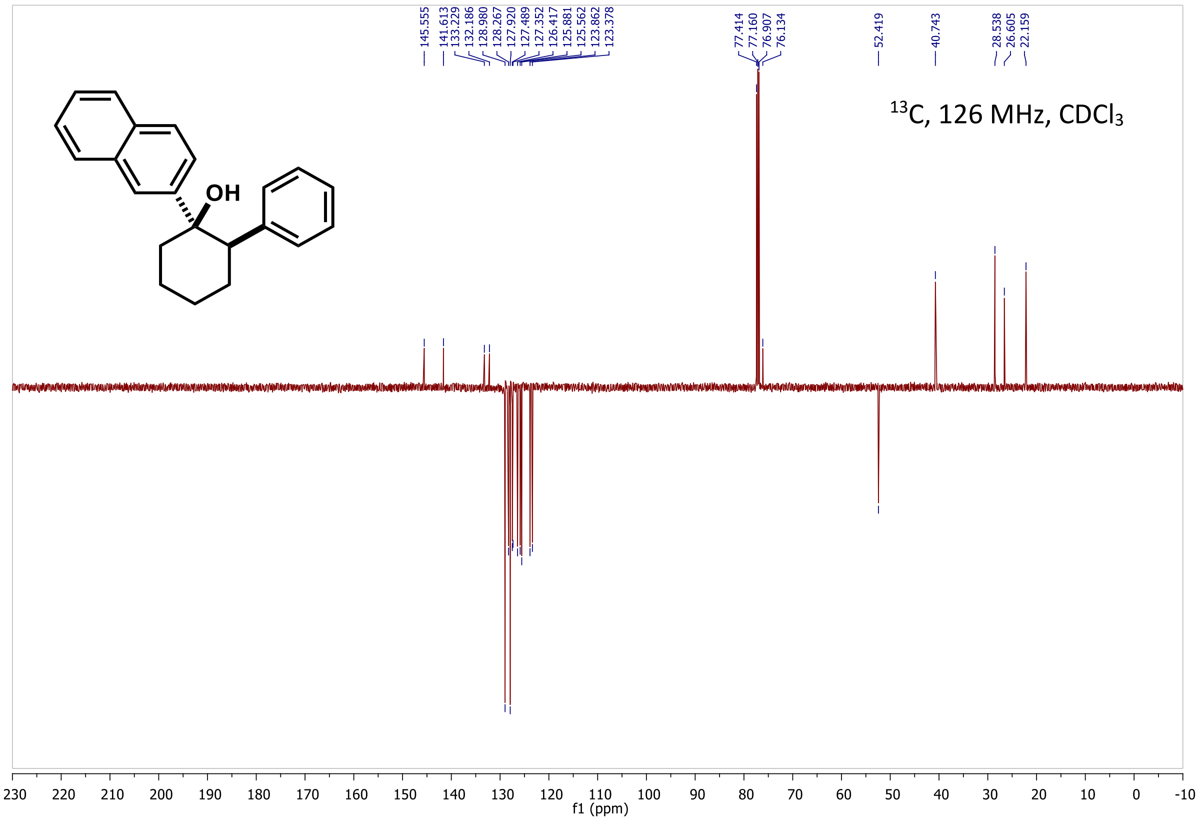
13C, 126 MHz, CDCl3
OH
f1 (ppm)

(S16)

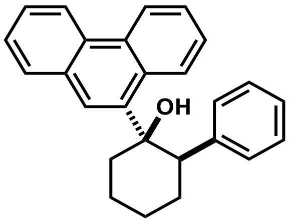

Prepared according to General Procedure A part B (2) using 9-bromophenanthrene (1.67 g, 6.5 mmol), n-butyllithium (2.6 mL, 6.5 mmol, 2.5 M in hexanes), THF (16 mL) and 2-phenylcyclohexanone (0.87 g, 5 mmol). The crude residue was purified by flash column chromatography (eluent = 2% EtOAc in hexanes, silica gel) to afford product as a yellow solid (1.25 g, 72% yield).

Mp.: 71-73 °C; **R_f** = 0.38 (eluent = 10% EtOAc in hexanes); **ν_{max} / cm^{-1}** (thin film) 3547, 3084, 3057, 2941, 2916, 2850, 1629, 1599, 1348, 1172, 929; **^{1}H NMR (500 MHz, $CDCl_3$)** δ_H: 1.72-1.96 (4H, m), 1.98-2.13 (2H, m), 2.34-2.52 (1H, m), 2.94 (1H, t, *J* 12.4 Hz), 4.15-4.28 (1H, m), 6.73 (2H, d, *J* 6.3 Hz), 6.84-7.01 (3H, m), 7.43-7.52 (1H, m), 7.53-7.61 (1H, m), 7.61-7.79 (4H, m), 8.56-8.65 (1H, m), 8.69-8.77 (1H, m), 8.79-8.88 (1H, m); **^{13}C NMR (126 MHz, $CDCl_3$)** δ_C: 22.4, 26.4, 28.0, 37.8, 49.1, 76.6, 122.3, 124.1, 125.1, 125.6, 126.0, 126.5, 126.6, 126.7, 128.0, 128.4, 129.2, 129.8, 130.0, 131.5, 131.8, 141.2; **HRMS (CI$^+$)** [$C_{26}H_{24}O$] requires [M]$^+$ 352.1827, found 352.1821 (- 1.7 ppm).

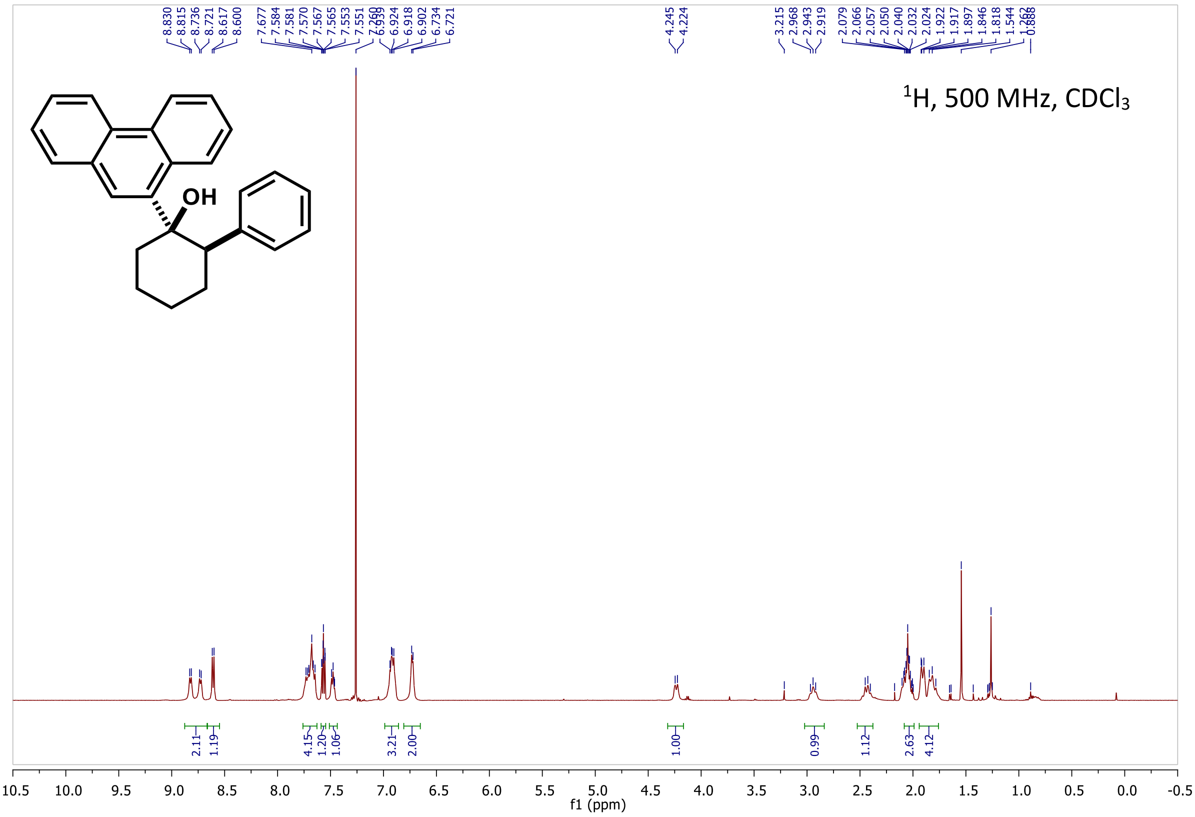

8.830
8.815
8.736
8.721
8.617
8.600
7.677
7.584
7.581
7.570
7.567
7.565
7.553
7.551
6.924
6.918
6.902
6.734
6.721
4.245
4.224
3.215
2.968
2.943
2.919
2.079
2.066
2.057
2.050
2.040
2.032
2.024
1.922
1.917
1.897
1.846
1.818
1.544
OH
^{1}H, 500 MHz, CDCl$_3$
2.11
1.19
4.15
1.20
1.06
3.21
2.00
1.00
0.99
1.12
2.63
4.12
f1 (ppm)

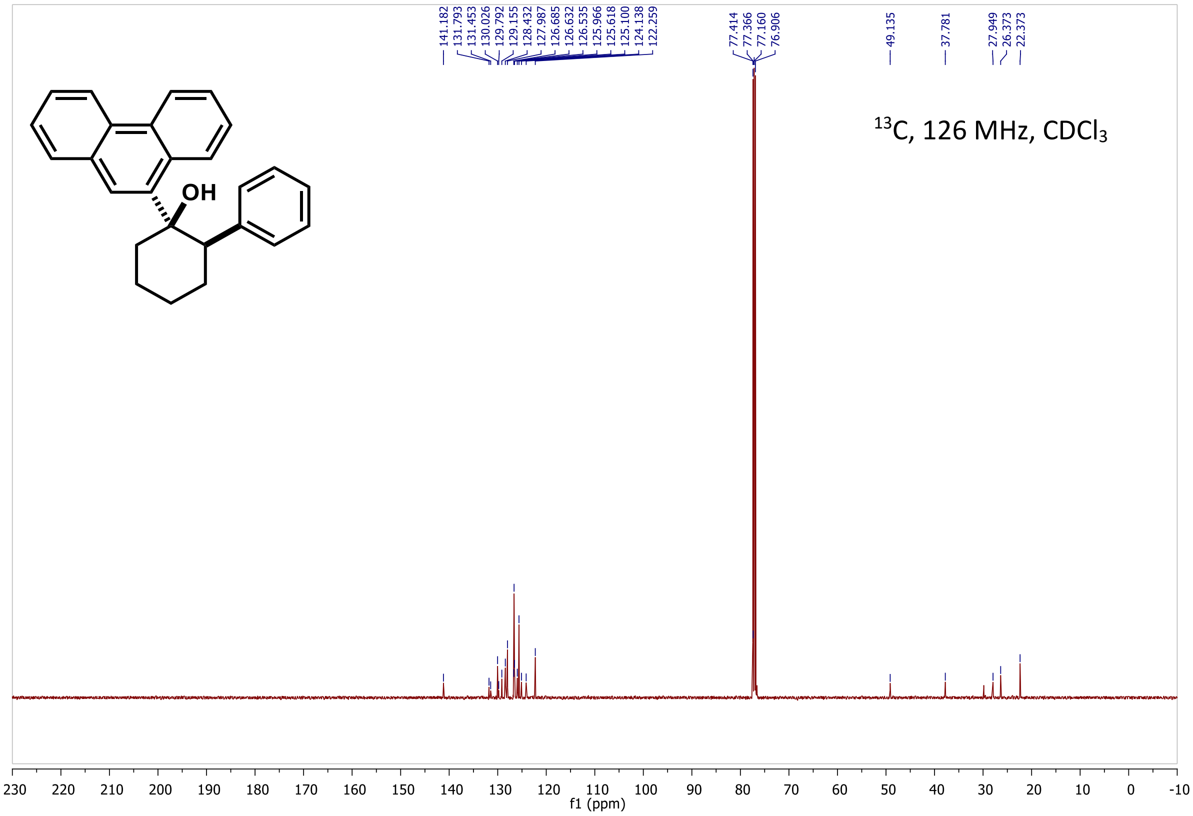

141.182
131.793
131.453
130.026
129.792
129.155
128.432
127.987
126.685
126.632
126.535
125.966
125.618
125.100
124.138
122.259
77.414
77.366
77.160
76.906
49.135
37.781
27.949
26.373
22.373
OH
^{13}C, 126 MHz, CDCl$_3$
f1 (ppm)

(S17)

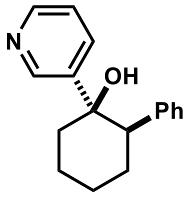

To a solution of 3-bromopyridine (1.53 g, 9.7 mmol, 1.5 equiv.) in Et_2O (30 mL) at -78 °C was added *n*-butyllithium (4.41 mL, 9.7 mmol, 1.5 equiv.) dropwise. The mixture was stirred at -78 °C for 30 minutes before a solution of 2-phenylcyclohexanone (1.13 g, 6.5 mmol, 1 equiv.) in Et_2O (6.5 mL) was added and the mixture was stirred up to room temperature and allowed to stir for 4 h. The reaction was quenched with water (20 mL) and extracted with EtOAc (50 mL). The layers were separated and the aqueous extracted with EtOAc (2 x 50 mL), organics combined, dried over $MgSO_4$, filtered and concentrated *in vacuo* yielding crude product. The crude residue was purified by flash column chromatography (eluent = 30 to 60% EtOAc in hexanes, silica gel) to afford product as a white solid (0.63 g, 38% yield).

Mp.: 125-127 °C; $\mathbf{R_f}$ = 0.30 (eluent = 50% EtOAc in hexanes); $\boldsymbol{\nu_{max}}$ **/ cm^{-1}** (thin film) 3183, 2938, 1491, 1418, 1271, 1190, 1123; **^{1}H NMR (500 MHz, $CDCl_3$)** δ_H: 1.49-1.60 (1H, m), 1.72-1.82 (2H, m), 1.84-1.93 (2H, m), 1.94-2.00 (1H, m), 2.02-2.10 (1H, m), 2.24 (1H, app qd, *J* 13.1, 3.6 Hz), 2.98 (1H, dd, *J* 12.8, 3.6 Hz), 6.87-6.94 (2H, m), 7.03-7.10 (4H, m), 7.44-7.49 (1H, m), 8.31-8.34 (1H, m), 8.51-8.55 (1H, m); **^{13}C NMR (126 MHz, $CDCl_3$)** δ_C: 21.8, 26.4, 28.4, 40.3, 53.3, 74.8, 122.7, 126.6, 128.0, 129.1, 133.0, 141.1, 143.3, 146.9, 147.4; **HRMS (CI$^+$)** [$C_{17}H_{19}NO$] requires $[M+H]^+$ 254.1545, found 254.1537 (- 3.1 ppm).

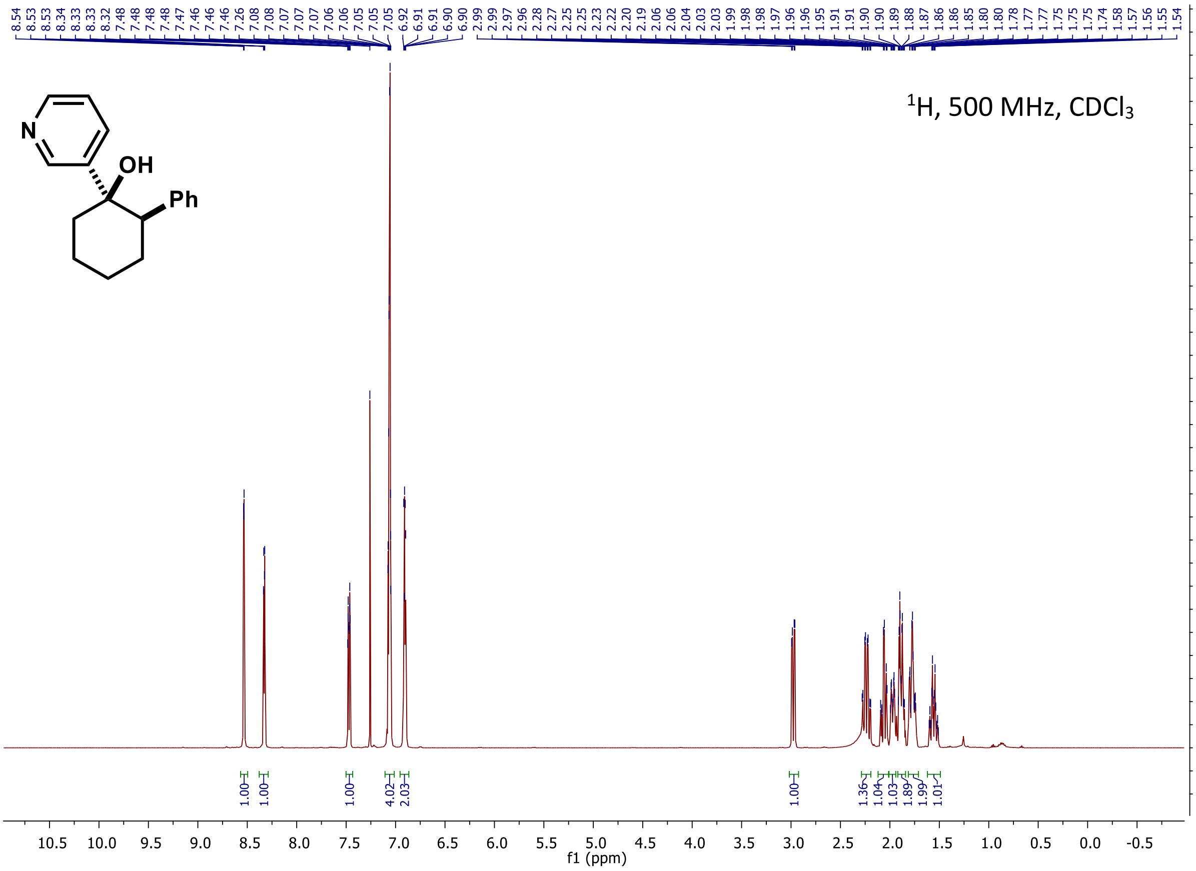

8.54 8.53 8.53 8.34 8.33 8.33 8.32 7.48 7.48 7.48 7.47 7.46 7.46 7.46 7.26 7.08 7.08 7.07 7.07 7.07 7.06 7.06 7.05 7.05 7.05 6.92 6.91 6.91 6.90 6.90 2.99 2.99 2.97 2.96 2.28 2.27 2.25 2.25 2.23 2.22 2.20 2.19 2.06 2.06 2.04 2.03 2.03 1.99 1.98 1.98 1.97 1.96 1.96 1.95 1.91 1.91 1.90 1.90 1.89 1.88 1.87 1.86 1.86 1.85 1.80 1.80 1.78 1.77 1.77 1.75 1.75 1.74 1.58 1.57 1.56 1.55 1.54
N
OH
Ph
^{1}H, 500 MHz, CDCl$_3$
1.00 1.00 1.00 4.02 2.03 1.00 1.36 1.04 1.03 1.89 1.99 1.01
10.5 10.0 9.5 9.0 8.5 8.0 7.5 7.0 6.5 6.0 5.5 5.0 4.5 4.0 3.5 3.0 2.5 2.0 1.5 1.0 0.5 0.0 -0.5
f1 (ppm)

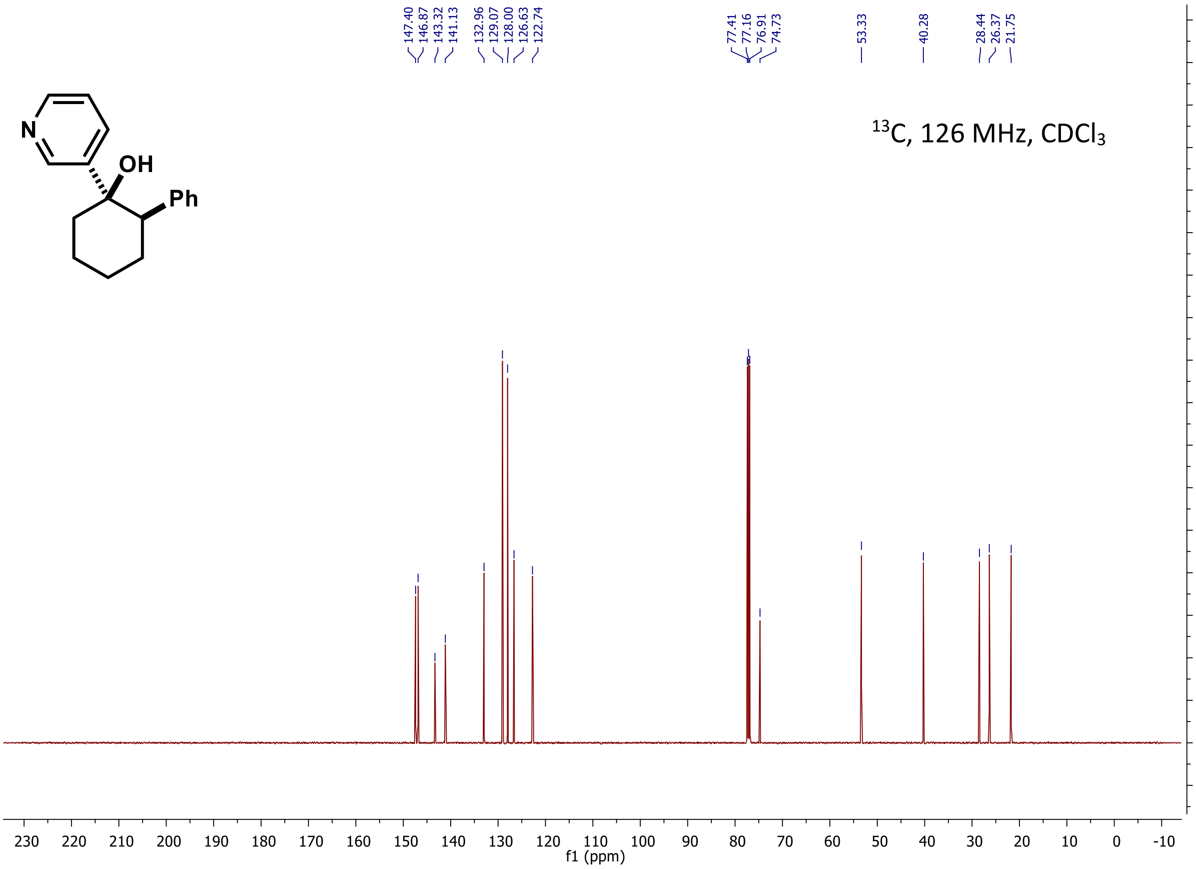

147.40 146.87 143.32 141.13 132.96 129.07 128.00 126.63 122.74 77.41 77.16 76.91 74.73 53.33 40.28 28.44 26.37 21.75
N
OH
Ph
^{13}C, 126 MHz, CDCl$_3$
230 220 210 200 190 180 170 160 150 140 130 120 110 100 90 80 70 60 50 40 30 20 10 0 -10
f1 (ppm)

(S18)

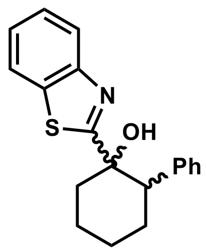

5:1 syn:anti

To a solution of benzothiazole (1.14 g, 8.45 mmol, 1.3 equiv.) in THF (20 mL) at -78 °C was added *n*-butyllithium (3.84 mL, 8.45 mmol, 1.3 equiv., 2.2 M in hexanes) dropwise forming a deep orange solution. The mixture was stirred at -78 °C for 30 minutes before a solution of 2-phenylcyclohexanone (1.13 g, 6.5 mmol, 1 equiv.) in THF (6.5 mL) was added and the mixture was allowed to stir up to room temperature and then stirred for 4 h. The reaction was quenched with water (20 mL) and extracted with EtOAc (50 mL). The layers were separated and the aqueous extracted with EtOAc (2 x 50 mL), organics combined, dried over $MgSO_4$, filtered and concentrated *in vacuo* yielding crude product as a 10:3 mixture of diastereomers. The crude residue was purified by flash column chromatography (eluent = 5 to 10% EtOAc in hexanes, silica gel) to afford product as an orange solid (0.89 g, 44% yield) as a 5:1 mixture of diastereomers.

Mp.: 87-89 °C **R_f** = 0.32 (eluent = 20% EtOAc in hexanes); **ν_{max} / cm^{-1}** (thin film) 3541, 2930, 1493, 1437, 1314; **HRMS (ES$^+$)** [$C_{19}H_{20}NOS$] requires [M+H]$^+$ 310.1266, found 310.1258 (-2.6 ppm).

Selected data for major diastereomer:

^{1}H NMR (300 MHz, CDCl$_3$) δ_H: 7.35-7.41 (1H, m), 7.47-7.53 (1H, m), 7.82 (1H, dd, *J* 8.0, 0.6 Hz), 8.06 (1H, dd, *J* 8.2, 0.6 Hz); **^{13}C NMR (101 MHz, CDCl$_3$)** δ_C: 22.5, 26.3, 28.7, 40.2, 55.8, 77.6, 121.5, 123.2, 124.6, 125.7, 127.1, 128.2, 129.2, 134.8, 140.3, 153.3, 177.2.

Selected data for minor diastereomer:

^{1}H NMR (300 MHz, CDCl$_3$) δ_H: 7.29-7.34 (1H, m), 7.45-7.47 (1H, m), 7.77 (1H, dd, *J* 8.0, 0.6 Hz), 7.99 (1H, dd, *J* 8.2, 0.5 Hz); **^{13}C NMR (101 MHz, CDCl$_3$)** δ_C: 21.6, 26.0, 28.2, 40.0, 52.4, 77.4, 121.8, 122.8, 124.6, 125.9, 126.8, 128.2, 128.8, 135.2, 140.9, 153.1, 180.5.

Selected data for both diasteromers:

^{1}H NMR (300 MHz, CDCl$_3$) δ_H: 1.50-1.73 (1H, m), 1.80-2.05 (4H, m), 2.07-2.20 (1H, m), 2.24-2.51 (2H, m), 2.90-3.18 (2H, m); 7.04-7.18 (5H, m).

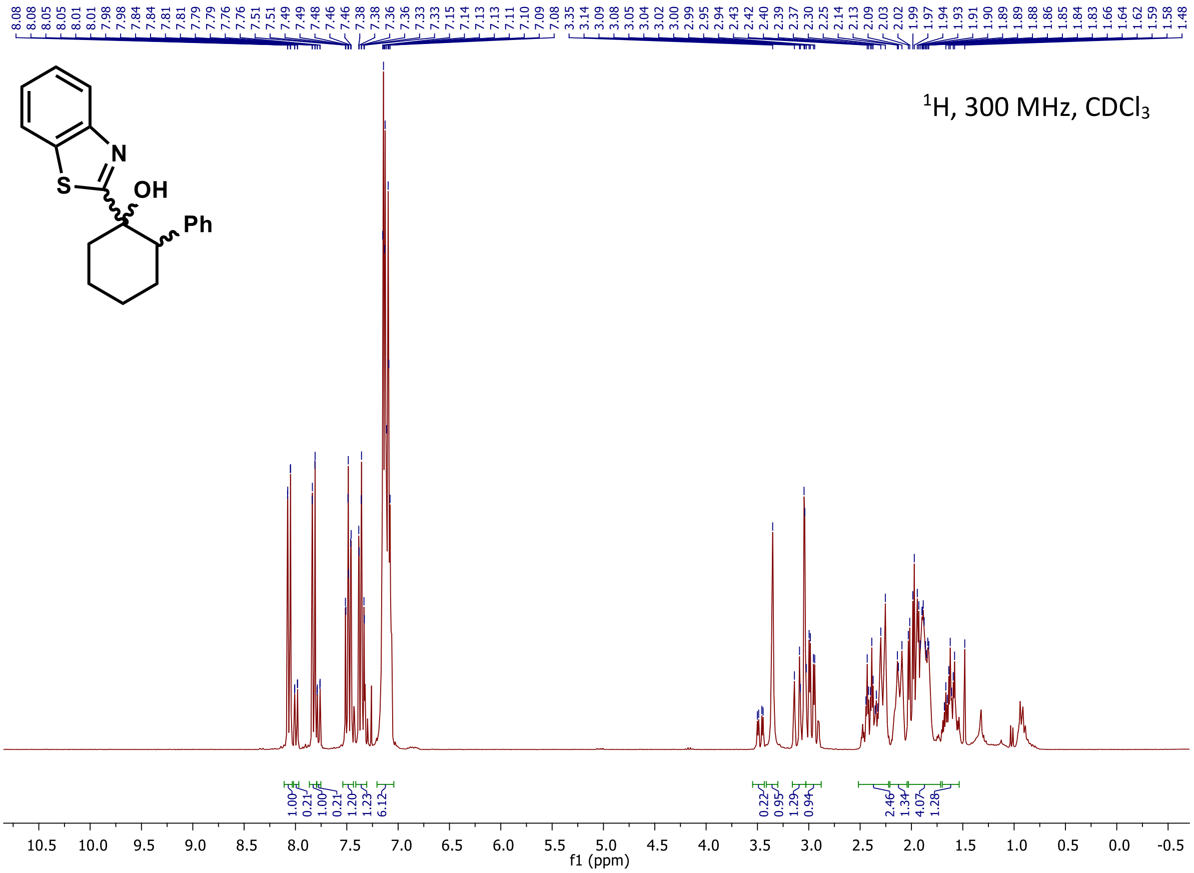
N
S
OH
Ph
1H, 300 MHz, CDCl3
1.00 0.21 1.00 0.21 1.20 1.23 6.12
0.22 0.95 1.29 0.94
2.46 1.34 4.07 1.28
f1 (ppm)

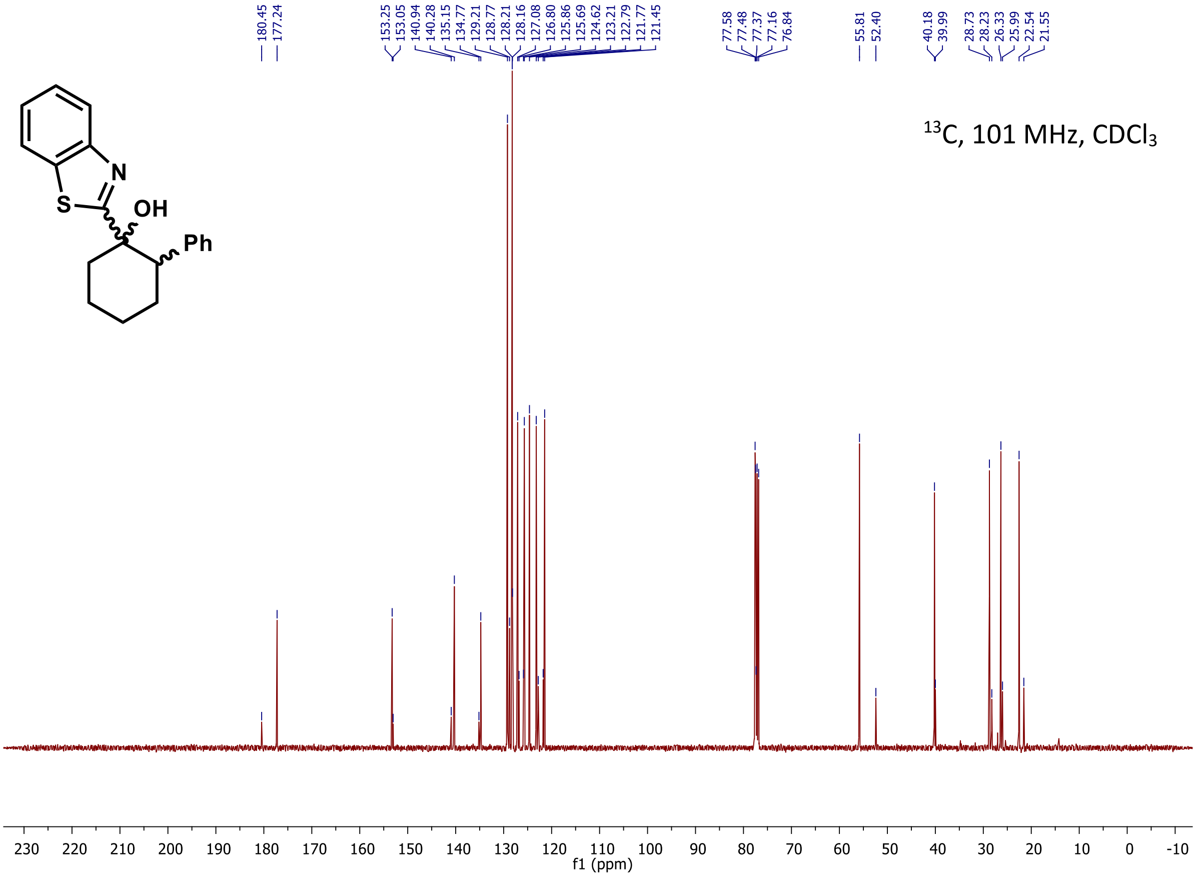
180.45 177.24
153.25 153.05 140.94 140.28 135.15 134.77 129.21 128.77 128.21 128.16 127.08 126.80 125.86 125.69 124.62 123.21 122.79 121.77 121.45
77.58 77.48 77.37 77.16 76.84
55.81 52.40
40.18 39.99
28.73 28.23 26.33 25.99 22.54 21.55
N
S
OH
Ph
13C, 101 MHz, CDCl3
f1 (ppm)

(S19)

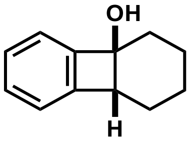

Prepared according to a literature procedure.[2]

(S20)

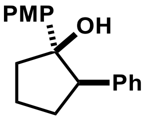

Prepared according to General Procedure A, part A using magnesium (1.44 g, 60 mmol), iodine (1 crystal), THF (100 mL), bromobenzene (7.85 g, 50 mmol), cyclopentene oxide (4.12 g, 42 mmol) and CuCl (395 mg, 4.2 mmol). The crude secondary alcohol (3.24 g, 20 mmol) was used directly in the next step with DMP (9.33 g, 22 mmol) and CH_2Cl_2 (40 mL). The crude residue was used directly in the next step without further purification.

Prepared according to General Procedure A part B(1) using magnesium (328 mg, 13.5 mmol), iodine (1 crystal), THF (25 mL, 0.5 M), 4-bromoanisole (2.10 g, 11.25 mmol) and 2-phenylcyclopentanone (1.20 g, 7.5 mmol). The crude residue was purified by flash column chromatography (eluent = 5 to 15% EtOAc in hexanes, silica gel) to afford product as a yellow oil (0.86 g, 43% yield).

R_f = 0.39 (eluent = 20% EtOAc in hexanes); **ν_{max} / cm^{-1}** (thin film) 3327, 1608, 1508, 1450, 1242, 1177, 1032; **^{1}H NMR (500 MHz, $CDCl_3$)** δ_H: 1.86-2.00 (1H, m), 2.07-2.21 (3H, m), 2.22-2.31 (1H, m), 2.33-2.45 (1H, m), 3.45 (1H, dd, *J* 11.4, 7.7 Hz), 3.80 (3H, s), 6.83 (2H, d, *J* 8.9 Hz), 7.00-7.02 (2H, m), 7.16-7.22 (3H, m), 7.25 (2H, d, *J* 8.9 Hz); **^{13}C NMR (126 MHz, $CDCl_3$)** δ_C: 22.6, 29.8, 43.2, 55.4, 57.6, 83.8, 113.4, 126.3, 127.0, 128.3, 129.0, 138.4, 138.6, 158.2; **HRMS (APCI$^+$)** [$C_{18}H_{20}O_2$] requires $[M-H_2O+H]^+$ 251.1436, found 251.1439 (+ 1.2 ppm).

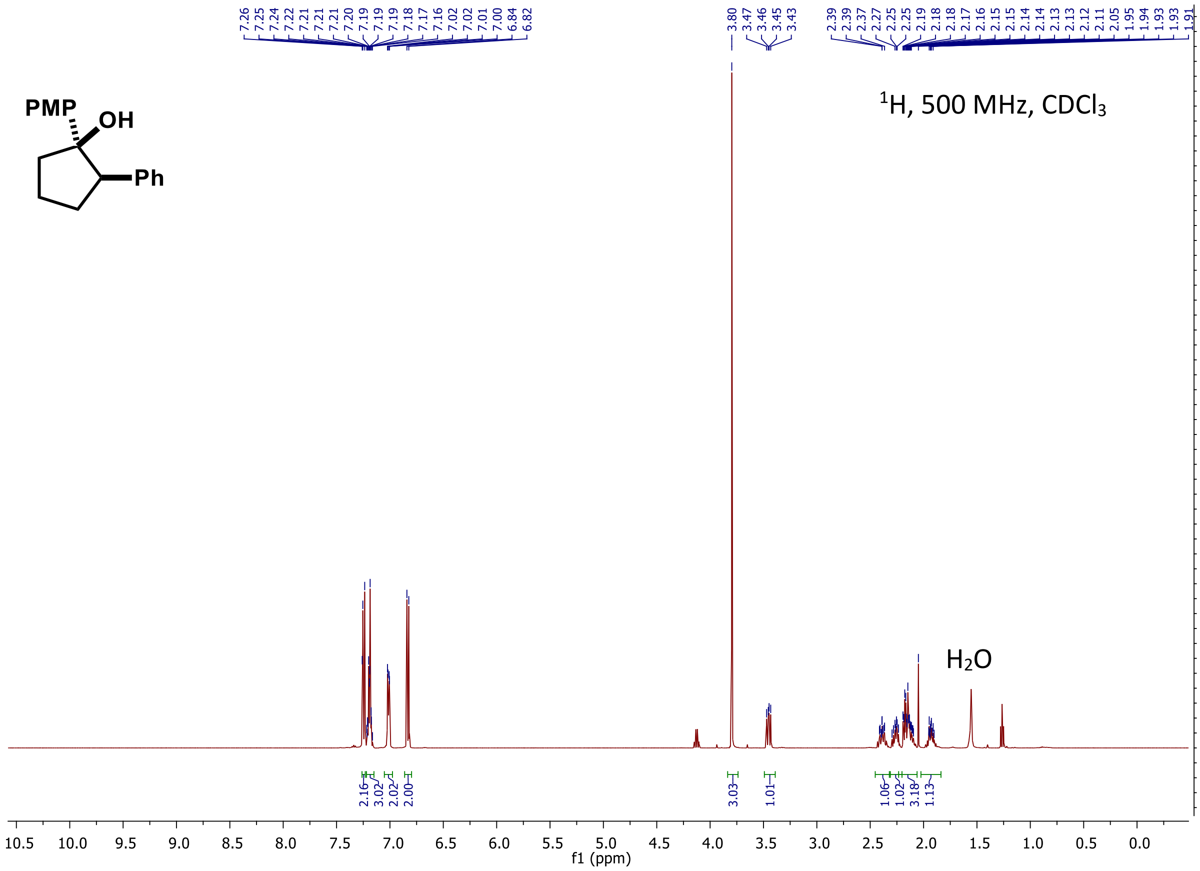
7.26 7.25 7.24 7.22 7.21 7.21 7.21 7.20 7.19 7.19 7.19 7.18 7.17 7.16 7.02 7.02 7.01 7.00 6.84 6.82
3.80 3.47 3.46 3.45 3.43
2.39 2.39 2.37 2.27 2.25 2.25 2.19 2.18 2.17 2.16 2.15 2.15 2.14 2.14 2.13 2.13 2.12 2.11 2.05 1.95 1.94 1.93 1.93 1.91
PMP
OH
Ph
^{1}H, 500 MHz, $CDCl_3$
H_2O
2.16 3.02 2.02 2.00
3.03
1.01
1.06 1.02 3.18 1.13
f1 (ppm)

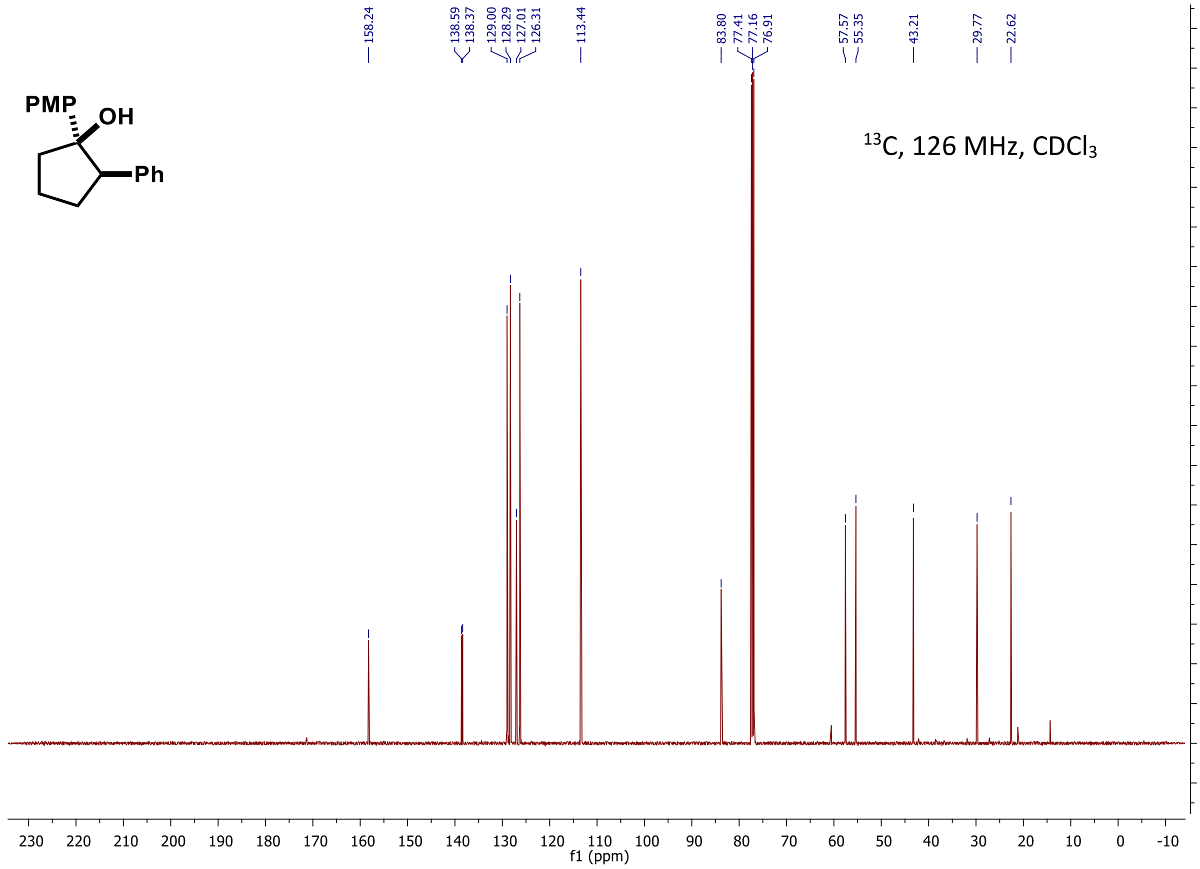
158.24
138.59 138.37 129.00 128.29 127.01 126.31
113.44
83.80 77.41 77.16 76.91
57.57 55.35
43.21
29.77
22.62
PMP
OH
Ph
^{13}C, 126 MHz, $CDCl_3$
f1 (ppm)

(S21)

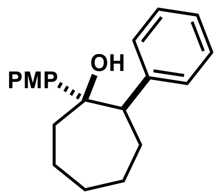

Prepared according to General Procedure A, part A using magnesium (1.70 g, 70 mmol), iodine (1 crystal), THF (120 mL), bromobenzene (9.10 g, 60 mmol), cycloheptene oxide (5.61 g, 50 mmol) and CuCl (495 mg, 5 mmol). The crude secondary alcohol (3.80 g, 20 mmol) was used directly in the next step with DMP (9.76 g, 22 mmol) and CH_2Cl_2 (40 mL). The crude residue was purified by flash column chromatography (eluent = 5-10 % EtOAc in hexanes, silica gel) to afford intermediate ketone as a colourless oil (2.35 g, 62% yield).

Prepared according to General Procedure A part B(2) using bromoanisole (1.12 g, 6 mmol), n-butyllithium (2.72 mL, 6 mmol, 2.2 M in hexanes), THF (20 mL) and 2-arylcyclohexanone (753 mg, 4 mmol). The crude residue was purified by flash column chromatography (eluent = 5 to 10% EtOAc in hexanes, silica gel) to afford product as a yellow oil (0.59 g, 49% yield).

R_f = 0.43 (eluent = 20% EtOAc in hexanes); **ν_{max} / cm^{-1}** (thin film) 3340, 2924, 1608, 1508, 1448, 1246, 1177, 1036; **^1H NMR (400 MHz, $CDCl_3$)** δ_H: 1.51-1.75 (4H, m), 1.75-1.88 (2H, m), 1.90-2.08 (3H, m), 2.17 (1H, dd, *J* 14.6, 10.5 Hz), 2.28-2.40 (1H, m), 3.19 (1H, d, *J* 10.0 Hz), 3.76 (3H, s), 6.75 (2H, d, *J* 8.7 Hz), 6.80-6.86 (2H, m), 7.02-7.11 (3H, m), 7.15 (2H, d, *J* 8.7 Hz); **^{13}C NMR (126 MHz, $CDCl_3$)** δ_C: 21.3, 28.4, 28.5, 29.6, 42.9, 55.3, 77.7, 113.1, 125.8, 126.2, 127.8, 129.3, 142.2, 143.5, 158.0; **HRMS (ES^+)** [$C_{20}H_{24}O_2$] requires $[M+Na]^+$ 319.1674, found 319.1674 (+ 0.0 ppm).

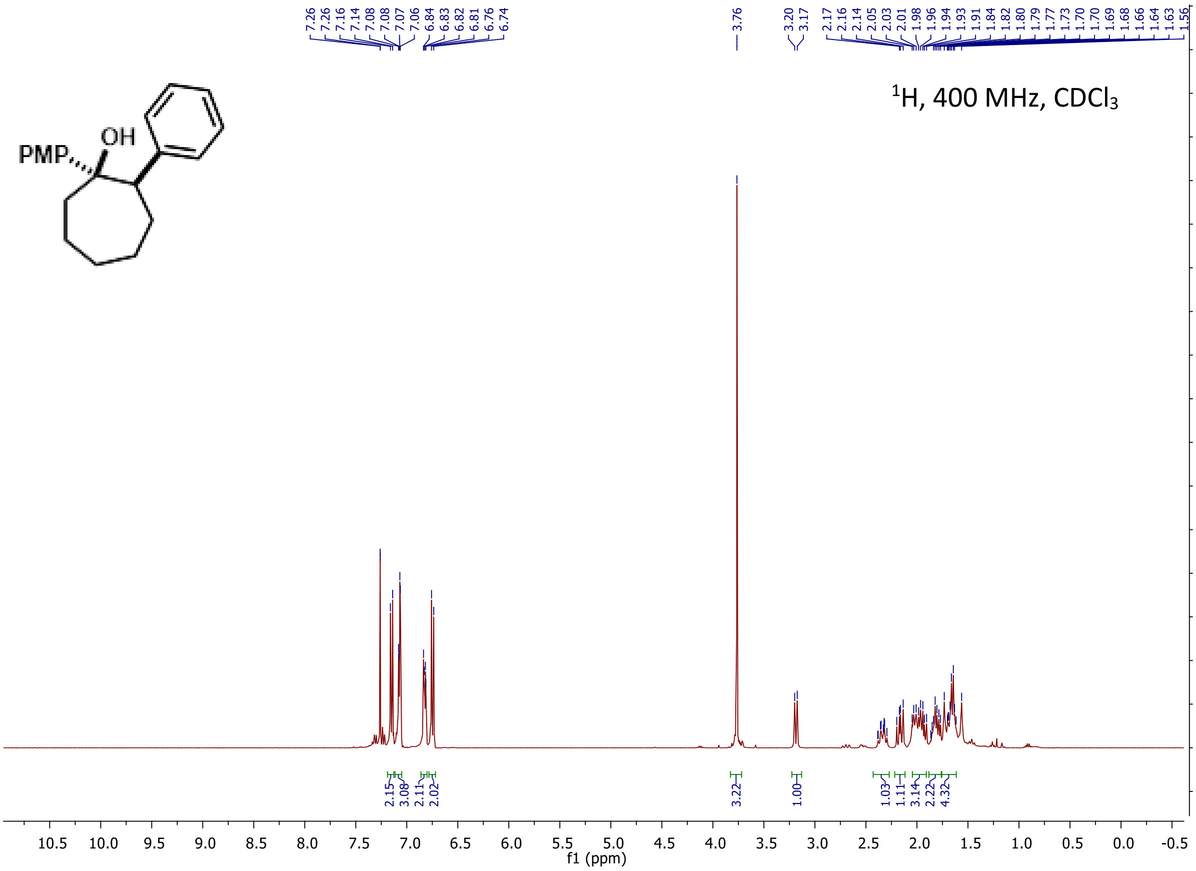
PMP
OH
1H, 400 MHz, CDCl3
7.26 7.26 7.16 7.14 7.08 7.08 7.07 7.06 6.84 6.83 6.82 6.81 6.76 6.74
3.76
3.20 3.17
2.17 2.16 2.14 2.05 2.03 2.01 1.98 1.96 1.94 1.93 1.91 1.84 1.82 1.80 1.79 1.77 1.73 1.70 1.69 1.68 1.66 1.64 1.63 1.56
2.15 3.08 2.11 2.02
3.22
1.00
1.03 1.11 3.14 2.22 4.32
f1 (ppm)

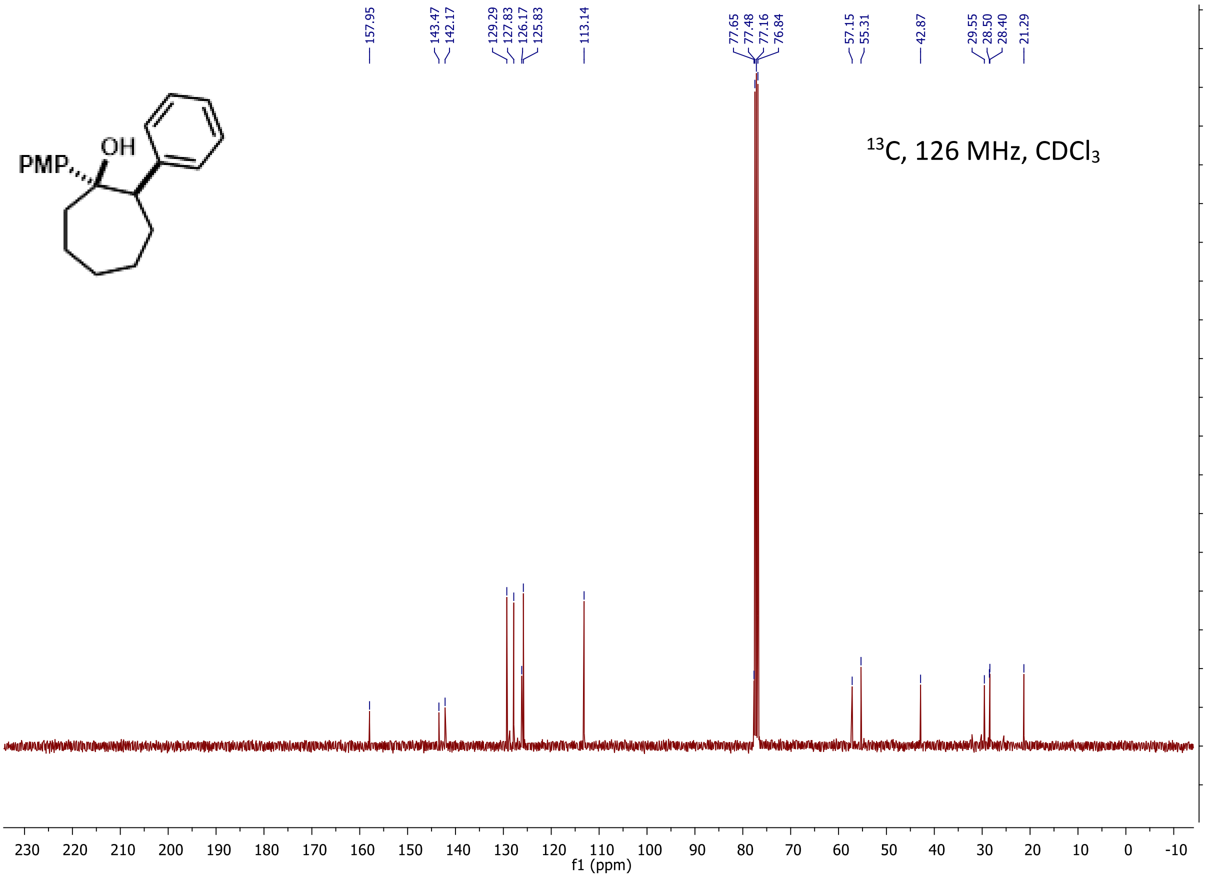
PMP
OH
13C, 126 MHz, CDCl3
157.95
143.47 142.17
129.29 127.83 126.17 125.83
113.14
77.65 77.48 77.16 76.84
57.15 55.31
42.87
29.55 28.50 28.40 21.29
f1 (ppm)

(S22)

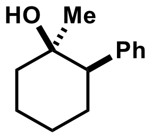

Prepared according to general procedure B using 2-phenylcyclohexanone (750 mg, 4.30 mmol), methylmagnesium bromide (3 mL, 3M in Et_2O) and THF (20 mL). The crude residue was purified by flash column chromatography (eluent = 5 to 15% EtOAc in hexanes, silica gel) to afford product as a colourless oil (670 mg, 82% yield).

R_f = 0.31 (eluent = 15% EtOAc in hexanes); **ν_{max} / cm^{-1}** (thin film) 3487, 2927, 1601, 1493, 1447, 1373, 1151, 1098; **^{1}H NMR (500 MHz, $CDCl_3$)** δ_H: 0.98 (3H, s), 1.18-1.23 (1H, m), 1.35 (1H, app qt, *J* 13.0, 3.7 Hz), 1.44-1.54 (1H, m), 1.58-1.64 (2H, m), 1.68-1.77 (1H, m), 1.78-1.88 (2H, m), 1.99-2.12 (1H, m), 2.50 (1H, dd, *J* 13.0, 3.5 Hz), 7.21-7.27 (3H, m), 7.28-7.34 (2H, m); **^{13}C NMR (126 MHz, $CDCl_3$)** δ_C: 22.1, 26.6, 28.6, 30.2, 40.2, 53.3, 70.8, 126.6, 128.2, 129.2, 143.0; **HRMS (ASAP)** [$C_{13}H_{18}O$] requires $[M-H_2O+H]^+$ 173.1330, found 173.1333 (+ 1.7 ppm).

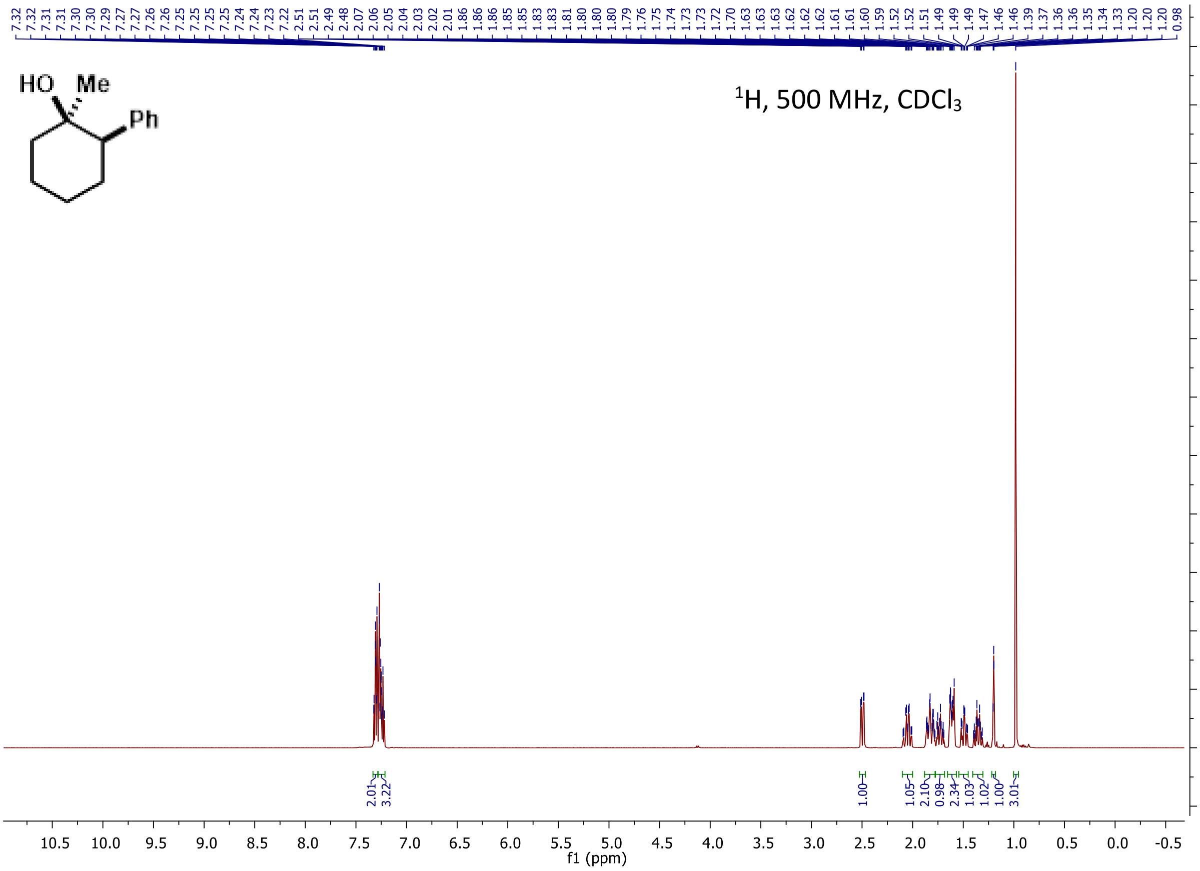
HO Me
Ph
^{1}H, 500 MHz, CDCl$_3$
f1 (ppm)

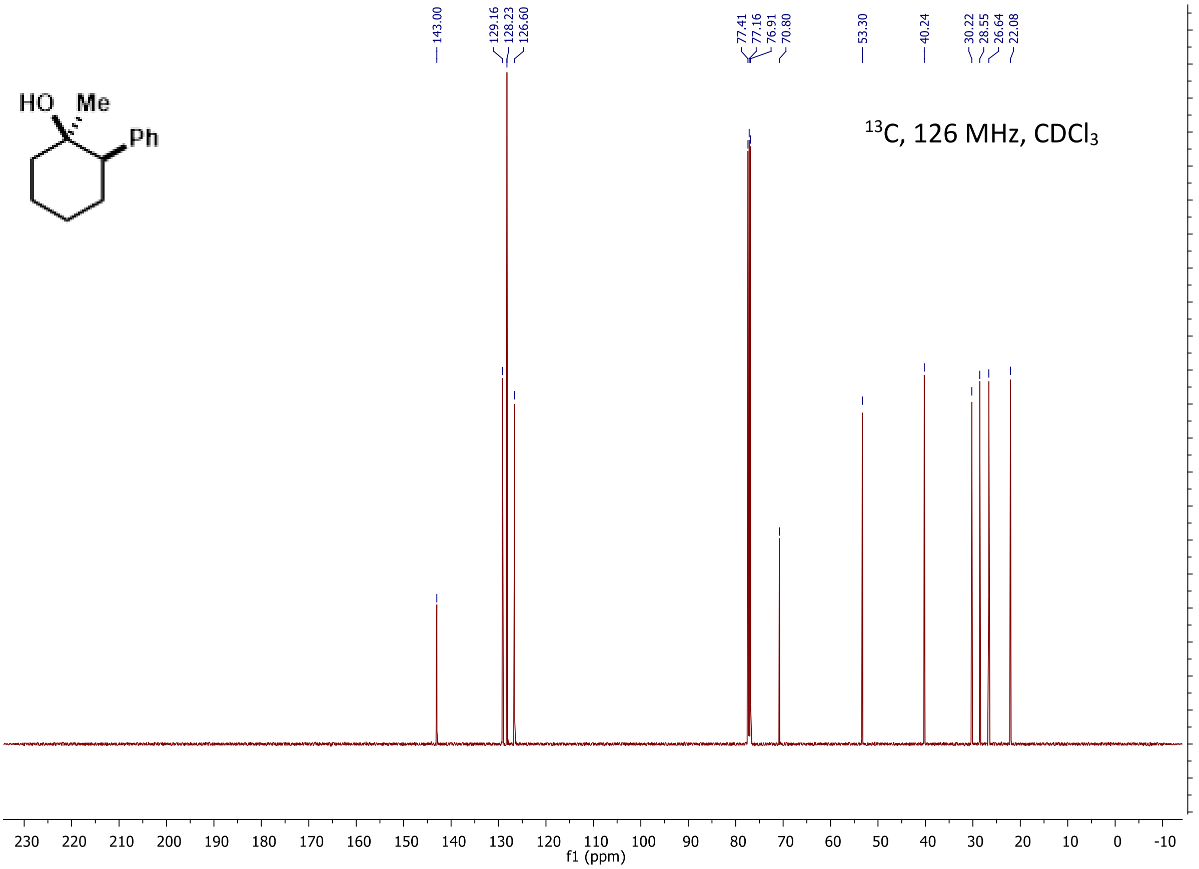
HO Me
Ph
^{13}C, 126 MHz, CDCl$_3$
143.00
129.16
128.23
126.60
77.41
77.16
76.91
70.80
53.30
40.24
30.22
28.55
26.64
22.08
f1 (ppm)

(S23)

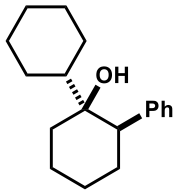

Prepared according to general procedure A, part B(1) using 2-phenylcyclohexanone (2.60 g, 15 mmol), bromocyclohexane (4.89 g, 30 mmol), magnesium turnings (875 mg, 36 mmol) and THF (60 mL). The crude residue was purified by flash column chromatography (eluent = 5 to 10% EtOAc in hexanes, silica gel) to afford product as a yellow solid (0.91 g, 23% yield).

Mp.: 58-60 °C, **R_f** = 0.55 (eluent = 20% EtOAc in hexanes); **ν_{max} / cm^{-1}** (thin film) 3535, 1438, 1278, 1049, 964; **^{1}H NMR (400 MHz, $CDCl_3$)** δ_H: 0.87-1.12 (5H, m), 1.22-1.50 (4H, m), 1.53-1.84 (9H, m), 1.91 (1H, s (br)), 2.01-2.17 (1H, m), 2.81 (1H, dd, *J* 12.7, 3.6 Hz), 7.18-7.26 (1H, m), 7.27-7.36 (4H, m); **^{13}C NMR (126 MHz, $CDCl_3$)** δ_C: 21.6, 26.0, 26.6, 26.7, 26.8, 26.8, 28.7, 29.8, 31.5, 46.3, 48.9, 75.3, 126.4, 128.3, 129.0, 143.5;); **HRMS (CI$^+$)** [$C_{18}H_{26}O$] requires $[M]^+$ 258.1984, found 258.1983 (- 0.3 ppm).

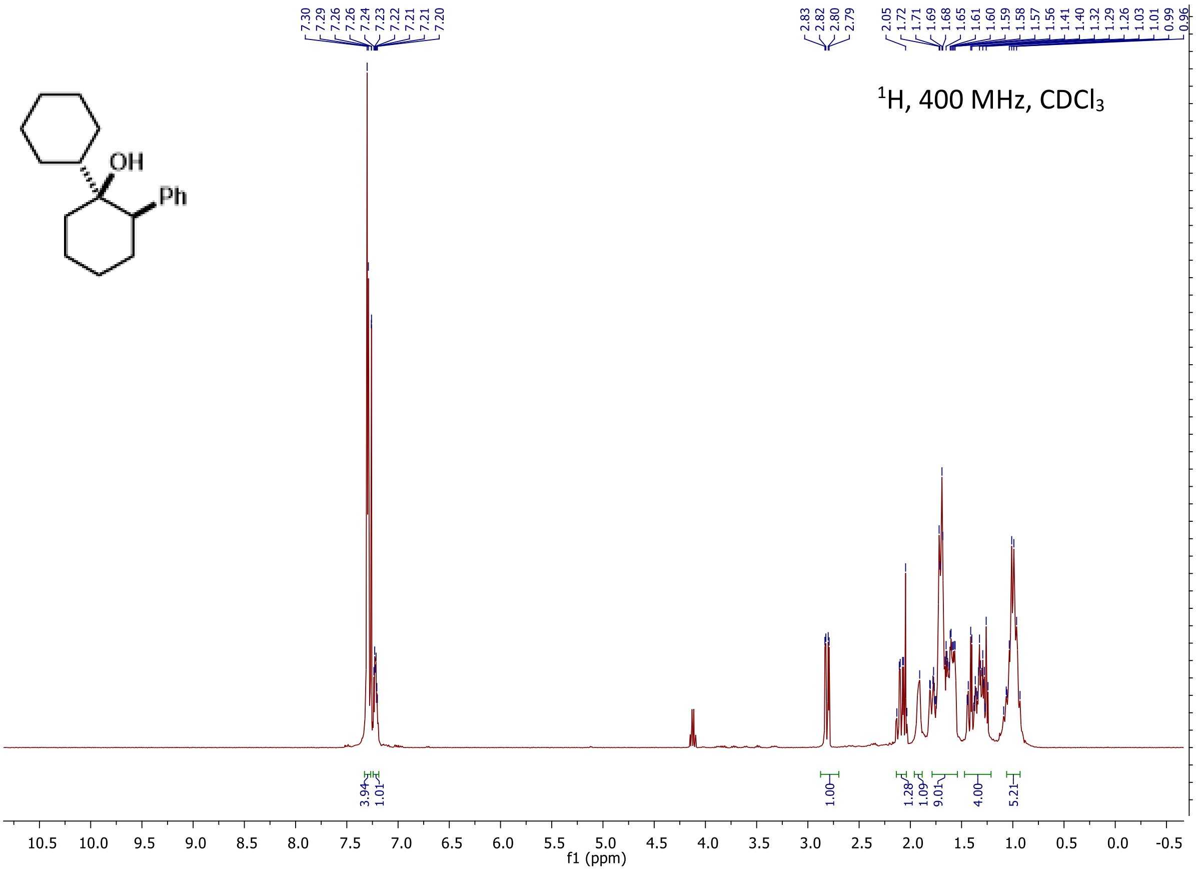

^{1}H, 400 MHz, CDCl$_3$
OH
Ph
7.30 7.29 7.26 7.24 7.23 7.22 7.21 7.21 7.20
2.83 2.82 2.80 2.79
2.05 1.72 1.71 1.69 1.68 1.65 1.61 1.60 1.59 1.58 1.57 1.56 1.41 1.40 1.32 1.29 1.26 1.03 1.01 0.99 0.96
3.94 1.01 1.00 1.28 1.09 9.01 4.00 5.21
f1 (ppm)

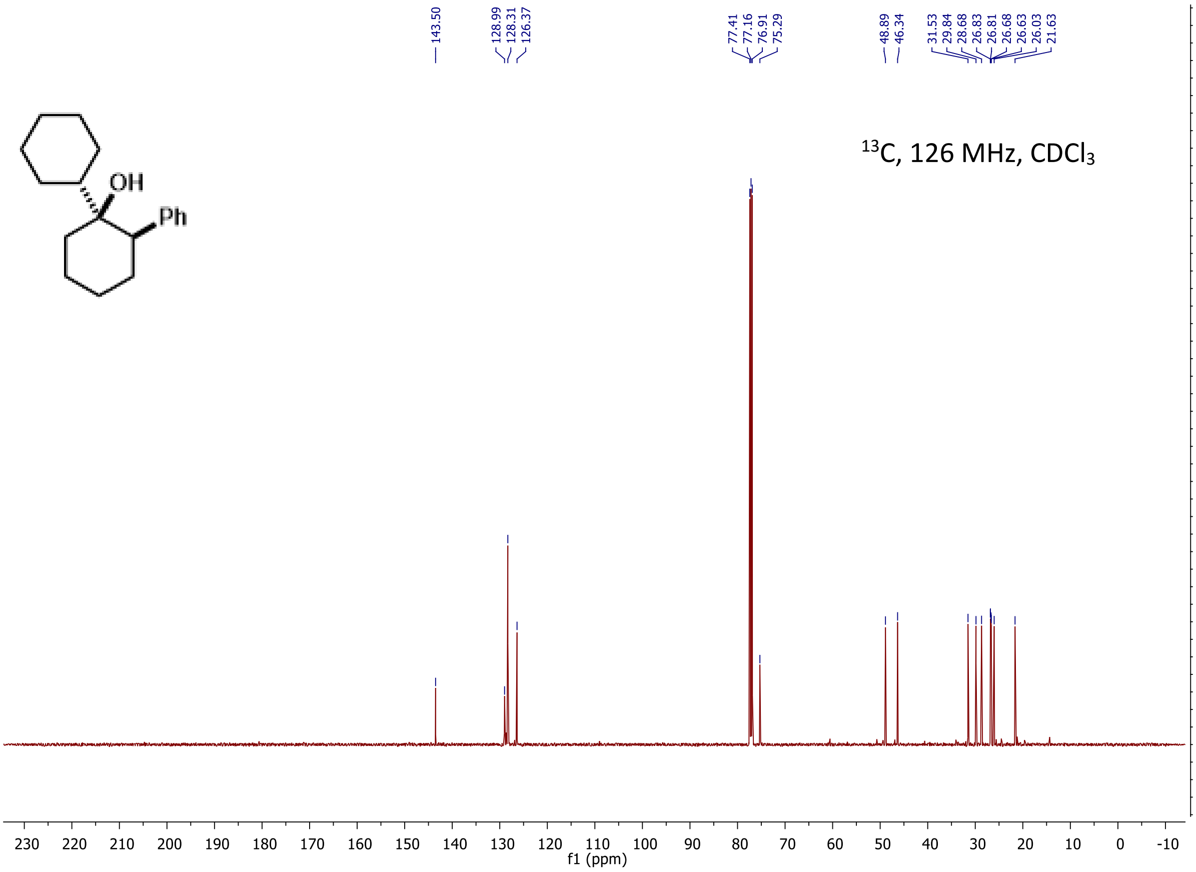

^{13}C, 126 MHz, CDCl$_3$
OH
Ph
143.50
128.99 128.31 126.37
77.41 77.16 76.91 75.29
48.89 46.34
31.53 29.84 28.68 26.83 26.81 26.68 26.63 26.03 21.63
f1 (ppm)

(S24)

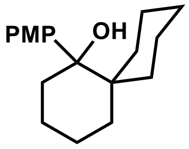

Intermediate ketone was prepared according to general procedure C part A using cyclohexanone (2.45 g, 25 mmol), toluene (50 mL), potassium tert-butoxide (5.90 g, 52.5 mmol) and 1,5 dibromopentane (5.75 g, 25 mmol). The crude oil was passed through a silica plug (eluent = 20% EtOAc in hexanes) affording crude product as a colourless oil (3.50 g, 84%).

Product was prepared according to general procedure C part B magnesium (432 mg, 18 mmol), iodine (1 crystal), THF (40 mL), 4-bromoanisole (2.80 g, 15 mmol) and spiro[5.5]undecan-1-one (1.66 g, 10 mmol). The crude residue was purified by flash column chromatography (eluent = 5% EtOAc in hexanes, silica gel) to afford product as a colourless oil (550 mg, 20% yield)

R_f = 0.34 (eluent = 10% EtOAc in hexanes); **ν_{max} / cm^{-1}** (thin film) 3489, 1609, 1513, 1462, 1240; **^{1}H NMR (500 MHz, $CDCl_3$)** δ_H: 0.67-0.98 (2H, m), 1.01-1.07 (1H, m), 1.30-1.68 (12H, m), 1.73-1.89 (2H, m), 1.97-2.08 (1H, m), 2.47 (1H, td, *J* 13.7, 4.5 Hz), 3.82 (3H, s), 6.85 (2H, d, *J* 9.0 Hz), 7.37 (2H, d, *J* 9.0 Hz);); **^{13}C NMR (126 MHz, $CDCl_3$)** δ_C: 20.7, 21.0, 21.3, 21.7, 26.1, 26.8, 28.7, 31.3, 34.7, 40.0, 55.4, 78.5, 112.4, 128.9, 138.0, 158.2; **HRMS (CI^+)** [$C_{18}H_{26}O_2$] requires $[M+H]^+$ 275.2011, found 275.2009 (- 0.7 ppm).

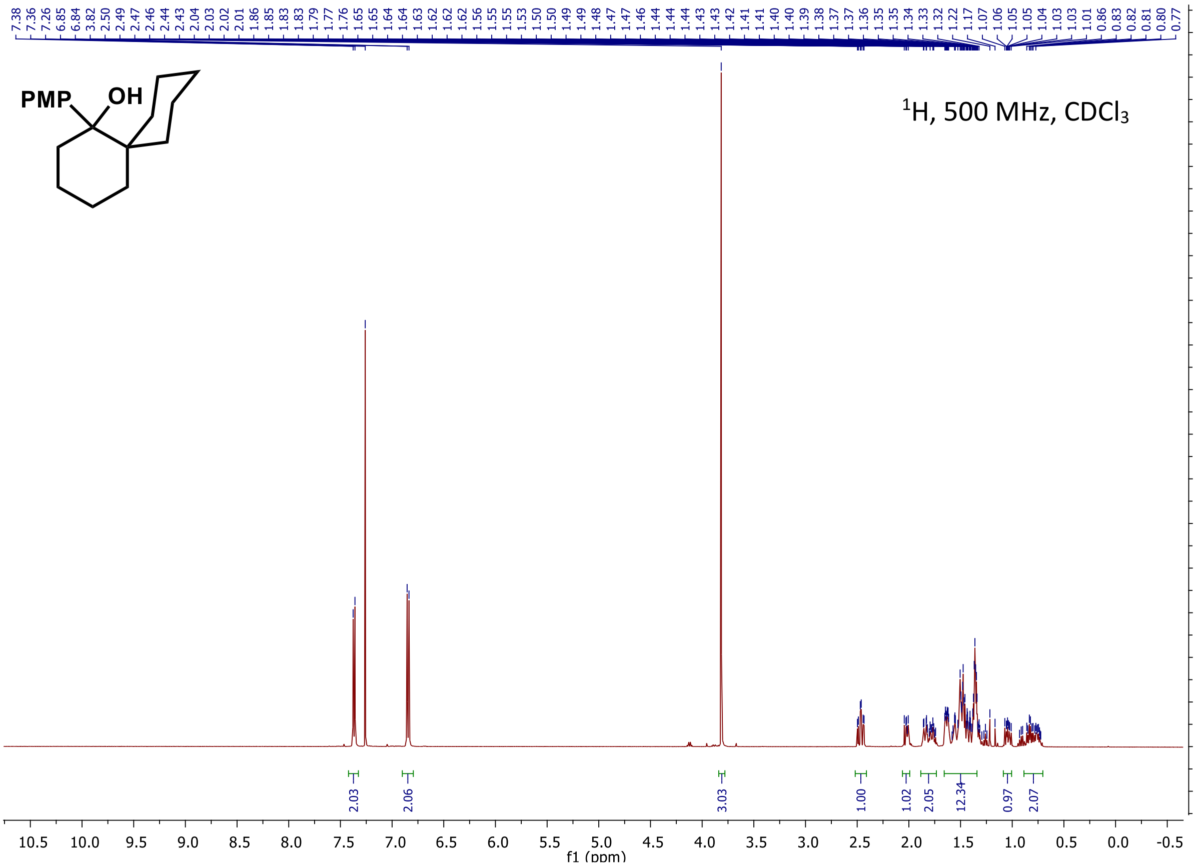
7.38
7.36
7.26
6.85
6.84
3.82
2.50
2.49
2.47
2.46
2.44
2.43
2.04
2.03
2.02
2.01
1.86
1.85
1.83
1.83
1.79
1.77
1.76
1.65
1.65
1.64
1.64
1.63
1.62
1.62
1.62
1.56
1.55
1.55
1.53
1.50
1.50
1.49
1.49
1.48
1.47
1.47
1.46
1.44
1.44
1.44
1.43
1.43
1.42
1.41
1.41
1.40
1.40
1.39
1.38
1.37
1.37
1.36
1.35
1.35
1.34
1.33
1.32
1.22
1.17
1.07
1.06
1.05
1.05
1.04
1.03
1.03
1.01
0.86
0.83
0.82
0.81
0.80
0.77
PMP
OH
^{1}H, 500 MHz, CDCl$_3$
2.03
2.06
3.03
1.00
1.02
2.05
12.34
0.97
2.07
10.5 10.0 9.5 9.0 8.5 8.0 7.5 7.0 6.5 6.0 5.5 5.0 4.5 4.0 3.5 3.0 2.5 2.0 1.5 1.0 0.5 0.0 -0.5
f1 (ppm)

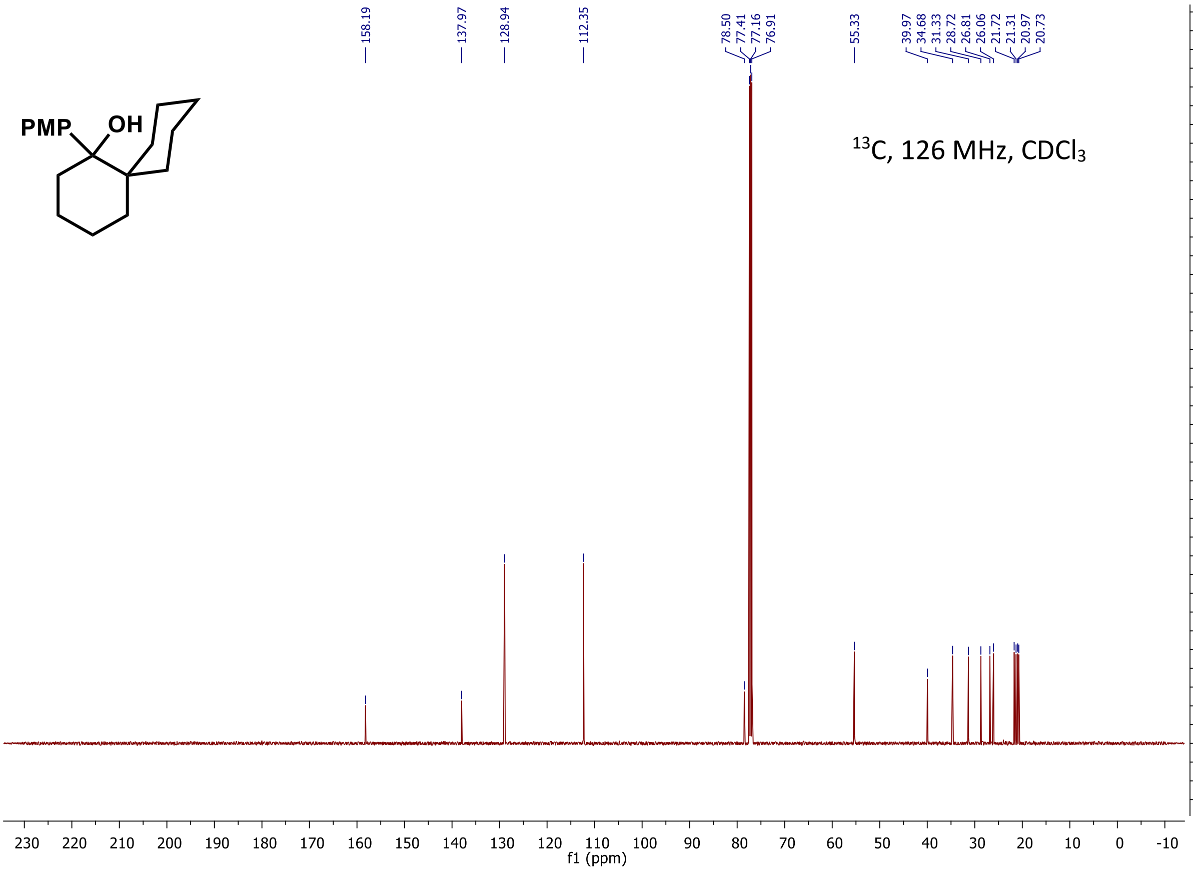
158.19
137.97
128.94
112.35
78.50
77.41
77.16
76.91
55.33
39.97
34.68
31.33
28.72
26.81
26.06
21.72
21.31
20.97
20.73
PMP
OH
^{13}C, 126 MHz, CDCl$_3$
230 220 210 200 190 180 170 160 150 140 130 120 110 100 90 80 70 60 50 40 30 20 10 0 -10
f1 (ppm)

(S25)

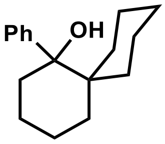

Intermediate ketone was prepared according to general procedure C part A using cyclohexanone (2.45 g, 25 mmol), toluene (50 mL), potassium tert-butoxide (5.90 g, 52.5 mmol) and 1,5 dibromopentane (5.75 g, 25 mmol). The crude oil was passed through a silica plug (eluent = 20% EtOAc in hexanes) affording crude product as a colourless oil (3.50 g, 84%).

Product was prepared according to general procedure C part B magnesium (576 mg, 24 mmol), iodine (1 crystal), THF (40 mL), 4-bromobenzene (3.14 g, 20 mmol) and spiro[5.5]undecan-1-one (2.26 g, 13.5 mmol). The crude residue was purified by flash column chromatography (eluent = 5% EtOAc in hexanes, silica gel) to afford product as a colourless oil (530 mg, 16% yield)

R_f = 0.55 (eluent = 5% EtOAc in toluene); **ν_{max} / cm^{-1}** (thin film) 3323, 2924, 1443; **^{1}H NMR (500 MHz, $CDCl_3$)** δ_H: 0.67-0.80 (1H, m), 0.80-0.92 (1H, m), 0.98-1.11 (1H, m), 1.29-1.41 (4H, m), 1.41-1.62 (5H, m), 1.62-1.71 (2H, m), 1.74-1.85 (1H, m), 1.88 (1H, dd, *J* 13.0, 1.9 Hz), 1.98-2.08 (1H, m), 2.51 (1H, td, *J* 13.7, 4.5 Hz), 7.22-7.26 (1H, m), 7.29-7.33 (2H, m), 7.44-7.49 (2H, m); **^{13}C NMR (126 MHz, $CDCl_3$)** δ_C: 20.7, 20.9, 21.3, 21.7, 26.0, 26.8, 28.7, 31.4, 34.5, 39.8, 78.7, 126.5, 127.0, 127.9, 145.8; **HRMS (CI^+)** [$C_{17}H_{24}O$] requires $[M]^+$ 244.1827, found 244.1824 (- 0.7 ppm).

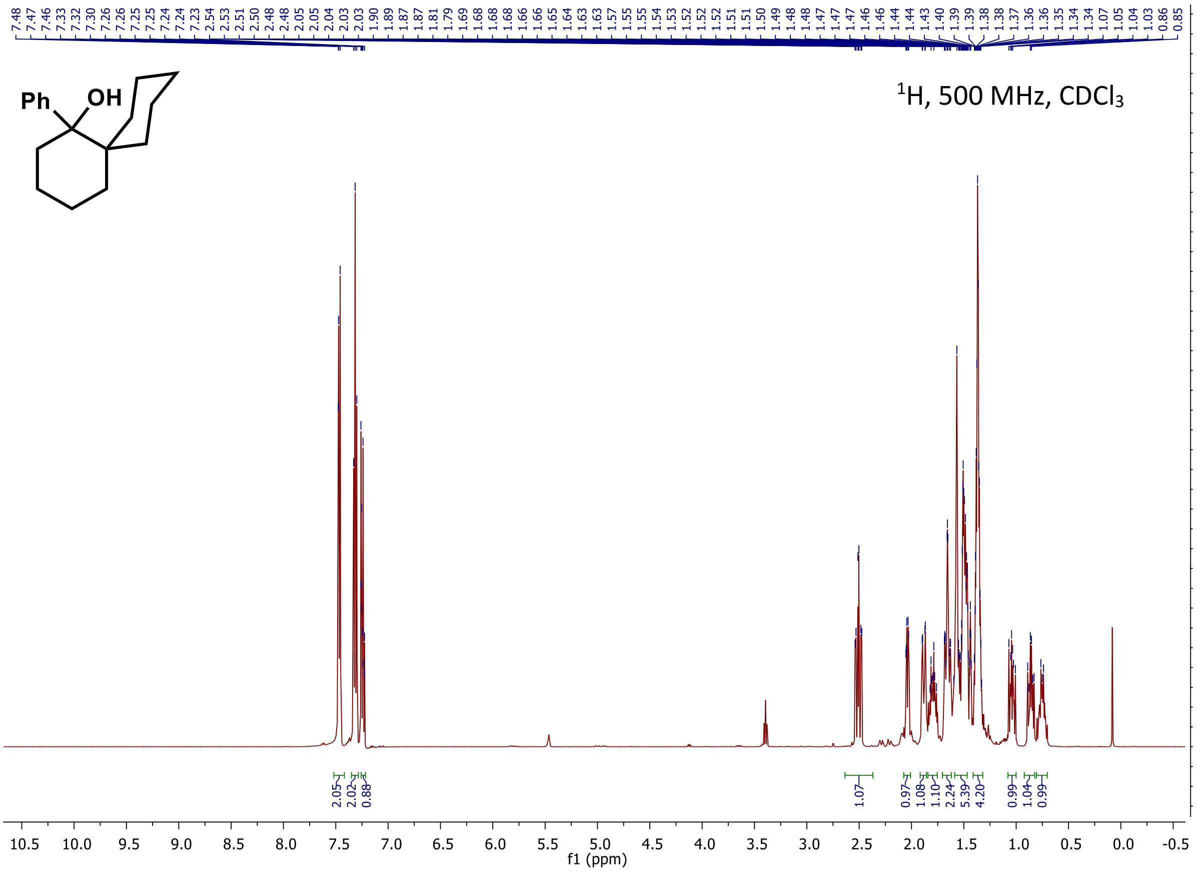
7.48
7.47
7.46
7.33
7.32
7.30
7.26
7.26
7.25
7.25
7.24
7.24
7.23
2.54
2.53
2.51
2.50
2.48
2.48
2.05
2.05
2.04
2.03
2.03
1.90
1.89
1.87
1.87
1.81
1.79
1.69
1.68
1.68
1.66
1.65
1.64
1.63
1.63
1.57
1.55
1.55
1.54
1.53
1.52
1.52
1.52
1.51
1.51
1.50
1.49
1.48
1.48
1.47
1.47
1.46
1.46
1.44
1.44
1.43
1.40
1.39
1.39
1.38
1.38
1.37
1.36
1.36
1.35
1.34
1.34
1.07
1.05
1.04
1.03
0.86
0.85
Ph
OH
^{1}H, 500 MHz, CDCl$_3$
2.05
2.02
0.88
1.07
0.97
1.08
1.10
2.24
5.39
4.20
0.99
1.04
0.99
10.5 10.0 9.5 9.0 8.5 8.0 7.5 7.0 6.5 6.0 5.5 5.0 4.5 4.0 3.5 3.0 2.5 2.0 1.5 1.0 0.5 0.0 -0.5
f1 (ppm)

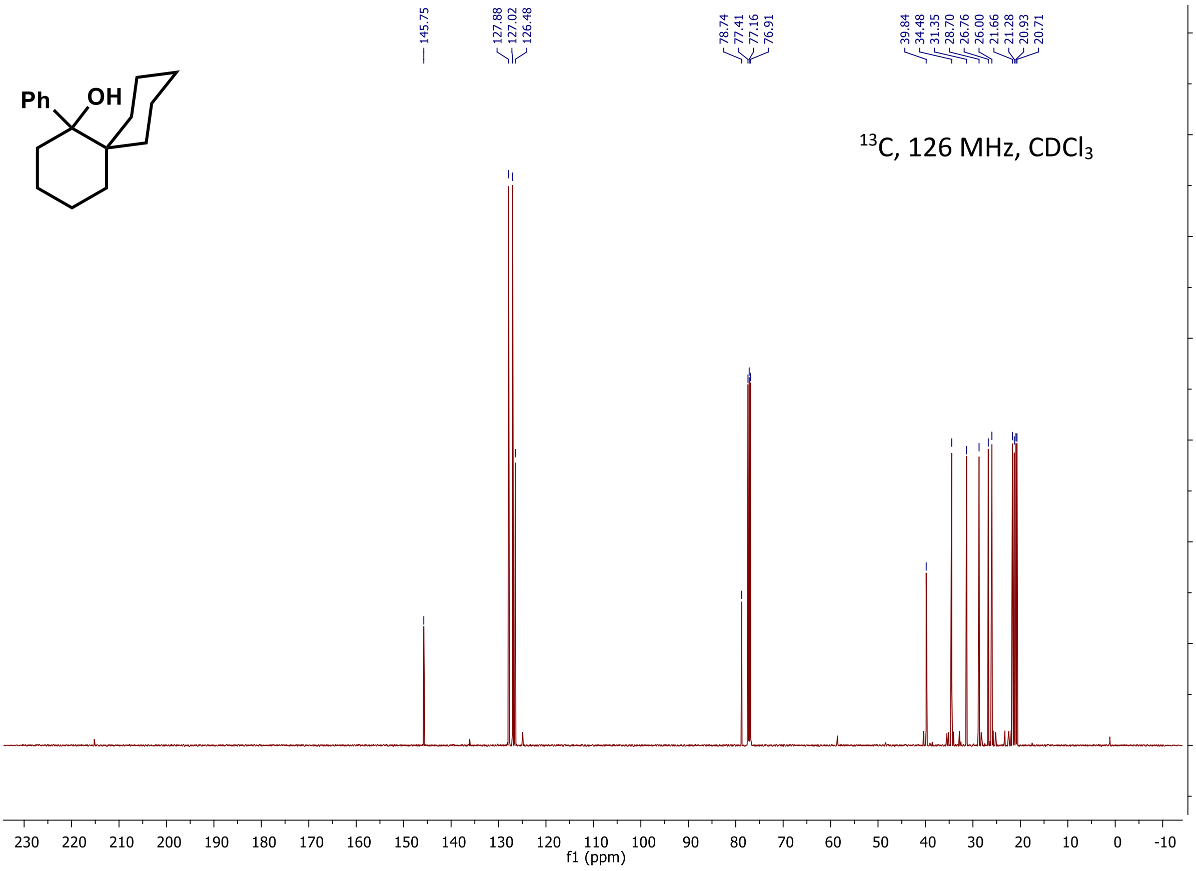
145.75
127.88
127.02
126.48
78.74
77.41
77.16
76.91
39.84
34.48
31.35
28.70
26.76
26.00
21.66
21.28
20.93
20.71
Ph
OH
^{13}C, 126 MHz, CDCl$_3$
230 220 210 200 190 180 170 160 150 140 130 120 110 100 90 80 70 60 50 40 30 20 10 0 -10
f1 (ppm)

(S26)

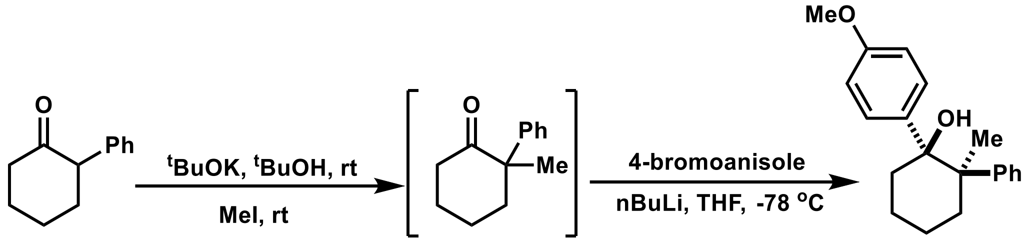

To a solution of 2-phenylcyclohexanone (0.87 g, 5 mmol, 1 equiv.) in tert-butanol (10 mL) was added potassium *tert*-butoxide (586 mg, 5.25 mmol, 1.05 equiv.). The mixture was stirred at room temperature for 1 h before methyl iodide (1.41 g, 10 mmol, 2 equiv.) was added and the reaction stirred for a further 3 h at room temperature. The reaction was quenched with water (15 mL) and extracted with EtOAc (30 mL). The organics were dried over $MgSO_4$, filtered, and concentrated *in vacuo*. The crude residue was passed through a silica plug yielding intermediate ketone as a colourless oil.

To a solution of 4-bromoanisole (1.40 g, 7.5 mmol, 1.5 equiv.) in THF (25 mL) at -78 °C was added *n*-butyllithium (3.0 mL, 7.5 mmol, 2.5 M in hexanes). The mixture was stirred at -78 °C for 1 h before the crude 2-methyl-2-phenylcyclohexan-1-one (0.94 g, 5 mmol, 1 equiv.) in THF (5 mL) was added dropwise and the mixture was allowed to stir up to room temperature overnight. The reaction was quenched with water (20 mL) and extracted with EtOAc (20 mL). The layers were separated and the aqueous extracted with EtOAc (2 x 20 mL). The organics were combined, dried over $MgSO_4$, filtered and concentrated *in vacuo* yielding crude product. The crude residue was purified by flash column chromatography (eluent = 5 to 10 % EtOAc in hexanes, silica gel) to afford product as a yellow oil (860 mg, 58% yield over 2 steps).[3]

R_f = 0.14 (eluent = 10% EtOAc in toluene); **v_{max} / cm^{-1}** (thin film) 3558, 2949, 2924, 1606, 1508, 1440, 1246, 1180, 1031, 825; **^{1}H NMR (500 MHz, $CDCl_3$)** δ_H: 1.37 (3H, s), 1.48-1.51 (1H, m), 1.65-1.69 (2H, m), 1.74-1.86 (2H, m), 1.95-2.04 (1H, m), 2.54-2.60 (1H, m), 2.78-2.84 (1H, m), 3.78 (3H, s), 6.66-6.71 (2H, m), 6.78-6.81 (2H, m), 7.05-7.08 (2H, m), 7.16-7.20 (3H, m); **^{13}C NMR (126 MHz, $CDCl_3$)** δ_C: 21.4, 21.5, 21.7, 33.4, 34.7, 45.6, 55.2, 112.0, 126.2, 127.0, 128.1, 128.7, 137.9, 144.7, 158.2; **HRMS (EI$^+$)** [$C_{20}H_{24}O_2$] requires $[M]^+$ 296.1776, found 296.1775 (- 0.3 ppm).

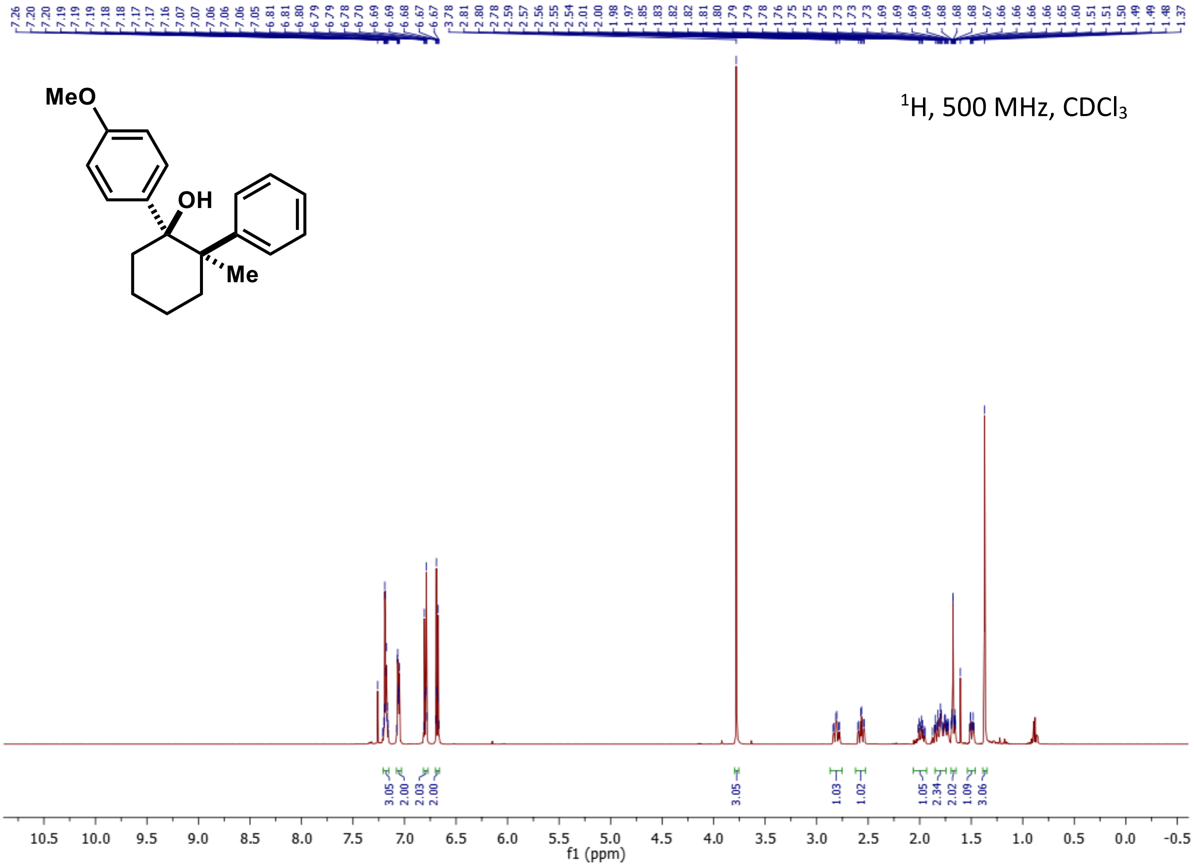
MeO
OH
Me
^{1}H, 500 MHz, $CDCl_3$
f1 (ppm)

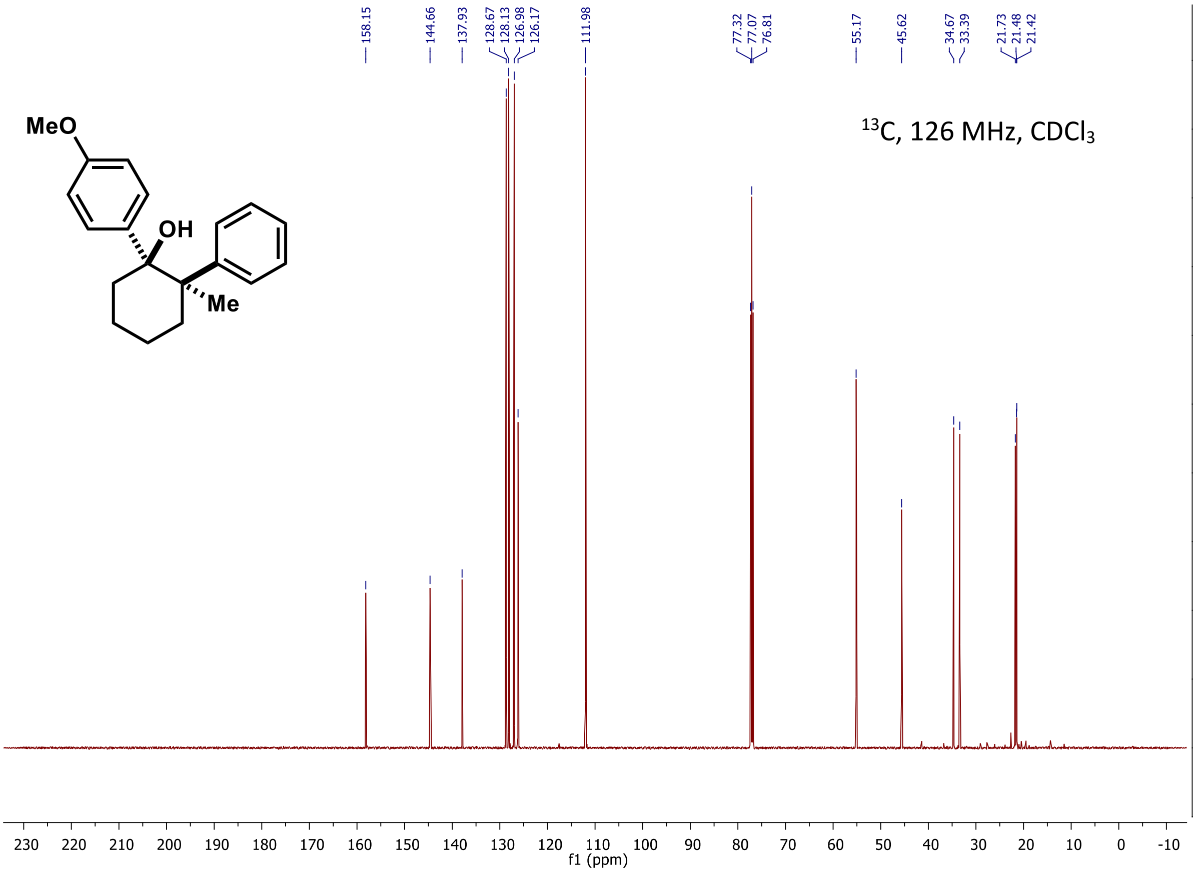
158.15
144.66
137.93
128.67
128.13
126.98
126.17
111.98
77.32
77.07
76.81
55.17
45.62
34.67
33.39
21.73
21.48
21.42
MeO
OH
Me
^{13}C, 126 MHz, $CDCl_3$
f1 (ppm)

(S27)

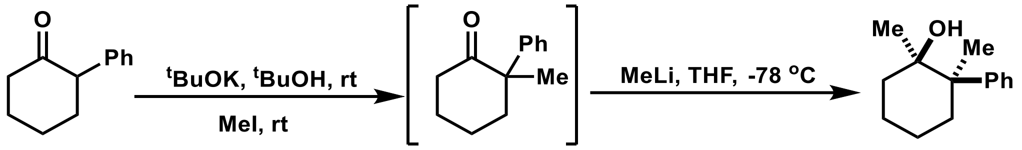

To a solution of 2-phenylcyclohexanone (1.30 g, 7.5 mmol, 1 equiv.) in tert-butanol (15 mL) was added potassium *tert*-butoxide (880 mg, 7.84 mmol, 1.05 equiv.). The mixture was stirred at room temperature for 1 h before methyl iodide (2.12 g, 15 mmol, 2 equiv.) was added and the reaction stirred for a further 3 h at room temperature. The reaction was quenched with water (15 mL) and extracted with EtOAc (30 mL). The organics were dried over $MgSO_4$, filtered and concentrated *in vacuo*. The crude residue was passed through a silica plug yielding intermediate ketone as a colourless oil (0.90 g). The oil (0.90 g, 4.80 mmol) was dissolved in dry THF (20 mL) and the mixture was cooled to -78 °C using an acetone/dry ice bath. Methyllithium (3.63 mL, 5.80 mmol, 1.2 equiv., 1.6 M solution in Et_2O) was added dropwise and the mixture was allowed to stir up to room temperature overnight. The reaction was quenched with water (20 mL) and extracted with EtOAc (20 mL). The layers were separated and the aqueous extracted with EtOAc (2 x 20 mL). The organics were combined, dried over $MgSO_4$, filtered and concentrated *in vacuo* yielding crude product. The crude residue was purified by flash column chromatography (eluent =5% EtOAc in hexanes, silica gel) to afford product as a white solid (680 mg, 44% yield over 2 steps).[3]

Mp.: 58-60 °C**;** $\mathbf{R_f}$ = 0.28 (eluent = 10% EtOAc in hexanes); $\mathbf{\nu_{max}}$ **/ cm^{-1}** (thin film) 3350, 3032, 1497, 1440; **^{1}H NMR (500 MHz, CDCl$_3$)** δ_H: 1.00 (3H, s), 1.12 (1H, s (br)), 1.29-1.34 (1H, m), 1.46 (3H, s), 1.54-1.63 (2H, m), 1.63-1.71 (2H, m), 1.73-1.84 (2H, m), 2.59-2.67 (1H, m), 7.22-7.26 (1H, m), 7.32-7.38 (2H, m), 7.54-7.60 (2H, m); **^{13}C NMR (126 MHz, CDCl$_3$)** δ_C: 21.2, 21.6, 21.7, 26.6, 33.0, 35.6, 45.4, 73.1, 126.4, 127.9, 128.5, 145.5; **HRMS (CI$^+$)** [$C_{14}H_{20}O$] requires [M-H$^+$] 203.1436, found 203.1431 (- 2.5 ppm).

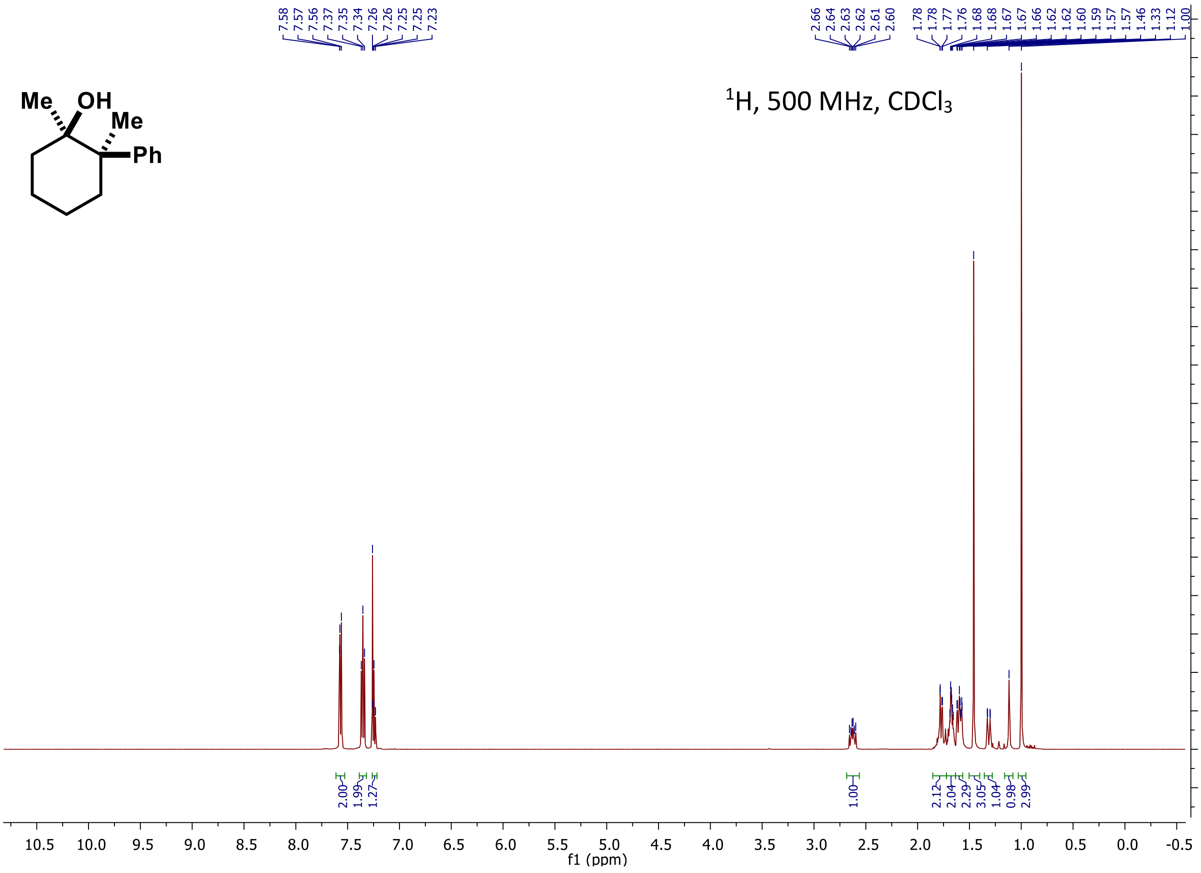
Me OH Me Ph
^{1}H, 500 MHz, $CDCl_3$
7.58 7.57 7.56 7.37 7.35 7.34 7.26 7.26 7.25 7.25 7.23
2.66 2.64 2.63 2.62 2.61 2.60
1.78 1.78 1.77 1.76 1.68 1.68 1.67 1.67 1.66 1.62 1.62 1.60 1.59 1.57 1.57 1.46 1.33 1.12 1.00
2.00 1.99 1.27 1.00 2.12 2.04 2.29 3.05 1.04 0.98 2.99
f1 (ppm)

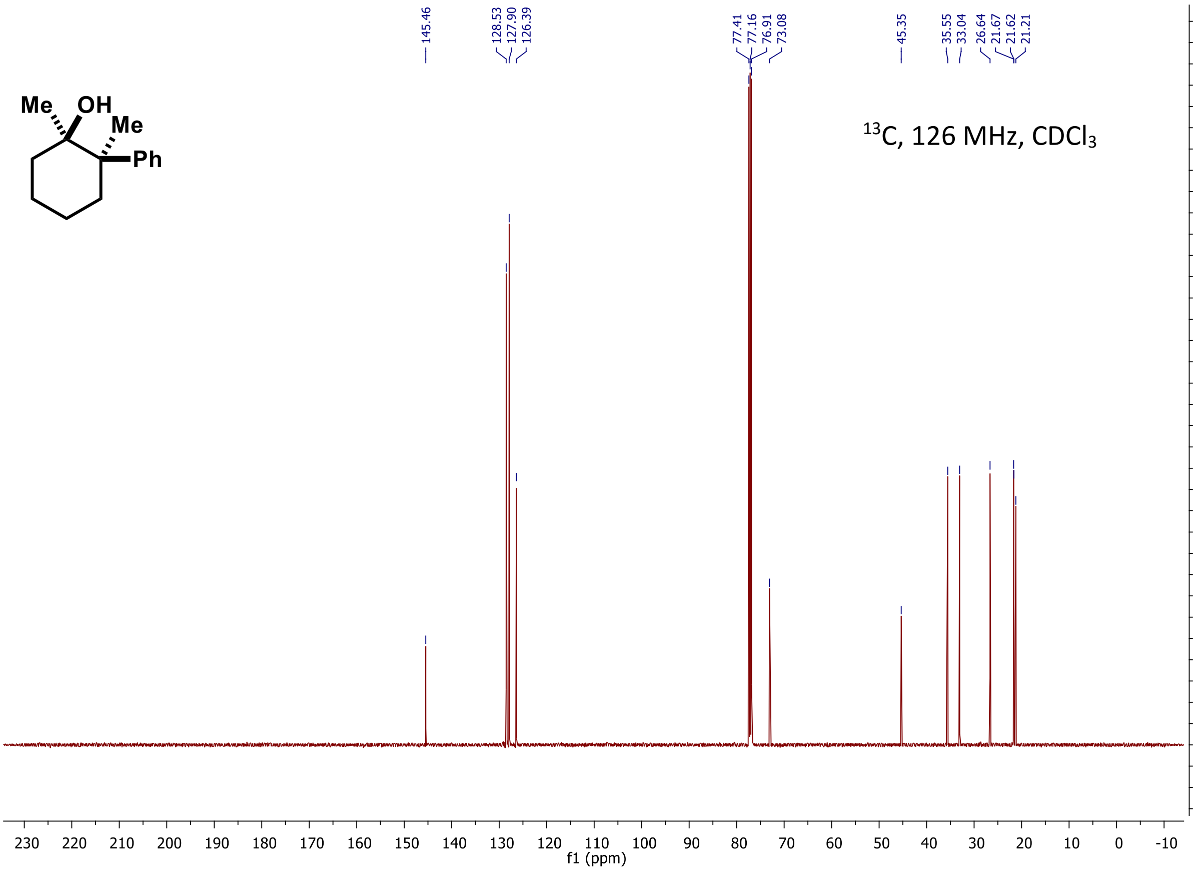
Me OH Me Ph
^{13}C, 126 MHz, $CDCl_3$
145.46
128.53 127.90 126.39
77.41 77.16 76.91 73.08
45.35
35.55 33.04 26.64 21.67 21.62 21.21
f1 (ppm)

(S28)

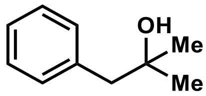

Sourced commercially.

(S29A)

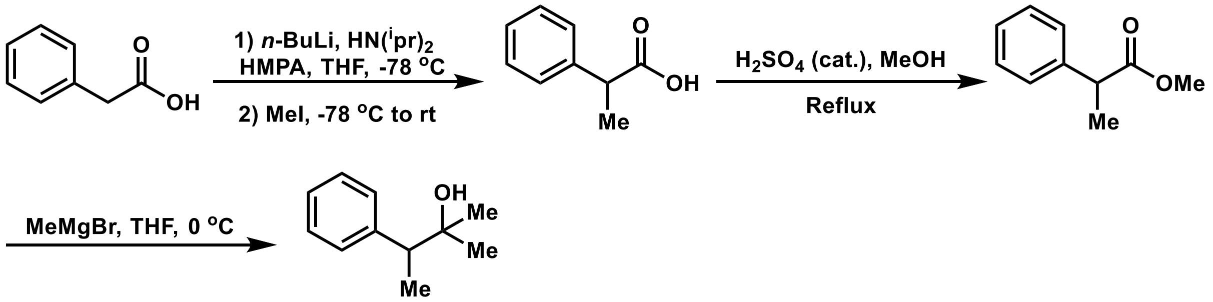

To a flame dried flask was added diisopropylamine (9.32 g, 92 mmol, 2.5 equiv.) and THF (100 mL) and the subsequent solution was cooled to -78 °C. *n*-butyllithium (35 mL, 87 mmol, 2.4 equiv., 2.5 M in hexanes) was added slowly and the mixture was stirred at -78 °C for 1 h. Phenylacetic acid (5.00 g, 37 mmol, 1 equiv.) in HMPA (10 mL) was added slowly and the mixture was stirred from -78 °C to 0 °C for 1 h before cooling back to -78 °C. Iodomethane (3.4 mL, 55 mmol, 1.5 equiv.) was added and the mixture was allowed to stir up to room temperature overnight. The reaction mixture was quenched with 1 M HCl (50 mL), extracted with ethyl acetate (2 x 100 mL), organics combined, dried over $MgSO_4$, filtered and concentrated *in vacuo* yielding crude carboxylic acid. The crude residue was dissolved in MeOH (100 mL), concentrated H_2SO_4 (5 drops) was added and the mixture heated to reflux for 5 h. The solvent was removed *in vacuo*, residue dissolved in EtOAc (100 mL), washed with a saturated solution of sodium bicarbonate (25 mL), organics dried over $MgSO_4$, filtered and concentrated *in vacuo* yielding crude methyl ester product. The crude residue was purified by flash column chromatography (eluent = 5 to 10% EtOAc in hexanes, silica gel) to pure ester (4.28 g, 70% yield).

To a flame dried flask was added methyl 2-phenylpropanoate (3.28 g, 20 mmol, 1 equiv.) and THF (40 mL) and the solution was cooled to 0 °C using an icebath. Methylmagnesium bromide (20 mL, 60 mmol, 3 equiv., 3M in Et_2O) was added dropwise and the mixture was allowed to stir up to room temperature overnight. The mixture was quenched with water (50 mL), extracted with EtOAc (2 x 50 mL), organics combined, dried over $MgSO_4$, filtered and concentrated *in vacuo* yielding pure product as a white solid (2.60 g, 79% yield).

Data consistent with the literature.[4]

Mp.: 49-52 °C; **R$_f$** = 0.29 (eluent = 20% EtOAc in hexanes); **ν_{max} / cm^{-1}** (thin film) 3346, 2985, 1490, 1450, 1360, 1149; **^{1}H NMR (500 MHz, CDCl$_3$)** δ_H: 1.18 (6H, s), 1.26-1.32 (1H, m, br), 1.34 (3H, d, *J* 7.2 Hz), 2.80 (1H, q, *J* 7.2 Hz), 7.21-7.27 (3H, m), 7.28-7.33 (2H, m); **^{13}C NMR (126 MHz, CDCl$_3$)** δ_C: 15.9, 27.1, 28.2, 50.5, 72.8, 126.7, 128.2, 129.1, 143.5.

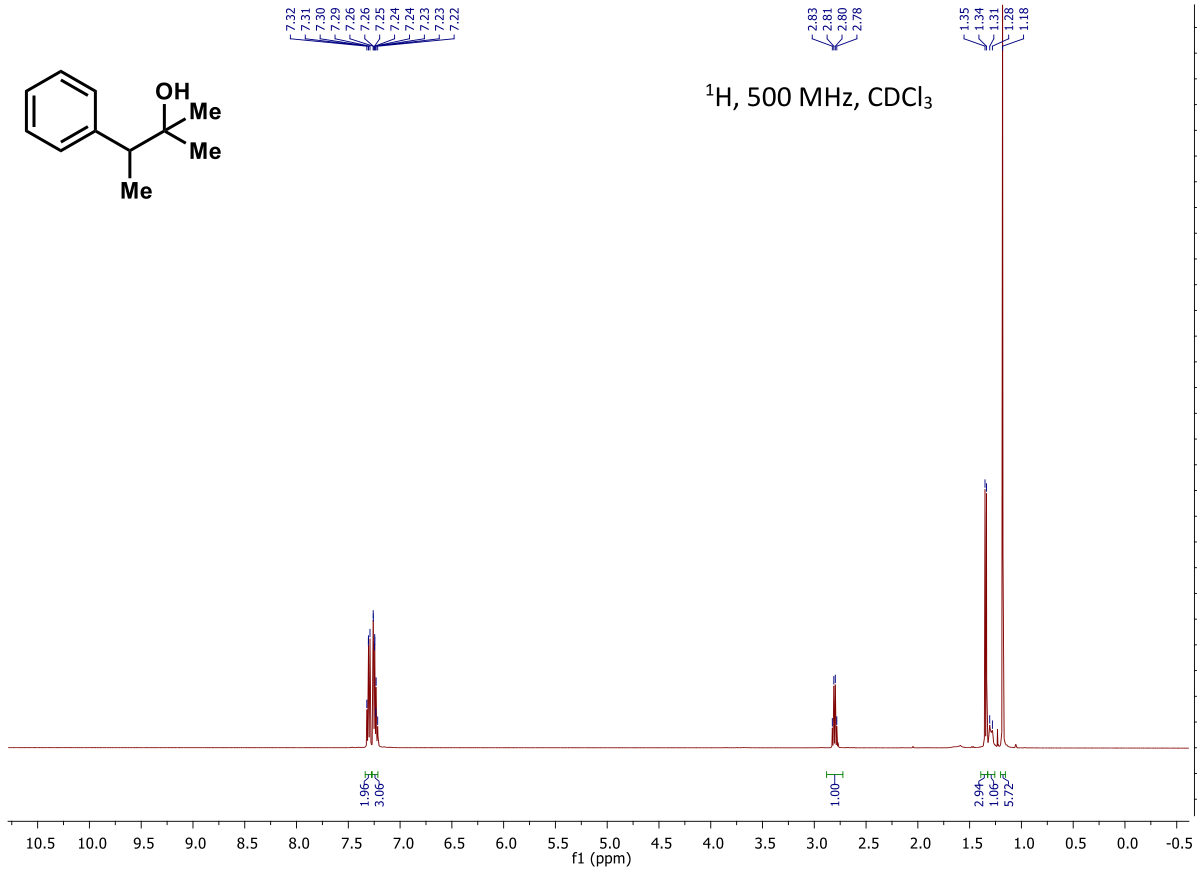

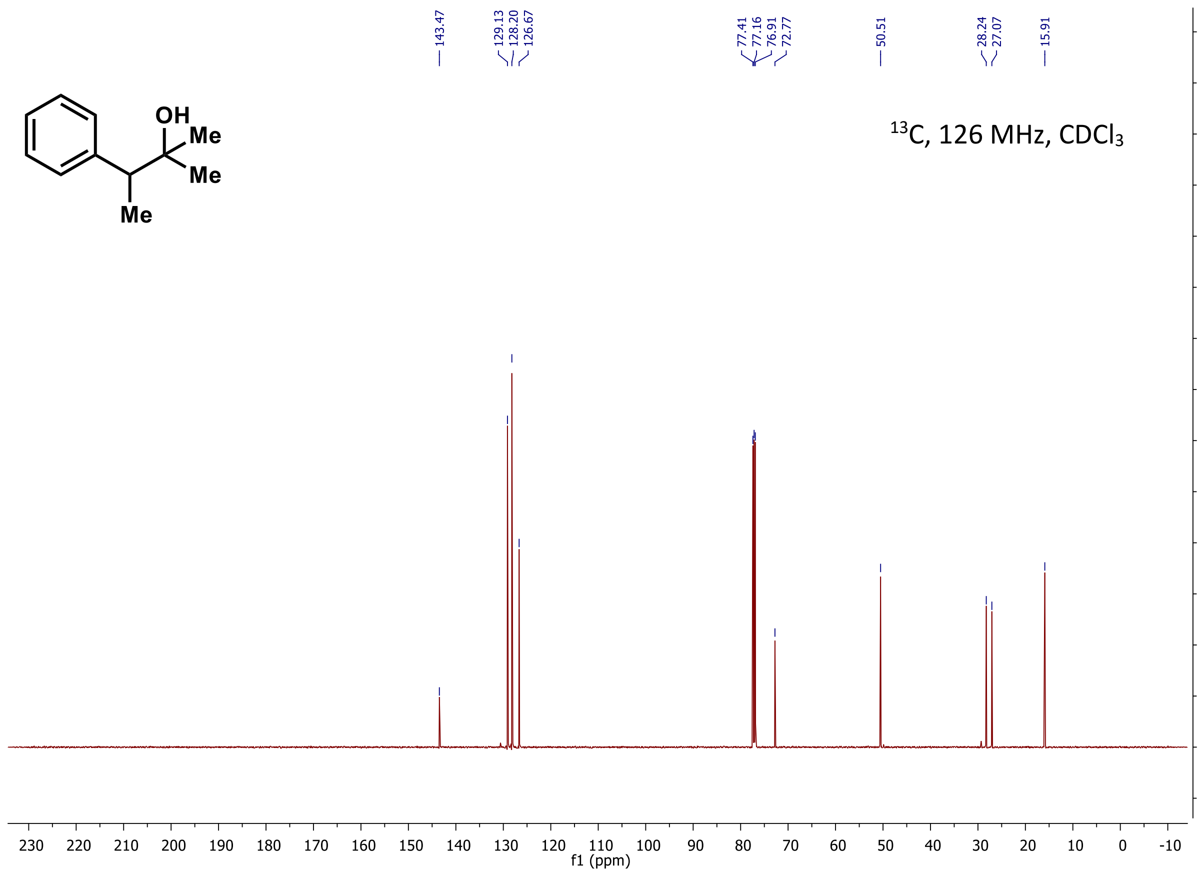

(S29B)

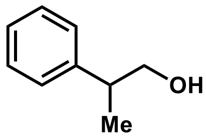

Sourced commercially.

Characterisation of Products

Electrochemical General Procedure X:

To an oven-dried 10 mL ElectraSyn vial equipped with a magnetic stirrer bar, was added substrate cycloalkanol (0.30 mmol) and *n*-Bu_4NPF_6 (232 mg, 0.60 mmol). The threaded glass of the vial was wrapped with PTFE tape and connected to the ElectraSyn cap, which was fitted with a graphite anode and a graphite cathode*. The vial was purged with N_2 gas via evacuate-refill cycles (× 3). Dichloromethane (4.5 mL) was added, followed by MeOH (1.5 mL) and the mixture was stirred to facilitate dissolution. The mixture was then purged via bubbling with N_2 gas for 3 minutes. The vial was then connected to an ElectraSyn. Electrolysis at 10 mA was conducted until 3.0 *F* of charge had been passed, under N_2 with continuous stirring. After electrolysis was complete mesitylene internal standard (42 µL, 0.30 mmol) was added to the reaction mixture followed by sampling for crude ^{1}H NMR analysis. The mixture was then concentrated *in vacuo* and the crude residue was purified by flash column chromatography on silica gel.

General Procedure Y: *Pt Cathode

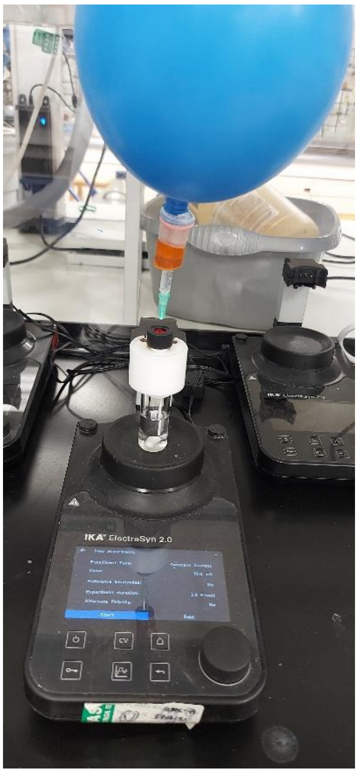

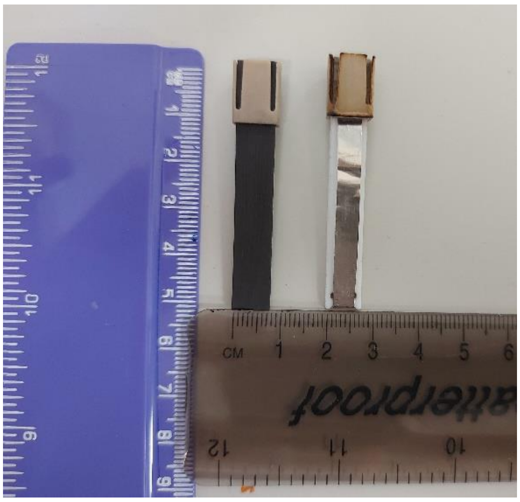

Left: IKA ElectraSyn 2.0 set up; Right: Exemplar graphite anode and platinum cathode

(2)

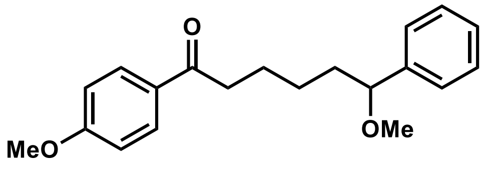

Prepared according to the General Procedure X using **1** (85.0 mg, 0.30 mmol, 1 equiv.), *n*-Bu_4NPF_6 (232 mg, 0.60 mmol, 2 equiv.), dichloromethane (4.5 mL) and MeOH (1.5 mL). The yield was determined by crude ^{1}H NMR using 1,3,5-trimethylbenzene (42 µL, 1 equiv.) as an internal standard: 67%. Purification by flash column chromatography (eluent = 5 to 15% EtOAc in hexanes, silica gel) to afford product as an off white solid (58.6 mg, 61% yield).

Mp.: 59-60 °C; $\mathbf{R_f}$ = 0.35 (eluent = 20% EtOAc in hexanes); $\mathbf{\nu_{max}}$ **/ cm^{-1}** (thin film) 2935, 1668, 1603, 1423, 1361, 1252, 1184, 1091; **^{1}H NMR (500 MHz, $CDCl_3$)** δ_H: 1.26-1.38 (1H, m), 1.39-1.53 (1H, m), 1.59-1.75 (3H, m), 1.77-1.89 (1H, m), 2.86 (2H, app. t, *J* 8.0 Hz), 3.17 (3H, m), 3.83 (3H, s), 4.08 (1H, dd, *J* 7.1, 6.1 Hz), 6.89 (2H, d, *J* 8.6 Hz), 7.21-7.29 (3H, m), 7.29-7.35 (2H, m), 7.89 (2H, d, *J* 8.6 Hz); **^{13}C NMR (126 MHz, $CDCl_3$)** δ_C: 24.5, 25.7, 38.1, 38.3, 55.5, 56.7, 83.9, 113.8, 126.8, 127.6, 128.5, 130.2, 130.4, 142.4, 163.4, 199.0; **HRMS (ES$^+$)** [$C_{20}H_{24}O_3$] requires [M+Na]$^+$ 335.1623, found 335.1631 (+2.4 ppm).

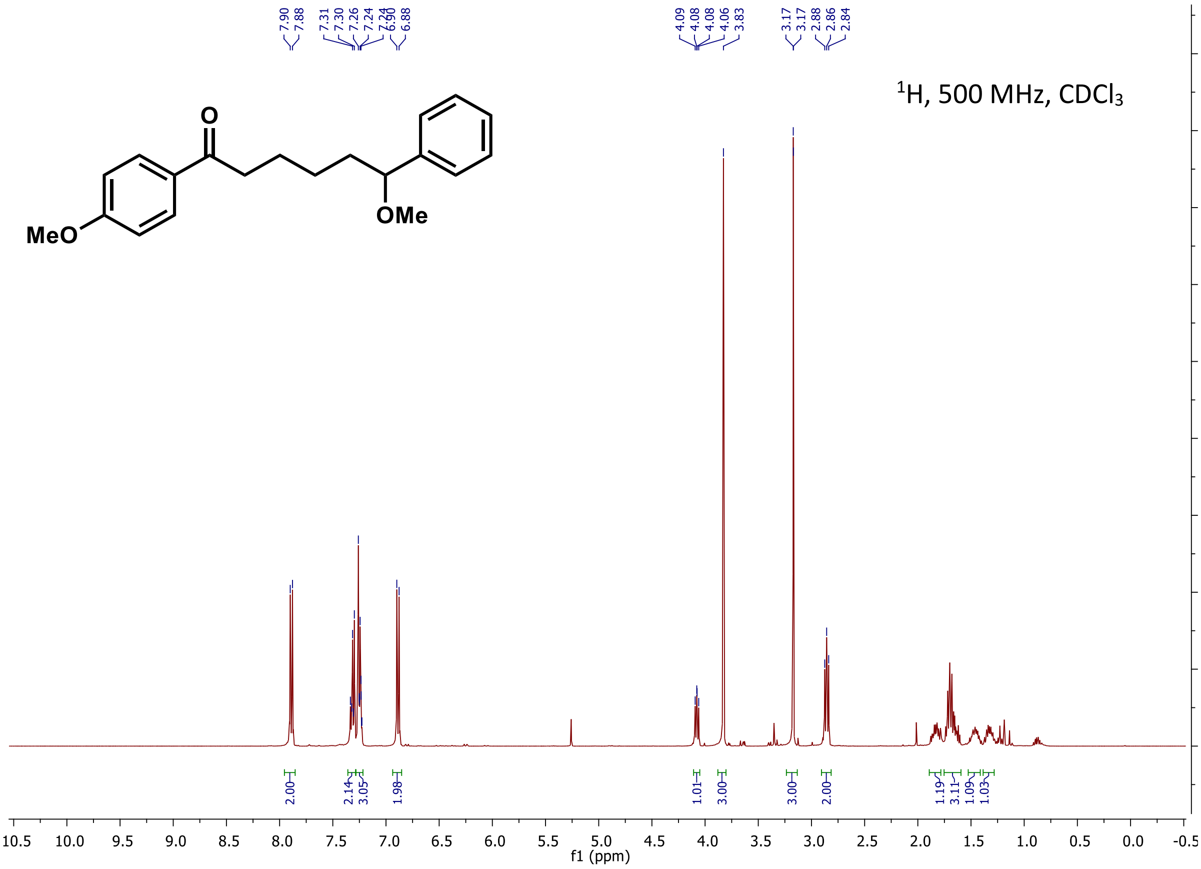

MeO
O
OMe
^{1}H, 500 MHz, CDCl$_3$
f1 (ppm)

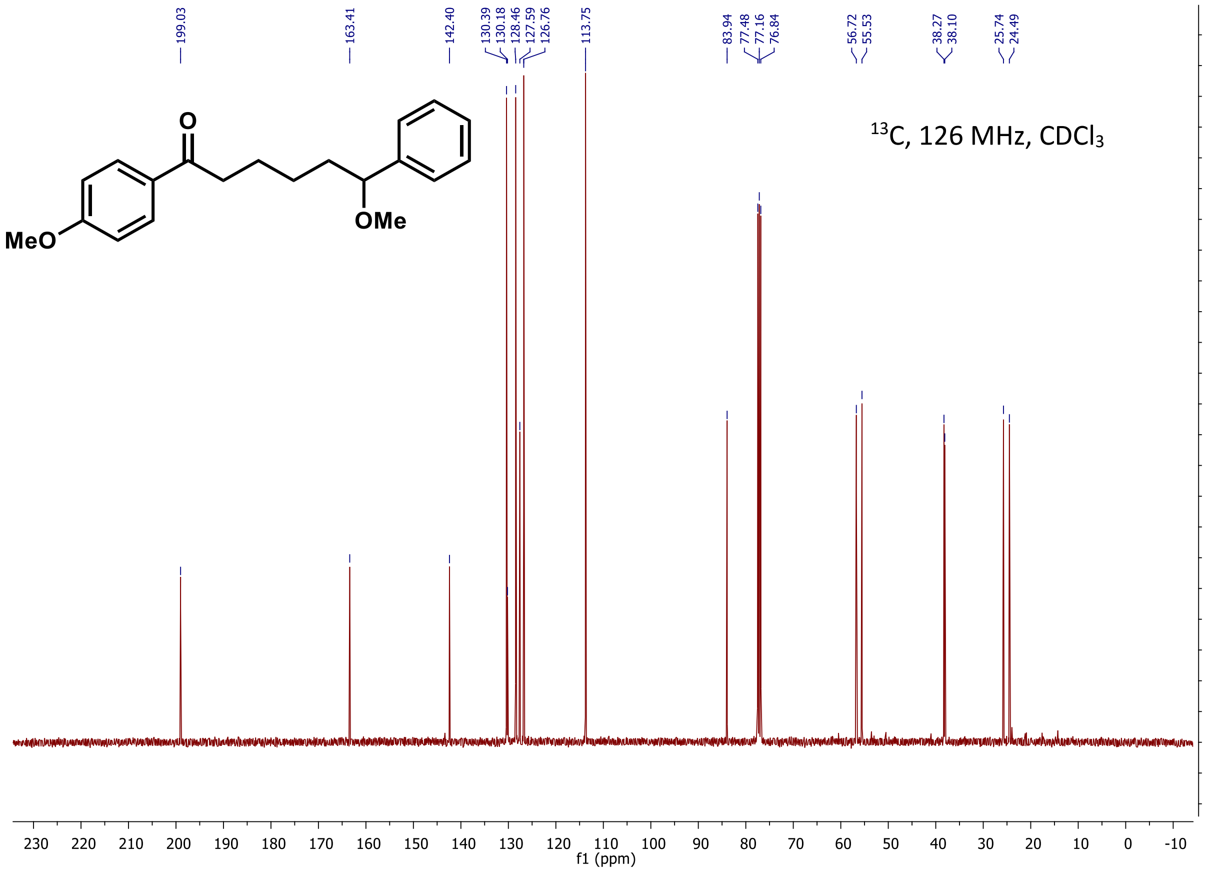

MeO
O
OMe
^{13}C, 126 MHz, CDCl$_3$
f1 (ppm)

(3)

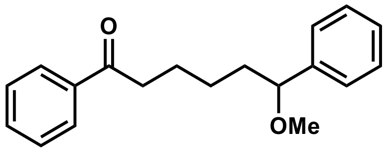

Prepared according to the General Procedure X using **S3** (75.6 mg, 0.30 mmol, 1 equiv.), *n*-Bu_4NPF_6 (232 mg, 0.60 mmol, 2 equiv.), dichloromethane (4.5 mL) and MeOH (1.5 mL). The yield was determined by crude ^{1}H NMR using 1,3,5-trimethylbenzene (42 µL, 1 equiv.) as an internal standard: 45%. Purification by flash column chromatography (eluent = 5 to 15% EtOAc in hexanes, silica gel) to afford product as a white solid (36.4 mg, 43% yield).

Mp.: 49-51 °C; **R_f** = 0.47 (eluent = 20% EtOAc in hexanes); **v_{max} / cm^{-1}** (thin film) 2940, 1680, 1595, 1447, 1364, 1248, 1209, 1188, 1067; **^{1}H NMR (500 MHz, $CDCl_3$)** δ_H: 1.28-1.40 (1H, m), 1.42-1.55 (1H, m), 1.60-1.76 (3H, m), 1.79-1.91 (1H, m), 2.92 (2H, t, *J* 8.0 Hz), 3.18 (3H, s), 4.09 (1H, dd, *J* 7.2, 6.1 Hz), 7.22-7.29 (3H, m), 7.29-7.35 (2H, m), 7.40-7.45 (2H, m), 7.52 (1H, app t, *J* 7.4 Hz), 7.91 (2H, app d, *J* 7.3 Hz); **^{13}C NMR (126 MHz, $CDCl_3$)** δ_C: 24.3, 25.7, 38.1, 38.6, 56.8, 84.0, 126.8, 127.6, 128.2, 128.5, 128.7, 133.0, 137.1, 142.4, 200.4; **HRMS (ES$^+$)** [$C_{19}H_{22}O_2$] requires [M+Na]$^+$ 305.1517, found 305.1519 (+ 0.7 ppm).

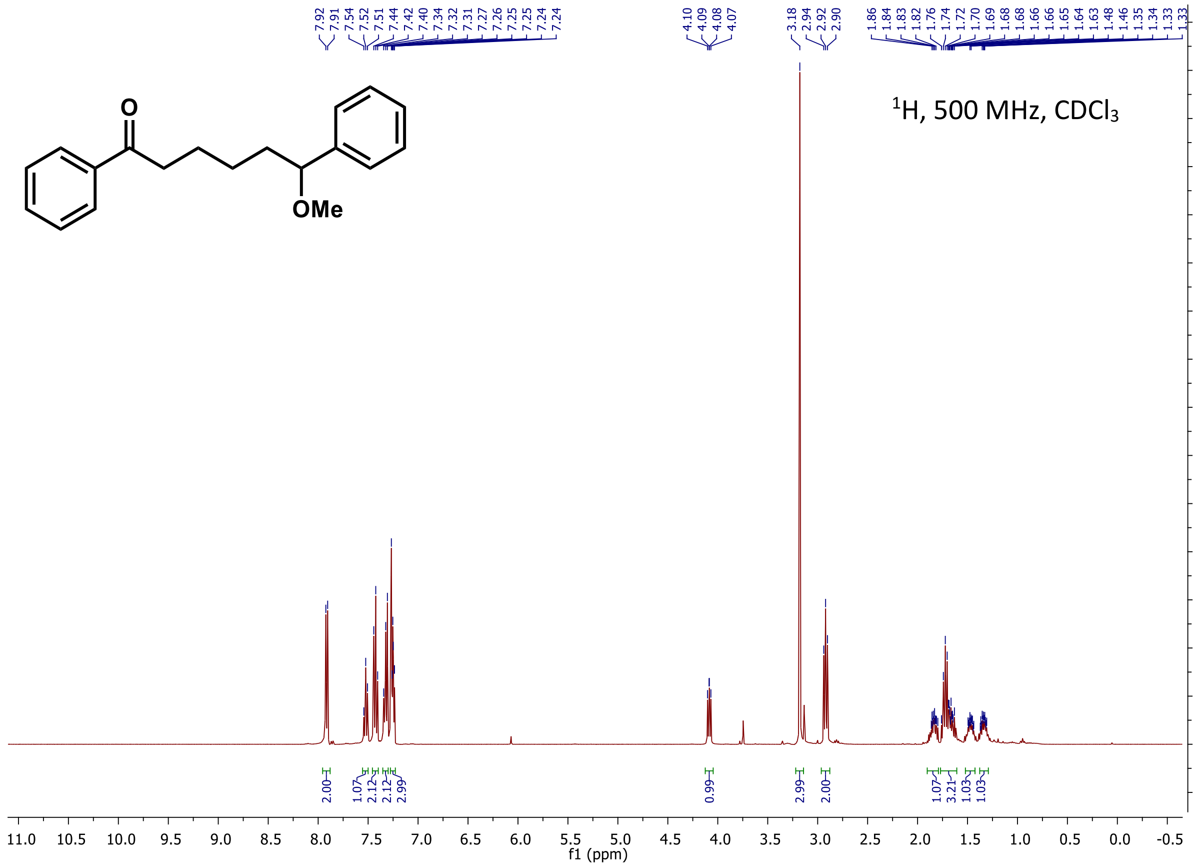

OMe
1H, 500 MHz, CDCl3
f1 (ppm)

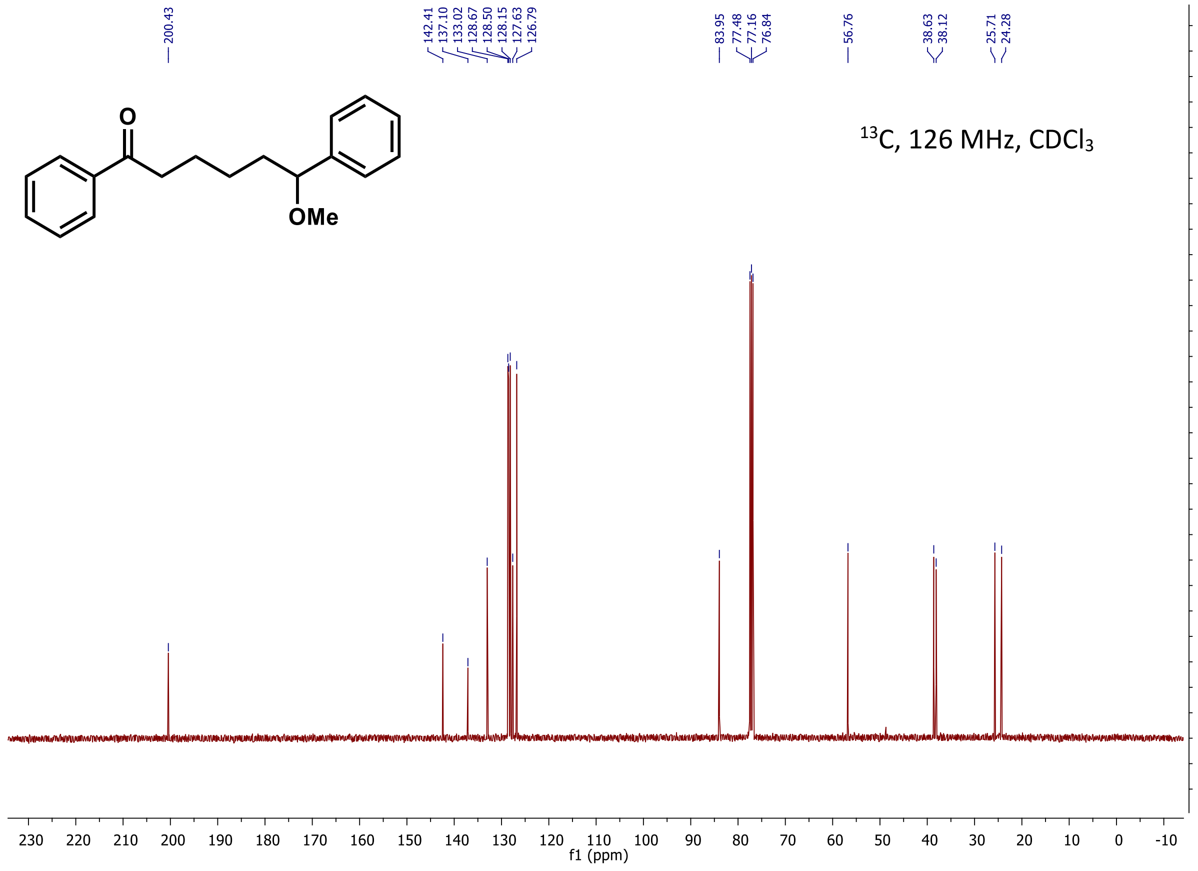

OMe
13C, 126 MHz, CDCl3
f1 (ppm)

(4)

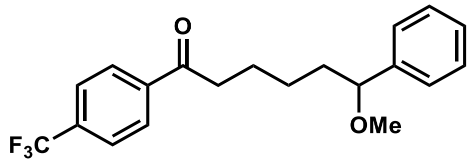

Prepared according to the General Procedure Y using **S4** (96.0 mg, 0.30 mmol, 1 equiv.), *n*-Bu_4NPF_6 (232 mg, 0.60 mmol, 2 equiv.), dichloromethane (4.5 mL) and MeOH (1.5 mL). The yield was determined by crude ^{1}H NMR using 1,3,5-trimethylbenzene (42 µL, 1 equiv.) as an internal standard: 55%. Purification by flash column chromatography (eluent = 5% EtOAc in hexanes, silica gel) to afford product as a yellow oil (52.4 mg, 50% yield).

R_f = 0.43 (eluent = 20% EtOAc in hexanes); **ν_{max} / cm^{-1}** (thin film) 1692, 1409, 1323, 1169, 1128, 1107, 1064; **^{1}H NMR (500 MHz, $CDCl_3$)** δ_H: 1.31-1.41 (1H, m), 1.45-1.57 (1H, m), 1.63-1.71 (1H, m), 1.72-1.79 (2H, m), 1.82-1.92 (1H, m), 2.96 (2H, dd, *J* 8.0, 6.8 Hz), 3.20 (3H, s), 4.11 (1H, dd, *J* 7.4, 5.8 Hz), 7.24-7.30 (3H, m), 7.32-7.38 (2H, m), 7.71 (2H, d, *J* 8.1 Hz), 8.03 (2H, d, *J* 8.1 Hz); **^{13}C NMR (126 MHz, $CDCl_3$)** δ_C: 24.1, 25.6, 38.1, 38.9, 56.8, 83.9, 123.8 (q, *J* 273 Hz), 125.8 (q, *J* 3.8 Hz), 126.8, 127.7, 128.5, 128.5, 134.4 (q, *J* 32.8 Hz), 139.8, 142.4, 199.3; **^{19}F NMR (471 MHz, $CDCl_3$)** δ_F: -63.1; **HRMS (ES^+)** [$C_{20}H_{21}O_2F_3$] requires $[M+Na]^+$ 373.1391, found 373.1388 (- 0.8 ppm).

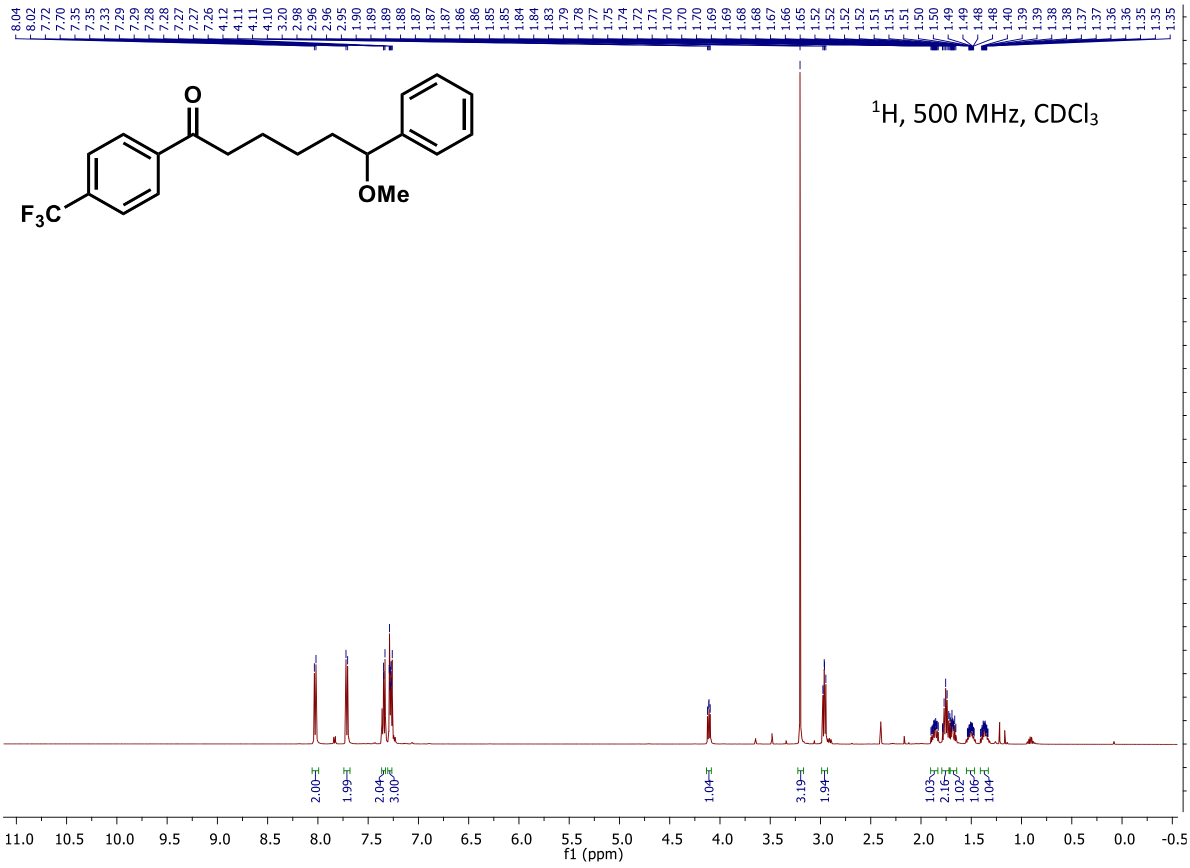

1H, 500 MHz, CDCl3
OMe
F3C
O
f1 (ppm)

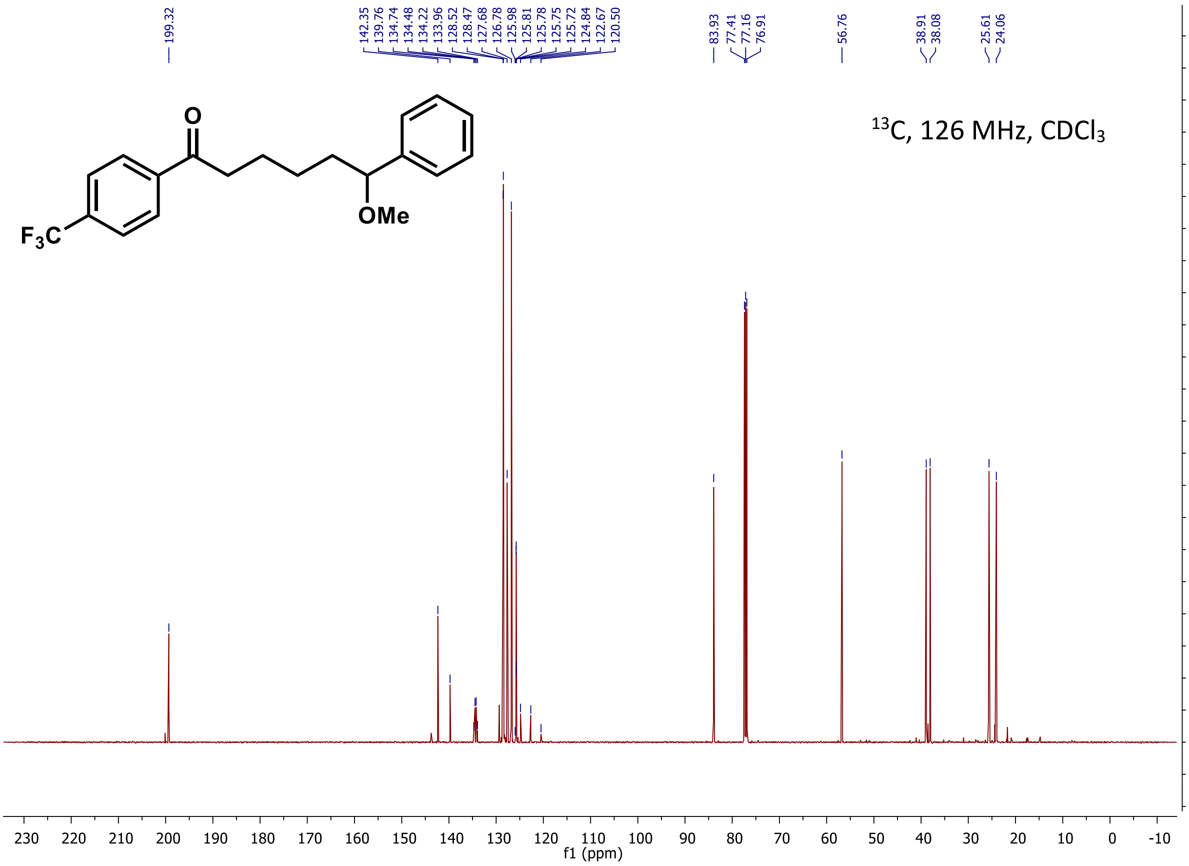

13C, 126 MHz, CDCl3
OMe
F3C
O
f1 (ppm)

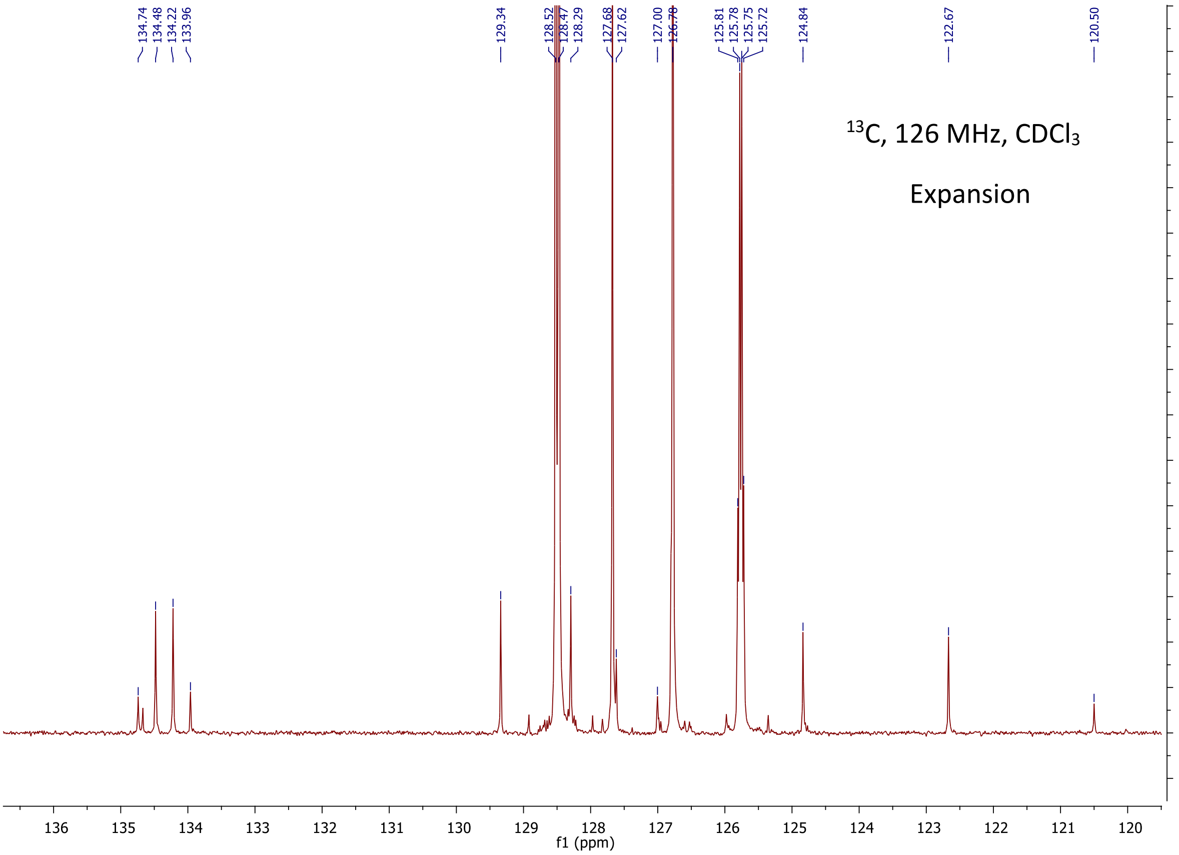

134.74
134.48
134.22
133.96
129.34
128.52
128.47
128.29
127.68
127.62
127.00
126.78
125.81
125.78
125.75
125.72
124.84
122.67
120.50
^{13}C, 126 MHz, CDCl$_3$
Expansion
136 135 134 133 132 131 130 129 128 127 126 125 124 123 122 121 120
f1 (ppm)

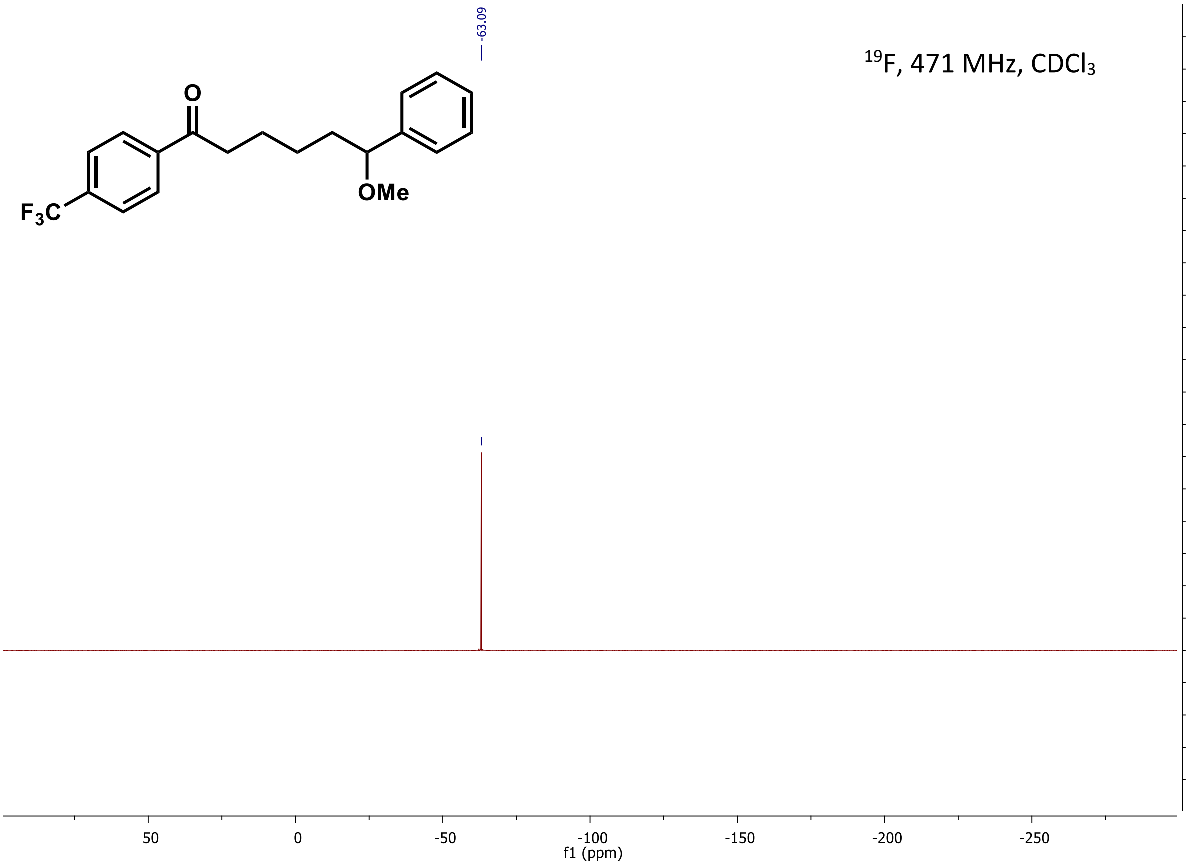

-63.09
^{19}F, 471 MHz, CDCl$_3$
O
F$_3$C
OMe
50 0 -50 -100 -150 -200 -250
f1 (ppm)

(5)

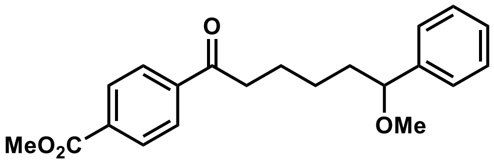

Prepared according to the General Procedure Y using **S5** (93.0 mg, 0.30 mmol, 1 equiv.), *n*-Bu_4NPF_6 6 (232 mg, 0.60 mmol, 2 equiv.), dichloromethane (4.5 mL) and MeOH (1.5 mL). The yield was determined by crude ^{1}H NMR using 1,3,5-trimethylbenzene (42 µL, 1 equiv.) as an internal standard: 39%. Purification by flash column chromatography (eluent = 10 to 20% EtOAc in hexanes, silica gel) to afford product as a yellow solid (39.7 mg, 39% yield).

R_f = 0.29 (eluent = 20% EtOAc in hexanes); **ν_{max} / cm^{-1}** (thin film) 2938, 1722, 1686, 1435, 1275, 1105 ; **^{1}H NMR (400 MHz, $CDCl_3$)** δ_H: 1.25-1.35 (1H, m), 1.40-1.50 (1H, m), 1.60-1.70 (3H, m), 1.75-1.85 (1H, m), 2.87-2.92 (2H, m), 3.13 (3H, s), 3.88 (3H, s), 4.04 (1H, dd, *J* 7.2, 6.0 Hz), 7.16-7.30 (5H, m), 7.91 (2H, d, *J* 8.3 Hz), 8.04 (2H, d, *J* 8.3 Hz); **^{13}C NMR (101 MHz, $CDCl_3$)** δ_C: 24.1, 25.6, 38.1, 39.0, 52.6, 56.8, 83.9, 126.8, 127.7, 128.0, 128.5, 129.9, 133.8, 140.3, 142.4, 166.4, 199.8; **HRMS (ES^+)** [$C_{21}H_{24}O_4$] requires $[M-CH_3]^+$ 325.1440, found 325.1429 (- 3.4 ppm).

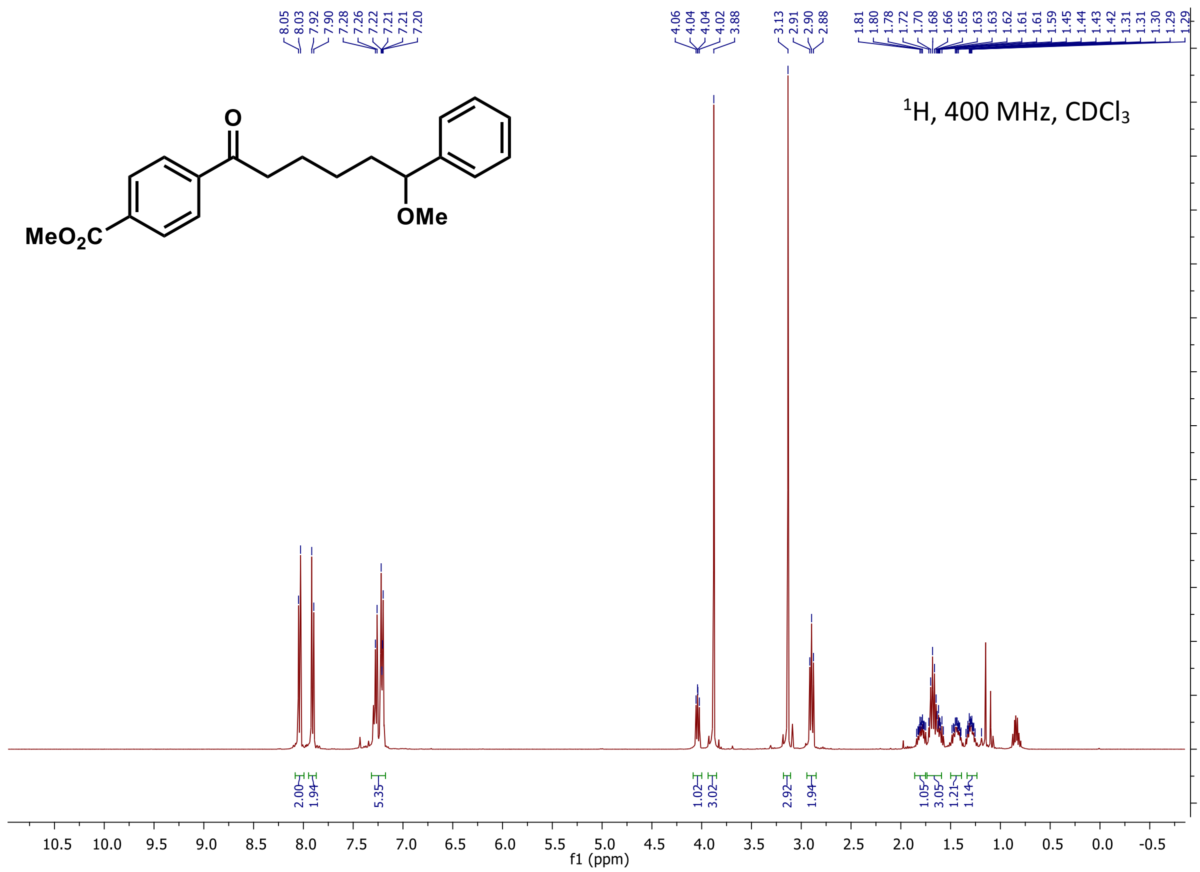

MeO2C
O
OMe
1H, 400 MHz, CDCl3
f1 (ppm)

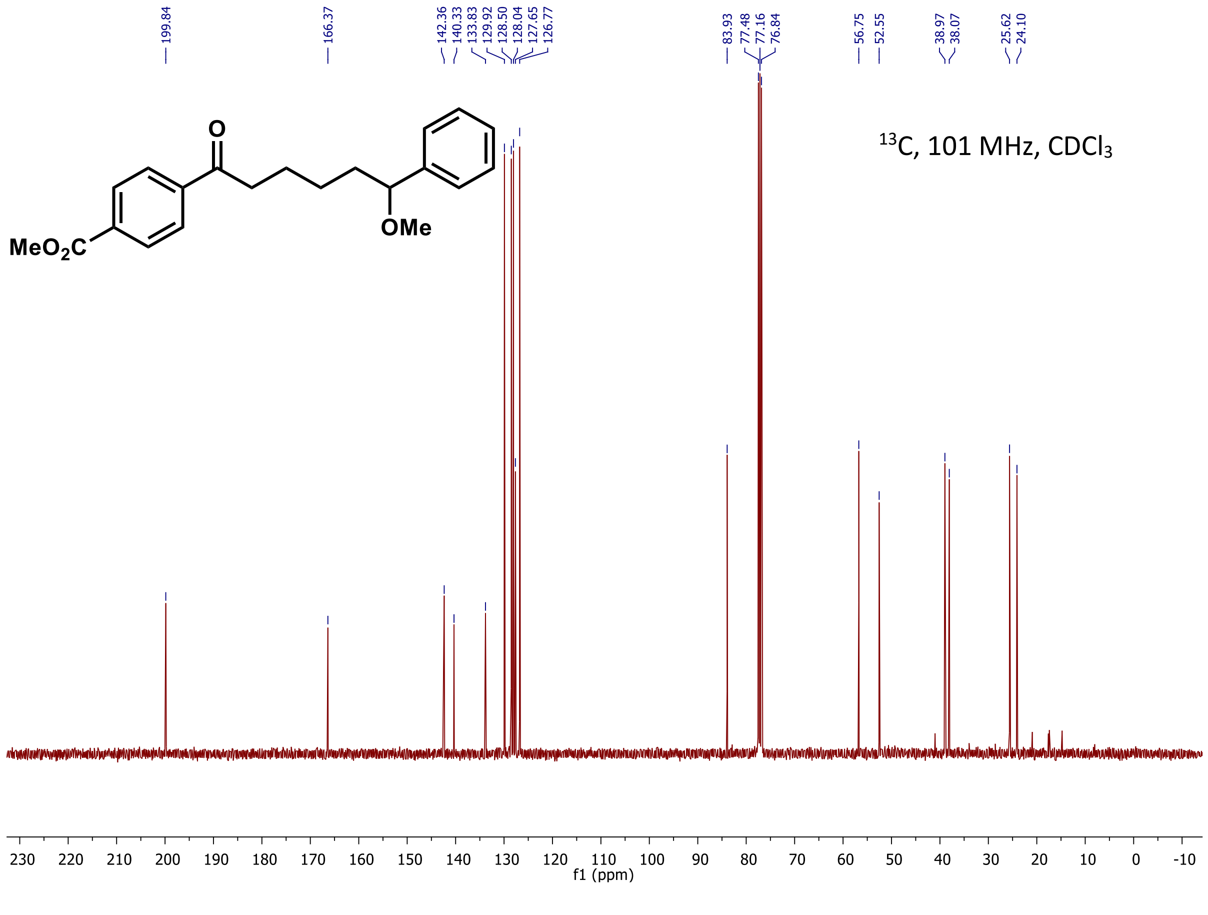

MeO2C
O
OMe
13C, 101 MHz, CDCl3
f1 (ppm)

(6)

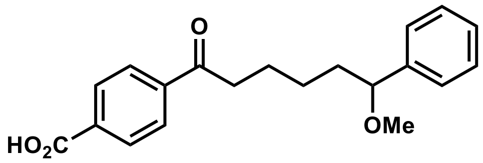

Prepared according to the General Procedure Y using **S6** (89.0 mg, 0.30 mmol, 1 equiv.), *n*-Bu_4NPF_6 (232 mg, 0.60 mmol, 2 equiv.), dichloromethane (4.5 mL) and MeOH (1.5 mL). The yield was determined by crude ^{1}H NMR using 1,3,5-trimethylbenzene (42 µL, 1 equiv.) as an internal standard: 53%. Purification by flash column chromatography (eluent = 50 to 60% EtOAc in hexanes, silica gel) to afford product as a yellow solid (46.8 mg, 48% yield).

Mp.: 106-108 °C; $\mathbf{R_f}$ = 0.17 (eluent = 60% EtOAc in hexanes); $\mathbf{\nu_{max}}$ **/ cm^{-1}** (thin film) 2933, 1691, 1687, 1408, 1285, 1105 ; **^{1}H NMR (400 MHz, $CDCl_3$)** δ_H: 1.31-1.44 (1H, m), 1.45-1.60 (1H, m), 1.64-1.81 (3H, m), 1.84-1.92 (1H, m), 2.92-3.03 (2H, m), 3.21 (3H, s), 4.12 (1H, dd, *J* 7.3, 6.0 Hz), 7.27-7.39 (5H, m), 8.01 (2H, d, *J* 8.5 Hz), 8.18 (2H, d, *J* 8.5 Hz); **^{13}C NMR (126 MHz, $CDCl_3$)** δ_C: 24.1, 25.7, 38.1, 39.1, 56.8, 84.0, 126.8, 127.7, 128.2, 128.6, 130.6, 132.9, 141.0, 142.3, 170.4, 199.9; **HRMS (ESN$^-$)** [$C_{20}H_{22}O_4$] requires [M-H$^+$] 325.1440, found 325.1446 (+ 1.8 ppm).

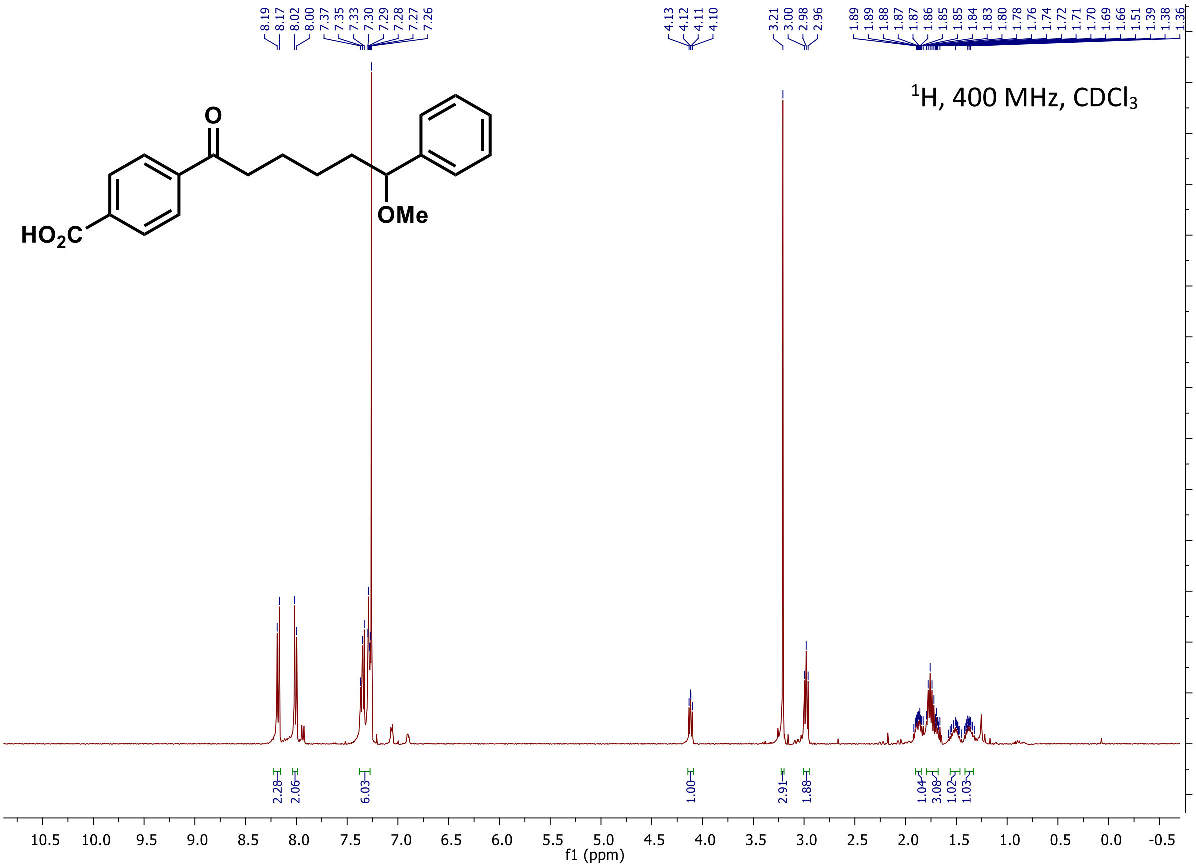
1H, 400 MHz, CDCl3
HO2C
O
OMe
f1 (ppm)

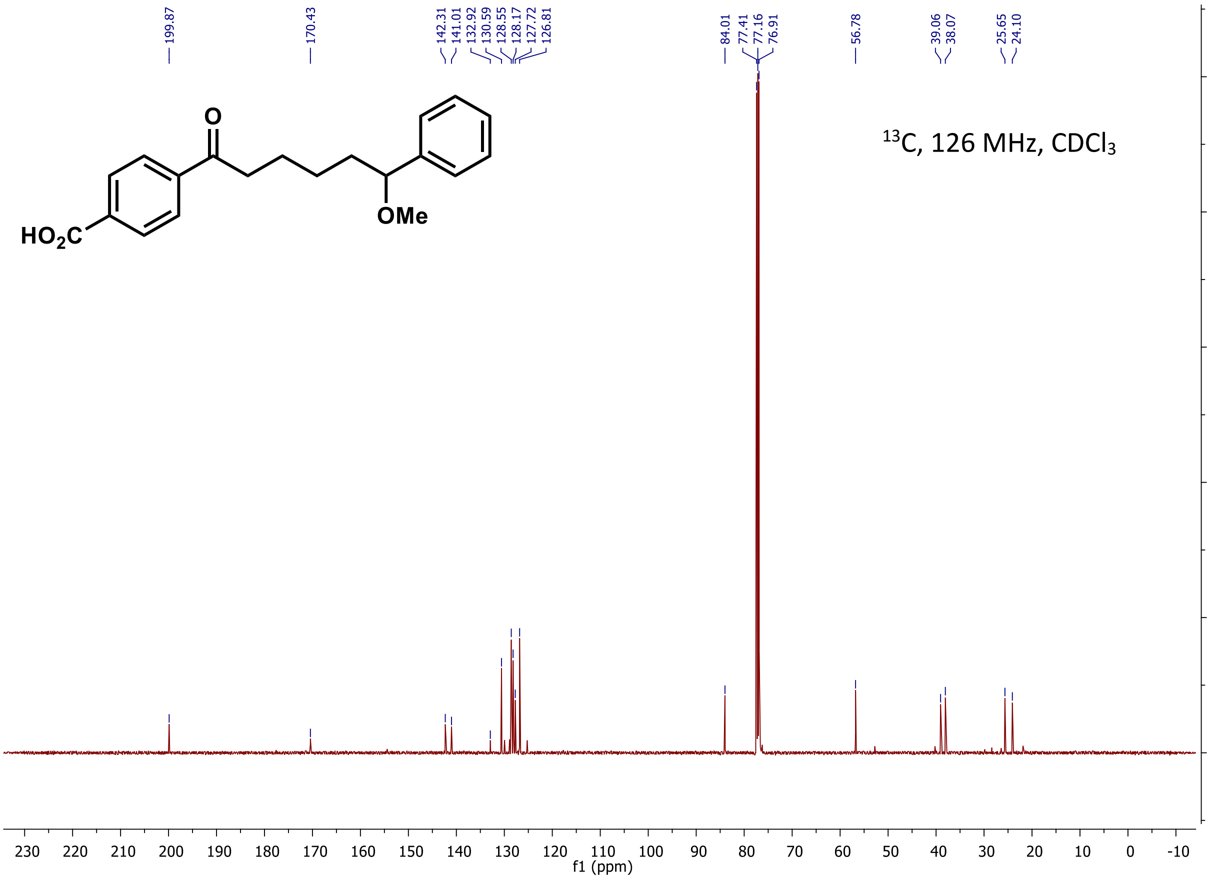
13C, 126 MHz, CDCl3
HO2C
O
OMe
f1 (ppm)

(7)

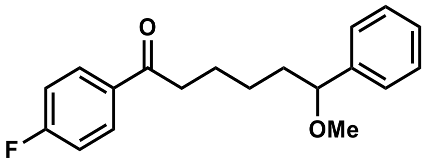

Prepared according to the General Procedure Y using **S7** (81.0 mg, 0.30 mmol, 1 equiv.), *n*-Bu_4NPF_6 (232 mg, 0.60 mmol, 2 equiv.), dichloromethane (4.5 mL) and MeOH (1.5 mL). The yield was determined by crude ^{1}H NMR using 1,3,5-trimethylbenzene (42 µL, 1 equiv.) as an internal standard: 60%. Purification by flash column chromatography (eluent = 5% EtOAc in hexanes, silica gel) to afford product as a yellow oil (61.1 mg, 57% yield).

R_f = 0.45 (eluent = 20% EtOAc in hexanes); **ν_{max} / cm^{-1}** (thin film) 2936, 1684, 1595, 1504, 1452, 1408, 1226, 1155, 1105; **^{1}H NMR (500 MHz, $CDCl_3$)** δ_H: 1.29-1.40 (1H, m), 1.43-1.55 (1H, m), 1.63-1.78 (3H, m), 1.81-1.92 (1H, m), 2.91 (2H, dd, *J* 7.9, 6.9 Hz), 3.20 (3H, s), 4.10 (1H, dd, *J* 7.3, 6.0 Hz), 7.05-7.14 (2H, m), 7.24-7.32 (3H, m), 7.31-7.37 (2H, m), 7.89-8.00 (2H, m); **^{13}C NMR (126 MHz, $CDCl_3$)** δ_C: 24.2, 25.7, 38.1, 38.5, 56.8, 83.9, 115.7 (d, J 21.4 Hz), 126.8, 127.6, 128.5, 130.8 (d, *J* 10.1 Hz), 133.5 (d *J* 2.5 Hz), 142.4, 165.7 (d, *J* 254 Hz), 198.8; **^{19}F NMR (471 MHz, $CDCl_3$)** δ_F: -105.7; **HRMS (ES$^+$)** [$C_{19}H_{21}O_2F$] requires $[M+Na]^+$ 323.1423, found 323.1421 (-0.6 ppm).

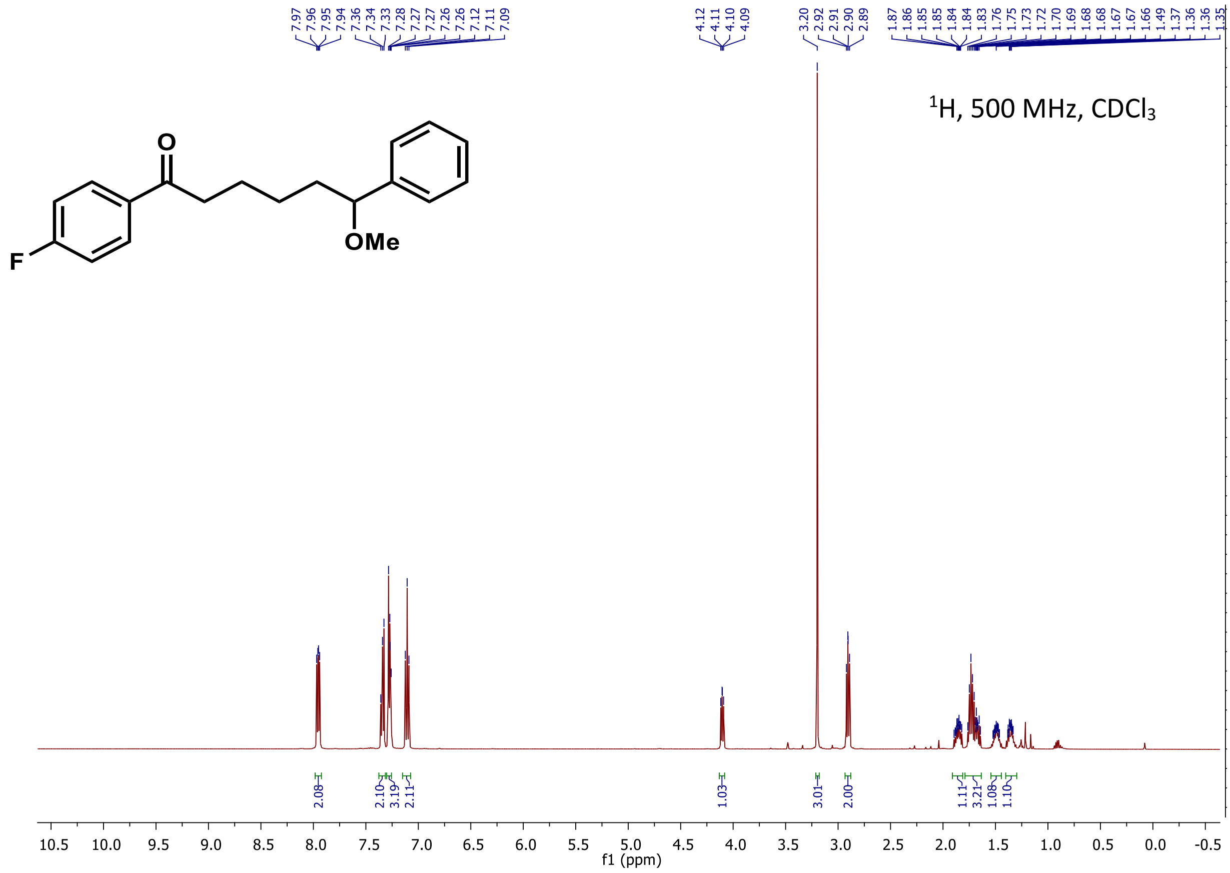

O
F
OMe
¹H, 500 MHz, CDCl₃
f1 (ppm)

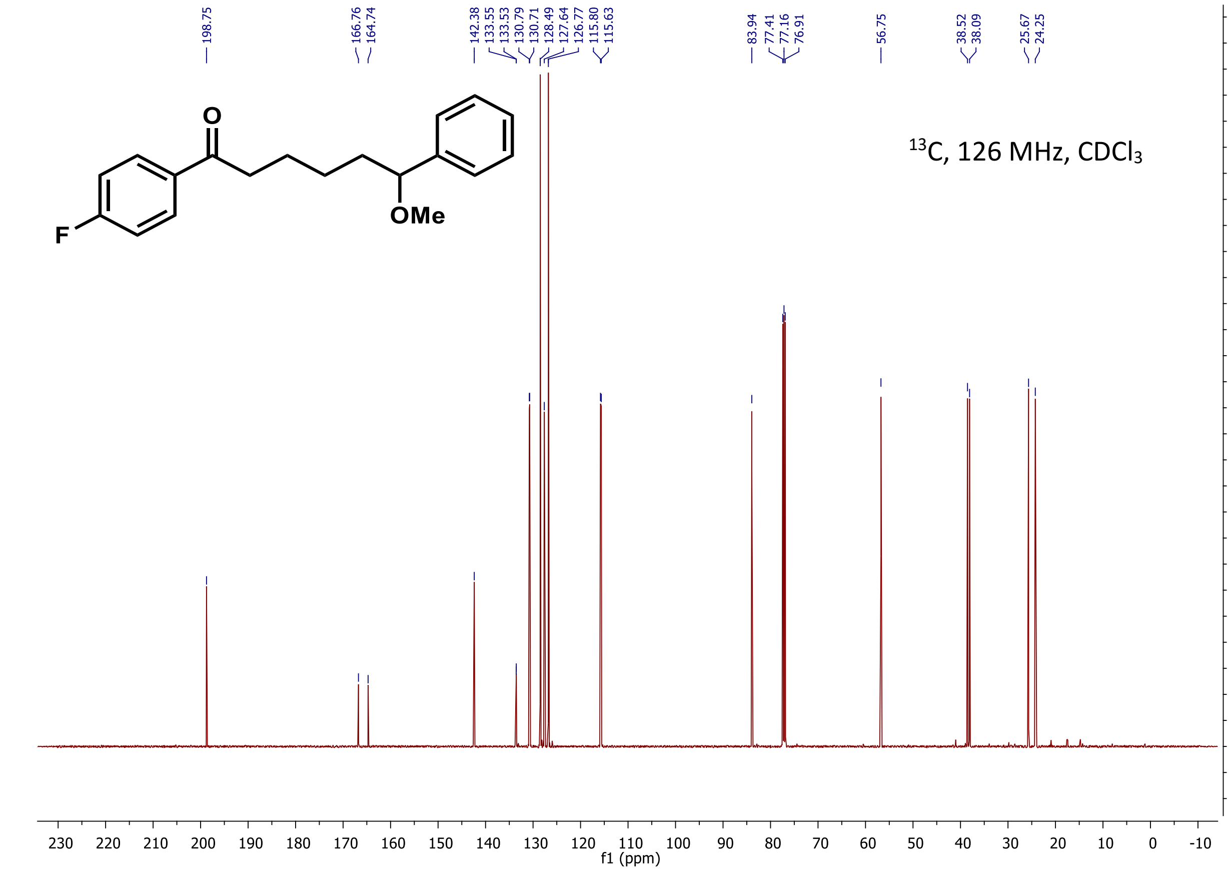

O
F
OMe
¹³C, 126 MHz, CDCl₃
f1 (ppm)

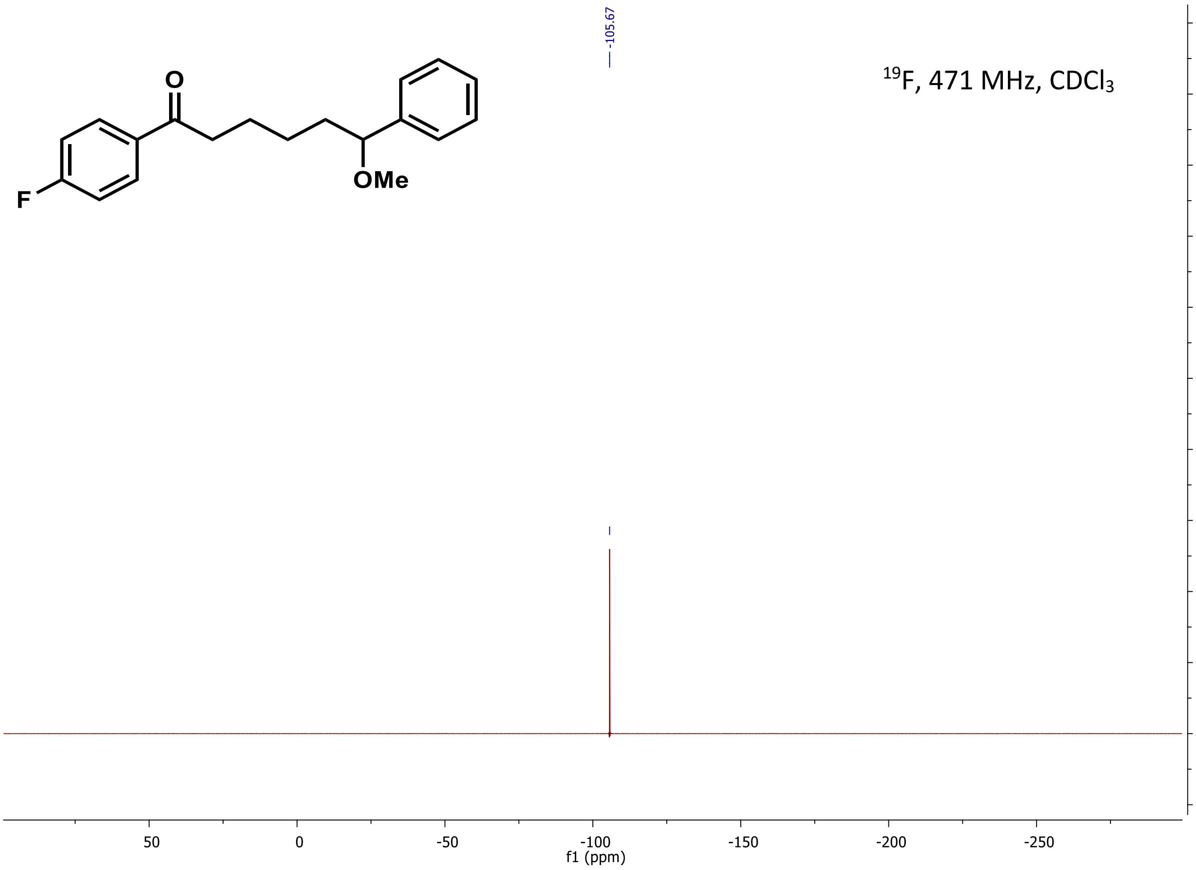

O
F
OMe
-105.67
¹⁹F, 471 MHz, CDCl₃
50
0
-50
-100
-150
-200
-250
f1 (ppm)

(8)

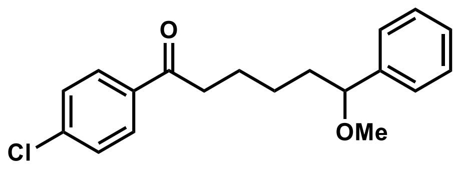

Prepared according to the General Procedure Y using **S8** (86.0 mg, 0.30 mmol, 1 equiv.), *n*-Bu_4NPF_6 (232 mg, 0.60 mmol, 2 equiv.), dichloromethane (4.5 mL) and MeOH (1.5 mL). The yield was determined by crude ^{1}H NMR using 1,3,5-trimethylbenzene (42 µL, 1 equiv.) as an internal standard: 45%. Purification by flash column chromatography (eluent = 5 to 15% EtOAc in hexanes, silica gel) to afford product as a colourless oil (31.3 mg, 33% yield).

R_f = 0.48 (eluent = 20% EtOAc in hexanes); **ν_{max} / cm^{-1}** (thin film) 2933, 1684, 1587, 1452, 1398, 1090, 1011; **^{1}H NMR (500 MHz, $CDCl_3$)** δ_H: 1.29-1.40 (1H, m), 1.43-1.54 (1H, m), 1.63-1.77 (3H, m), 1.79-1.91 (1H, m), 2.90 (2H, dd, *J* 8.1, 6.8 Hz), 3.20 (3H, s), 4.10 (1H, dd, *J* 7.4, 5.9 Hz), 7.24-7.31 (3H, m), 7.31-7.38 (2H, m), 7.42 (2H, d, *J* 8.8 Hz), 7.87 (2H, d, *J* 8.8 Hz), **^{13}C NMR (126 MHz, $CDCl_3$)** δ_C: 24.3, 25.7, 38.1, 38.6, 56.8, 84.0, 126.8, 127.7, 128.5, 129.0, 129.6, 135.4, 139.5, 142.4, 199.2; **HRMS (ES^+)** [$C_{19}H_{21}O_2Cl$] requires $[M+Na]^+$ 339.1128, found 339.1122 (- 1.8 ppm).

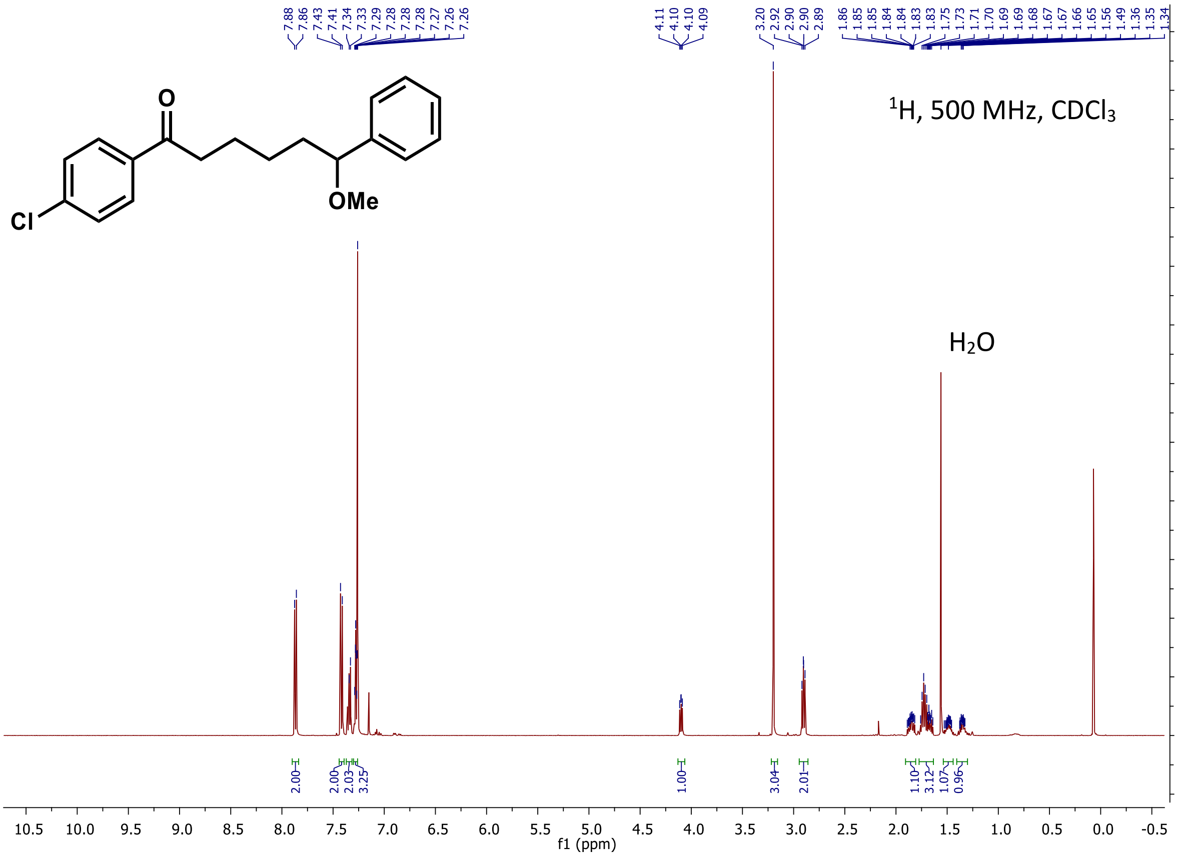
O
Cl
OMe
1H, 500 MHz, CDCl3
H2O
f1 (ppm)

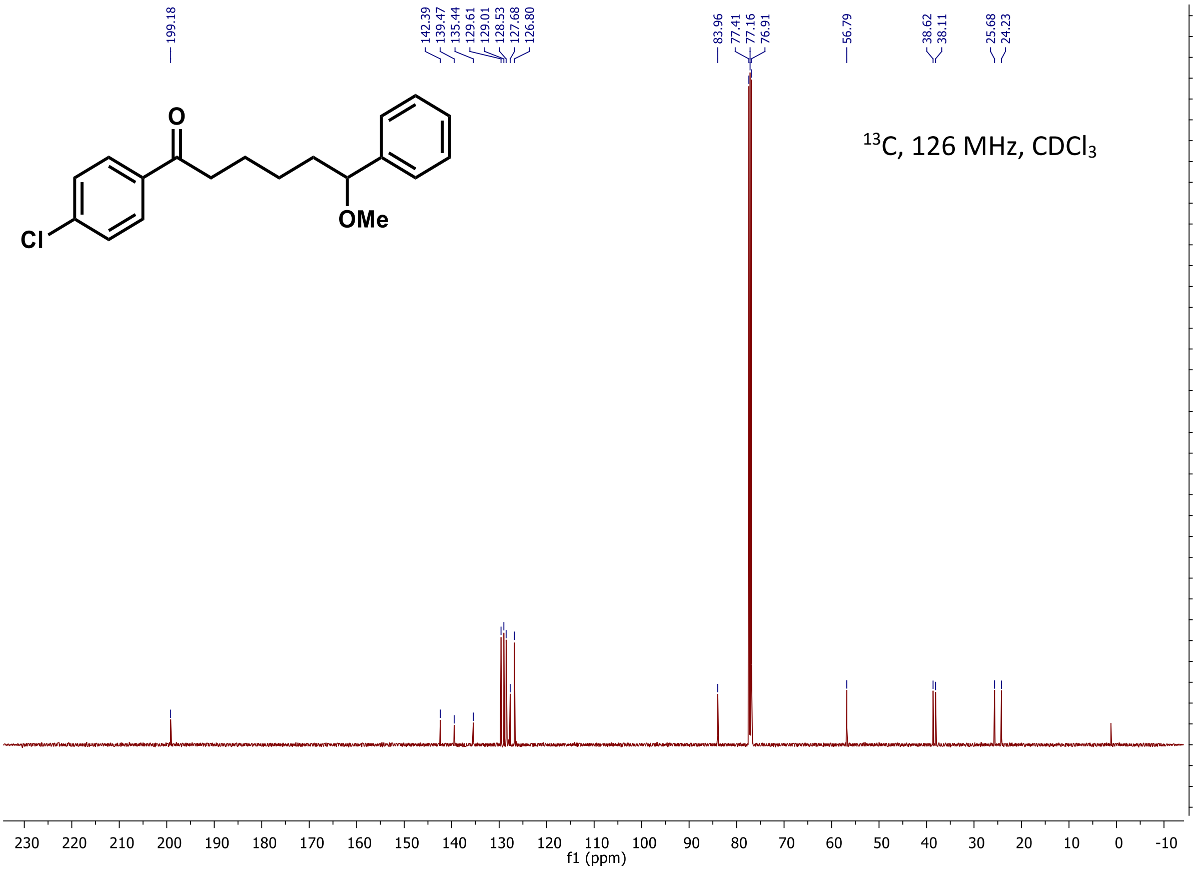
O
Cl
OMe
13C, 126 MHz, CDCl3
f1 (ppm)

(9)

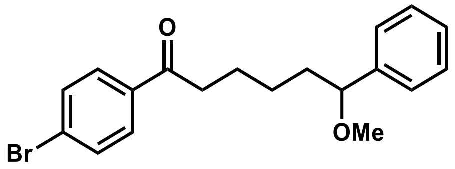

Prepared according to the General Procedure Y using **S9** (99.4 mg, 0.30 mmol, 1 equiv.), *n*-Bu_4NPF_6 (232 mg, 0.60 mmol, 2 equiv.), dichloromethane (4.5 mL) and MeOH (1.5 mL). The yield was determined by crude ^{1}H NMR using 1,3,5-trimethylbenzene (42 µL, 1 equiv.) as an internal standard: 56%. Purification by flash column chromatography (eluent = 5 to 15% EtOAc in hexanes, silica gel) to afford product as a colourless oil (57.3 mg, 53% yield).

R_f = 0.42 (eluent = 20% EtOAc in hexanes); **ν_{max} / cm^{-1}** (thin film) 2934, 1684, 1584, 1454, 1103, 1059; **^{1}H NMR (500 MHz, $CDCl_3$)** δ_H: 1.30-1.40 (1H, m), 1.43-1.54 (1H, m), 1.64-1.76 (3H, m), 1.80-1.91 (1H, m), 2.90 (2H, dd, *J* 8.0, 6.7 Hz), 3.20 (3H, s), 4.10 (1H, dd, *J* 7.4, 5.9 Hz), 7.24-7.30 (3H, m), 7.31-7.37 (2H, m), 7.58 (2H, d, *J* 8.7 Hz), 7.79 (2H, d, *J* 8.7 Hz); **^{13}C NMR (126 MHz, $CDCl_3$)** δ_C: 24.2, 25.7, 38.1, 38.6, 56.8, 84.0, 126.8, 127.7, 128.2, 128.5, 129.7, 132.0, 135.8, 142.4, 199.4; **HRMS (ES$^+$)** [$C_{19}H_{21}O_2Br$] requires $[M+Na]^+$ 383.0623, found 383.0625 (+ 0.5 ppm).

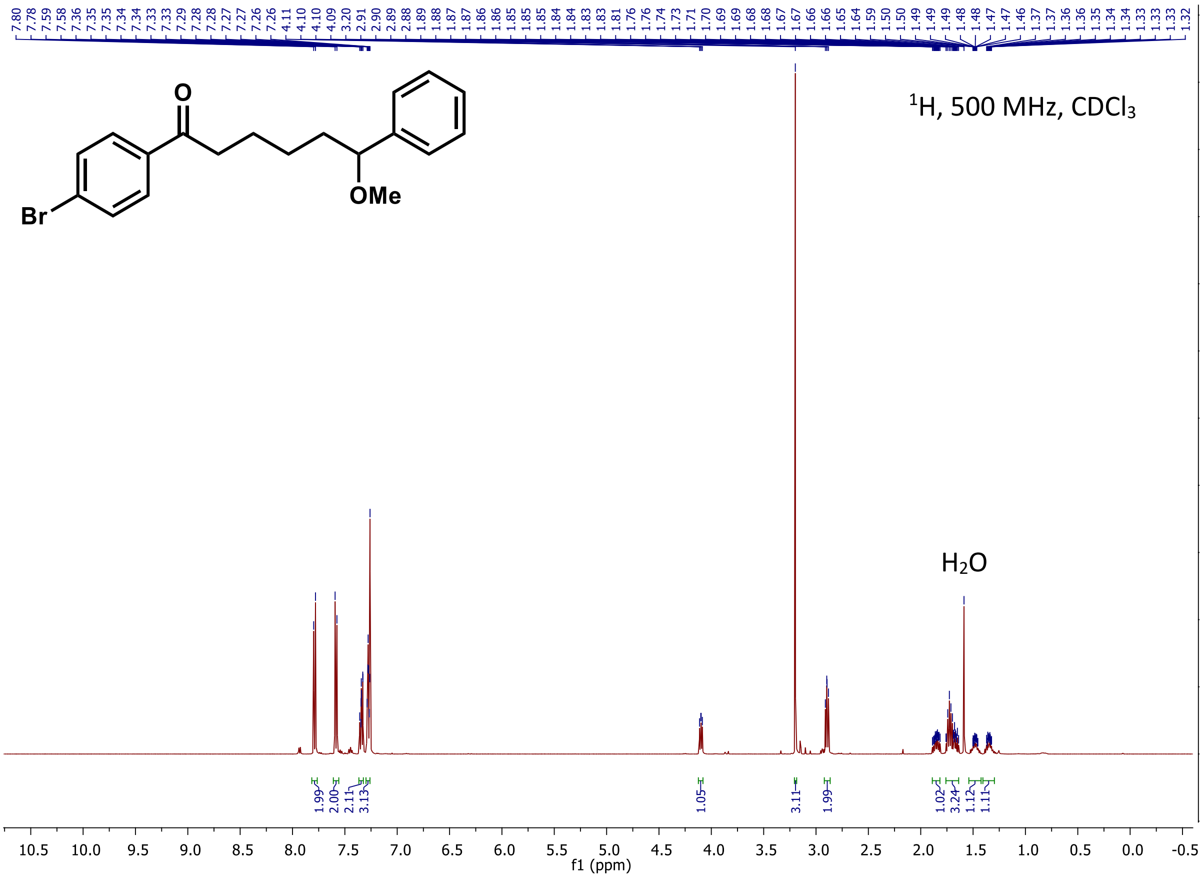

7.80
7.78
7.59
7.58
7.36
7.35
7.35
7.34
7.34
7.33
7.33
7.29
7.28
7.28
7.27
7.27
7.26
7.26
4.11
4.10
4.09
3.20
2.91
2.90
2.89
2.88
1.89
1.88
1.87
1.87
1.86
1.86
1.85
1.85
1.85
1.84
1.84
1.83
1.83
1.81
1.76
1.74
1.73
1.71
1.70
1.69
1.69
1.68
1.68
1.67
1.67
1.66
1.66
1.65
1.64
1.59
1.50
1.50
1.49
1.49
1.48
1.48
1.47
1.47
1.46
1.37
1.37
1.36
1.35
1.34
1.34
1.33
1.33
1.33
1.32
O
Br
OMe
^{1}H, 500 MHz, $CDCl_3$
H_2O
1.99
2.00
2.11
3.13
1.05
3.11
1.99
1.02
3.24
1.12
1.11
10.5 10.0 9.5 9.0 8.5 8.0 7.5 7.0 6.5 6.0 5.5 5.0 4.5 4.0 3.5 3.0 2.5 2.0 1.5 1.0 0.5 0.0 -0.5
f1 (ppm)

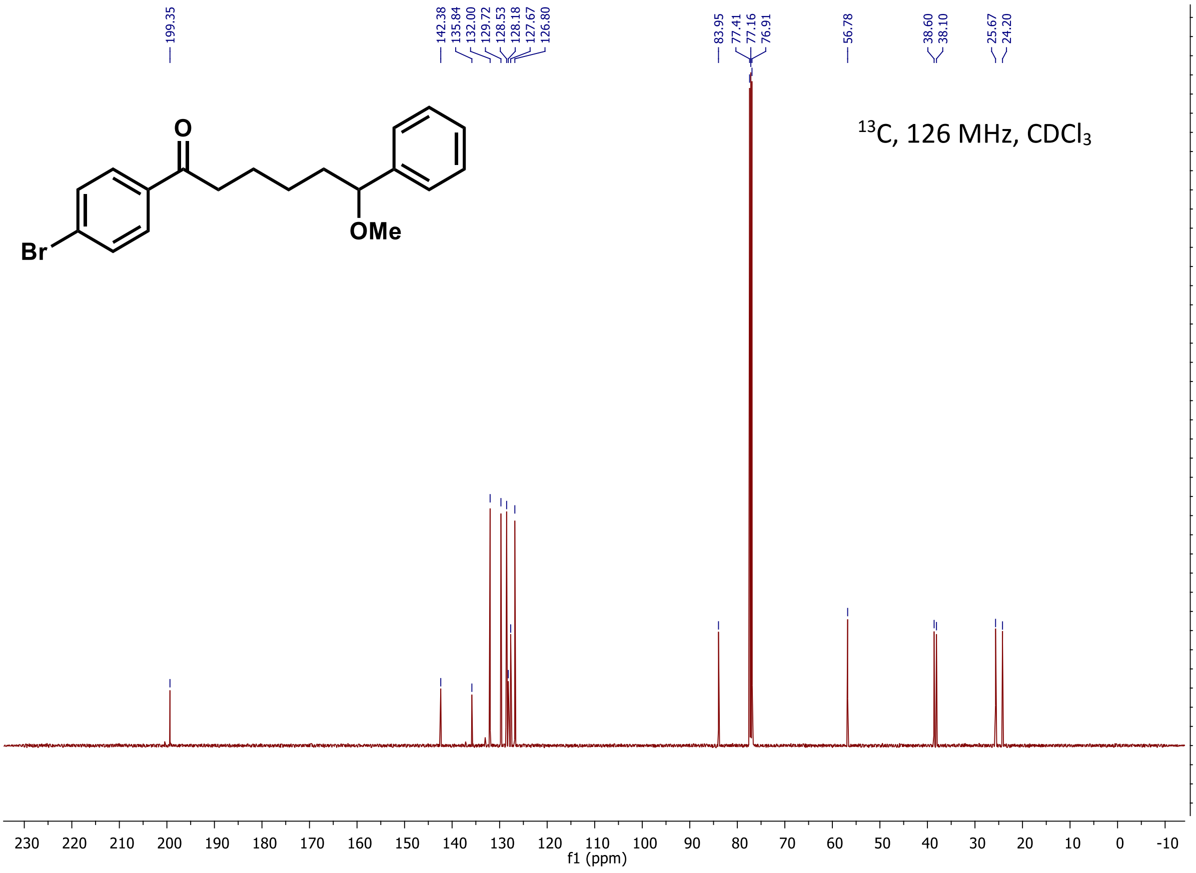

199.35
142.38
135.84
132.00
129.72
128.53
128.18
127.67
126.80
83.95
77.41
77.16
76.91
56.78
38.60
38.10
25.67
24.20
O
Br
OMe
^{13}C, 126 MHz, $CDCl_3$
230 220 210 200 190 180 170 160 150 140 130 120 110 100 90 80 70 60 50 40 30 20 10 0 -10
f1 (ppm)

(10)

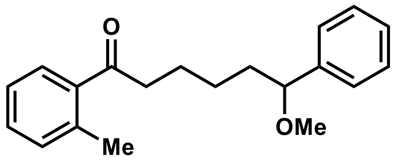

Prepared according to the General Procedure **S10** (79.8 mg, 0.30 mmol, 1 equiv.), *n*-Bu_4NPF_6 (232 mg, 0.60 mmol, 2 equiv.), dichloromethane (4.5 mL) and MeOH (1.5 mL). The yield was determined by crude ^{1}H NMR using 1,3,5-trimethylbenzene (42 µL, 1 equiv.) as an internal standard: 49%. Purification by flash column chromatography (eluent = 5 to 10% EtOAc in hexanes, silica gel) to afford product as a colourless oil (36.4 mg, 41% yield).

R_f = 0.67 (eluent =10% EtOAc in hexanes); **ν_{max} / cm^{-1}** (thin film) 2937, 1730, 1600, 1244, 1043, 906, 721; **^{1}H NMR (500 MHz, $CDCl_3$)** δ_H: 1.28-1.37 (1H, m), 1.42-1.51 (1H, m), 1.63-1.74 (3H, m), 1.81-1.88 (1H, m), 2.46 (3H, s), 2.84-2.88 (2H, m), 3.20 (3H, s), 4.08-4.11 (1H, m), 7.22-7.26 (2H, m), 7.26-7.29 (3H, m), 7.33-7.36 (3H, m), 7.56-7.59 (1H, m); **^{13}C NMR (126 MHz, $CDCl_3$)** δ_C: 21.3, 24.4, 25.6, 38.1, 41.7, 56.8, 84.0, 125.7, 126.8, 127.6, 128.4, 131.2, 132.0, 138.0, 138.4, 142.2, 204.8; **HRMS (CI^+)** [$C_{20}H_{24}O_2$] requires $[M+H]^+$297.1855, found 297.1852 (- 1.0 ppm).

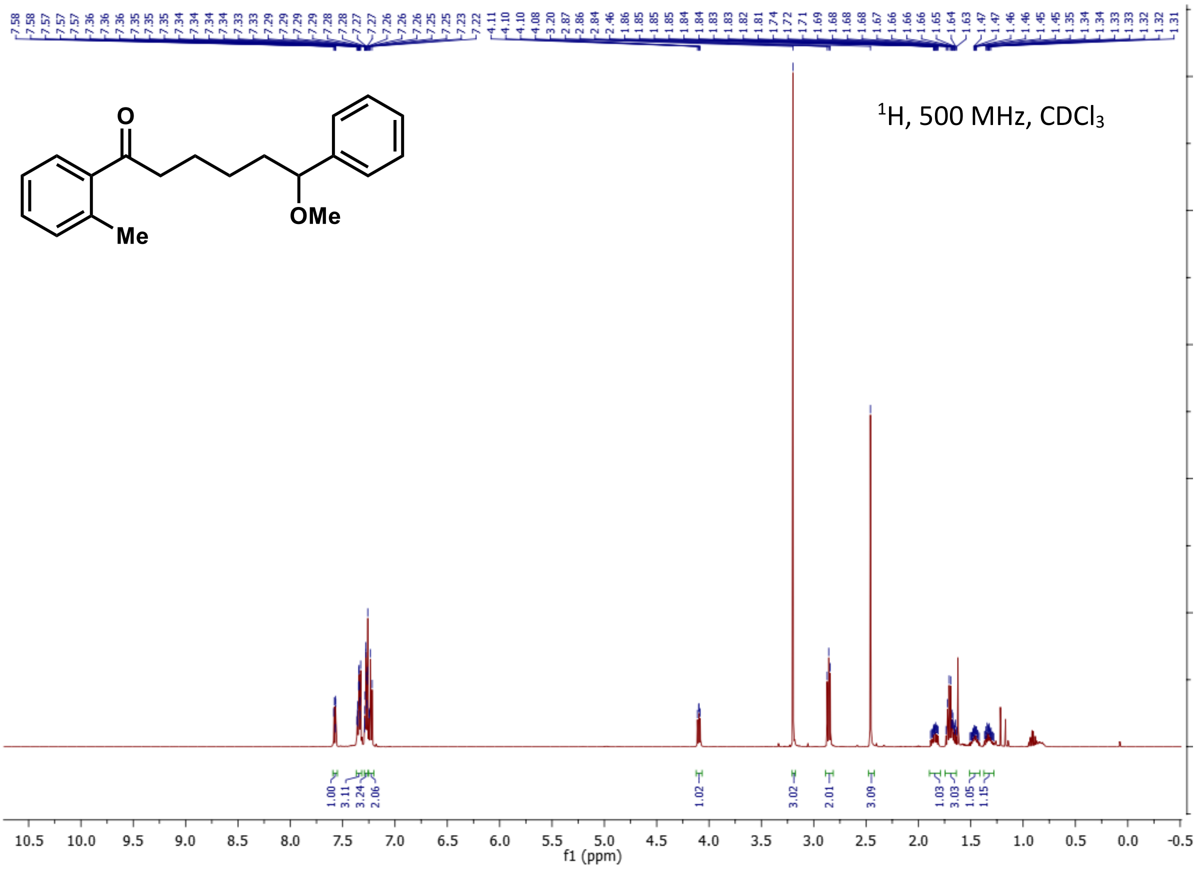

^{1}H, 500 MHz, CDCl$_3$
O
Me
OMe
f1 (ppm)

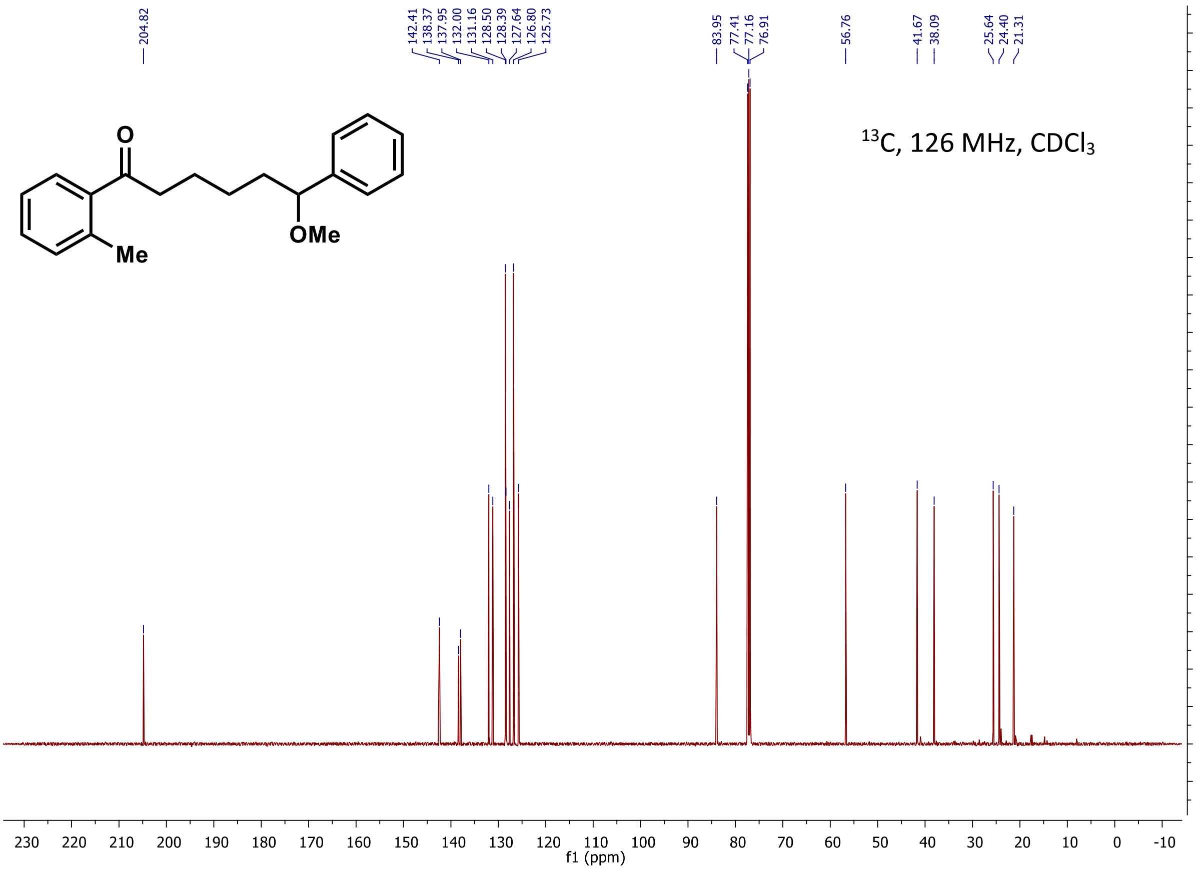

^{13}C, 126 MHz, CDCl$_3$
O
Me
OMe
f1 (ppm)

(11)

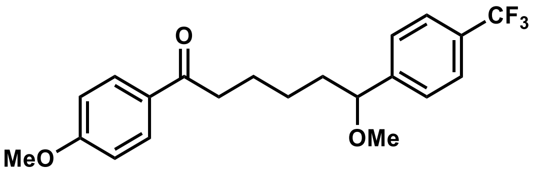

Prepared according to the General Procedure X using **S11** (105.0 mg, 0.30 mmol, 1 equiv.), *n*-Bu_4NPF_6 (232 mg, 0.60 mmol, 2 equiv.), dichloromethane (4.5 mL) and MeOH (1.5 mL). The yield was determined by crude ^{1}H NMR using 1,3,5-trimethylbenzene (42 µL, 1 equiv.) as an internal standard: 55%. Purification by flash column chromatography (eluent = 5 to 15% EtOAc in hexanes, silica gel) to afford product as a colourless oil (52.9 mg, 46% yield).

R_f = 0.37 (eluent = 15% EtOAc in hexanes); **ν_{max} / cm^{-1}** (thin film) 2937, 1674, 1599, 1510, 1418, 1259, 1166, 1120, 1064; **^{1}H NMR (500 MHz, $CDCl_3$)** δ_H: 1.29-1.40 (1H, m), 1.41-1.53 (1H, m), 1.61-1.78 (3H, m), 1.78-1.89 (1H, m), 2.89 (2H, dd, *J* 7.9, 6.8 Hz), 3.21 (3H, s), 3.86 (3H, s), 4.17 (1H, dd, *J* 7.4, 5.8 Hz), 6.92 (2H, d, *J* 8.9 Hz), 7.39 (2H, d, *J* 8.1 Hz), 7.60 (2H, d, *J* 8.1 Hz), 7.91 (2H, d, *J* 8.9 Hz), **^{13}C NMR (126 MHz, $CDCl_3$)** δ_C: 24.4, 25.6, 38.1, 38.2, 55.6, 57.0, 83.4, 113.8, 124.3 (q, 272 Hz), 125.5 (q, 3.8 Hz), 127.0, 129.8 (q, 32.8 Hz), 130.2, 130.4, 146.8, 163.5, 198.9; **^{19}F NMR (471 MHz, $CDCl_3$)** δ_F: -62.4; **HRMS (ES^+)** [$C_{21}H_{23}F_3O_3$] requires $[M+H]^+$ 381.1678, found 381.1674 (-1.0 ppm).

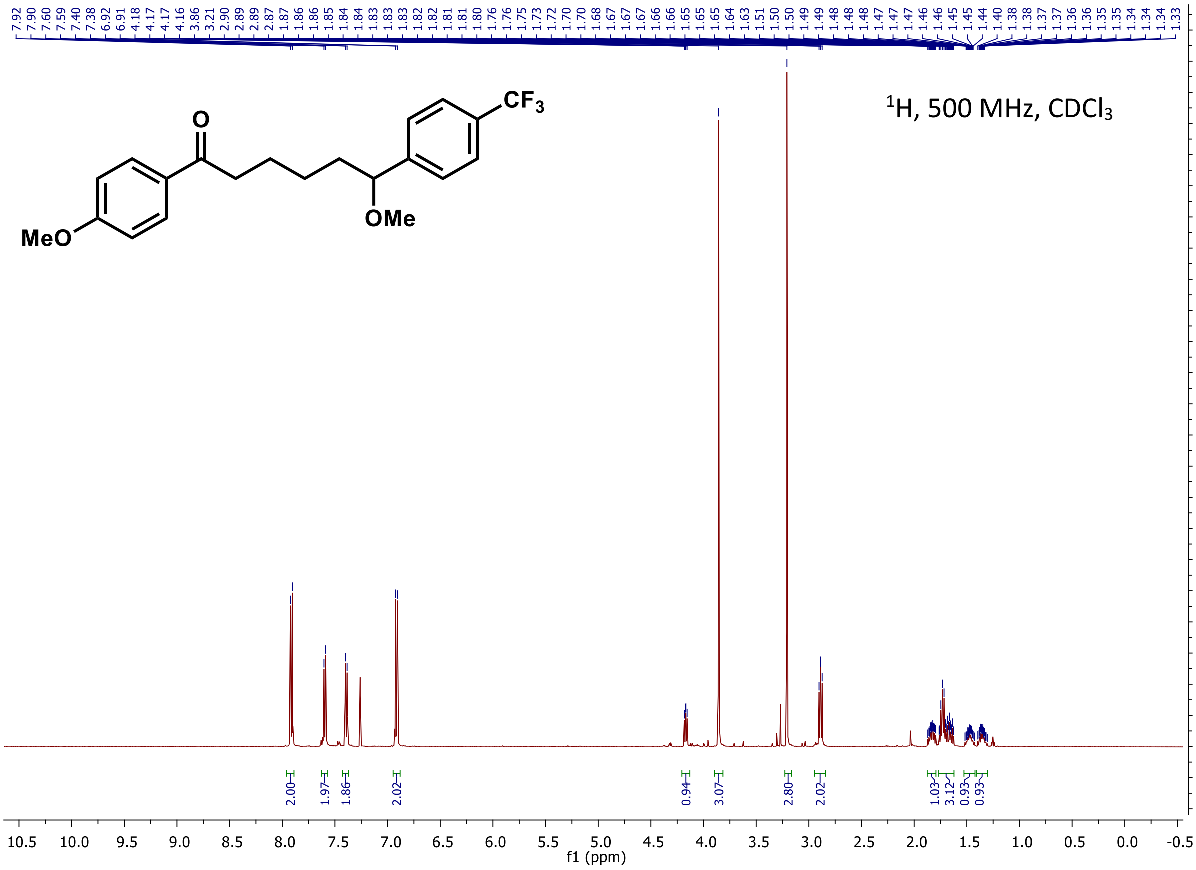

¹H, 500 MHz, CDCl₃
CF₃
O
MeO
OMe
f1 (ppm)

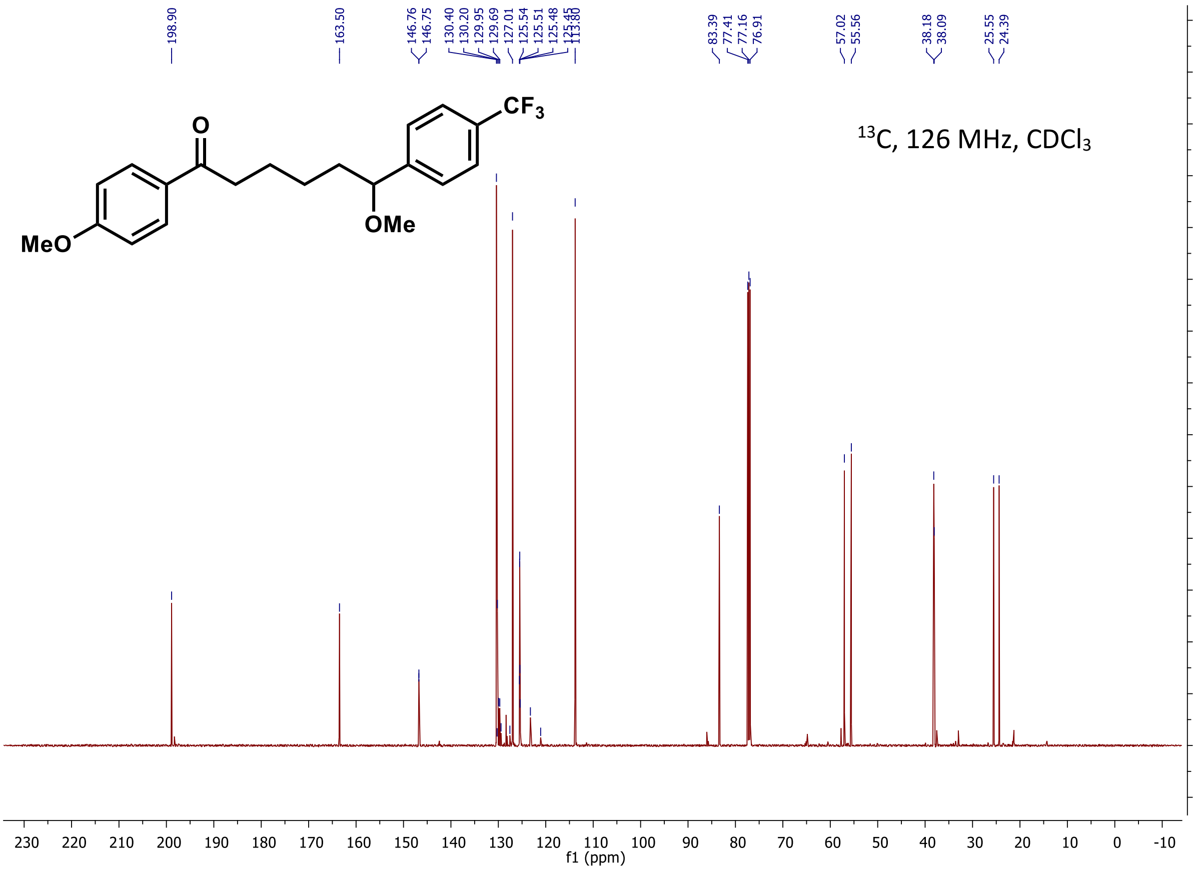

¹³C, 126 MHz, CDCl₃
CF₃
O
MeO
OMe
f1 (ppm)

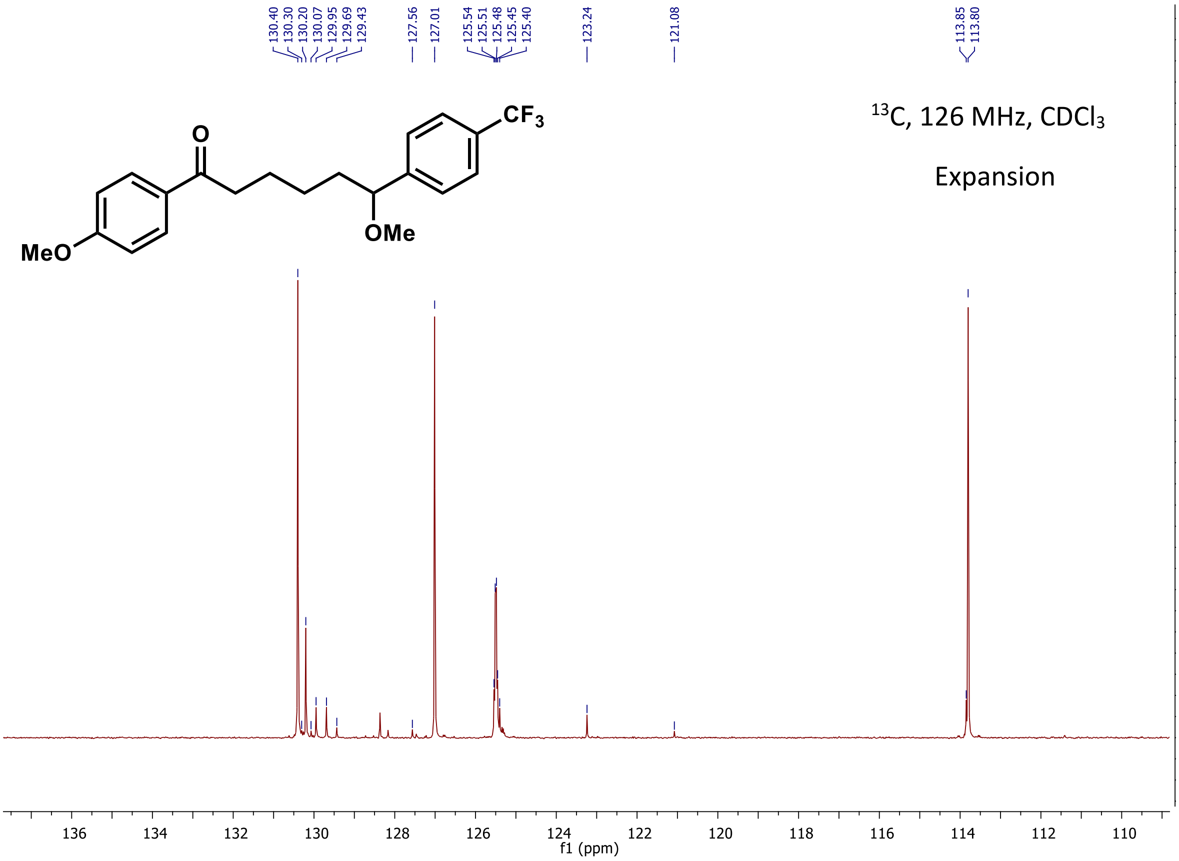

130.40
130.30
130.20
130.07
129.95
129.69
129.43
127.56
127.01
125.54
125.51
125.48
125.45
125.40
123.24
121.08
113.85
113.80
CF3
O
MeO
OMe
^{13}C, 126 MHz, CDCl$_3$
Expansion
136
134
132
130
128
126
124
122
120
118
116
114
112
110
f1 (ppm)

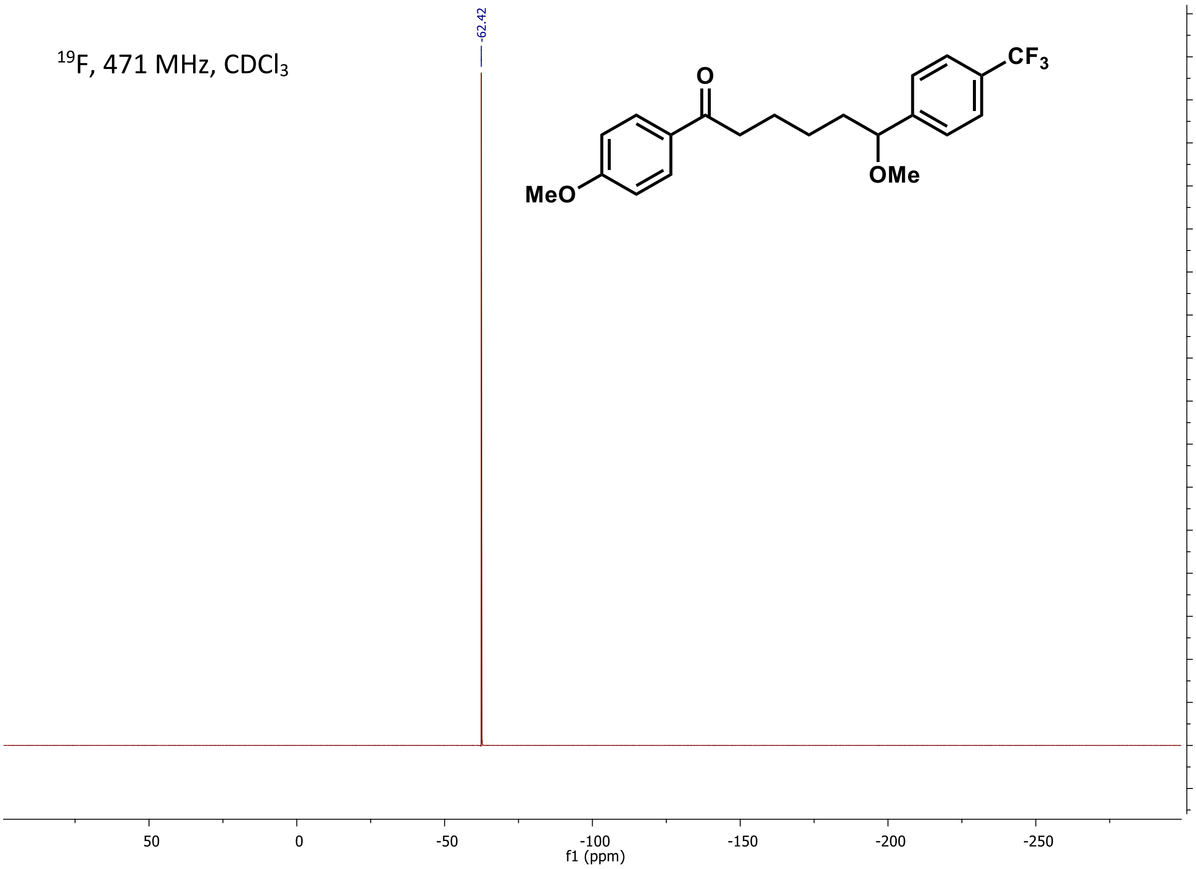

-62.42
^{19}F, 471 MHz, CDCl$_3$
O
CF3
MeO
OMe
50
0
-50
-100
-150
-200
-250
f1 (ppm)

(12)

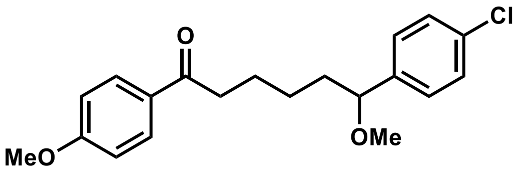

Prepared according to the General Procedure X using **S12** (95.0 mg, 0.30 mmol, 1 equiv.), *n*-Bu_4NPF_6 (232 mg, 0.60 mmol, 2 equiv.), dichloromethane (4.5 mL) and MeOH (1.5 mL). The yield was determined by crude ^{1}H NMR using 1,3,5-trimethylbenzene (42 µL, 1 equiv.) as an internal standard: 75%. Purification by flash column chromatography (eluent = 10 to 15% EtOAc in hexanes, silica gel) to afford product as a colourless oil (72.8 mg, 70% yield).

R_f = 0.29 (eluent =10% EtOAc in hexanes); **v_{max} / cm^{-1}** (thin film) 2927, 1674, 1600, 1559, 1170; **^{1}H NMR (500 MHz, $CDCl_3$)** δ_H: 1.28- 1.37 (1H, m), 1.41-1.52 (1H, m), 1.60-1.67 (1H, m), 1.69-1.75 (2H, m), 1.79-1.86 (1H, m), 2.87-2.90 (2H, t, *J* 7.4 Hz), 3.18 (3H, s), 3.87 (3H, s), 4.08 (1H, t, *J* 6.7 Hz), 6.92 (2H, d, *J* 8.9 Hz), 7.21 (2H, d, *J* 8.5 Hz), 7.31 (2H, d, *J* 8.3 Hz), 7.92 (2H, d, *J* 8.9 Hz); **^{13}C NMR (126 MHz, $CDCl_3$)** δ_C: 24.5, 25.6, 38.1, 38.3, 55.6, 56.8, 83.3, 113.8, 128.2, 128.7, 130.2, 130.4, 133.3, 141.0, 163.5, 199.0; **HRMS (EI^+)** [$C_{20}H_{23}ClO_3$] requires $[M-CH_3]^+$ 331.1101, Found 331.1094 (- 2.1 ppm). Loss of methyl group observed upon ionisation.

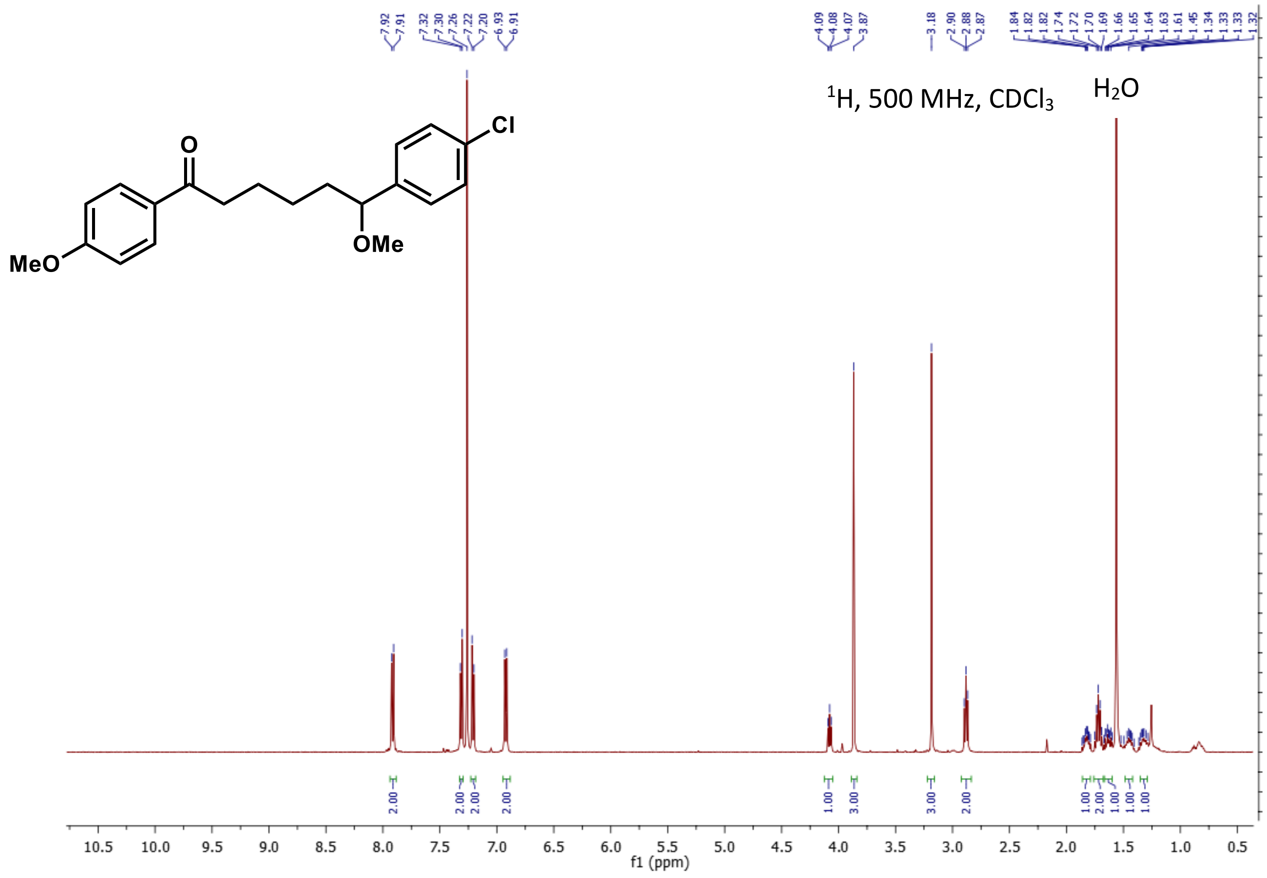

1H, 500 MHz, CDCl3
H2O
MeO
OMe
O
Cl
f1 (ppm)

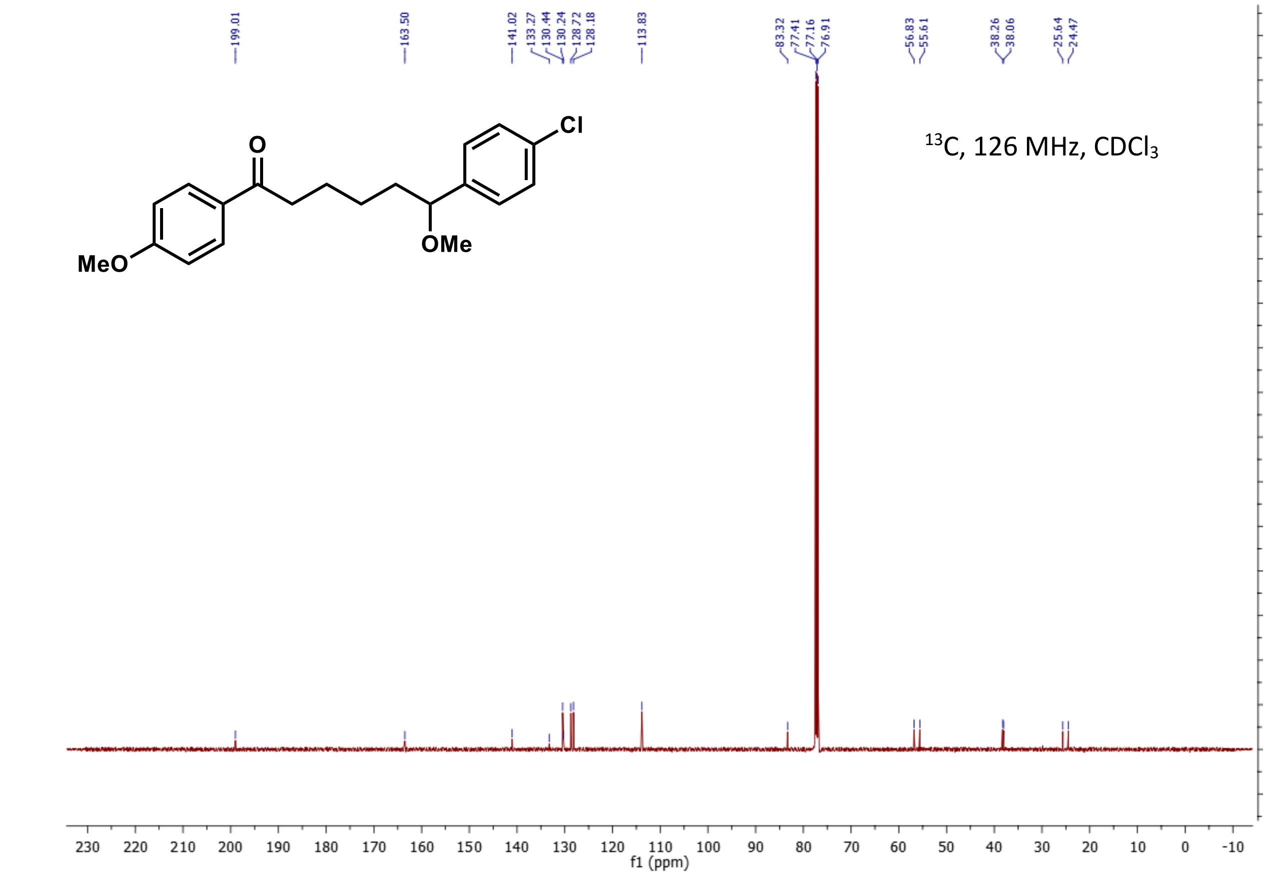

13C, 126 MHz, CDCl3
MeO
OMe
O
Cl
f1 (ppm)

(13)

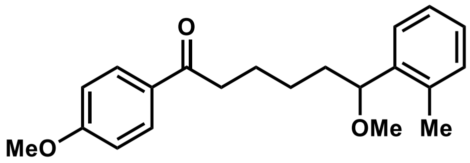

Prepared according to the General Procedure X using **S13** (88.9 mg, 0.30 mmol, 1 equiv.), *n*-Bu_4NPF_6 (232 mg, 0.60 mmol, 2 equiv.), dichloromethane (4.5 mL) and MeOH (1.5 mL). The yield was determined by crude ^{1}H NMR using 1,3,5-trimethylbenzene (42 µL, 1 equiv.) as an internal standard: 55%. Purification by flash column chromatography (eluent = 10 to 15% EtOAc in hexanes, silica gel) to afford product as a yellow oil (41.1 mg, 42% yield).

$\mathbf{R_f}$ = 0.20 (eluent =10% EtOAc in hexanes); $\mathbf{\nu_{max}}$ **/ cm^{-1}** (thin film) 2935, 1735, 1676, 1600, 1371, 1238, 1043, 910, 715; **^{1}H NMR (500 MHz, $CDCl_3$)** δ_H: 1.38- 1.47 (1H, m), 1.52-1.61 (1H, m), 1.62-1.82 (4H, m), 2.32 (3H, s), 2.89-2.92 (2H, m), 3.19 (3H, s), 3.87 (3H, s), 4.38-4.41 (1H, m), 6.91-6.94 (2H, m), 7.12-7.18 (2H, m), 7.19-7.23 (1H, m), 7.34-7.35 (1H, m), 7.91-7.94 (2H, m); **^{13}C NMR (126 MHz, $CDCl_3$)** δ_C: 19.2, 24.7, 26.0, 37.3, 38.4, 55.6, 56.7, 80.3, 113.8, 126.0, 126.3, 127.1, 130.3, 130.5, 130.5, 135.5, 140.6, 163.5, 199.1; **HRMS (EI$^+$)** [$C_{21}H_{26}O_3$] requires [M-MeOH+H]$^+$ 295.1698, Found 295.1692 (- 2.0 ppm). Elimination of methanol observed upon ionisation.

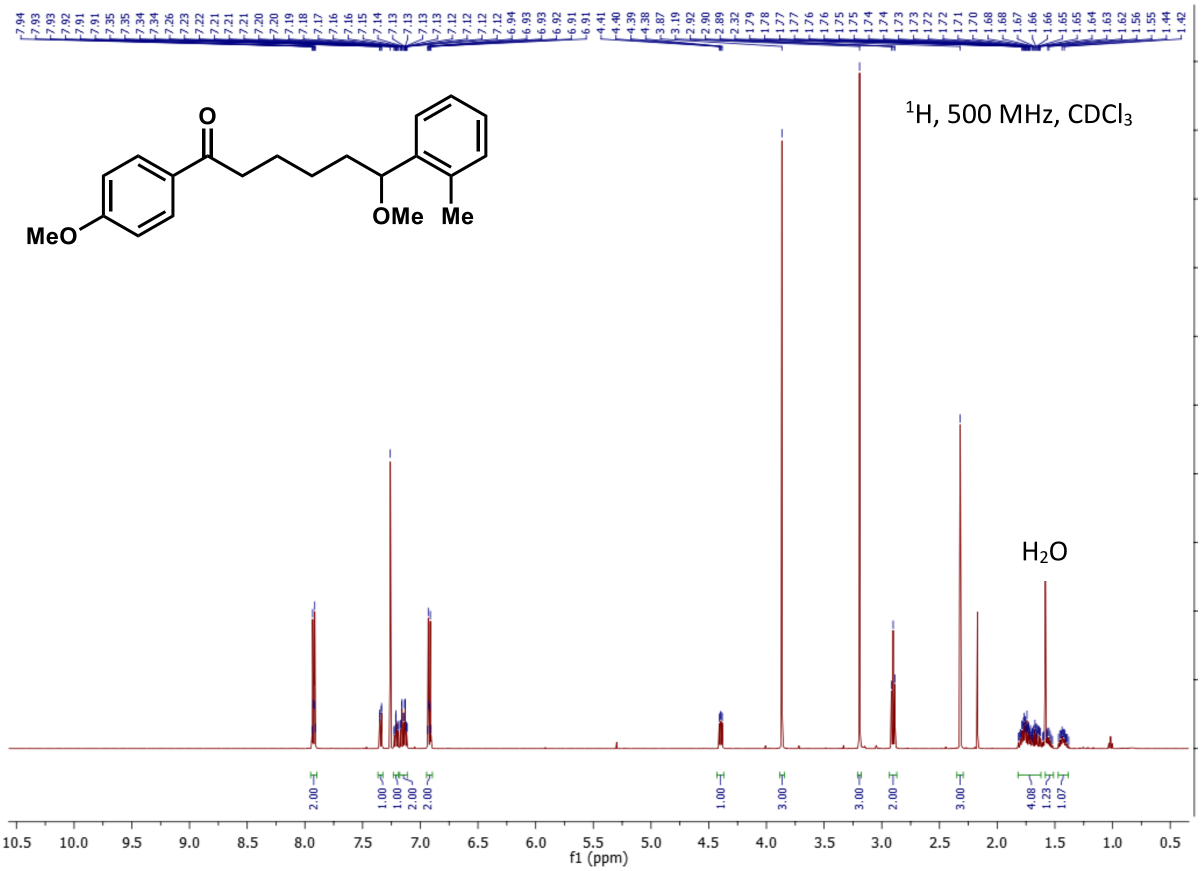
1H, 500 MHz, CDCl3
OMe
Me
MeO
O
H2O
f1 (ppm)

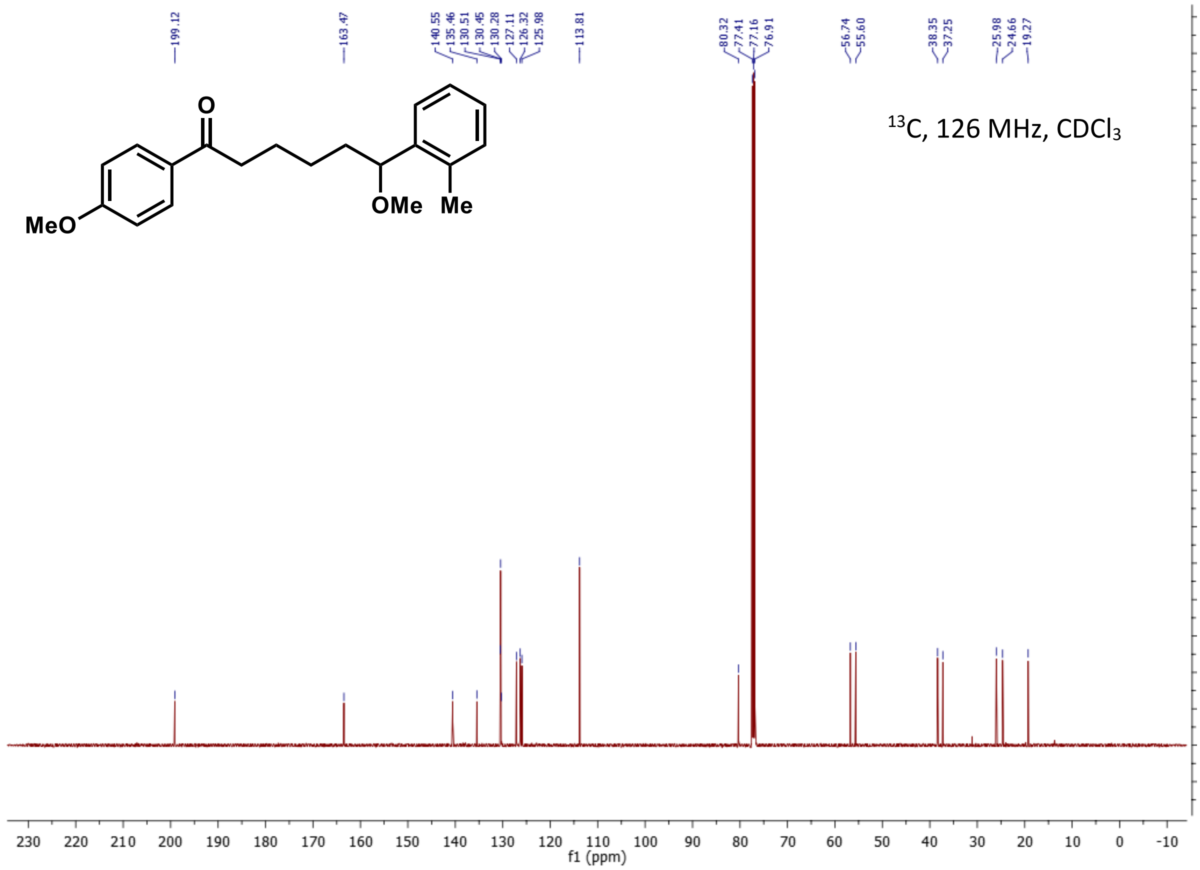
13C, 126 MHz, CDCl3
OMe
Me
MeO
O
f1 (ppm)

(14)

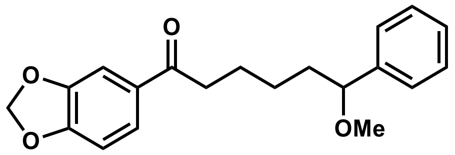

Prepared according to the General Procedure X using **S14** (88.9 mg, 0.30 mmol, 1 equiv.), *n*-Bu_4NPF_6 (232 mg, 0.60 mmol, 2 equiv.), dichloromethane (4.5 mL) and MeOH (1.5 mL). The yield was determined by crude ^{1}H NMR using 1,3,5-trimethylbenzene (42 µL, 1 equiv.) as an internal standard: 41%. Purification and isolation by silica gel chromatography proved challenging due to inseparable impurities.

<u>Clean resolved signals for product:</u>

^{1}H NMR (500 MHz, $CDCl_3$) δ_H: 6.02 (2H, s), 6.81 (1H, d, *J* 8.2 Hz), 7.48-7.52 (1H, m)

<u>Other identifiable product signals:</u>

^{1}H NMR (500 MHz, $CDCl_3$) δ_H: 2.80-2.85 (2H, m), 4.05-4.10 (1H, m)

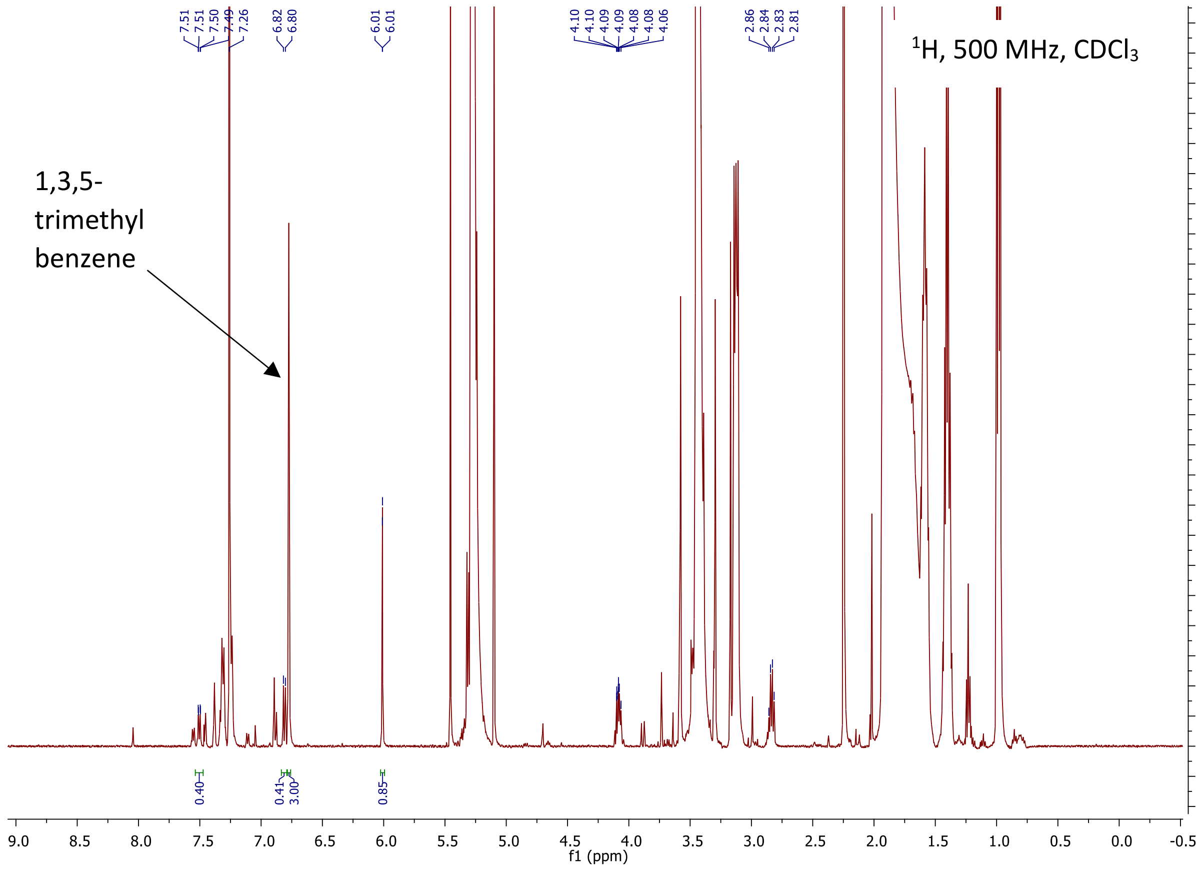

(15)

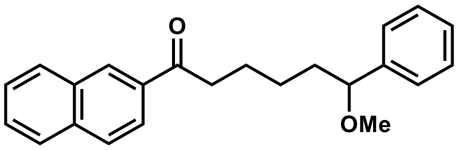

Prepared according to the General Procedure X using **S15** (90.7 mg, 0.30 mmol, 1 equiv.), *n*-Bu_4NPF_6 (232 mg, 0.60 mmol, 2 equiv.), dichloromethane (4.5 mL) and MeOH (1.5 mL). The yield was determined by crude ^{1}H NMR using 1,3,5-trimethylbenzene (42 µL, 1 equiv.) as an internal standard: 72%. Purification by flash column chromatography (eluent = 5 to 15% EtOAc in hexanes, silica gel) to afford product as a colourless oil (64.2 mg, 64% yield).

R_f = 0.36 (eluent = 20% EtOAc in hexanes); **ν_{max} / cm^{-1}** (thin film) 3649, 3549, 2980, 2939, 2816, 1680, 1527, 1240, 1064; **^{1}H NMR (500 MHz, $CDCl_3$)** δ_H: 1.34-1.45 (1H, m), 1.49-1.58 (1H, m), 1.66-1.95 (4H, m), 3.06-3.10 (2H, m), 3.21 (3H, s), 4.12 (1H, dd, *J* 7.4, 5.9 Hz), 7.27-7.31 (3H, m), 7.32-7.37 (2H, m), 7.51-7.64 (2H, m), 7.84-7.91 (2H, m), 7.94-7.98 (1H, m), 7.99-8.03 (1H, m), 8.44 (1H, d, *J* 1.1 Hz); **^{13}C NMR (126 MHz, $CDCl_3$)** δ_C: 24.5. 25.8, 38.2, 38.7, 56.8, 84.0, 124.1, 126.8, 126.9, 127.7, 127.9, 128.5, 128.5, 128.5, 129.7, 129.8, 132.7, 134.5, 135.7, 142.5, 200.4; **HRMS (EI$^+$)** [$C_{23}H_{24}O_2$] requires $[M]^+$ 332.1776, found 332.1767 (- 2.7 ppm).

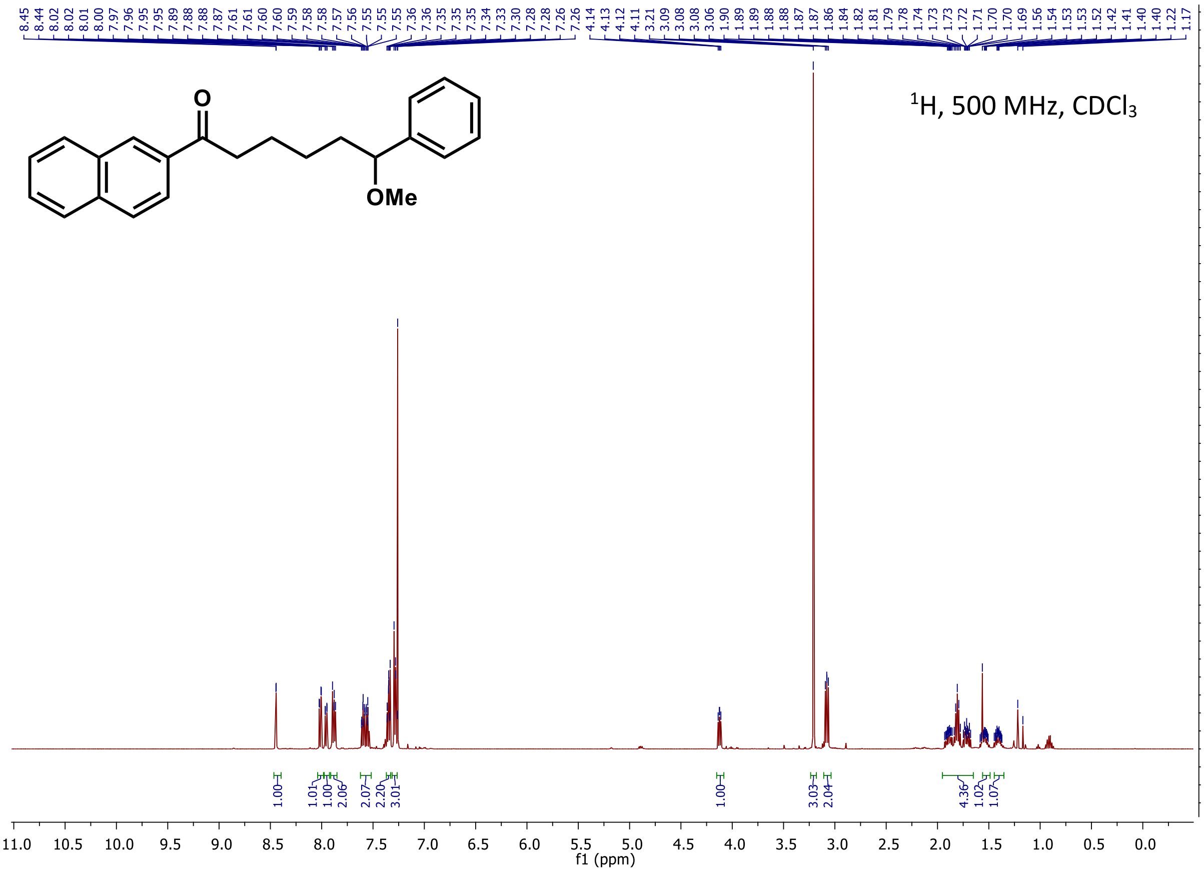

O
OMe
¹H, 500 MHz, CDCl₃
f1 (ppm)

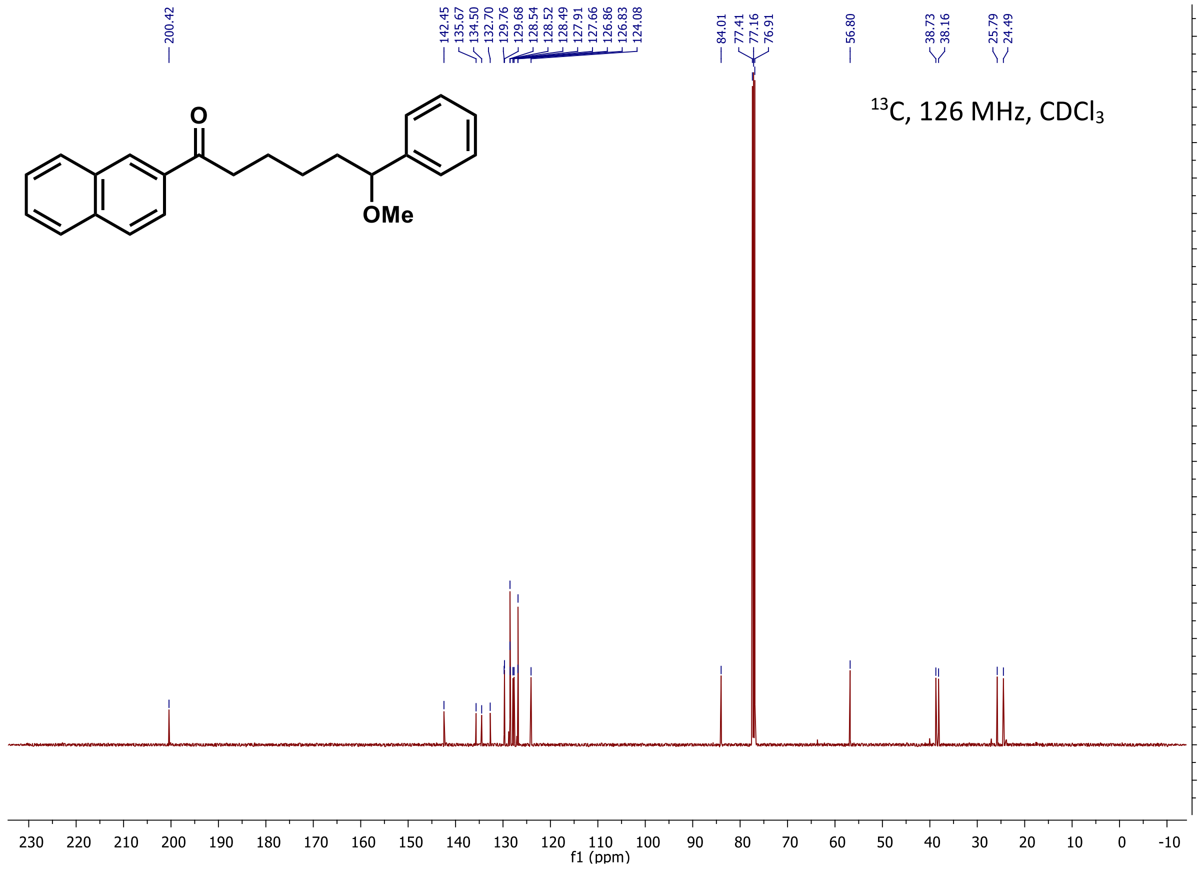

O
OMe
¹³C, 126 MHz, CDCl₃
200.42
142.45
135.67
134.50
132.70
129.76
129.68
128.54
128.52
128.49
127.91
127.66
126.86
126.83
124.08
84.01
77.41
77.16
76.91
56.80
38.73
38.16
25.79
24.49
f1 (ppm)

(16)

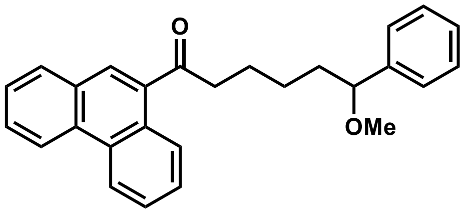

Prepared according to the General Procedure X using **S16** (105.7 mg, 0.30 mmol, 1 equiv.), *n*-Bu_4NPF_6 (232 mg, 0.60 mmol, 2 equiv.), dichloromethane (4.5 mL) and MeOH (1.5 mL). The yield was determined by crude ^{1}H NMR using 1,3,5-trimethylbenzene (42 µL, 1 equiv.) as an internal standard: 59%. Purification by flash column chromatography (eluent = 5 to 15% EtOAc in hexanes, silica gel) to afford product as yellow oil (65.5 mg, 57% yield).

R_f = 0.35 (eluent = 20% EtOAc in hexanes); **ν_{max} / cm^{-1}** (thin film) 3549, 3022, 2980, 2941, 2816, 1681, 1404, 1085; **^{1}H NMR (500 MHz, $CDCl_3$)** δ_H: 1.31-1.47 (1H, m), 1.50-1.60 (1H, m), 1.67-1.76 (1H, m), 1.78-1.95 (3H, m), 3.07-3.13 (2H, m), 3.20 (3H, s), 4.12 (1H, dd, *J* 7.2, 6.2 Hz), 7.26-7.30 (3H, m), 7.31-7.37 (2H, m), 7.62-7.66 (2H, m), 7.67-7.72 (1H, m), 7.71-7.77 (1H, m), 7.91-7.96 (1H, m), 8.05 (1H, m), 8.45-8.49 (1H, m), 8.68 (1H, d, *J* 8.0 Hz), 8.70-8.74 (1H, m); **^{13}C NMR (126 MHz, $CDCl_3$)** δ_C: 24.7, 25.7, 38.1, 42.4, 56.8, 84.0, 122.8, 123.0, 126.7, 126.8 (2C), 127.2, 127.6, 127.7, 128.5 (2C), 128.8, 129.0, 129.8, 130.2, 130.9, 131.9, 135.8, 142.4, 205.1; **HRMS (ES$^+$)** [$C_{27}H_{26}O_2$] requires [M+Na]$^+$ 405.1830, found 405.1832 (+ 0.5 ppm).

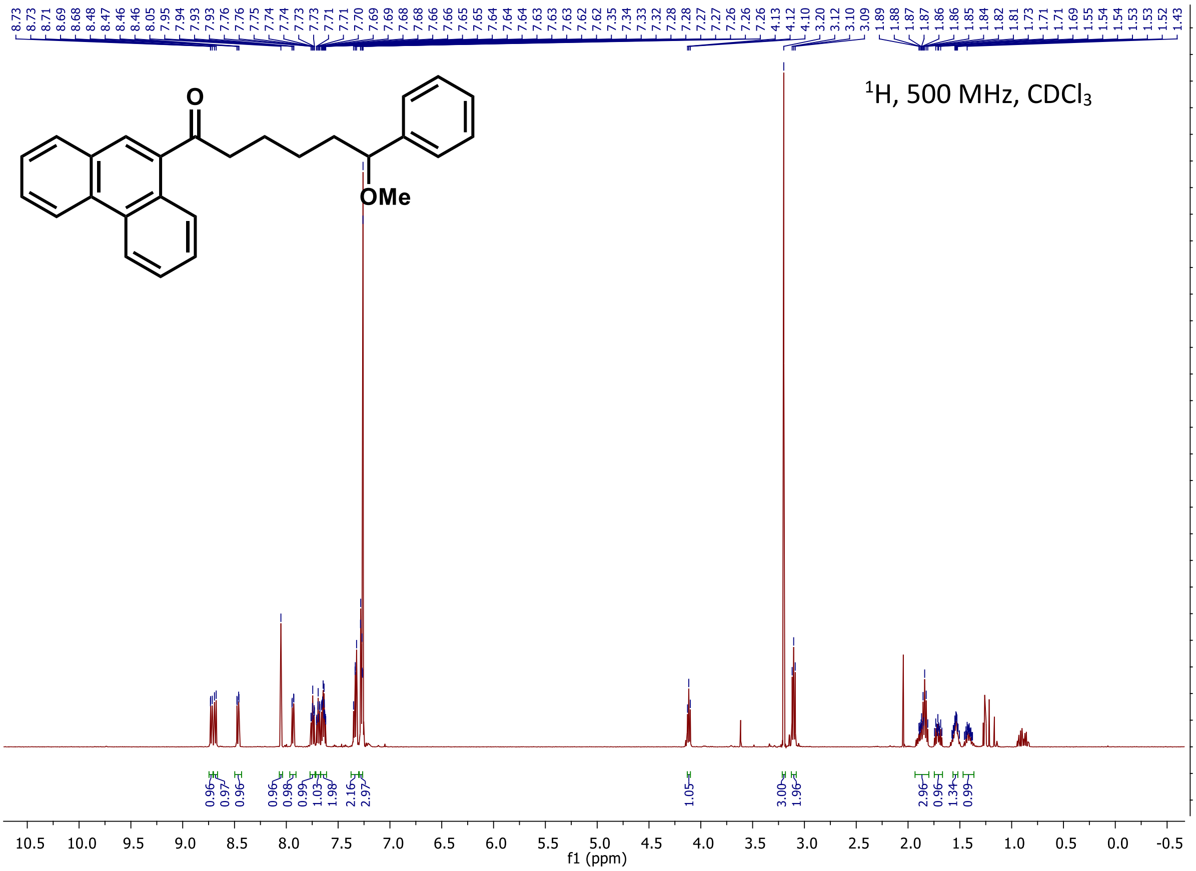

^{1}H, 500 MHz, CDCl$_3$
O
OMe
f1 (ppm)

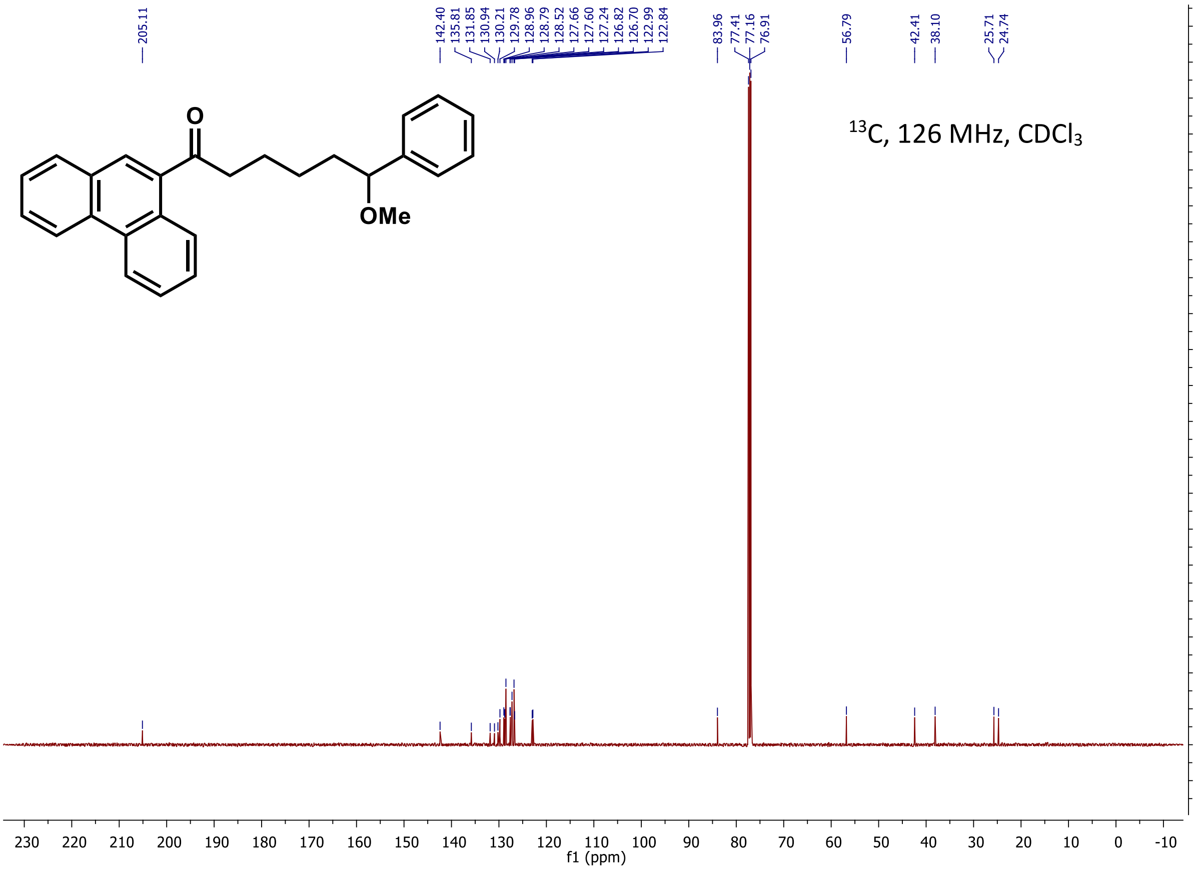

^{13}C, 126 MHz, CDCl$_3$
O
OMe
205.11
142.40
135.81
131.85
130.94
130.21
129.78
128.96
128.79
128.52
127.66
127.60
127.24
126.82
126.70
122.99
122.84
83.96
77.41
77.16
76.91
56.79
42.41
38.10
25.71
24.74
f1 (ppm)

(17)

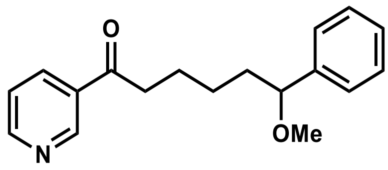

Prepared according to the General Procedure Y passing 4.5 *F* of charge using **S17** (76.0 mg, 0.30 mmol, 1 equiv.), *n*-Bu_4NPF_6 (232 mg, 0.60 mmol, 2 equiv.), dichloromethane (4.5 mL) and MeOH (1.5 mL). The yield was determined by crude ^{1}H NMR using 1,3,5-trimethylbenzene (42 µL, 1 equiv.) as an internal standard: 34%. Purification by flash column chromatography (eluent = 20 to 50% EtOAc in hexanes, silica gel) to afford product as a colourless oil (15.8 mg, 19% yield).

R_f = 0.15 (eluent = 35% EtOAc in hexanes); **ν_{max} / cm^{-1}** (thin film) 2930, 1686, 1585, 1452, 1360, 1103; **^{1}H NMR (500 MHz, $CDCl_3$)** δ_H: 1.32-1.42 (1H, m), 1.45-1.56 (1H, m), 1.64-1.72 (1H, m), 1.73-1.80 (2H, m), 1.82-1.90 (1H, m), 2.95 (2H, dd, *J* 7.9, 6.7 Hz), 3.20 (3H, s), 4.10 (1H, dd, *J* 7.4, 5.9 Hz), 7.27-7.31 (3H, m), 7.33-7.37 (2H, m), 7.42 (1H, dd, *J* 7.8, 4.9 Hz), 8.22 (1H, d, *J* 7.9 Hz), 8.74-8.80 (1H, m), 9.14 (1H, s); **^{13}C NMR (126 MHz, $CDCl_3$)** δ_C: 24.0, 25.6, 38.1, 39.0, 56.8, 83.9, 123.9, 126.8, 127.7, 128.5, 132.4, 135.7, 142.3, 149.6, 153.3, 199.0; **HRMS (CI$^+$)** [$C_{18}H_{21}NO_2$] requires $[M+H]^+$ 284.1651, found 284.1643 (-2.8 ppm).

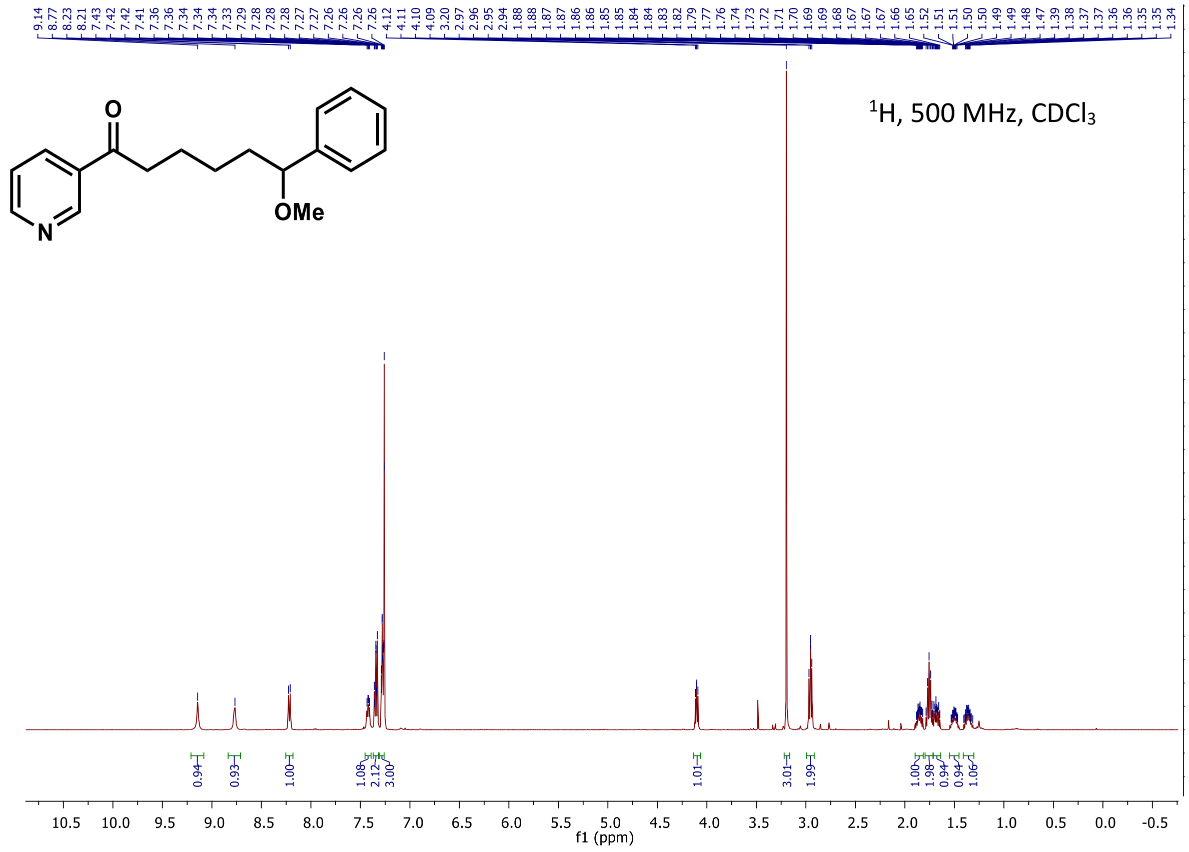

OMe
N
O
¹H, 500 MHz, CDCl₃
f1 (ppm)

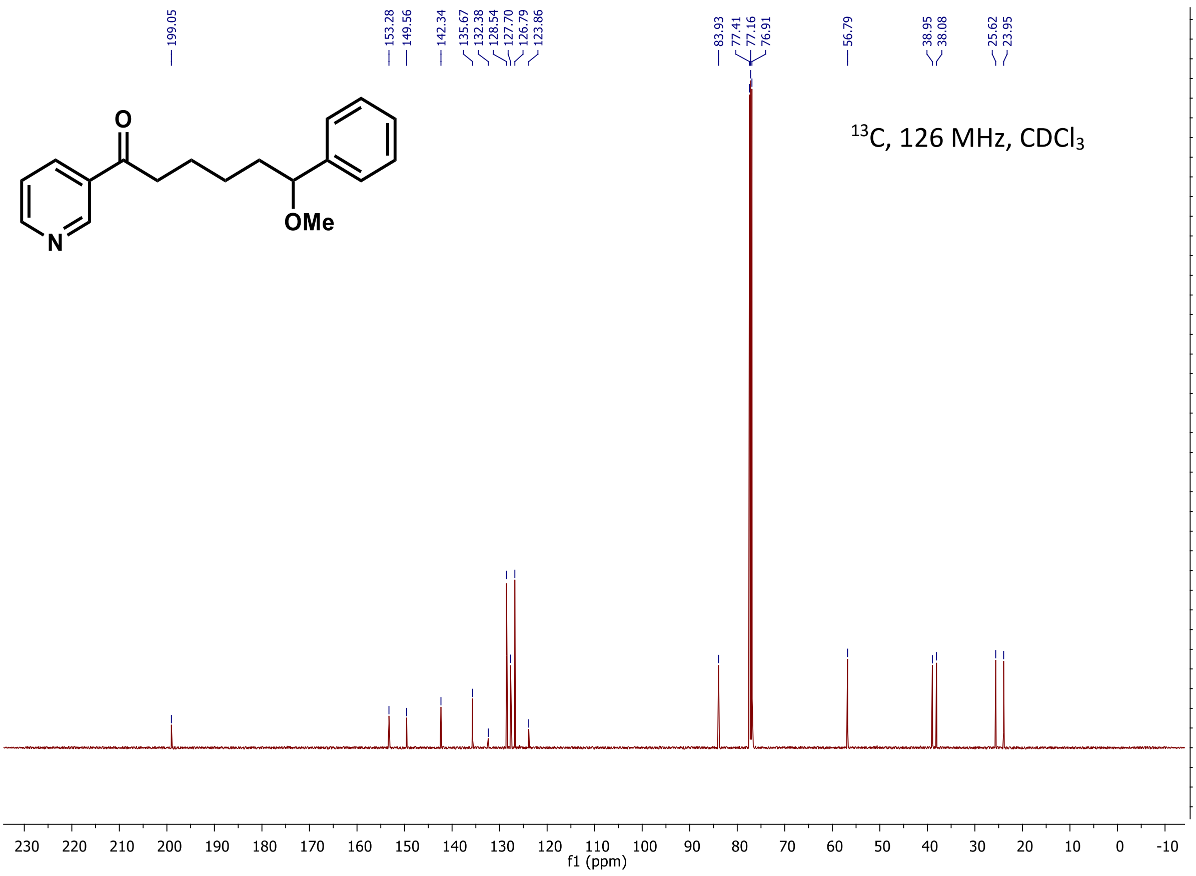

OMe
N
O
¹³C, 126 MHz, CDCl₃
f1 (ppm)

(18)

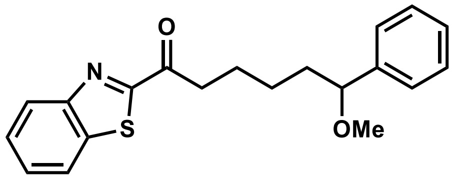

Prepared according to the General Procedure Y using **S18** (93.0 mg, 0.30 mmol, 1 equiv.), *n*-Bu_4NPF_6 (232 mg, 0.60 mmol, 2 equiv.), dichloromethane (4.5 mL) and MeOH (1.5 mL). The yield was determined by crude ^{1}H NMR using 1,3,5-trimethylbenzene (42 µL, 1 equiv.) as an internal standard: 39%. Purification by flash column chromatography (eluent = 5 to 10% EtOAc in hexanes, silica gel) to afford product as a yellow oil (37.8 mg, 37% yield).

Mp.: 54-56 °C; $\mathbf{R_f}$ = 0.41 (eluent = 20% EtOAc in hexanes); $\mathbf{v_{max}}$ **/ cm^{-1}** (thin film) 2932, 1684, 1487, 1451, 1105; **^{1}H NMR (500 MHz, $CDCl_3$)** δ_H: 1.32-1.45 (1H, m), 1.49-1.56 (1H, m), 1.66-1.75 (1H, m), 1.80-1.93 (3H, m), 3.20 (3H, s), 3.23-3.27 (2H, m), 4.11 (1H, dd, *J* 7.3, 6.0 Hz), 7.26-7.29 (3H, m), 7.31-7.37 (2H, m), 7.49-7.55 (1H, m), 7.55-7.59 (1H, m), 7.97 (1H, d, *J* 7.3 Hz), 8.17 (1H, d, *J* 7.5 Hz); **^{13}C NMR (126 MHz, $CDCl_3$)** δ_C: 24.0, 25.6, 38.0, 38.7, 56.8, 84.0, 122.6, 125.6, 126.8, 127.1, 127.7, 127.8, 128.5, 137.4, 142.4, 153.7, 166.7, 195.6; **HRMS (CI$^+$)** [$C_{20}H_{21}NO_2S$] requires [M-MeOH+H]$^+$ 308.1109, found 308.1103 (- 1.9 ppm). Elimination of methanol observed upon ionisation.

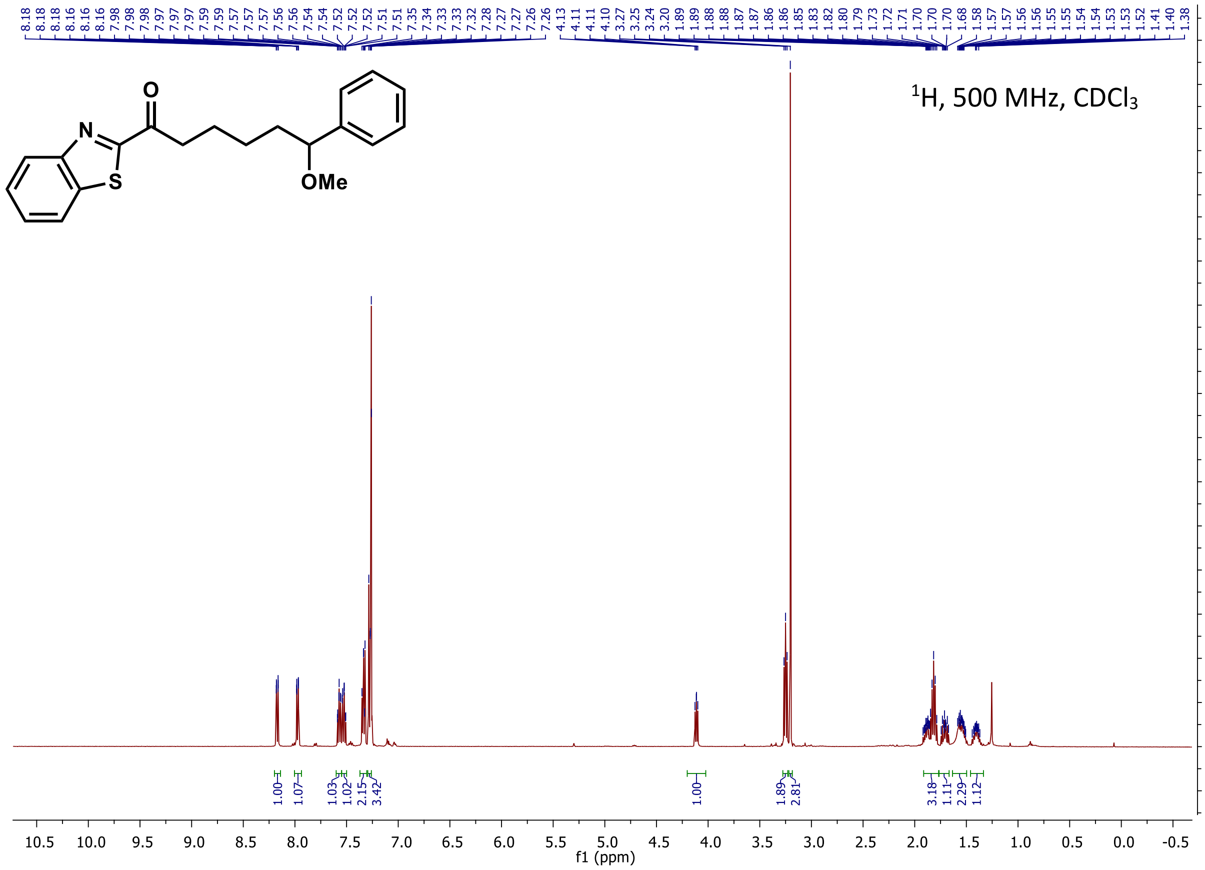
1H, 500 MHz, CDCl3
OMe
f1 (ppm)

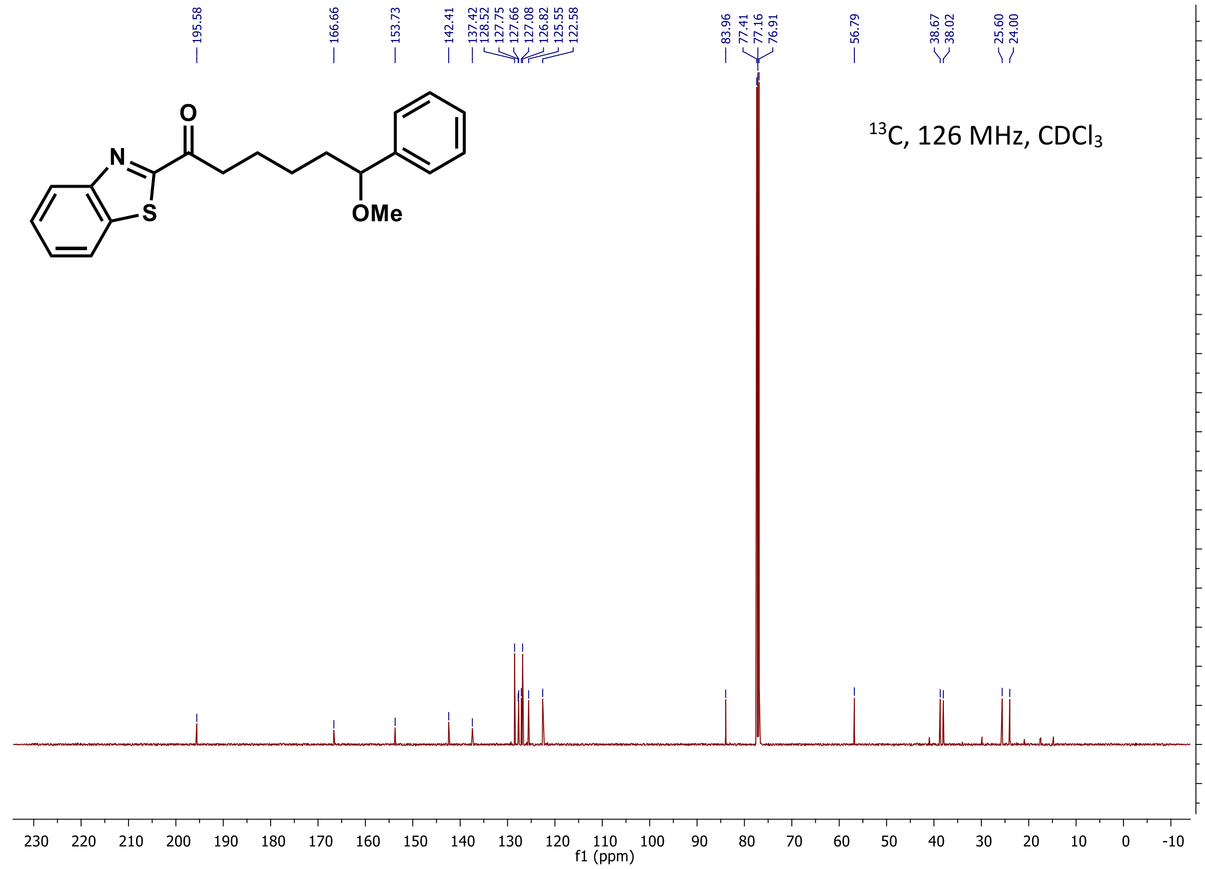
13C, 126 MHz, CDCl3
OMe
195.58
166.66
153.73
142.41
137.42
128.52
127.75
127.66
127.08
126.82
125.55
122.58
83.96
77.41
77.16
76.91
56.79
38.67
38.02
25.60
24.00
f1 (ppm)

(19)

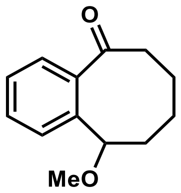

Prepared according to the General Procedure Y using **S19** (52.0 mg, 0.30 mmol, 1 equiv.), *n*-Bu_4NPF_6 (232 mg, 0.60 mmol, 2 equiv.), dichloromethane (4.5 mL) and MeOH (1.5 mL). The yield was determined by crude ^{1}H NMR using 1,3,5-trimethylbenzene (42 µL, 1 equiv.) as an internal standard: 75%. Purification by flash column chromatography (eluent = 5-10% EtOAc in hexanes, silica gel) to afford product as a colourless oil (43.1 mg, 70% yield).

R_f = 0.31 (eluent = 20% EtOAc in hexanes); **ν_{max} / cm^{-1}** (thin film) 2928, 1686, 1597, 1446, 1254, 1092; **^{1}H NMR (500 MHz, $CDCl_3$)** δ_H: 1.53-1.63 (2H, m), 1.65-1.73 (1H, m), 1.77-1.86 (1H, m), 1.90-2.01 (2H, m), 2.66-2.87 (2H, m), 3.21 (3H, s), 4.55 (1H, dd, *J* 7.8, 4.2 Hz), 7.29-7.33 (2H, m), 7.34 (1H, d, *J* 7.6 Hz), 7.40-7.48 (1H, m); **^{13}C NMR (126 MHz, $CDCl_3$)** δ_C: 23.4, 24.3, 35.9, 46.6, 57.0, 81.4, 126.1, 126.4, 127.7, 130.6, 139.4, 139.8, 209.4; **HRMS (CI$^+$)** [$C_{13}H_{16}O_2$] requires [M]$^+$ 204.1150, found 204.1145 (-1.9 ppm).

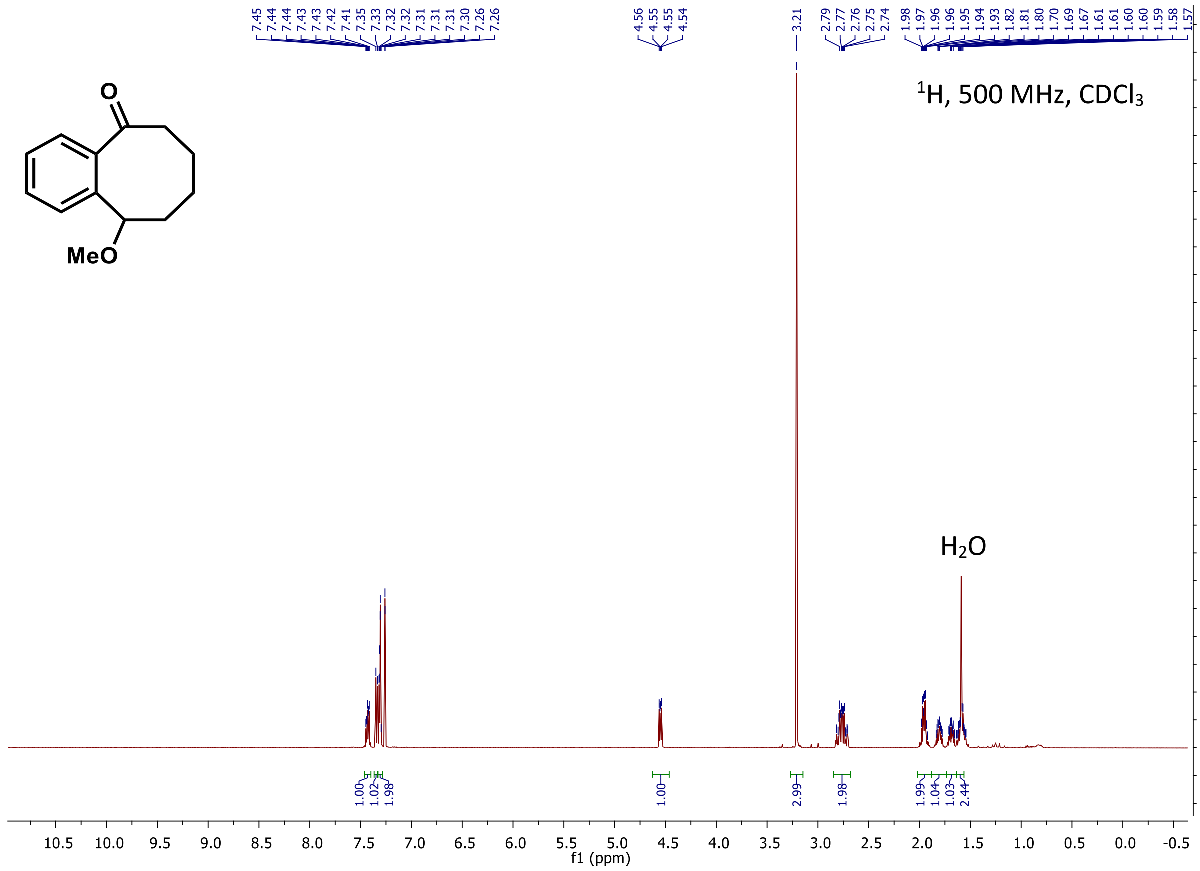
7.45 7.44 7.44 7.43 7.43 7.42 7.41 7.35 7.33 7.32 7.32 7.31 7.31 7.31 7.30 7.26 7.26
4.56 4.55 4.55 4.54
3.21
2.79 2.77 2.76 2.75 2.74
1.98 1.97 1.96 1.96 1.95 1.94 1.93 1.82 1.81 1.80 1.70 1.69 1.67 1.61 1.61 1.60 1.60 1.59 1.58 1.57
O
MeO
1H, 500 MHz, CDCl3
H2O
1.00 1.02 1.98
1.00
2.99
1.98
1.99 1.04 1.03 2.44
f1 (ppm)

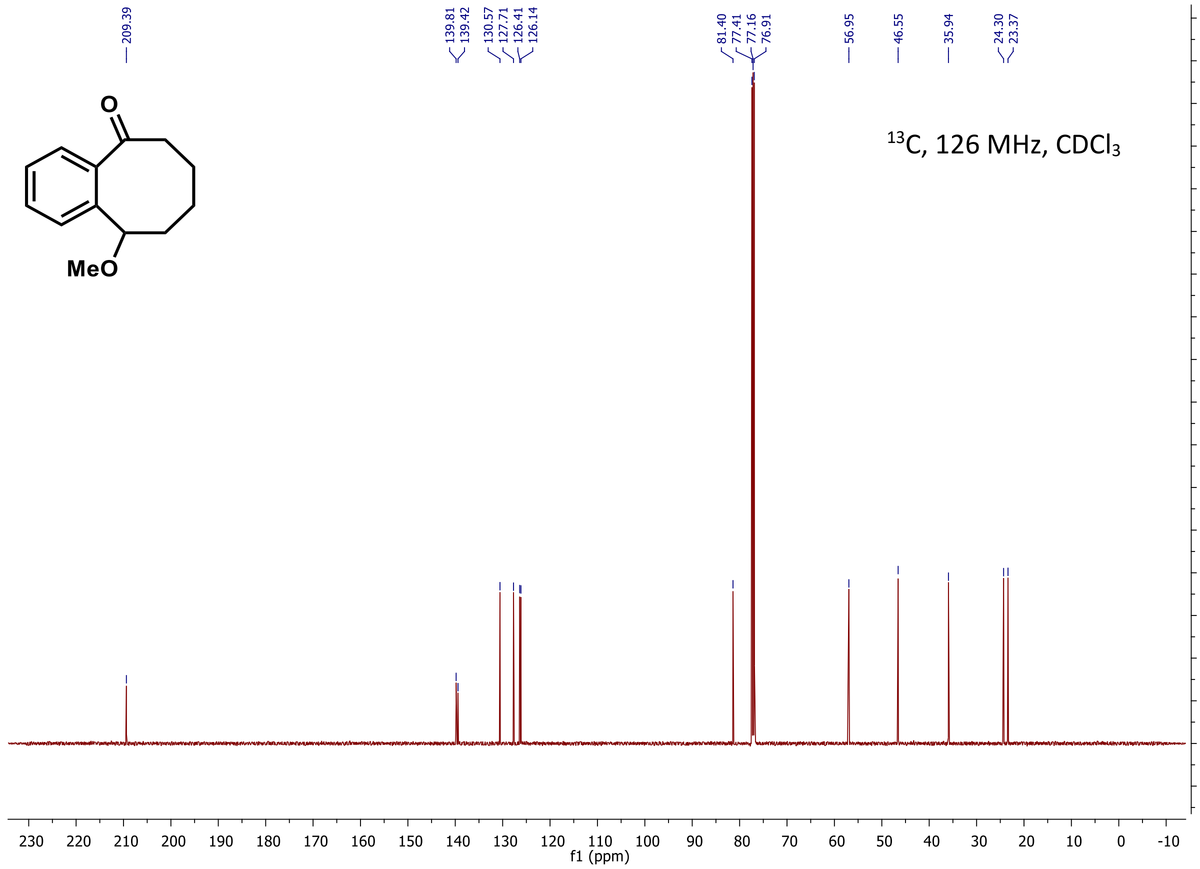
209.39
139.81 139.42 130.57 127.71 126.41 126.14
81.40 77.41 77.16 76.91
56.95
46.55
35.94
24.30 23.37
O
MeO
13C, 126 MHz, CDCl3
f1 (ppm)

(20)

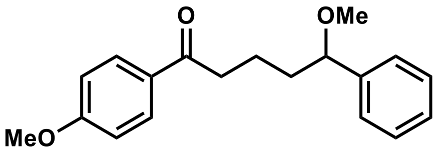

Prepared according to the General Procedure Y **S20** (80.5 mg, 0.30 mmol, 1 equiv.), *n*-Bu_4NPF_6 (232 mg, 0.60 mmol, 2 equiv.), dichloromethane (4.5 mL) and MeOH (1.5 mL). The yield was determined by crude ^{1}H NMR using 1,3,5-trimethylbenzene (42 µL, 1 equiv.) as an internal standard: 53%. Purification by flash column chromatography (eluent = 5-20% EtOAc in hexanes, silica gel) to afford product as a yellow oil (36.3 mg, 41% yield).

Mp.: 44-46 °C; **R_f** = 0.34 (eluent = 20% EtOAc in hexanes); **ν_{max} / cm^{-1}** (thin film) 2935, 1672, 1599, 1510, 1454, 1361, 1256, 1169, 1103; **^{1}H NMR (400 MHz, $CDCl_3$)** δ_H: 1.58-1.78 (2H, m), 1.78-2.02 (2H, m), 2.77-3.04 (2H, m), 3.20 (3H, s), 3.86 (3H, s), 4.13 (1H, dd, *J* 7.2, 5.4 Hz), 6.91 (2H, d, *J* 8.7 Hz), 7.19-7.47 (5H, m), 7.91 (2H, d, *J* 8.7 Hz); **^{13}C NMR (101 MHz, $CDCl_3$)** δ_C: 21.2, 37.9, 38.2, 55.6, 56.8, 84.1, 113.8, 126.8, 127.7, 128.5, 130.3, 130.4, 142.3, 163.5, 198.9; **HRMS (ES$^+$)** [$C_{19}H_{22}O_3$] requires [M+Na]$^+$ 321.1467, found 321.1461 (-1.9 ppm).

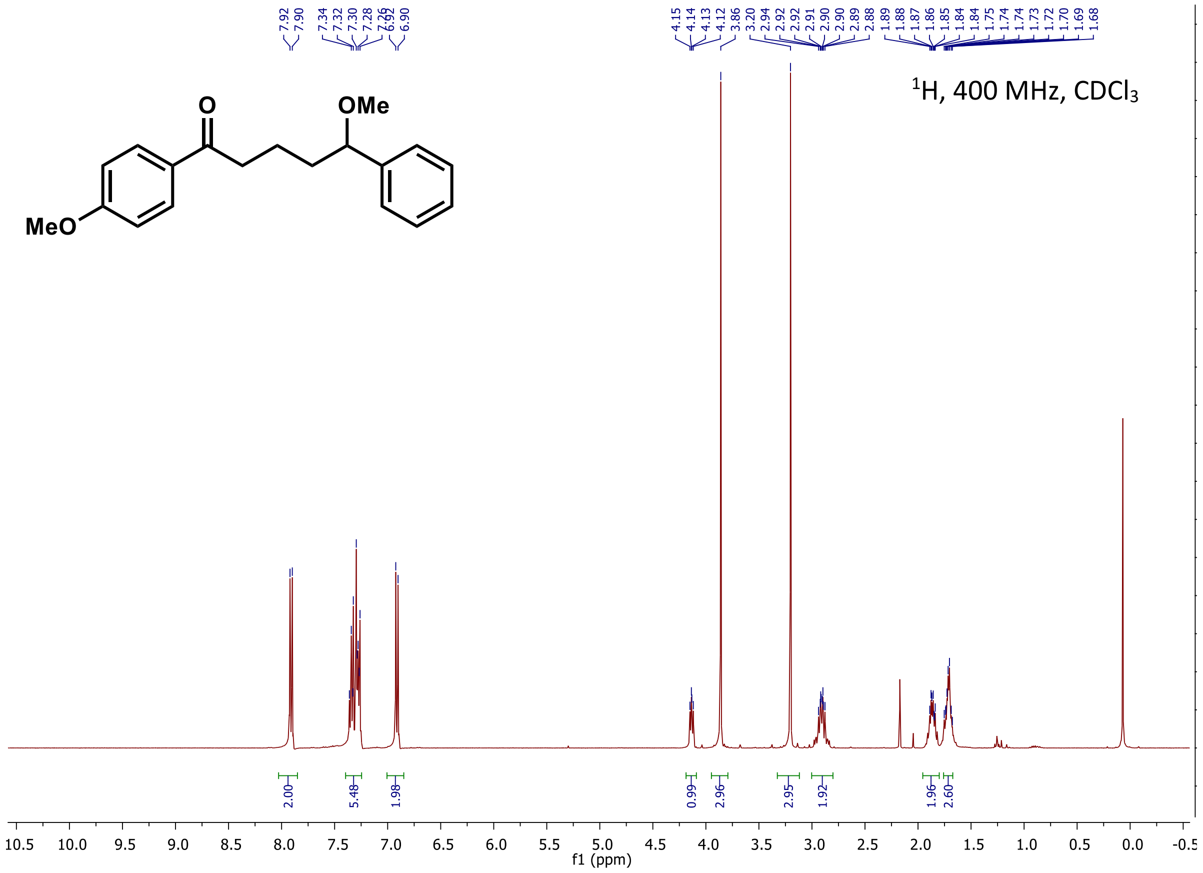

7.92
7.90
7.34
7.32
7.30
7.28
7.26
6.92
6.90
4.15
4.14
4.13
4.12
3.86
3.20
2.94
2.92
2.92
2.91
2.90
2.90
2.89
2.88
1.89
1.88
1.87
1.86
1.85
1.84
1.84
1.75
1.74
1.74
1.73
1.72
1.70
1.69
1.68
O
OMe
MeO
^{1}H, 400 MHz, CDCl$_3$
2.00
5.48
1.98
0.99
2.96
2.95
1.92
1.96
2.60
f1 (ppm)

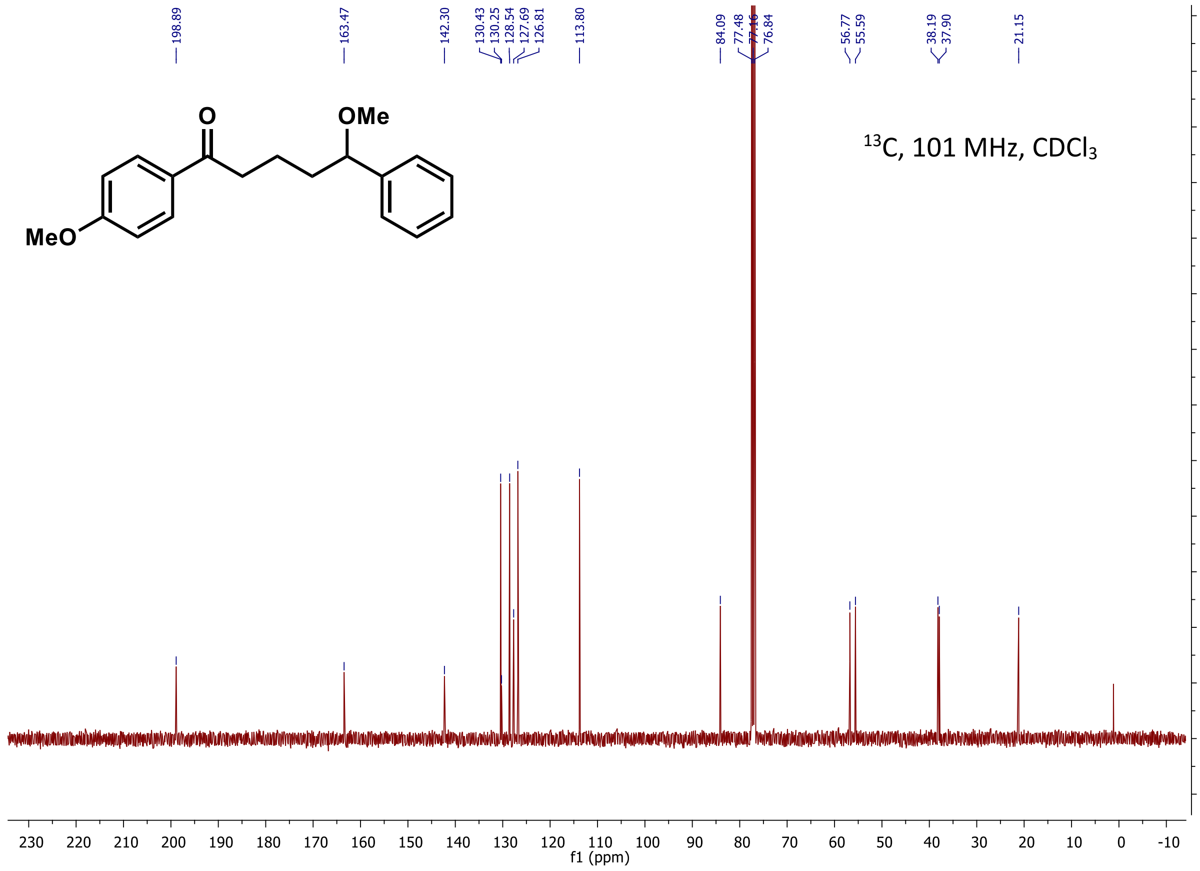

198.89
163.47
142.30
130.43
130.25
128.54
127.69
126.81
113.80
84.09
77.48
77.16
76.84
56.77
55.59
38.19
37.90
21.15
O
OMe
MeO
^{13}C, 101 MHz, CDCl$_3$
f1 (ppm)

(21)

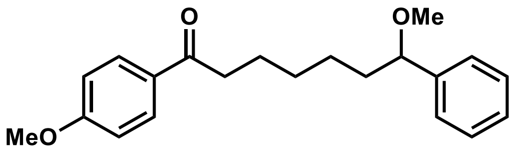

Prepared according to the General Procedure Y using **S21** (89.0 mg, 0.30 mmol, 1 equiv.), *n*-Bu_4NPF_6 (232 mg, 0.60 mmol, 2 equiv.), dichloromethane (4.5 mL) and MeOH (1.5 mL). The yield was determined by crude ^{1}H NMR using 1,3,5-trimethylbenzene (42 µL, 1 equiv.) as an internal standard: 77%. Purification by flash column chromatography (eluent = 5-10% EtOAc in hexanes, silica gel) to afford product as a colourless oil (65.6 mg, 67% yield).

Mp.: 47-49 °C; $\mathbf{R_f}$ = 0.39 (eluent = 20% EtOAc in hexanes); $\mathbf{\nu_{max}}$ **/ cm^{-1}** (thin film) 2933, 2850, 1670, 1601, 1578, 1508, 1450, 1346, 1222, 1177, 1107; **^{1}H NMR (400 MHz, $CDCl_3$)** δ_H: 1.16-1.45 (4H, m), 1.56-1.77 (3H, m), 1.75-1.90 (1H, m), 2.87 (2H, t, *J* 7.4 Hz), 3.20 (3H, s), 3.87 (3H, s), 4.08 (1H, app. t, *J* 7.8 Hz), 6.92 (2H, d, *J* 8.5 Hz), 7.23-7.30 (3H, m), 7.31-7.38 (2H, m), 7.92 (2H, d, *J* 8.5 Hz); **^{13}C NMR (101 MHz, $CDCl_3$)** δ_C: 24.6, 25.8, 29.4, 38.1, 38.3, 55.6, 56.8, 84.2, 113.8, 126.8, 127.6, 128.5, 130.3, 130.4, 142.6, 163.5, 199.2; **HRMS (ES$^+$)** [$C_{21}H_{26}O_3$] requires [M+Na]$^+$ 349.1780, found 349.1779 (- 0.3 ppm).

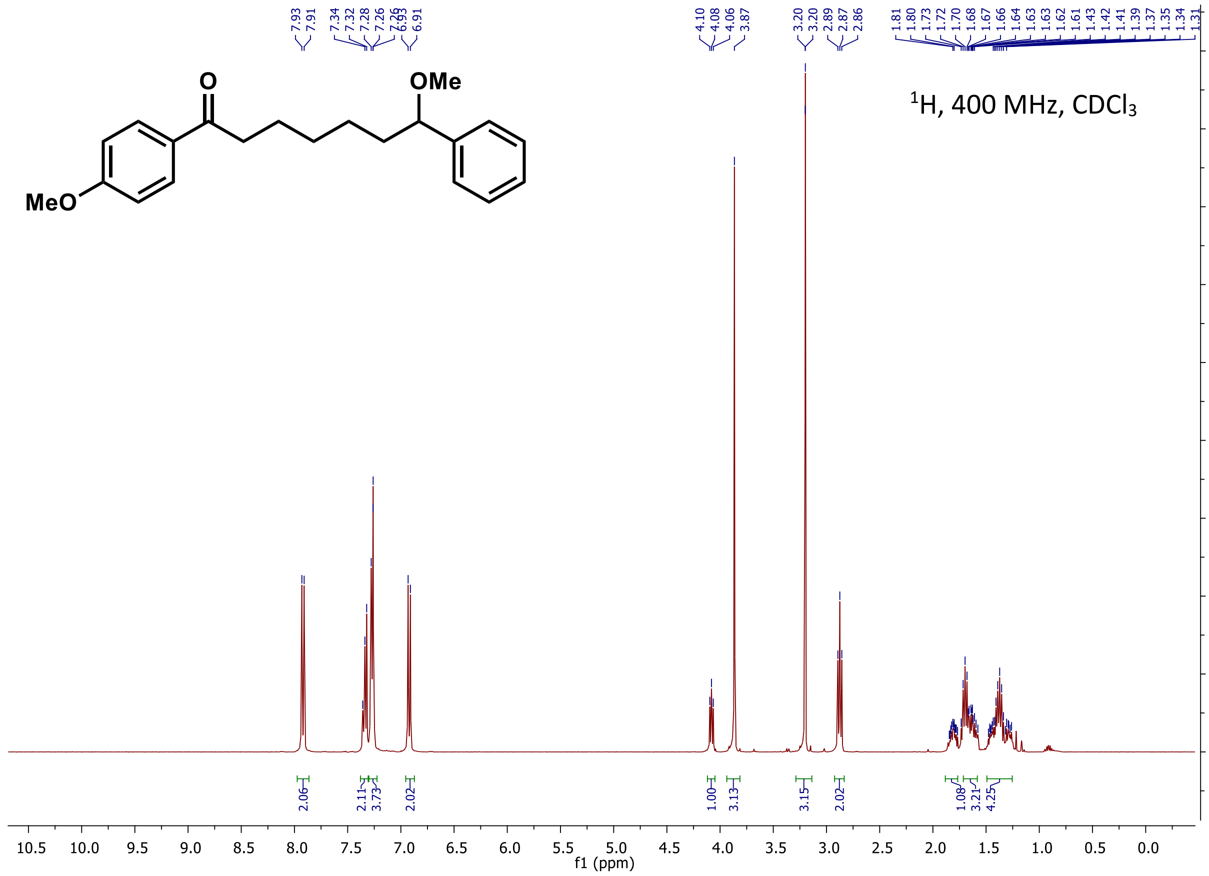
1H, 400 MHz, CDCl3
MeO
O
OMe
f1 (ppm)

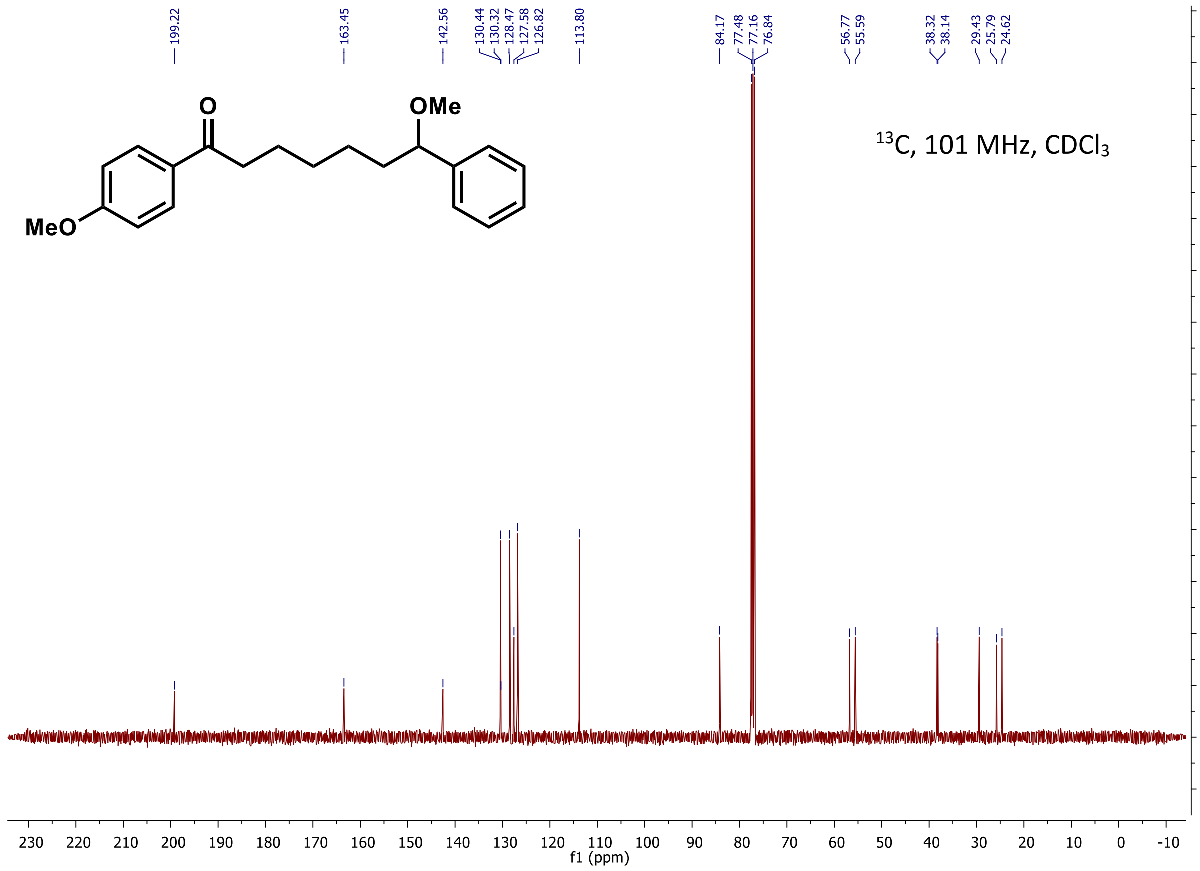
13C, 101 MHz, CDCl3
MeO
O
OMe
f1 (ppm)

(22)

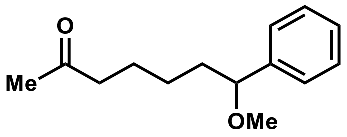

Prepared according to the General Procedure X using **S22** (57.0 mg, 0.30 mmol, 1 equiv.), *n*-Bu_4NPF_6 (232 mg, 0.60 mmol, 2 equiv.), dichloromethane (4.5 mL) and MeOH (1.5 mL). The yield was determined by crude ^{1}H NMR using 1,3,5-trimethylbenzene (42 µL, 1 equiv.) as an internal standard: 45%. Purification by flash column chromatography (eluent = 5 to 15% EtOAc in hexanes, silica gel) to afford product as a colourless oil (27.0 mg, 41% yield).

R_f = 0.20 (eluent = 15% EtOAc in hexanes); **ν_{max} / cm^{-1}** (thin film) 2810, 1711, 1452,1356, 908; **^{1}H NMR (500 MHz, $CDCl_3$)** δ_H: 1.20-1.30 (1H, m), 1.33-1.44 (1H, m), 1.52-1.59 (2H, m), 1.61-1.68 (1H, m), 1.75-1.80 (1H, m), 2.10 (3H, s), 2.35-2.44 (2H, m), 3.19 (3H, s), 4.07 (1H, dd, *J* 7.4, 5.9 Hz), 7.24-7.30 (3H, m), 7.31-7.36 (2H, m); **^{13}C NMR (126 MHz, $CDCl_3$)** δ_C: 23.8, 25.5, 30.0, 38.0, 43.8, 56.7, 83.9, 126.8, 127.6, 128.5, 142.4, 209.2; **HRMS (ES$^+$)** [$C_{14}H_{20}O_2$] requires [M+Na]$^+$ 243.1361, found 243.1362 (+ 0.4 ppm).

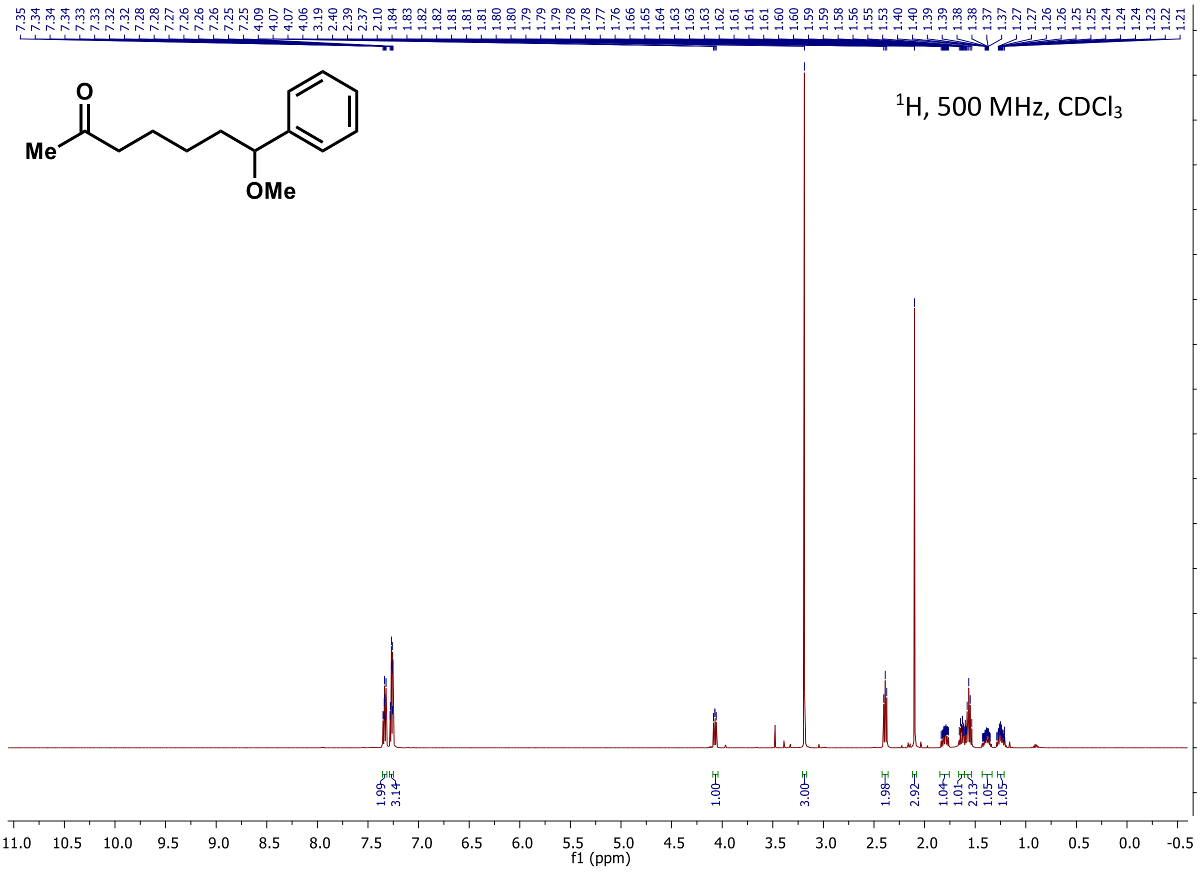

¹H, 500 MHz, CDCl₃
Me
O
OMe
f1 (ppm)

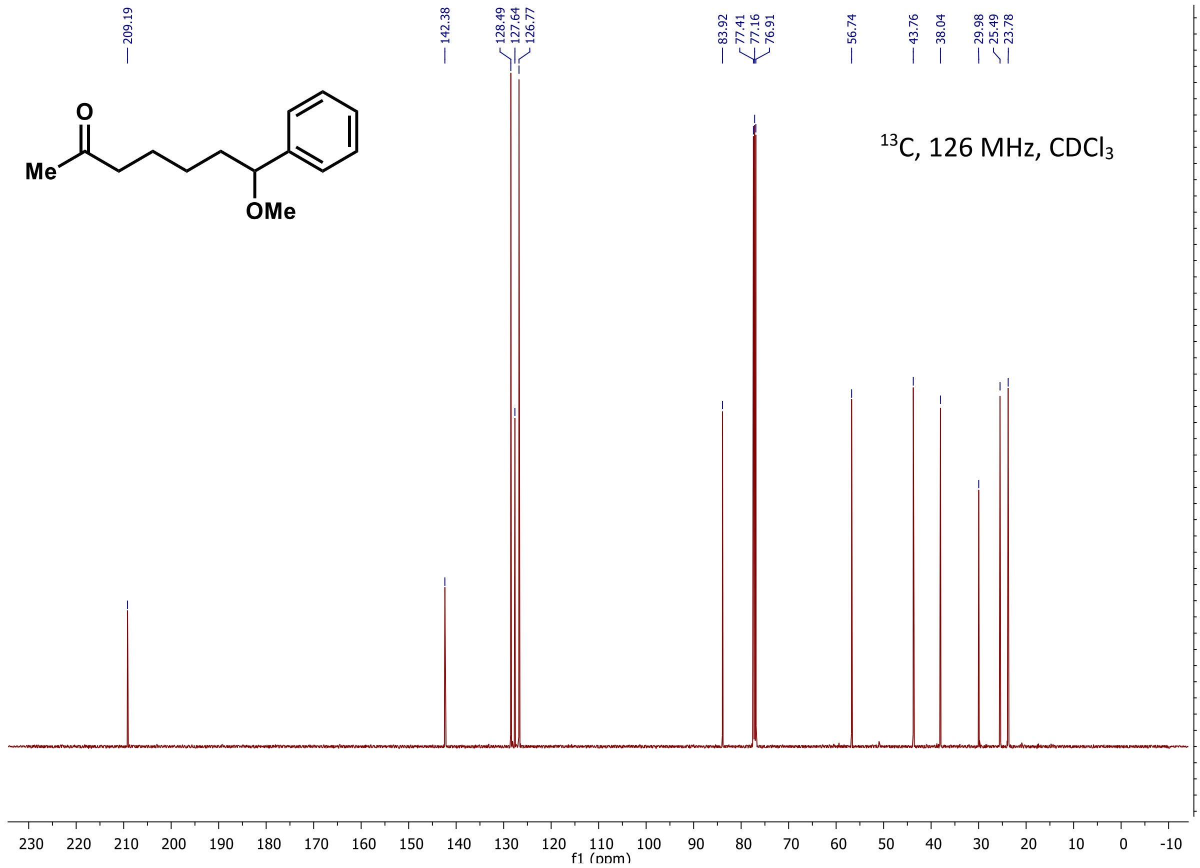

¹³C, 126 MHz, CDCl₃
Me
O
OMe
209.19
142.38
128.49
127.64
126.77
83.92
77.41
77.16
76.91
56.74
43.76
38.04
29.98
25.49
23.78
f1 (ppm)

(23)

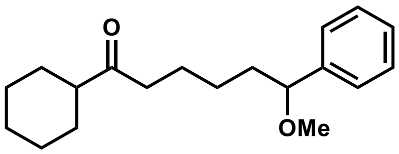

Prepared according to the General Procedure Y using **S23** (78.0 mg, 0.30 mmol, 1 equiv.), *n*-Bu_4NPF_6 (232 mg, 0.60 mmol, 2 equiv.), dichloromethane (4.5 mL) and MeOH (1.5 mL). The yield was determined by crude ^{1}H NMR using 1,3,5-trimethylbenzene (42 µL, 1 equiv.) as an internal standard: 54%. Purification by flash column chromatography (eluent = 5% EtOAc in hexanes, silica gel) to afford product as a colourless oil (36.4 mg, 42% yield).

R_f = 0.37 (eluent = 15% EtOAc in hexanes); **ν_{max} / cm^{-1}** (thin film) 2926, 2852, 1705, 1450, 1355, 1097; **^{1}H NMR (500 MHz, $CDCl_3$)** δ_H: 1.12-1.44 (7H, m), 1.50-1.59 (2H, m), 1.60-1.69 (2H, m), 1.71-1.86 (5H, m), 2.24-2.33 (1H, m), 2.35-2.44 (2H, m), 3.19 (3H, s), 4.07 (1H, dd, *J* 7.3, 6.0 Hz), 7.24-7.29 (3H, m), 7.32-7.36 (2H, m); **^{13}C NMR (126 MHz, $CDCl_3$)** δ_C: 23.7, 25.6, 25.8, 26.0, 28.6, 38.1, 40.6, 51.0, 56.8, 84.0, 126.8, 127.6, 128.5, 142.5, 214.4; **HRMS (CI$^+$)** [$C_{19}H_{28}O_2$] requires [M-CH_3]$^+$ 273.1855, found 273.1849 (-2.2 ppm). Loss of methyl group observed upon ionisation.

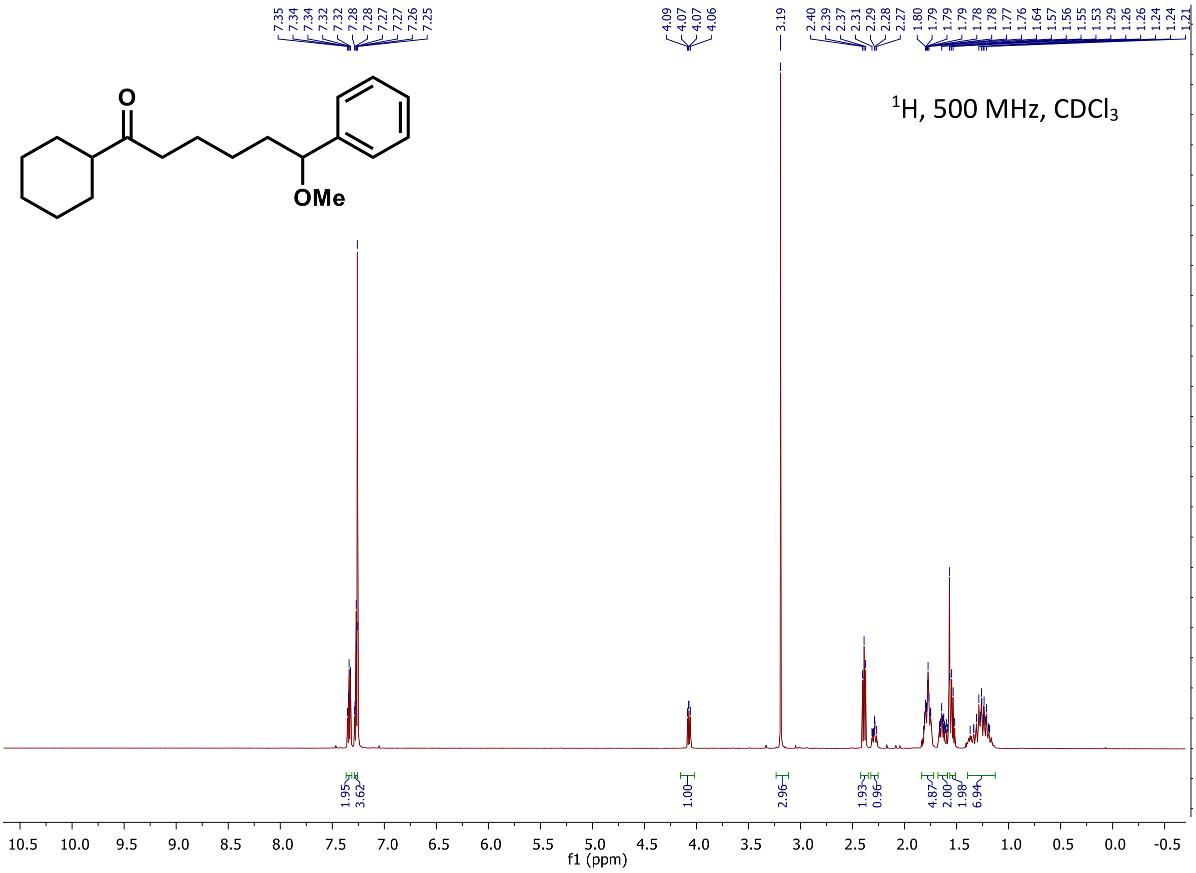
O
OMe
^{1}H, 500 MHz, CDCl$_3$
7.35 7.34 7.34 7.32 7.32 7.28 7.28 7.27 7.27 7.26 7.25
4.09 4.07 4.07 4.06
3.19
2.40 2.39 2.37 2.31 2.29 2.28 2.27 1.80 1.79 1.79 1.79 1.78 1.78 1.77 1.76 1.64 1.57 1.56 1.55 1.53 1.29 1.26 1.26 1.24 1.24 1.21
1.95 3.62 1.00 2.96 1.93 0.96 4.87 2.00 1.98 6.94
f1 (ppm)

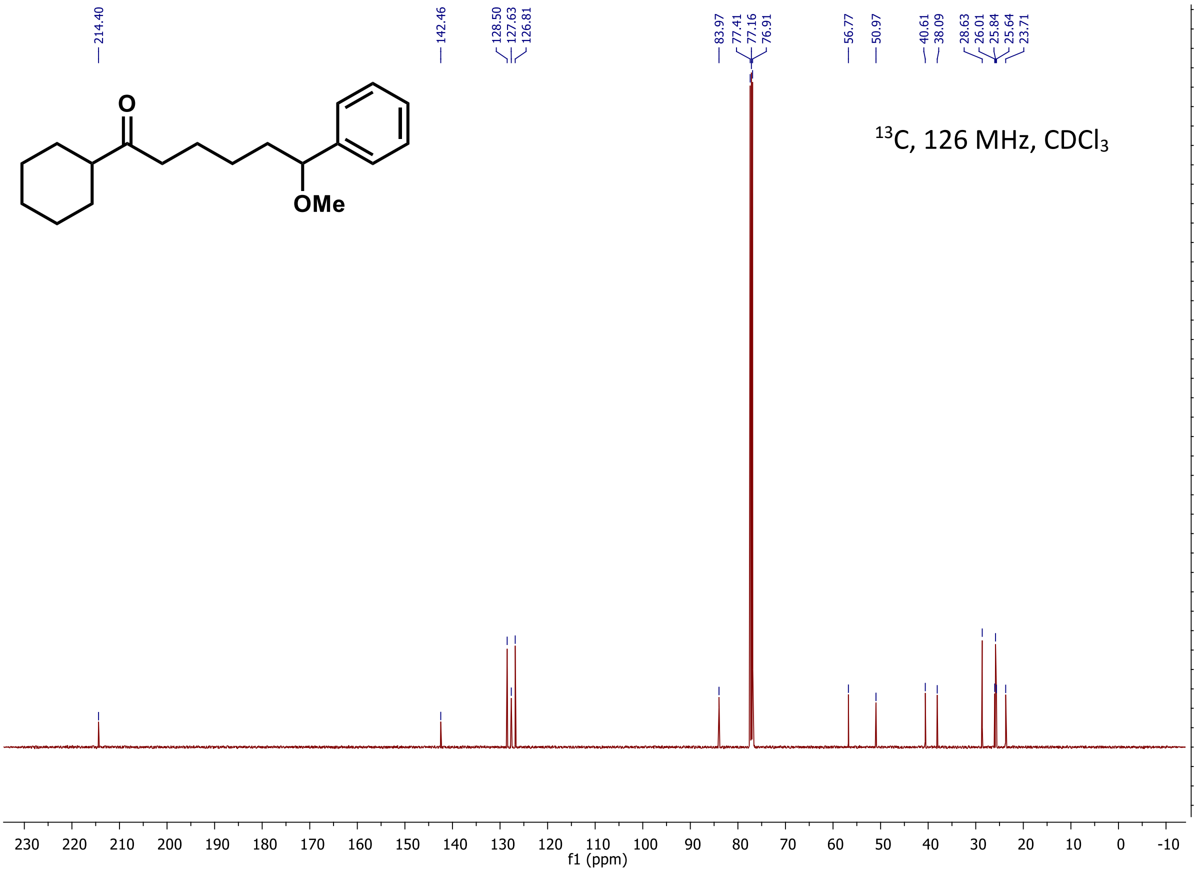
O
OMe
^{13}C, 126 MHz, CDCl$_3$
214.40
142.46
128.50 127.63 126.81
83.97 77.41 77.16 76.91
56.77 50.97
40.61 38.09
28.63 26.01 25.84 25.64 23.71
f1 (ppm)

(24)

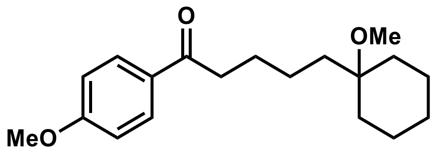

Prepared according to the General Procedure Y using **S24** (82.3 mg, 0.30 mmol, 1 equiv.), *n*-Bu_4NPF_6 (232 mg, 0.60 mmol, 2 equiv.), dichloromethane (4.5 mL) and MeOH (1.5 mL). The yield was determined by crude ^{1}H NMR using 1,3,5-trimethylbenzene (42 µL, 1 equiv.) as an internal standard: 53%. Purification by flash column chromatography (eluent = 5 to 10% EtOAc in hexanes, silica gel) to afford product as a colourless oil (36.4 mg, 40% yield).

R_f = 0.36 (eluent = 20% EtOAc in hexanes); **ν_{max} / cm^{-1}** (thin film) 2924, 1672, 1601, 1508, 1246, 1177; **^{1}H NMR (300 MHz, $CDCl_3$)** δ_H: 1.25-1.29 (3H, m), 1.35-1.55 (9H, m), 1.66-1.74 (4H, m), 2.90-2.95 (2H, m), 3.12 (3H, s), 3.87 (3H, s), 6.93 (2H, d, *J* 8.9 Hz), 7.94 (2H, d, *J* 8.9 Hz); **^{13}C NMR (101 MHz, $CDCl_3$)** δ_C: 22.0, 22.6, 25.3, 26.2, 34.2, 36.3, 38.5, 48.1, 55.6, 75.0, 113.8, 130.4, 130.4, 163.5, 199.2; **HRMS (CI$^+$)** [$C_{19}H_{28}O_3$] requires [M-MeOH+H]$^+$ 273.1855, found 273.1851 (- 1.9 ppm). Elimination of methanol observed upon ionisation.

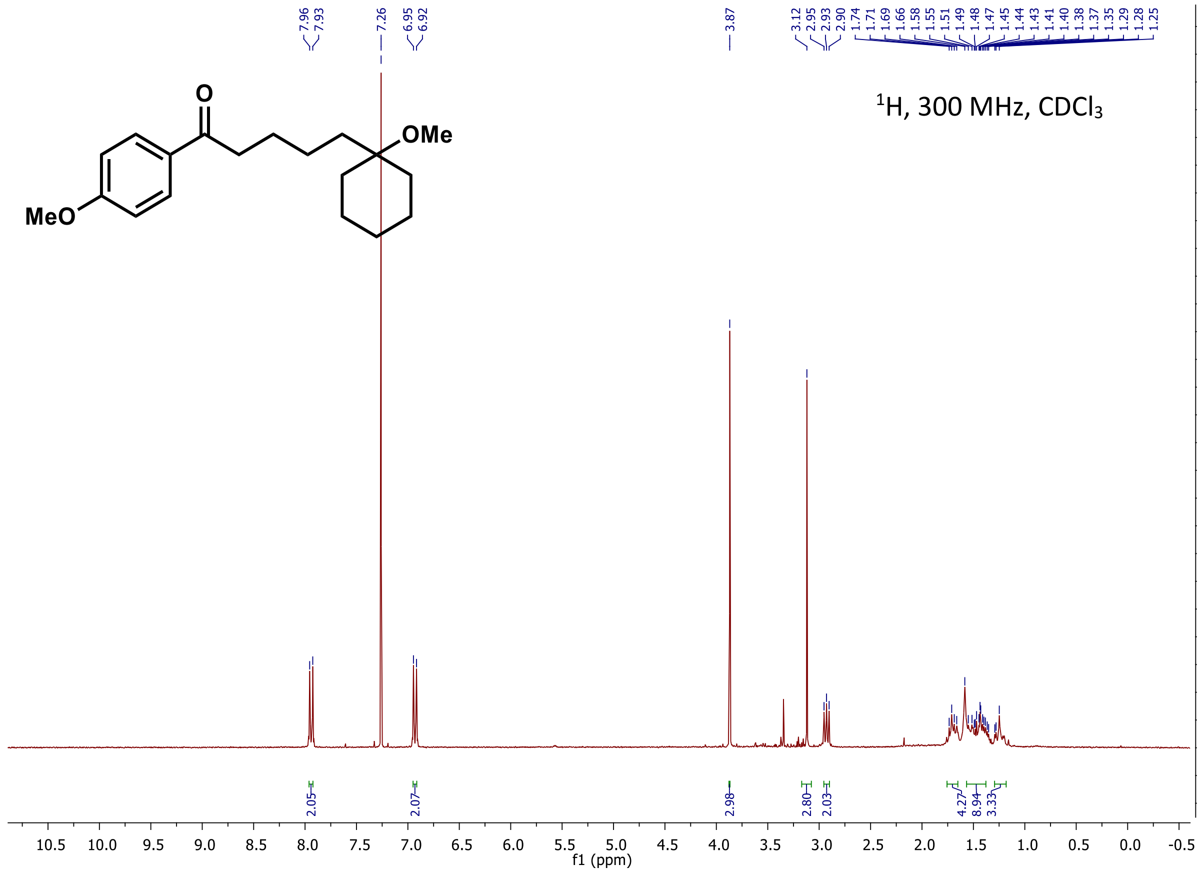

MeO
O
OMe
1H, 300 MHz, CDCl3
f1 (ppm)

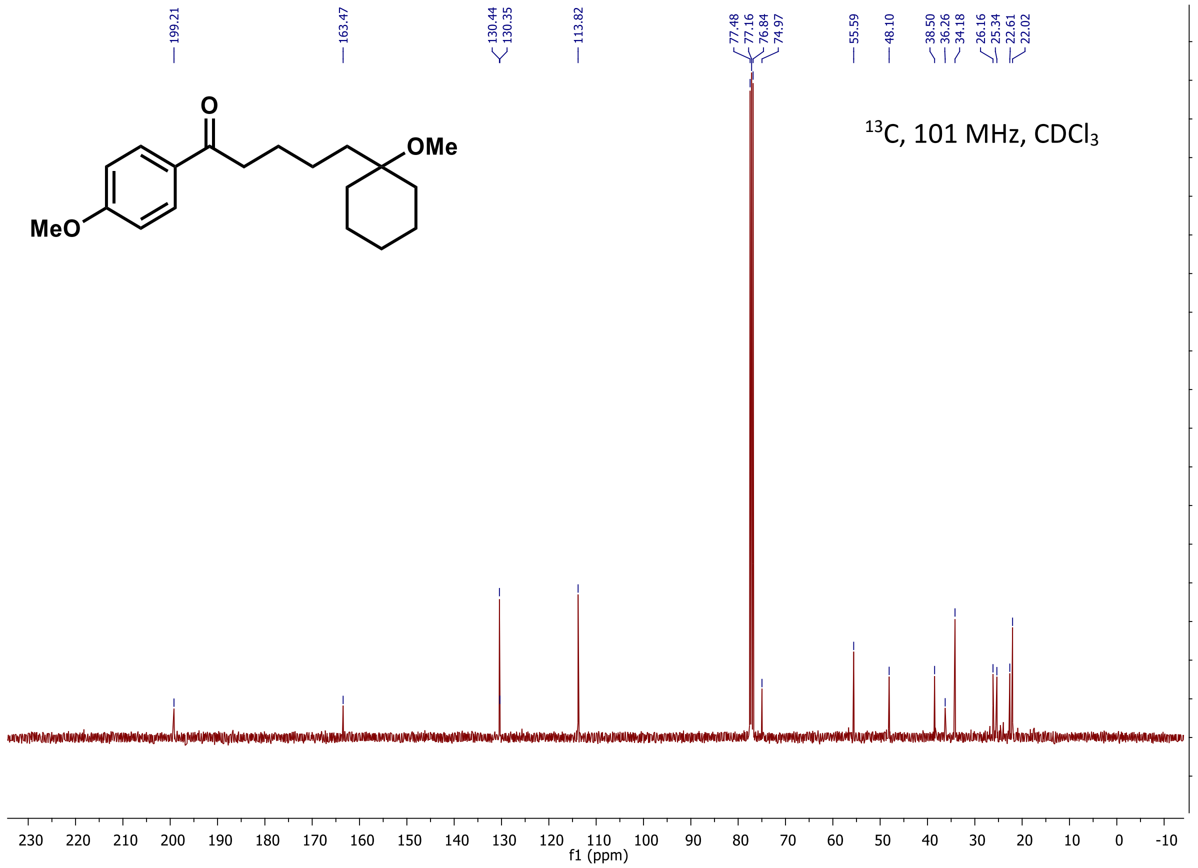

MeO
O
OMe
13C, 101 MHz, CDCl3
f1 (ppm)

(25)

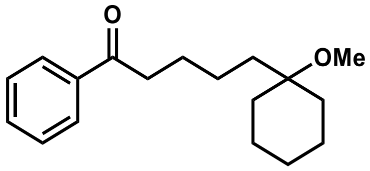

Prepared according to the General Procedure Y using **S25** (73.0 mg, 0.30 mmol, 1 equiv.), *n*-Bu_4NPF_6 (232 mg, 0.60 mmol, 2 equiv.), dichloromethane (4.5 mL) and MeOH (1.5 mL). The yield was determined by crude ^1H NMR using 1,3,5-trimethylbenzene (42 µL, 1 equiv.) as an internal standard: 58%. Purification by flash column chromatography (eluent = 0 to 8% EtOAc in hexanes, silica gel) to afford product as a colourless oil (35.4 mg, 43% yield).

R_f = 0.27 (eluent = 5% EtOAc in hexanes); **ν_{max} / cm^{-1}** (thin film) 2929, 1684, 1447, 1088; **^1H NMR (500 MHz, $CDCl_3$)** δ_H: 1.20-1.30 (3H, m), 1.34-1.58 (9H, m), 1.61-1.76 (4H, m), 2.95-3.01 (2H, m), 3.12 (3H, s), 7.42-7.50 (2H, m), 7.51-7.57 (1H, m), 7.93-7.97 (2H, m); **^{13}C NMR (126 MHz, $CDCl_3$)** δ_C: 22.0, 22.5, 25.1, 26.2, 34.2, 36.2, 38.8, 48.1, 75.0, 128.2, 128.7, 133.0, 137.2, 200.6; **HRMS (ES^+)** [$C_{18}H_{26}O_2$] requires $[M+Na]^+$ 297.1830, found 297.1832 (+ 0.7 ppm).

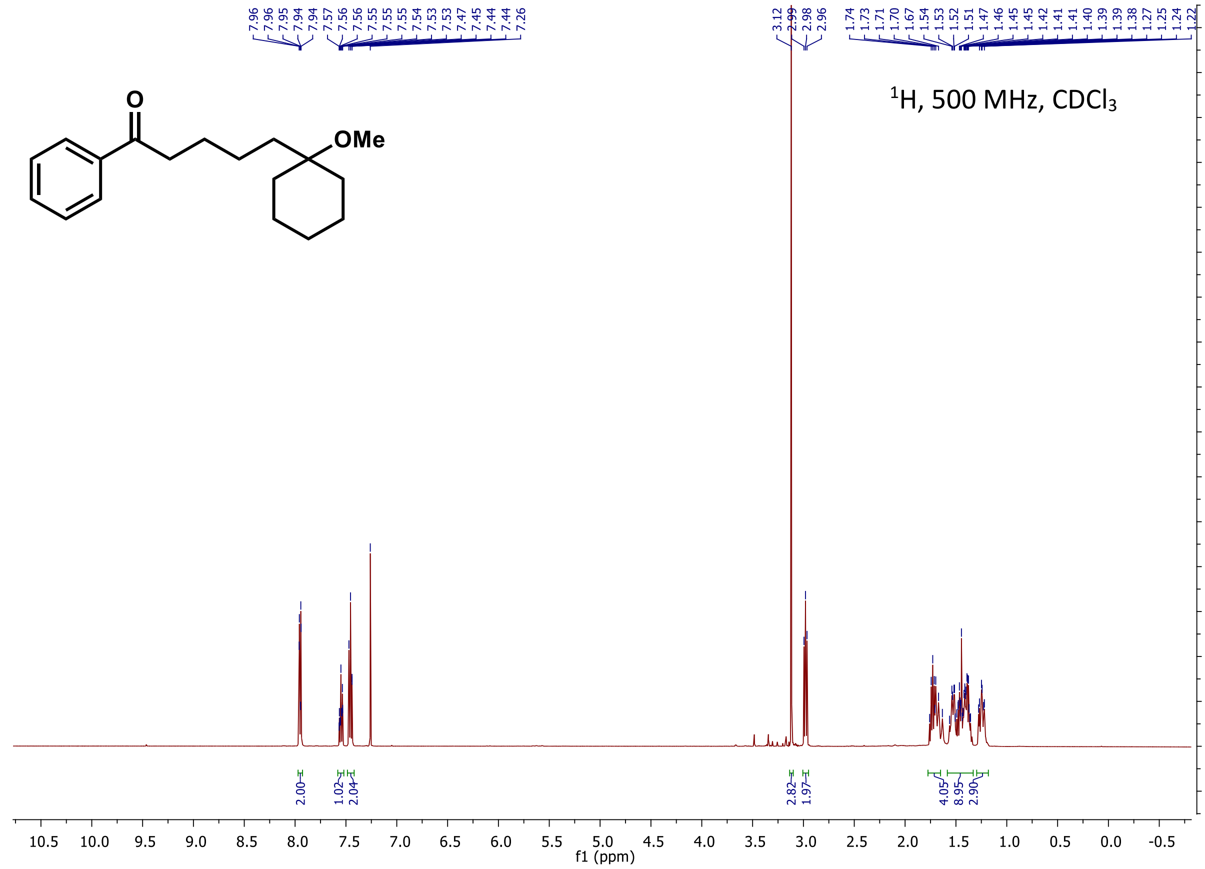

7.96 7.96 7.95 7.94 7.94 7.57 7.56 7.56 7.55 7.55 7.55 7.54 7.53 7.53 7.47 7.45 7.44 7.44 7.26
3.12 2.99 2.98 2.96
1.74 1.73 1.71 1.70 1.67 1.54 1.53 1.52 1.51 1.47 1.46 1.45 1.45 1.42 1.41 1.41 1.40 1.39 1.39 1.38 1.27 1.25 1.24 1.22
O
OMe
1H, 500 MHz, CDCl3
2.00 1.02 2.04 2.82 1.97 4.05 8.95 2.90
10.5 10.0 9.5 9.0 8.5 8.0 7.5 7.0 6.5 6.0 5.5 5.0 4.5 4.0 3.5 3.0 2.5 2.0 1.5 1.0 0.5 0.0 -0.5
f1 (ppm)

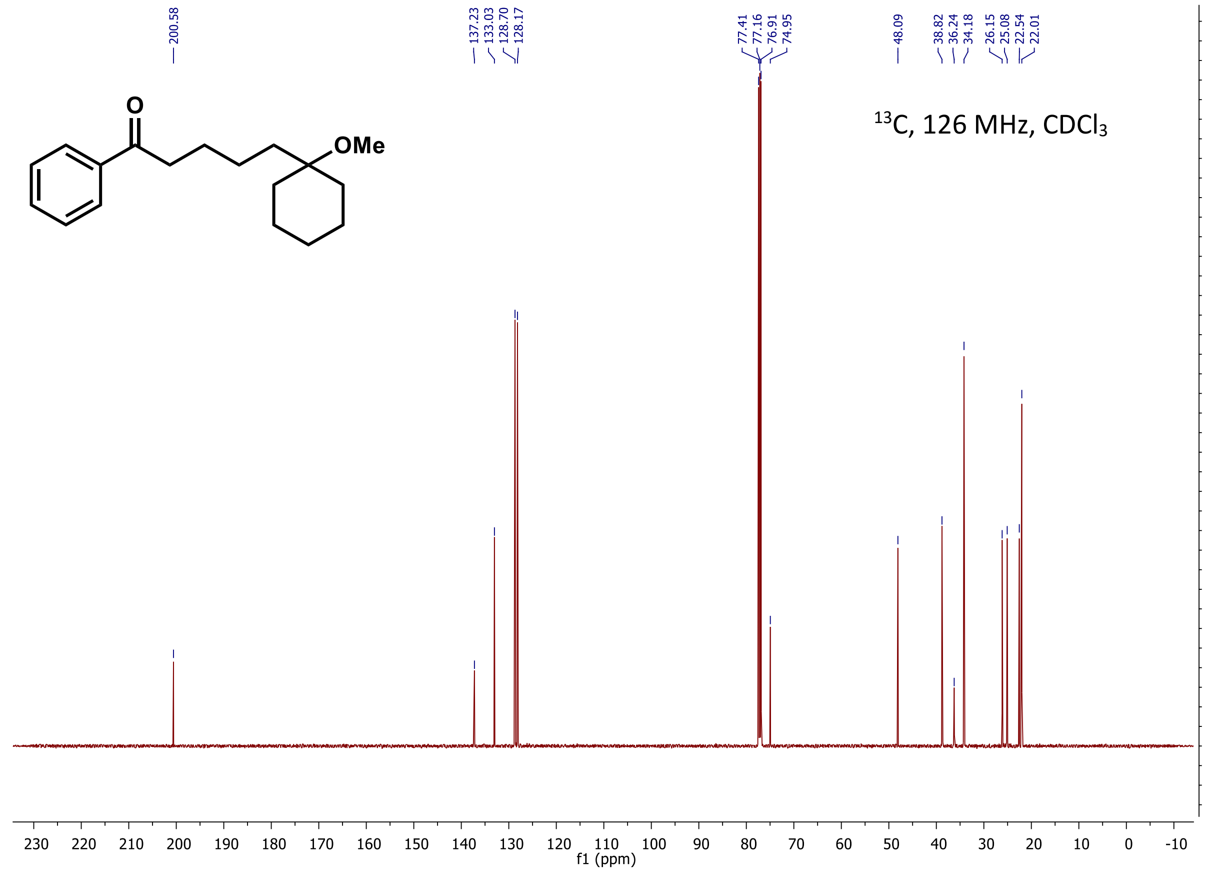

200.58
137.23 133.03 128.70 128.17
77.41 77.16 76.91 74.95
48.09
38.82 36.24 34.18
26.15 25.08 22.54 22.01
O
OMe
13C, 126 MHz, CDCl3
230 220 210 200 190 180 170 160 150 140 130 120 110 100 90 80 70 60 50 40 30 20 10 0 -10
f1 (ppm)

(26)

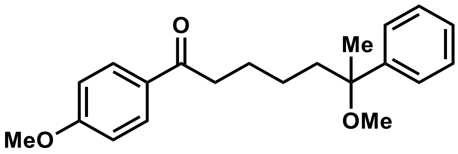

Prepared according to the General Procedure X using **S26** (88.9 mg, 0.30 mmol, 1 equiv.), *n*-Bu_4NPF_6 (232 mg, 0.60 mmol, 2 equiv.), dichloromethane (4.5 mL) and MeOH (1.5 mL). The yield was determined by crude ^{1}H NMR using 1,3,5-trimethylbenzene (42 µL, 1 equiv.) as an internal standard: 73%. Purification by flash column chromatography (eluent = 10 to 15% EtOAc in hexanes, silica gel) to afford product as a yellow oil (61.7 mg, 63% yield).

R_f = 0.23 (eluent =10% EtOAc in hexanes); **ν_{max} / cm^{-1}** (thin film) 2935, 2349, 2326, 1674, 1600, 1490, 1438, 1245, 1100, 1140, 700; **^{1}H NMR (500 MHz, $CDCl_3$)** δ_H: 1.20- 1.37 (2H, m), 1.53 (3H, s), 1.63-1.69 (2H, m), 1.78-1.82 (2H, m), 2.82-2.86 (2H, m), 3.08 (3H, s), 3.86 (3H, s), 6.88-6.92 (2H, m), 7.22-7.26 (1H, m), 7.32-7.37 (4H, m), 7.88-7.91 (2H, m); **^{13}C NMR (126 MHz, $CDCl_3$)** δ_C: 23.1, 23.9, 25.0, 38.4, 42.8, 50.4, 55.6, 79.1, 113.8, 126.3, 126.9, 128.2, 130.3, 130.4, 145.3, 163.4, 199.0; **HRMS (CI$^+$)** [$C_{21}H_{26}O_3$] requires [M]$^+$ 326.1882, found 326.1875 (- 2.1 ppm).

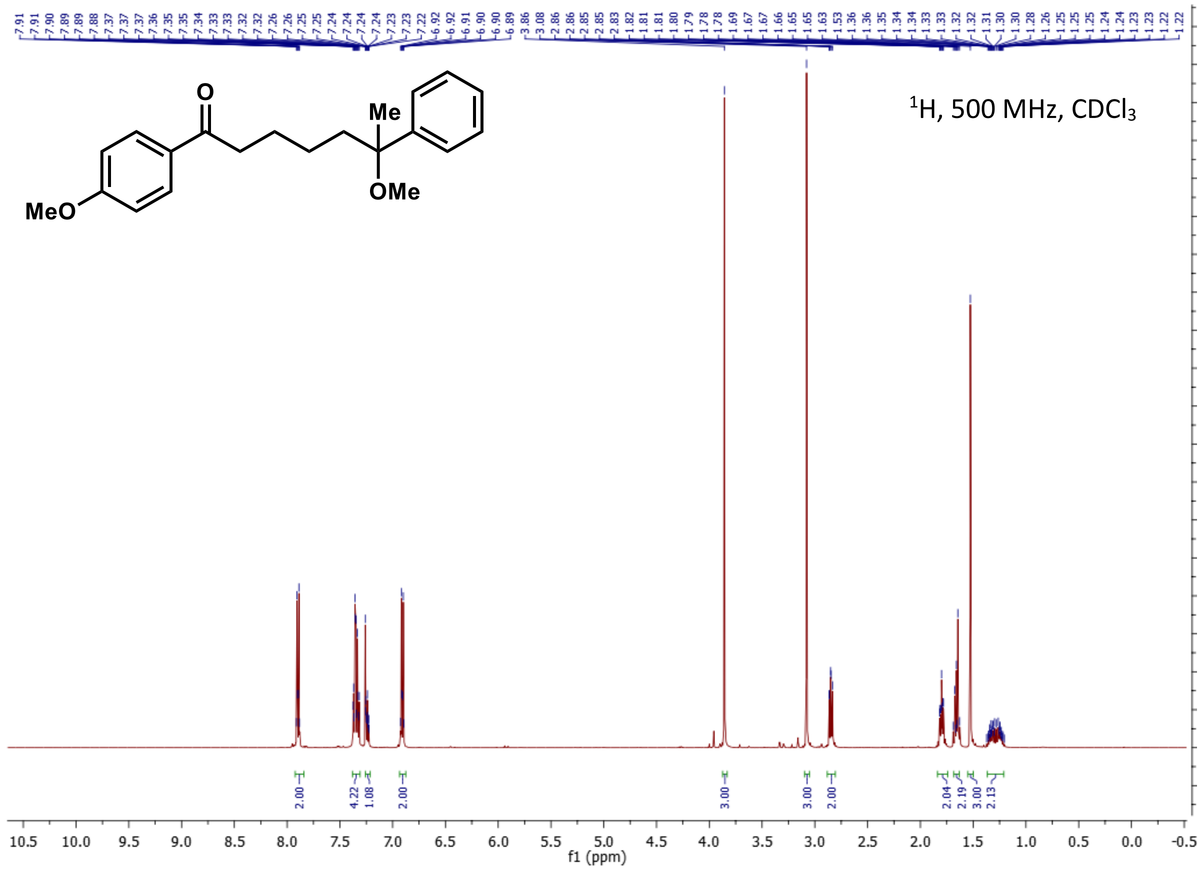

^{1}H, 500 MHz, $CDCl_3$
MeO
O
Me
OMe
f1 (ppm)

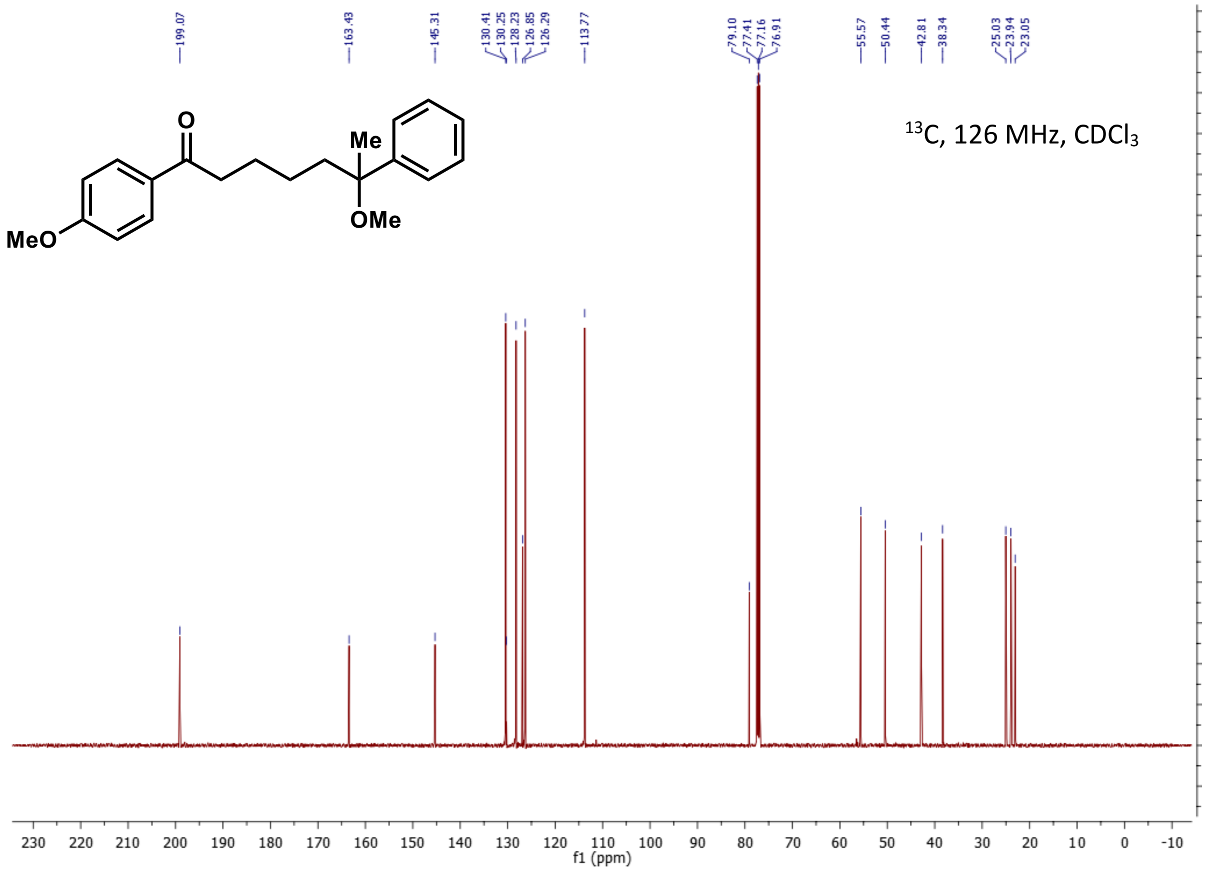

^{13}C, 126 MHz, $CDCl_3$
MeO
O
Me
OMe
199.07
163.43
145.31
130.41
130.25
128.23
126.85
126.29
113.77
79.10
77.41
77.16
76.91
55.57
50.44
42.81
38.34
25.03
23.94
23.05
f1 (ppm)

(27)

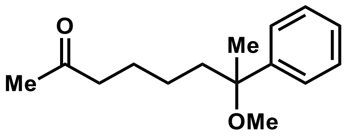

Prepared according to the General Procedure Y using **S27** (61.0 mg, 0.30 mmol, 1 equiv.), *n*-Bu_4NPF_6 (232 mg, 0.60 mmol, 2 equiv.), dichloromethane (4.5 mL) and MeOH (1.5 mL). The yield was determined by crude ^{1}H NMR using 1,3,5-trimethylbenzene (42 µL, 1 equiv.) as an internal standard: 70%. Purification by flash column chromatography (eluent = 10 to 25% EtOAc in hexanes, silica gel) to afford product as a colourless oil (46.3 mg, 65% yield).

R_f = 0.21 (eluent = 20% EtOAc in hexanes); **ν_{max} / cm^{-1}** (thin film) 2940, 1713, 1445, 1371, 1163, 1072, 700 ; **^{1}H NMR (500 MHz, $CDCl_3$)** δ_H: 1.10-1.30 (2H, m), 1.45-1.54 (5H, m), 1.71-1.77 (2H, m), 2.08 (3H, s), 2.33-2.38 (2H, m), 3.07 (3H, s), 7.22-7.27 (1H, m), 7.31-7.37 (4H, m); **^{13}C NMR (126 MHz, $CDCl_3$)** δ_C: 23.0, 23.7, 24.3, 30.0, 42.8, 43.8, 50.4, 79.0, 126.3, 126.9, 128.2, 145.3, 209.2; **HRMS (CI$^+$)** [$C_{15}H_{22}O_2$] requires [M-MeOH+H]$^+$ 203.1436, found 203.1429 (-3.4 ppm). Elimination of methanol observed upon ionisation.

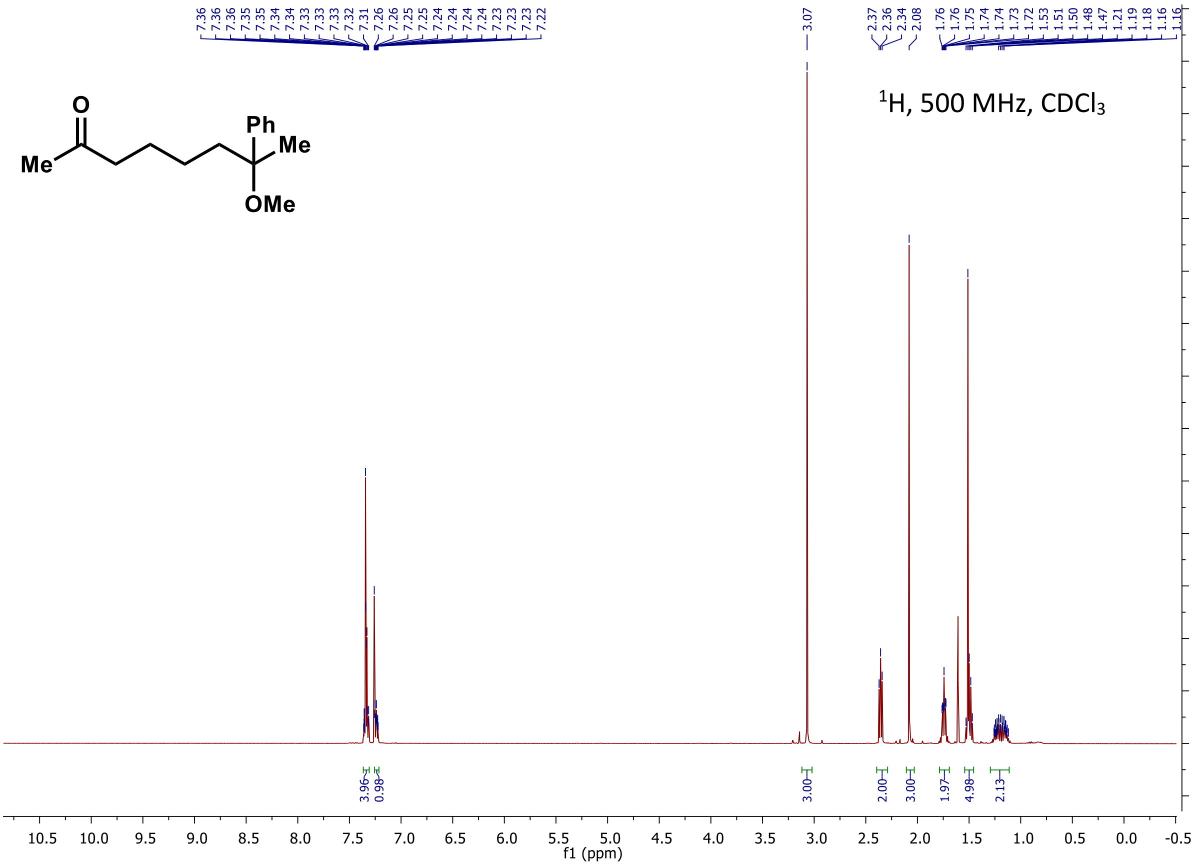

O
Ph
Me
Me
OMe
^{1}H, 500 MHz, CDCl$_3$
f1 (ppm)

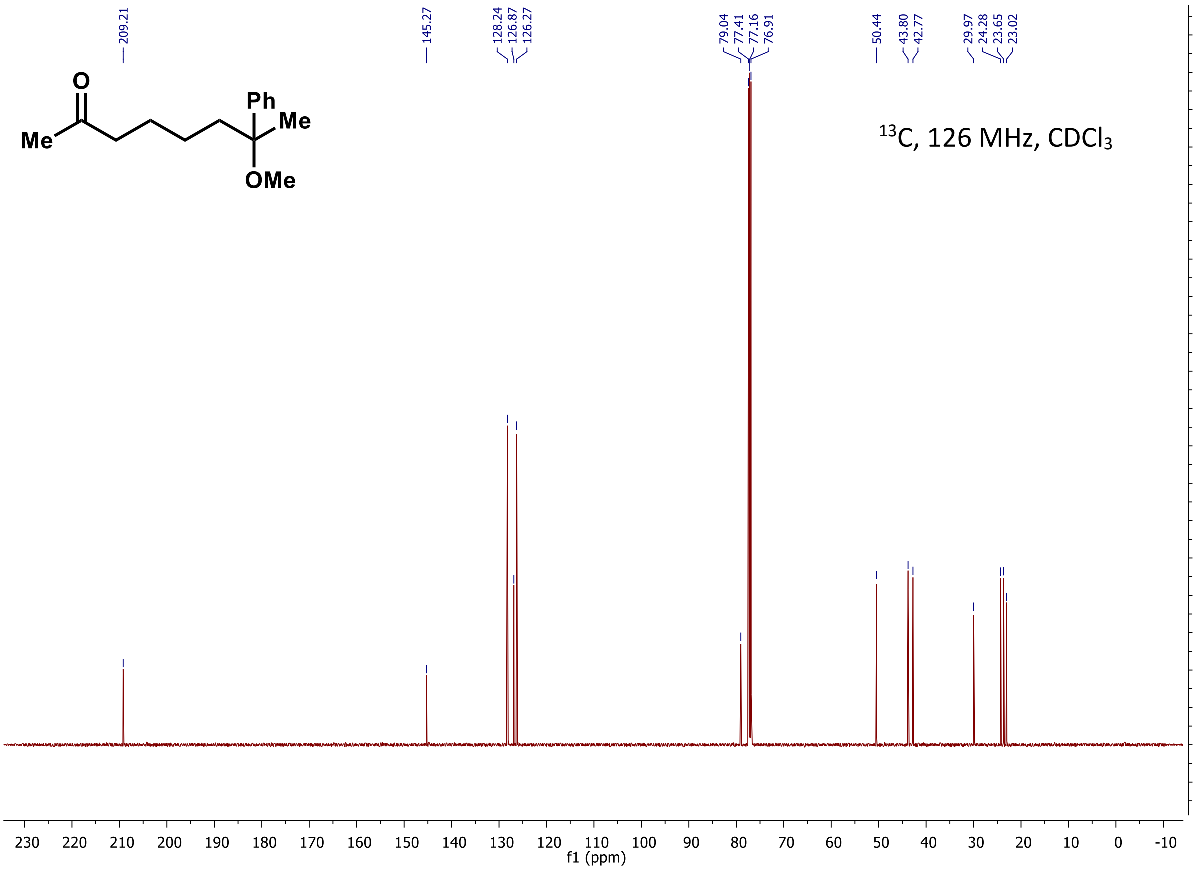

O
Ph
Me
Me
OMe
^{13}C, 126 MHz, CDCl$_3$
f1 (ppm)

(28)

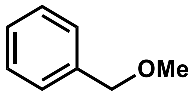

Prepared according to a modified General Procedure Y using **S28** (90.0 mg, 0.60 mmol, 1 equiv.), *n*-Bu_4NPF_6 (232 mg, 0.60 mmol, 2 equiv.), dichloromethane (4.5 mL) and MeOH (1.5 mL). The yield was determined by crude ^{1}H NMR using 1,3,5-trimethylbenzene (42 µL, 0.5 equiv.) as an internal standard: 54%. Purification by flash column chromatography (eluent = 5 to 15% EtOAc in hexanes, silica gel) to afford product as a colourless oil. Data consistent with the literature.[5]

R$_f$ = 0.48 (eluent = 5% EtOAc in hexanes; **^{1}H NMR (500 MHz, $CDCl_3$)** δ_H: 3.40 (3H, s), 4.47 (2H, s), 7.33 (5H, m); **^{13}C NMR (126 MHz, $CDCl_3$)** δ_C: 58.2, 74.9, 127.8, 127.9, 128.5, 138.3.

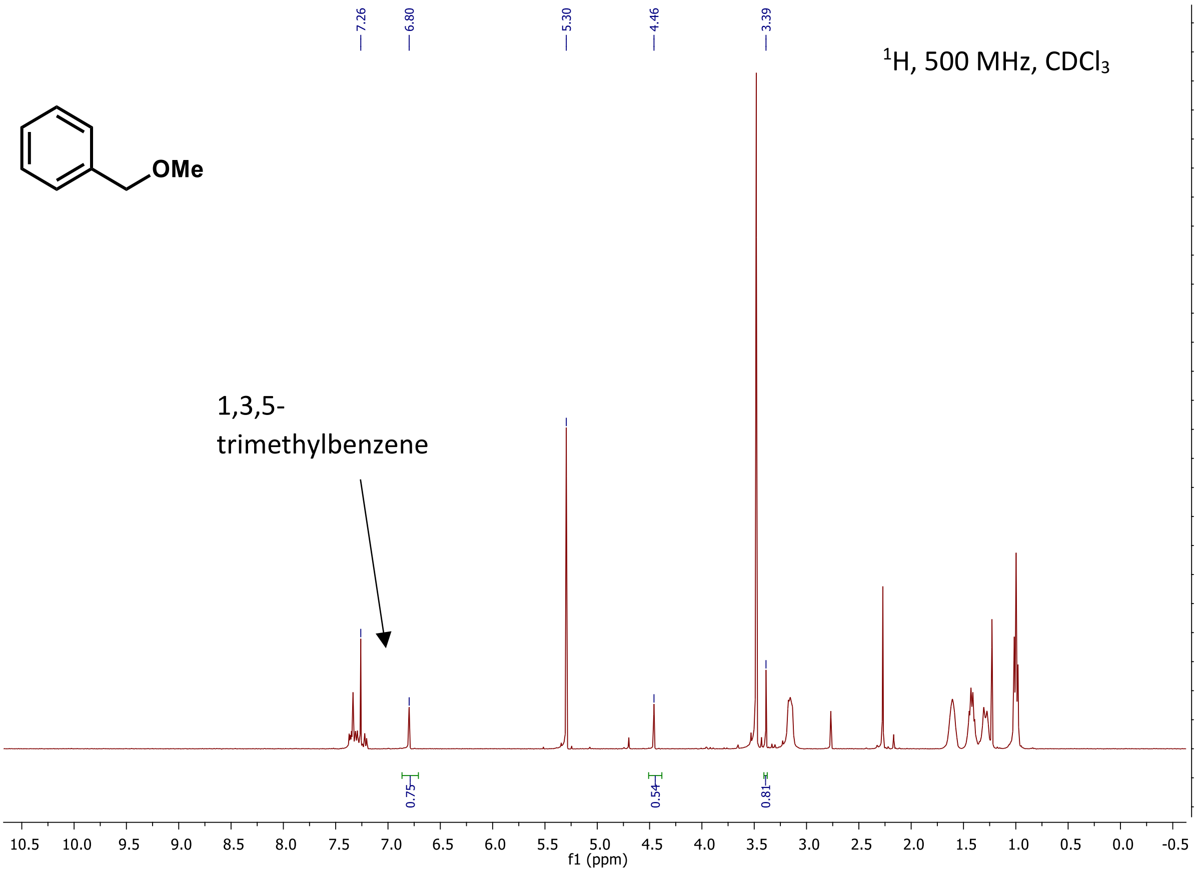

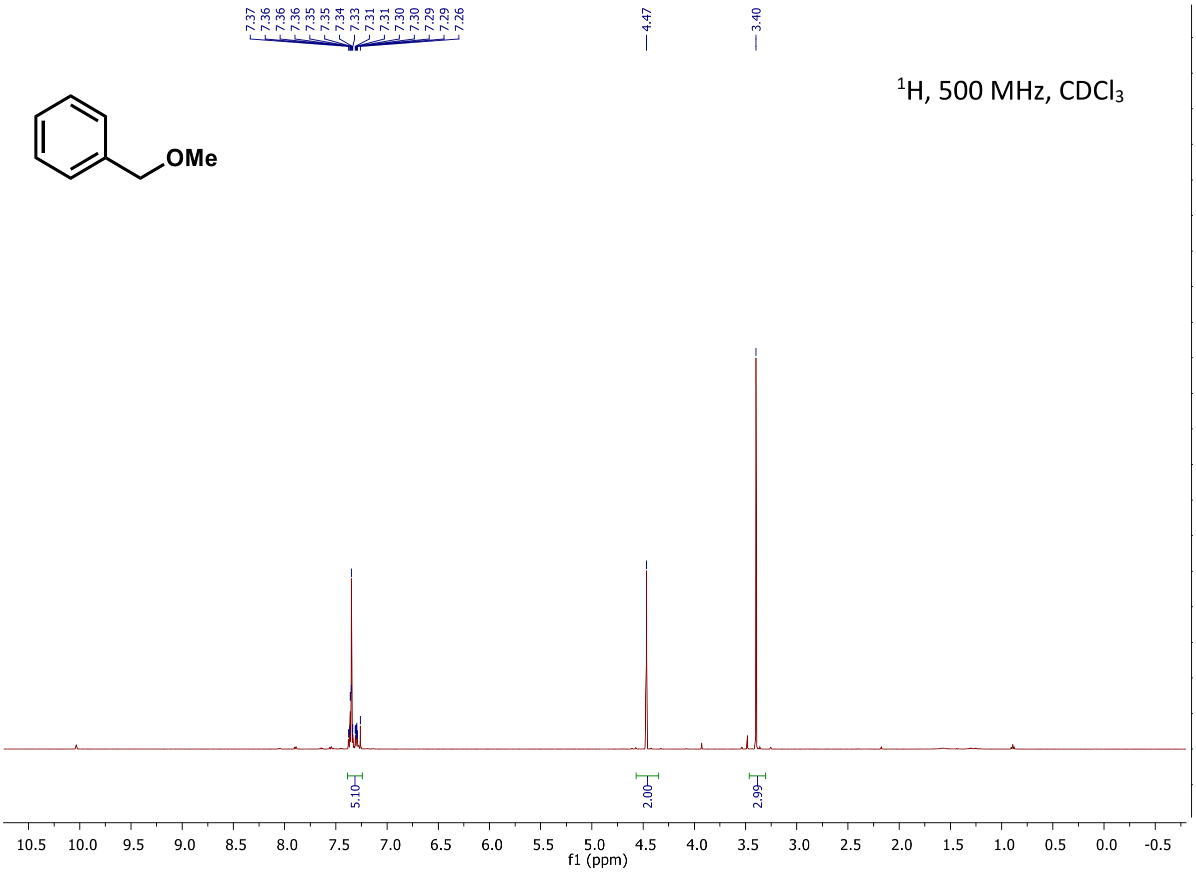
7.37
7.36
7.36
7.36
7.35
7.35
7.34
7.33
7.31
7.31
7.30
7.30
7.29
7.29
7.26
4.47
3.40
^{1}H, 500 MHz, CDCl$_3$
OMe
5.10
2.00
2.99
f1 (ppm)

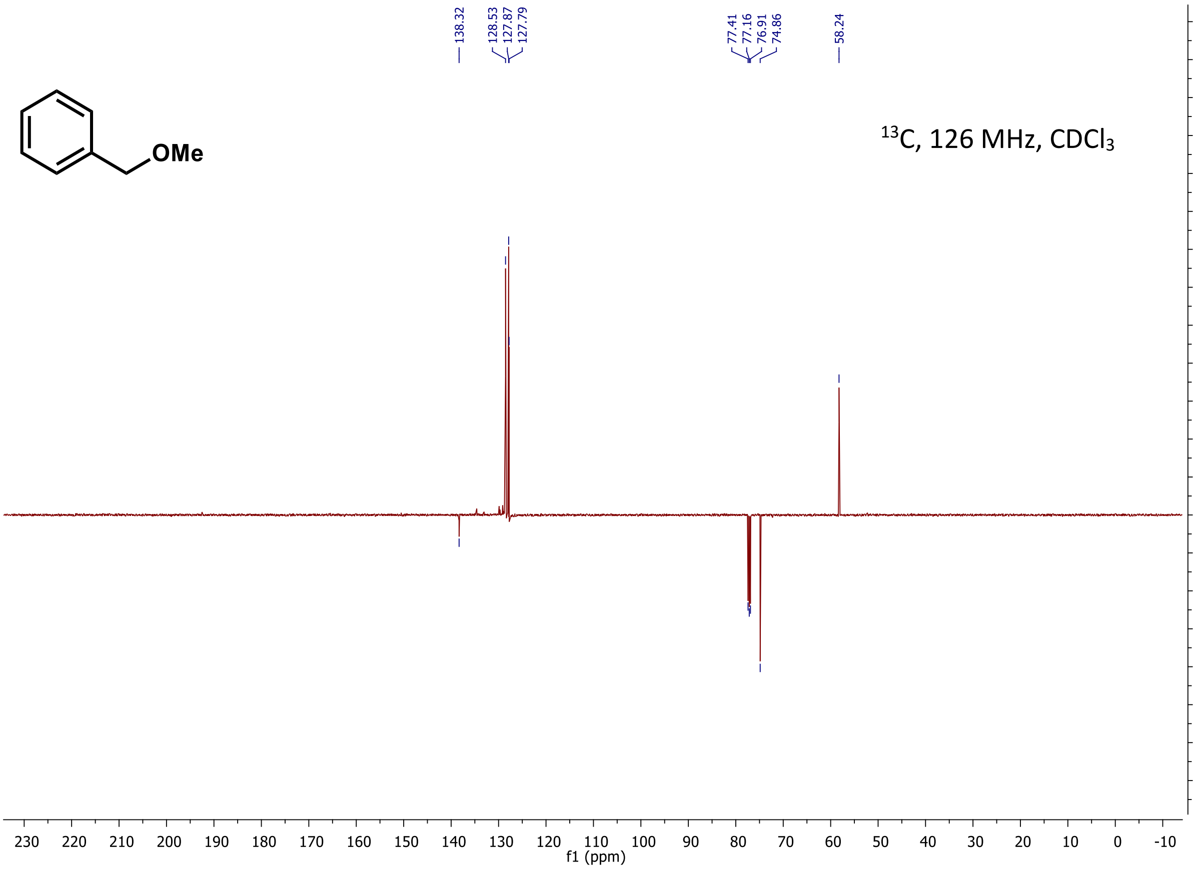
138.32
128.53
127.87
127.79
77.41
77.16
76.91
74.86
58.24
^{13}C, 126 MHz, CDCl$_3$
OMe
f1 (ppm)

(29)

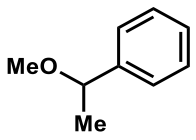

Prepared according to the General Procedure Y using **S29A** (49.3 mg, 0.30 mmol, 1 equiv.), *n*-Bu_4NPF_6 (232 mg, 0.60 mmol, 2 equiv.), dichloromethane (4.5 mL) and MeOH (1.5 mL). The yield was determined by crude ^{1}H NMR using 1,3,5-trimethylbenzene (42 µL, 1 equiv.) as an internal standard: 65%. Purification by flash column chromatography (eluent = 0-5% Et_2O in pentane, silica gel) to afford product as a colourless oil (21.0 mg, 51% yield).

Data consistent with the literature.[6]

R$_f$ = 0.61 (eluent = 10% Et_2O in hexanes); **ν_{max} / cm^{-1}** (thin film); **^{1}H NMR (500 MHz, $CDCl_3$)** δ_H: 1.37 (3H, d, *J* 6.5 Hz), 3.15 (3H, s), 4.22 (1H, q, *J* 6.5 Hz), 7.15-7.33 (5H, m); **^{13}C NMR (126 MHz, $CDCl_3$)** δ_C: 24.0, 56.6, 79.8, 126.3, 127.6, 128.6, 143.6.

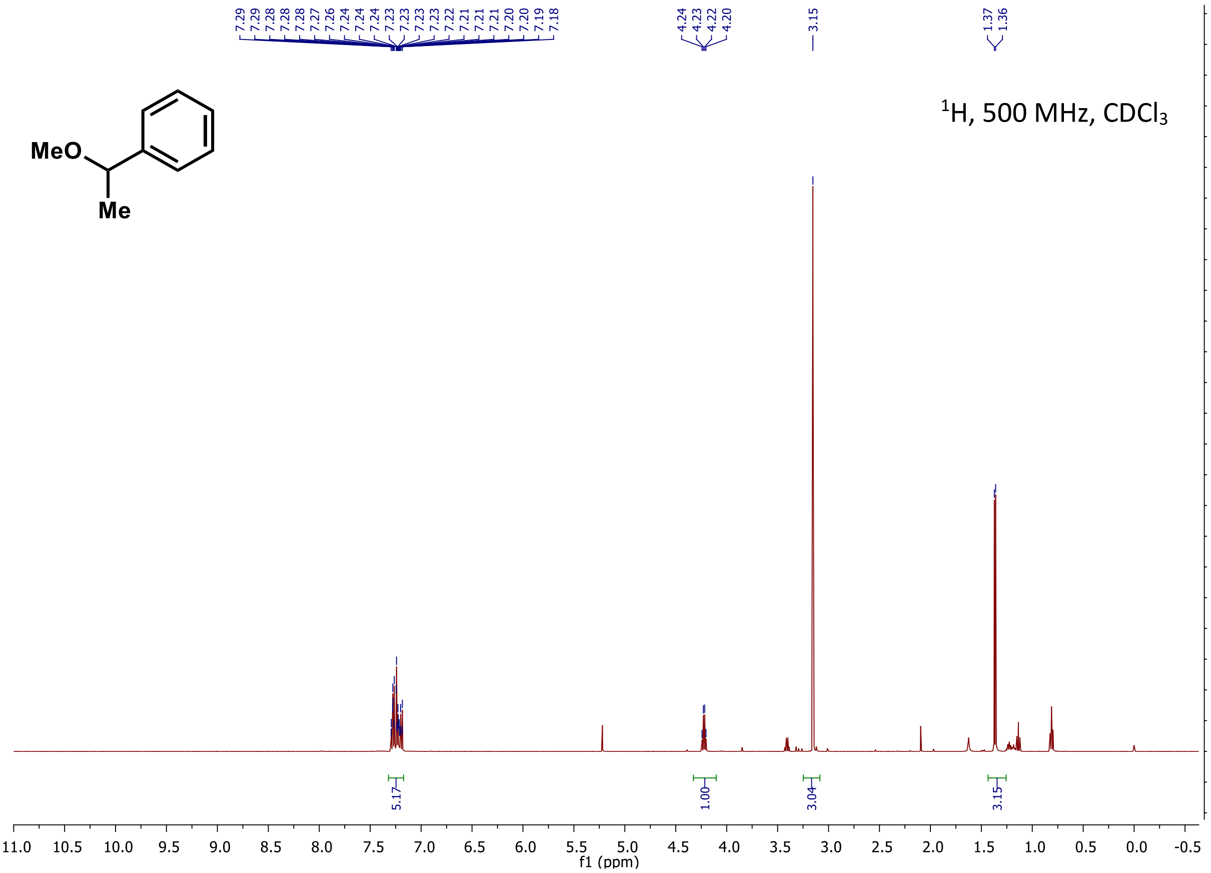

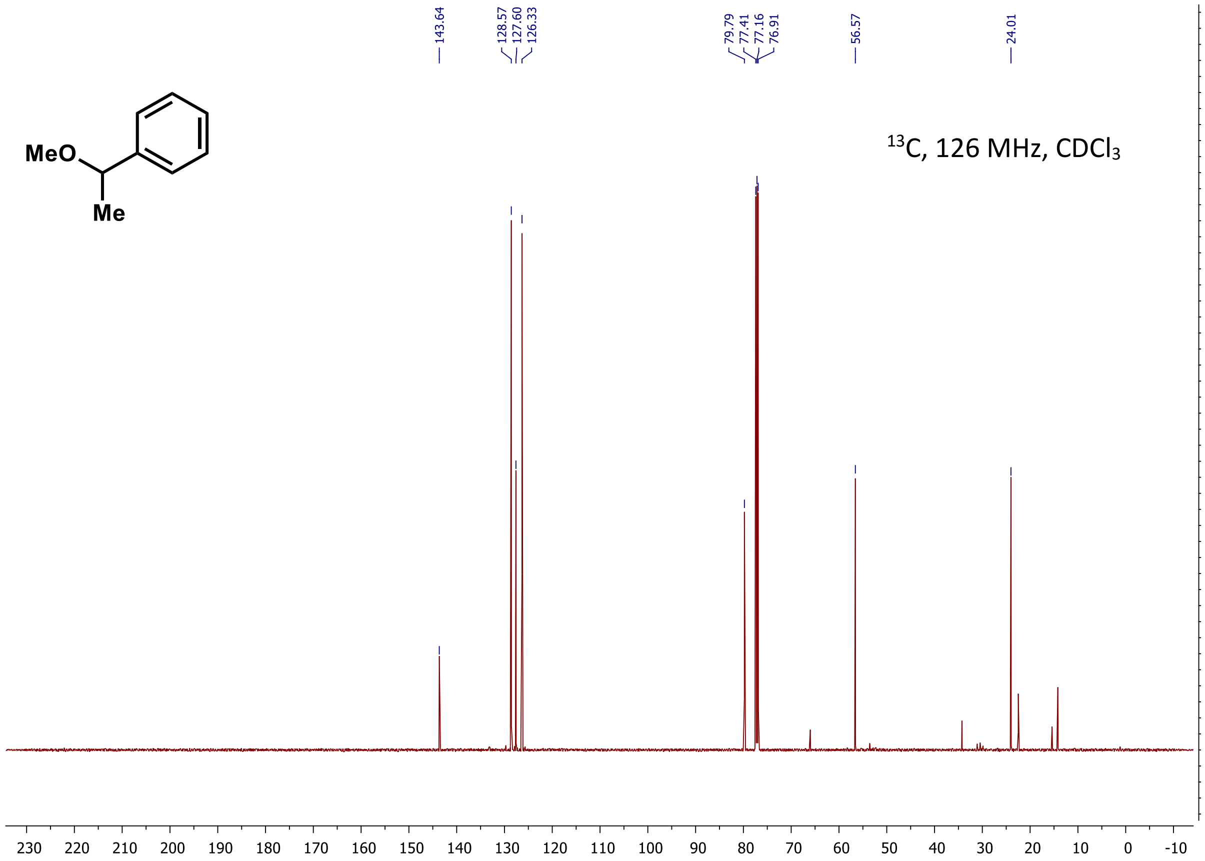

Also prepared according to the General Procedure Y using **S29B** (40.8 mg, 0.30 mmol, 1 equiv.), NBu_4PF_6 (232 mg, 0.60 mmol, 2 equiv.), dichloromethane (4.5 mL) and MeOH (1.5 mL). The yield was determined by crude ^{1}H NMR using 1,3,5-trimethylbenzene (42 µL, 1.0 equiv.) as an internal standard: 59%.

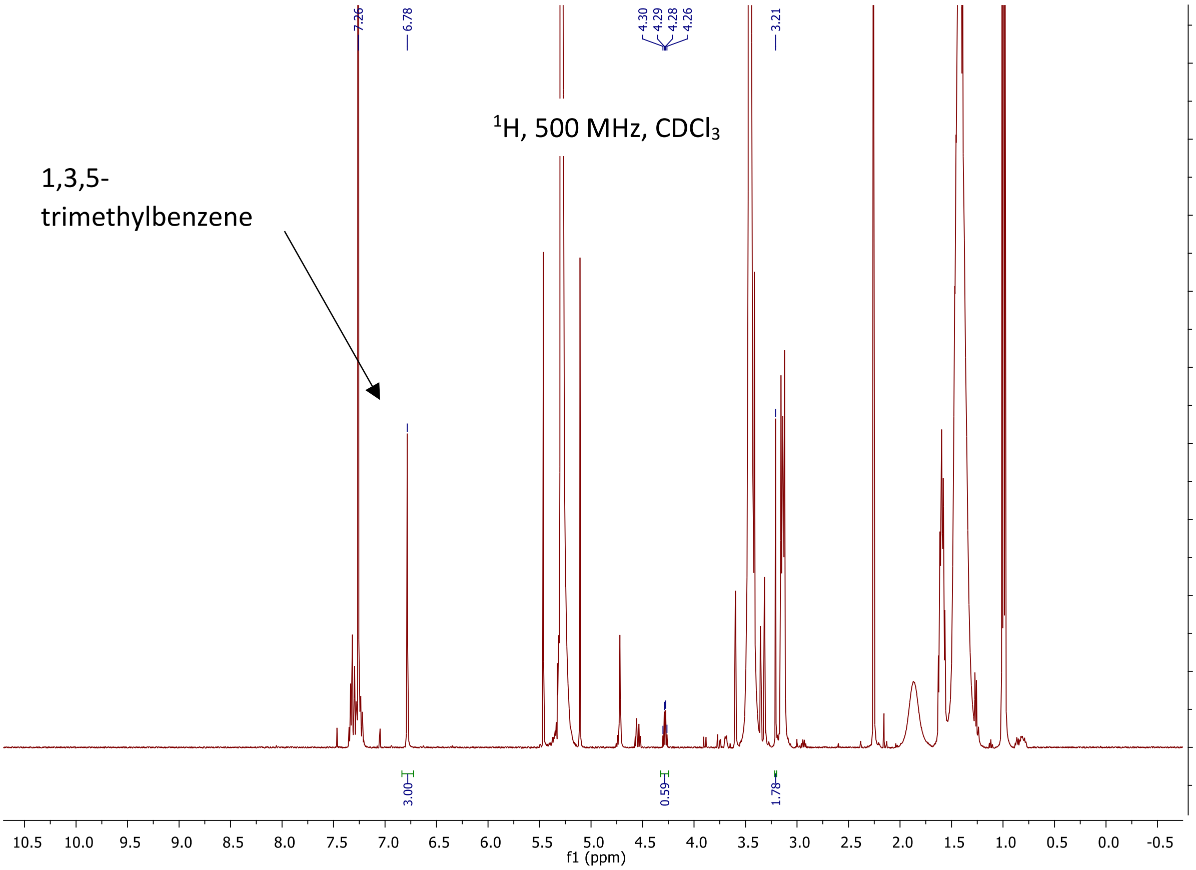

^{1}H, 500 MHz, $CDCl_3$
1,3,5-trimethylbenzene
7.26
6.78
4.30
4.29
4.28
4.26
3.21
3.00
0.59
1.78
10.5 10.0 9.5 9.0 8.5 8.0 7.5 7.0 6.5 6.0 5.5 5.0 4.5 4.0 3.5 3.0 2.5 2.0 1.5 1.0 0.5 0.0 -0.5
f1 (ppm)

(30)

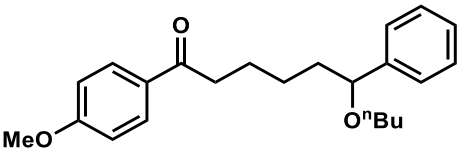

Prepared according to the General Procedure Y using **1** (85.0 mg, 0.30 mmol, 1 equiv.), *n*-Bu_4NPF_6 (232 mg, 0.60 mmol, 2 equiv.), dichloromethane (4.5 mL) and n-BuOH (1.5 mL). The yield was determined by crude ^{1}H NMR using 1,3,5-trimethylbenzene (42 µL, 1 equiv.) as an internal standard: 67%. Purification by flash column chromatography (eluent = 5% EtOAc in hexanes, silica gel) to afford product as a white solid (61.1 mg, 57% yield).

Mp.: 47-49 °C; **R_f** = 0.40 (eluent = 20% EtOAc in hexanes); **ν_{max} / cm^{-1}** (thin film) 2934, 2862, 1674, 1599, 1510, 1454, 1258, 1171, 1028; **^{1}H NMR (500 MHz, $CDCl_3$)** δ_H: 0.87 (3H, t, *J* 7.4 Hz), 1.30-1.40 (3H, m), 1.47-1.55 (3H, m), 1.61-1.69 (1H, m), 1.69-1.78 (2H, m), 1.79-1.91 (1H, m), 2.84-2.93 (2H, m), 3.22 (1H, app dt, *J* 9.3, 6.6 Hz), 3.30 (1H, app dt, *J* 9.3, 6.6 Hz), 3.87 (3H, s), 4.18 (1H, dd, *J* 7.7, 5.7 Hz), 6.92 (2H, d, *J* 8.9 Hz), 7.23-7.26 (1H, m), 7.27-7.29 (2H, m), 7.29-7.38 (2H, m), 7.92 (2H, d, *J* 8.9 Hz); **^{13}C NMR (126 MHz, $CDCl_3$)** δ_C: 14.1, 19.6, 24.6, 25.9, 32.2, 38.4, 38.4, 55.6, 68.8, 82.2, 113.8, 126.7, 127.4, 128.4, 130.3, 130.5, 143.4, 163.5, 199.2; **HRMS (ES$^+$)** [$C_{23}H_{30}O_3$] requires [M+Na]$^+$ 377.2093, found 377.2092 (- 0.3 ppm).

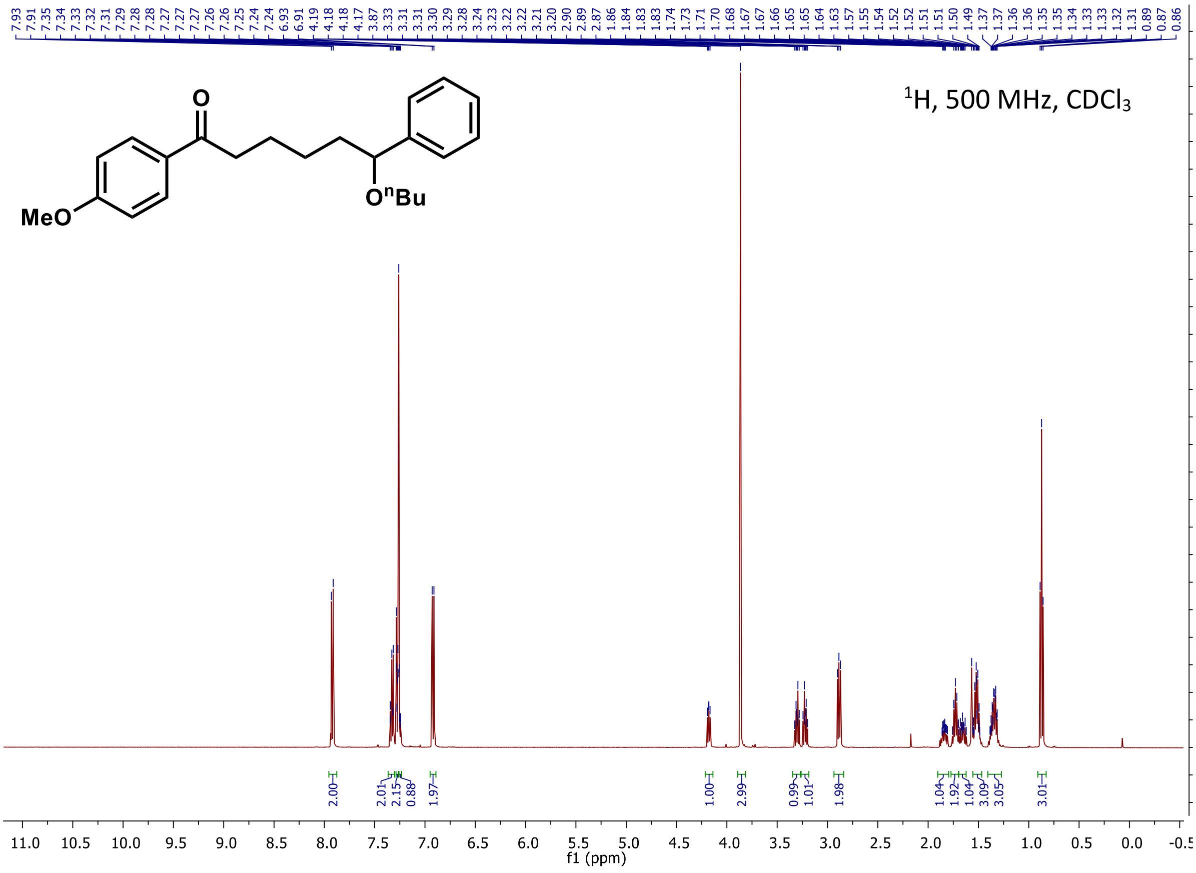
7.93 7.91 7.35 7.34 7.33 7.32 7.31 7.29 7.28 7.27 7.27 7.27 7.26 7.25 7.24 7.24 6.93 6.91 4.19 4.18 4.18 4.17 3.87 3.33 3.31 3.31 3.30 3.29 3.28 3.24 3.23 3.22 3.22 3.21 3.20 2.90 2.89 2.87 1.86 1.84 1.83 1.83 1.74 1.73 1.71 1.70 1.68 1.67 1.67 1.66 1.65 1.65 1.64 1.63 1.57 1.55 1.54 1.52 1.52 1.51 1.51 1.50 1.49 1.37 1.37 1.36 1.35 1.35 1.34 1.33 1.33 1.32 1.31 0.89 0.87 0.86
O
MeO
O^{n}Bu
^{1}H, 500 MHz, CDCl$_3$
2.00 2.01 2.15 0.88 1.97 1.00 2.99 0.99 1.01 1.98 1.04 1.92 1.04 3.09 3.05 3.01
f1 (ppm)

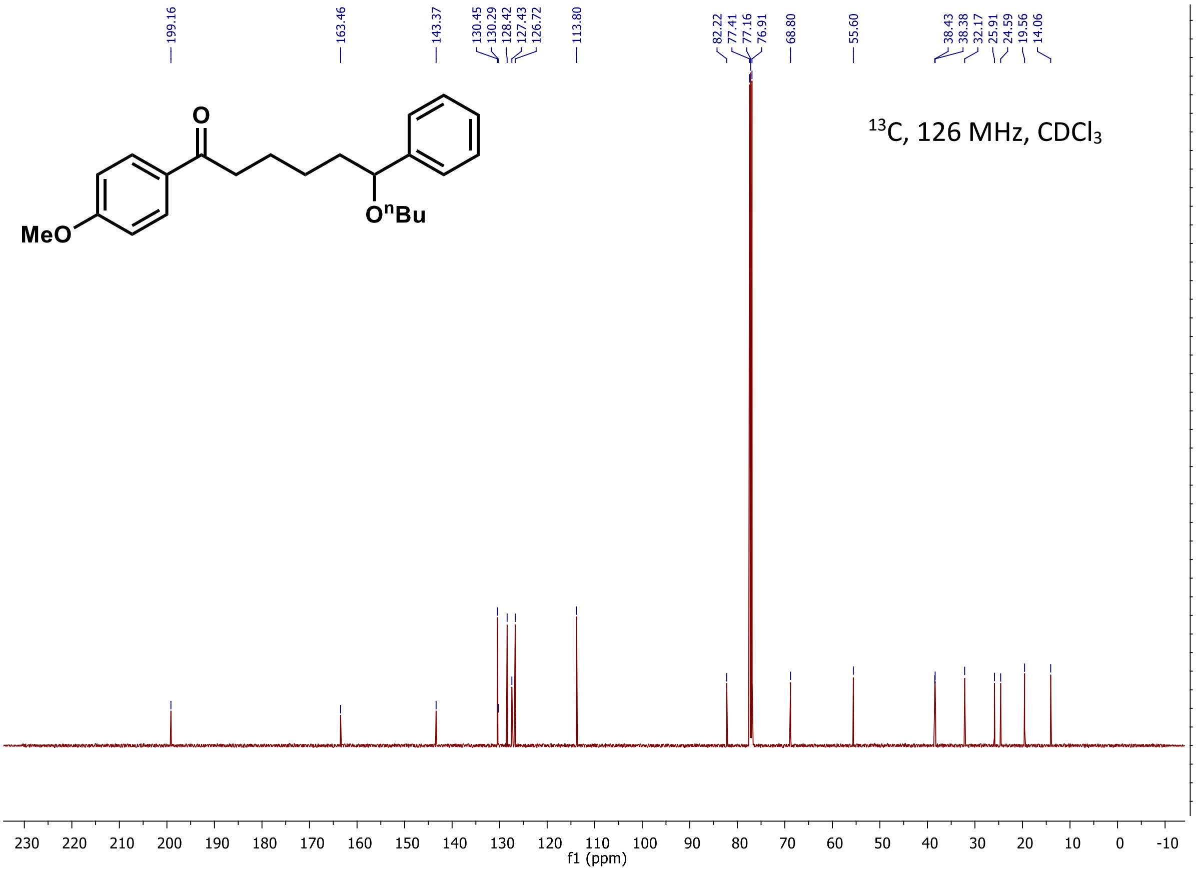
199.16
163.46
143.37
130.45 130.29 128.42 127.43 126.72
113.80
82.22 77.41 77.16 76.91
68.80
55.60
38.43 38.38 32.17 25.91 24.59 19.56 14.06
O
MeO
O^{n}Bu
^{13}C, 126 MHz, CDCl$_3$
f1 (ppm)

(31)

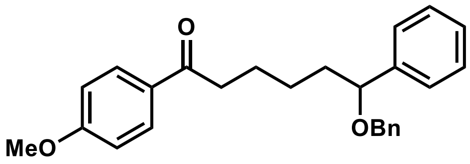

Prepared according to the General Procedure Y using **1** (85.0 mg, 0.30 mmol, 1 equiv.), *n*-Bu_4NPF_6 (232 mg, 0.60 mmol, 2 equiv.), dichloromethane (4.5 mL) and BnOH (1.5 mL). The yield was determined by crude ^{1}H NMR using 1,3,5-trimethylbenzene (42 µL, 1 equiv.) as an internal standard: 70%. Purification by flash column chromatography (eluent = 5% EtOAc in hexanes, silica gel) to afford product as a white solid (52.9 mg, 47% yield).

Mp.: 44-46 °C; $\mathbf{R_f}$ = 0.60 (eluent = 20% EtOAc in hexanes); $\mathbf{\nu_{max}}$ **/ cm^{-1}** (thin film) 2935, 1674, 1599, 1576, 1508, 1454, 1258, 1169; **^{1}H NMR (500 MHz, $CDCl_3$)** δ_H: 1.30-1.43 (1H, m), 1.49-1.59 (1H, m), 1.66-1.75 (3H, m), 1.85-1.98 (1H, m), 2.78-2.92 (2H, m), 3.86 (3H, s), 4.24 (1H, d, *J* 11.8 Hz), 4.32 (1H, dd, *J* 7.7, 5.7 Hz), 4.44 (1H, d, *J* 11.8 Hz), 6.92 (2H, d, *J* 8.9 Hz), 7.22-7.42 (10H, m), 7.91 (2H, d, *J* 8.9 Hz); **^{13}C NMR (126 MHz, $CDCl_3$)** δ_C: 24.6, 25.9, 38.3, 38.3, 55.6, 70.6, 81.5, 113.8, 127.0, 127.6, 127.7, 127.9, 128.5, 128.6, 130.3, 130.5, 138.8, 142.7, 163.5, 199.1; **HRMS (ES$^+$)** [$C_{26}H_{28}O_3$] requires $[M+Na]^+$ 411.1936, found 411.1924 (-2.9 ppm).

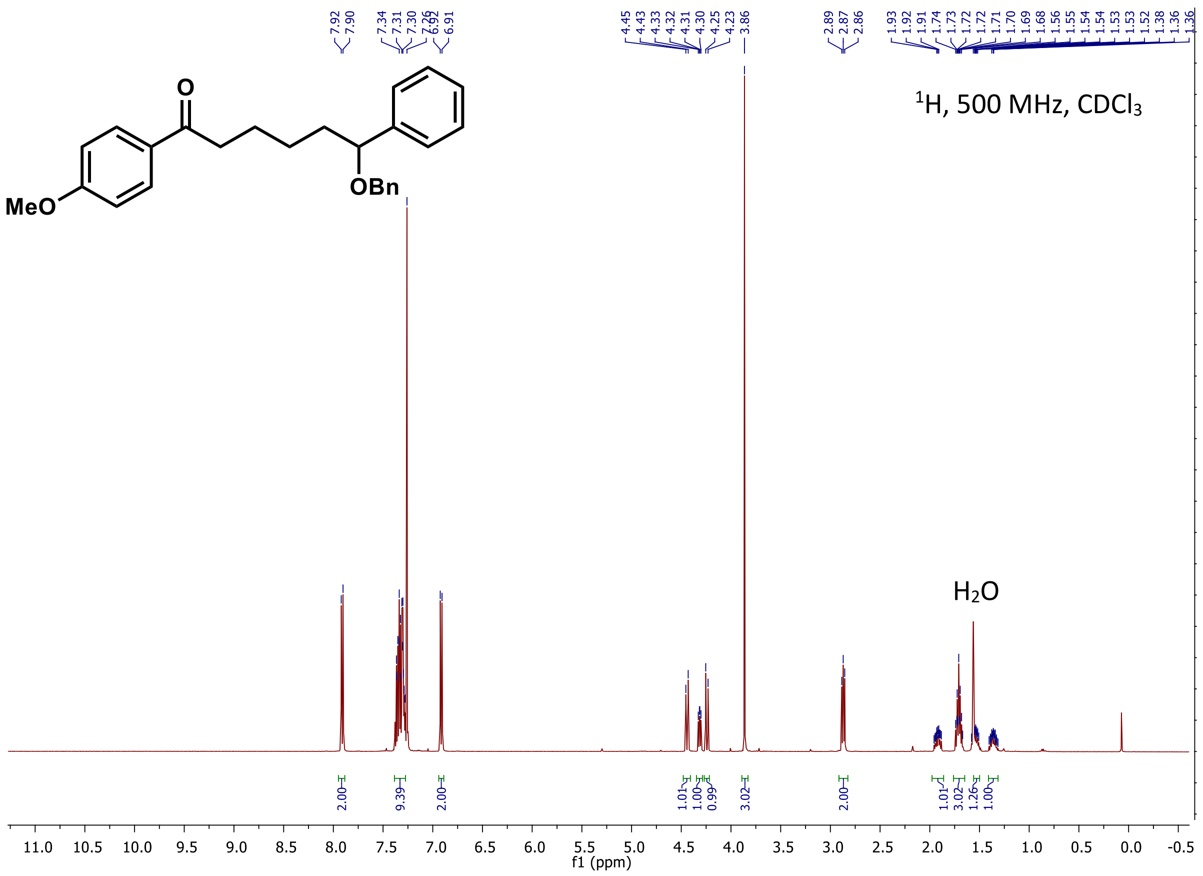
MeO
O
OBn
^{1}H, 500 MHz, CDCl$_3$
H_2O
f1 (ppm)

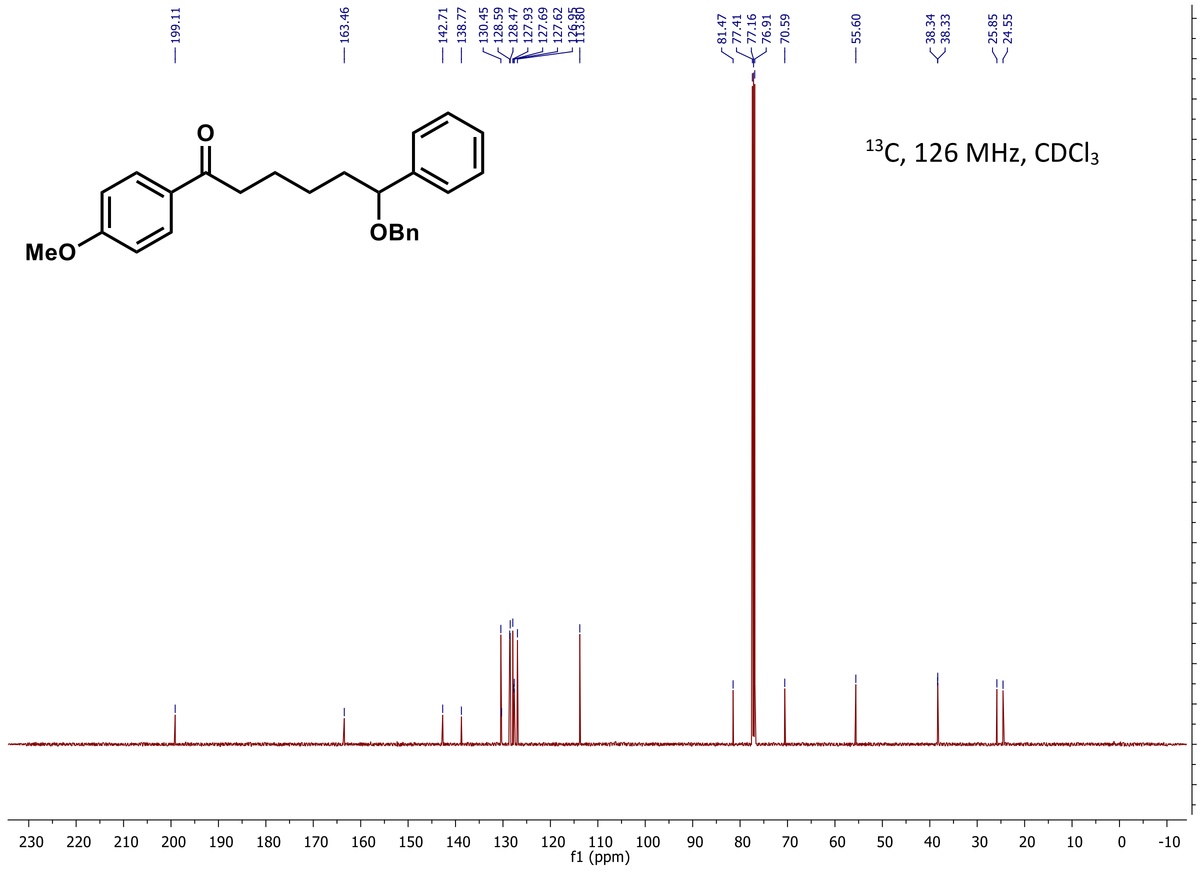
MeO
O
OBn
^{13}C, 126 MHz, CDCl$_3$
f1 (ppm)

(32)

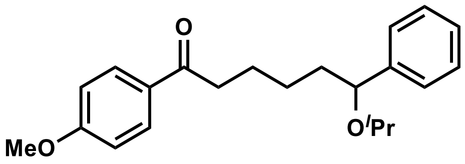

Prepared according to the General Procedure Y using **1** (85.0 mg, 0.30 mmol, 1 equiv.), *n*-Bu_4NPF_6 (232 mg, 0.60 mmol, 2 equiv.), dichloromethane (4.5 mL) and IPA (1.5 mL). The yield was determined by crude ^{1}H NMR using 1,3,5-trimethylbenzene (42 µL, 1 equiv.) as an internal standard: 58%. Purification by flash column chromatography (eluent = 5% EtOAc in hexanes, silica gel) to afford product as a yellow solid (55.9 mg, 55% yield).

Mp.: 60-62 °C; **R_f** = 0.43 (eluent = 20% EtOAc in hexanes); **ν_{max} / cm^{-1}** (thin film) 2972, 2860, 1674, 1600, 1506, 1460, 1375, 1263, 1165, 1024; **^{1}H NMR (500 MHz, $CDCl_3$)** δ_H: 1.07 (3H, d, *J* 6.1 Hz), 1.13 (3H, d, *J* 6.1 Hz), 1.30-1.41 (1H, m), 1.47-1.55 (1H, m), 1.59-1.67 (1H, m), 1.69-1.77 (2H, m), 1.77-1.84 (1H, m), 2.82-2.94 (2H, m), 3.46 (1H, hept, *J* 6.1 Hz), 3.87 (3H, s), 4.32 (1H, dd, *J* 8.0, 5.4 Hz), 6.92 (2H, d, *J* 8.9 Hz), 7.20-7.28 (1H,m), 7.27-7.37 (4H, m), 7.92 (2H, d, *J* 8.9 Hz); **^{13}C NMR (126 MHz, $CDCl_3$)** δ_C: 21.4, 23.6, 24.6, 26.0, 38.4, 38.8, 55.6, 68.9, 79.2, 113.8, 126.7, 127.3, 128.4, 130.3, 130.5, 144.1, 163.5, 199.2; **HRMS (ES$^+$)** [$C_{22}H_{28}O_3$] requires $[M+Na]^+$ 363.1936, found 363.1935 (- 0.3 ppm).

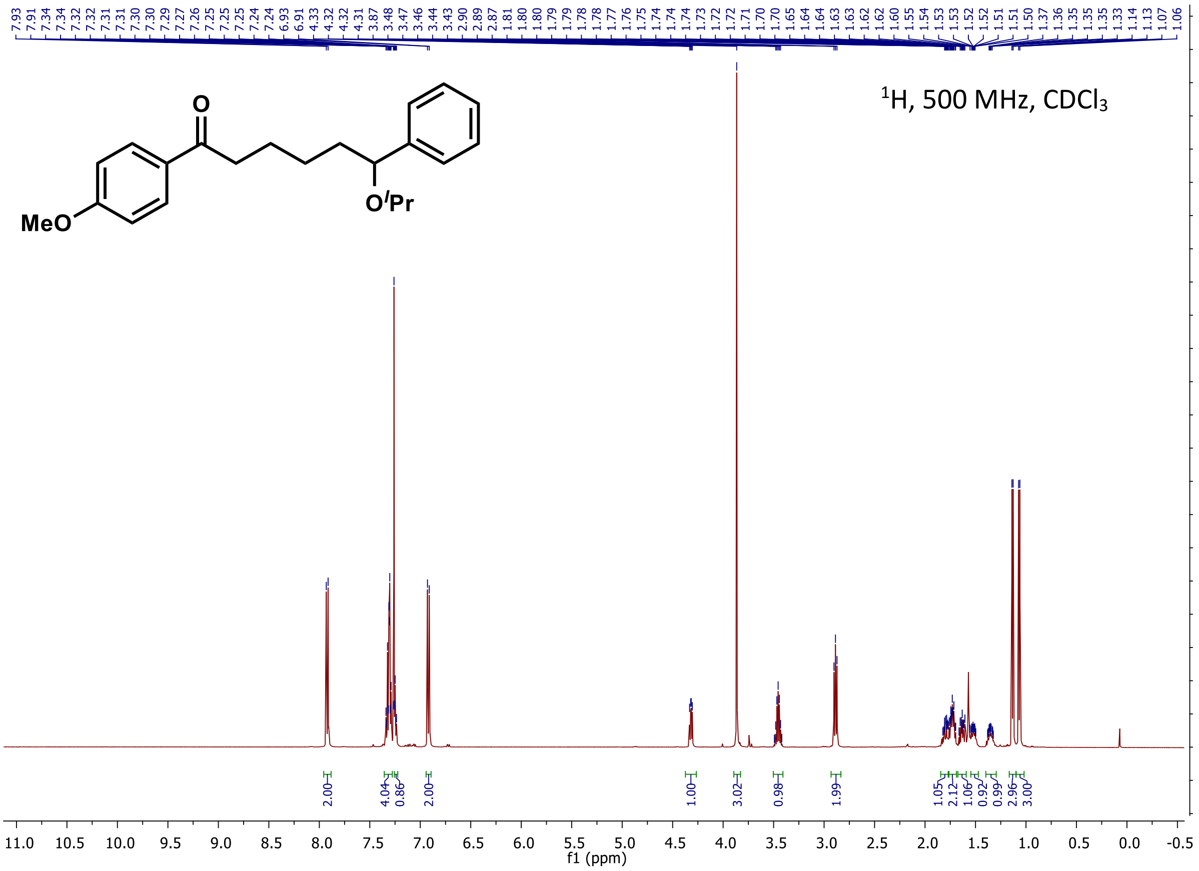

MeO
O
O^{i}Pr
^{1}H, 500 MHz, CDCl$_3$
f1 (ppm)

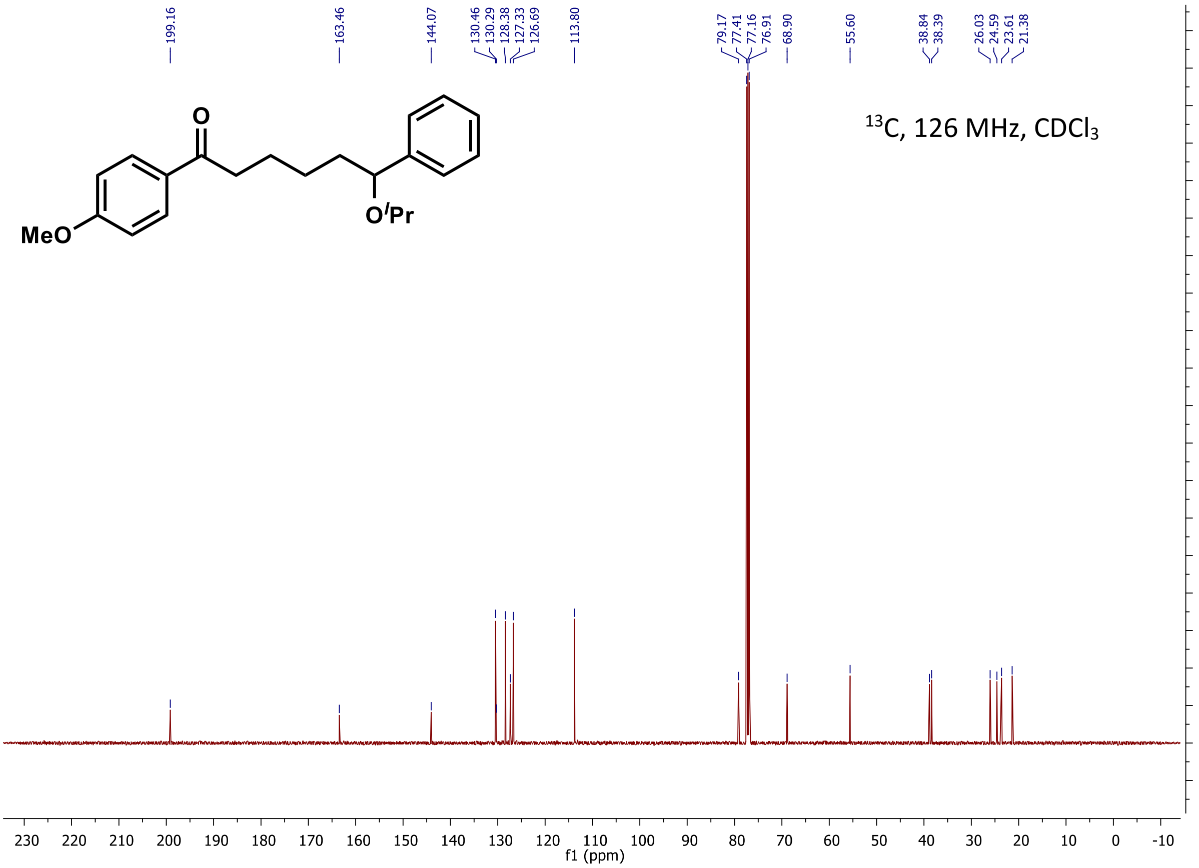

MeO
O
O^{i}Pr
^{13}C, 126 MHz, CDCl$_3$
199.16
163.46
144.07
130.46
130.29
128.38
127.33
126.69
113.80
79.17
77.41
77.16
76.91
68.90
55.60
38.84
38.39
26.03
24.59
23.61
21.38
f1 (ppm)

(33)

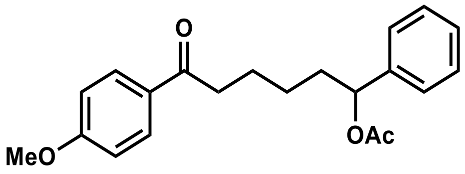

Prepared according to the General Procedure Y using **1** (85.0 mg, 0.30 mmol, 1 equiv.), *n*-Bu_4NOAc (181 mg, 0.60 mmol, 2 equiv.), dichloromethane (5.8 mL) and AcOH (0.2 mL). The yield was determined by crude ^{1}H NMR using 1,3,5-trimethylbenzene (42 µL, 1 equiv.) as an internal standard: 68%. Purification by flash column chromatography (eluent = 5-20% EtOAc in hexanes, silica gel) to afford product as a yellow oil (67.8 mg, 65% yield).

R_f = 0.27 (eluent = 20% EtOAc in hexanes); **ν_{max} / cm^{-1}** (thin film) 2938, 1732, 1674, 1599, 1508, 1369, 1226, 1169, 1024; **^{1}H NMR (500 MHz, $CDCl_3$)** δ_H: 1.28-1.37 (1H, m), 1.39-1.49 (1H, m), 1.70-1.78 (2H, m), 1.79-1.88 (1H, m), 1.92-2.01 (1H, m), 2.06 (3H, s), 2.80-2.94 (2H, m), 3.87 (3H, s), 5.74 (1H, dd, *J* 7.6, 6.3 Hz), 6.92 (2H, d, *J* 8.9 Hz), 7.28-7.37 (5H, m), 7.91 (2H, d, *J* 8.9 Hz); **^{13}C NMR (126 MHz, $CDCl_3$)** δ_C: 21.4, 24.3, 25.5, 36.3, 38.2, 55.6, 76.1, 113.8, 126.7, 128.0, 128.6, 130.2, 130.4, 140.8, 163.5, 170.5, 198.8; **HRMS (ES$^+$)** [$C_{21}H_{24}O_4$] requires [M+Na]$^+$ 363.1572, found 363.1572 (+0.0 ppm).

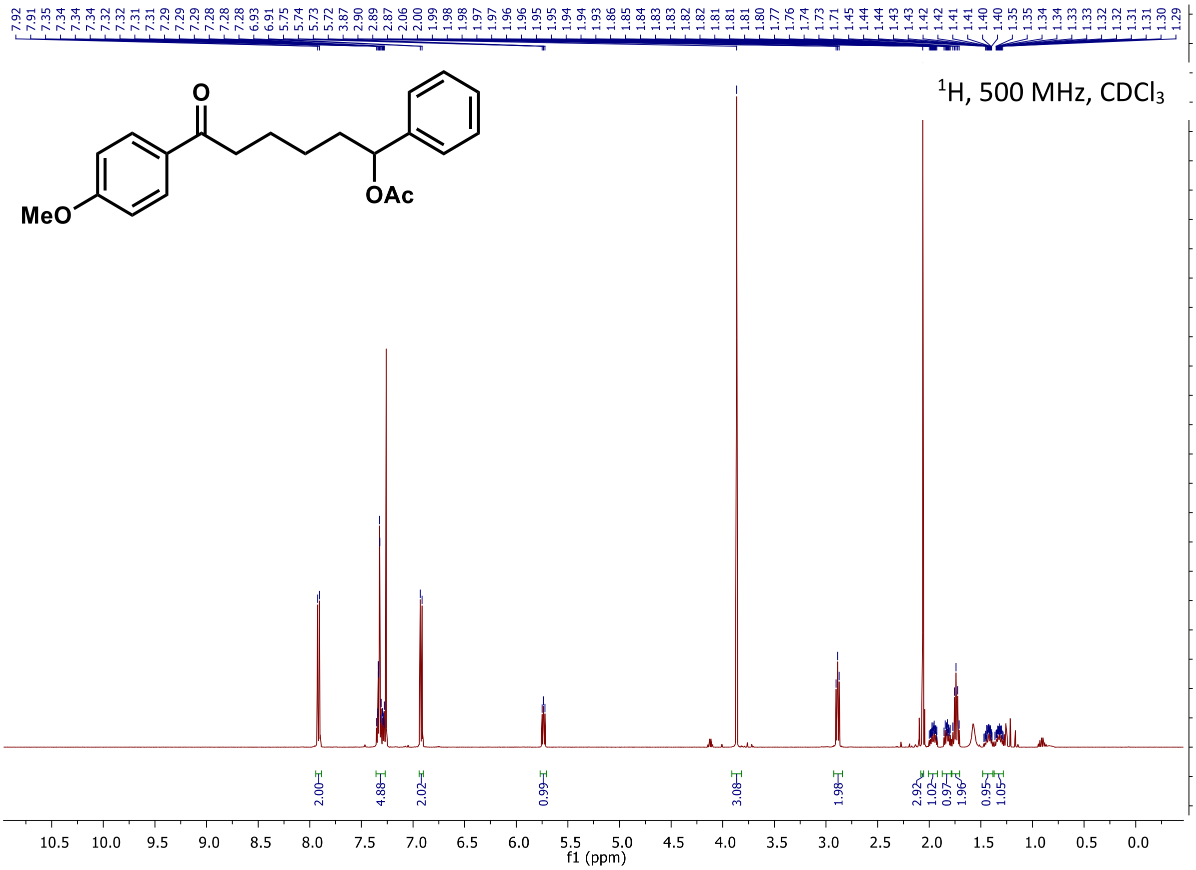

1H, 500 MHz, CDCl3
MeO
O
OAc
f1 (ppm)

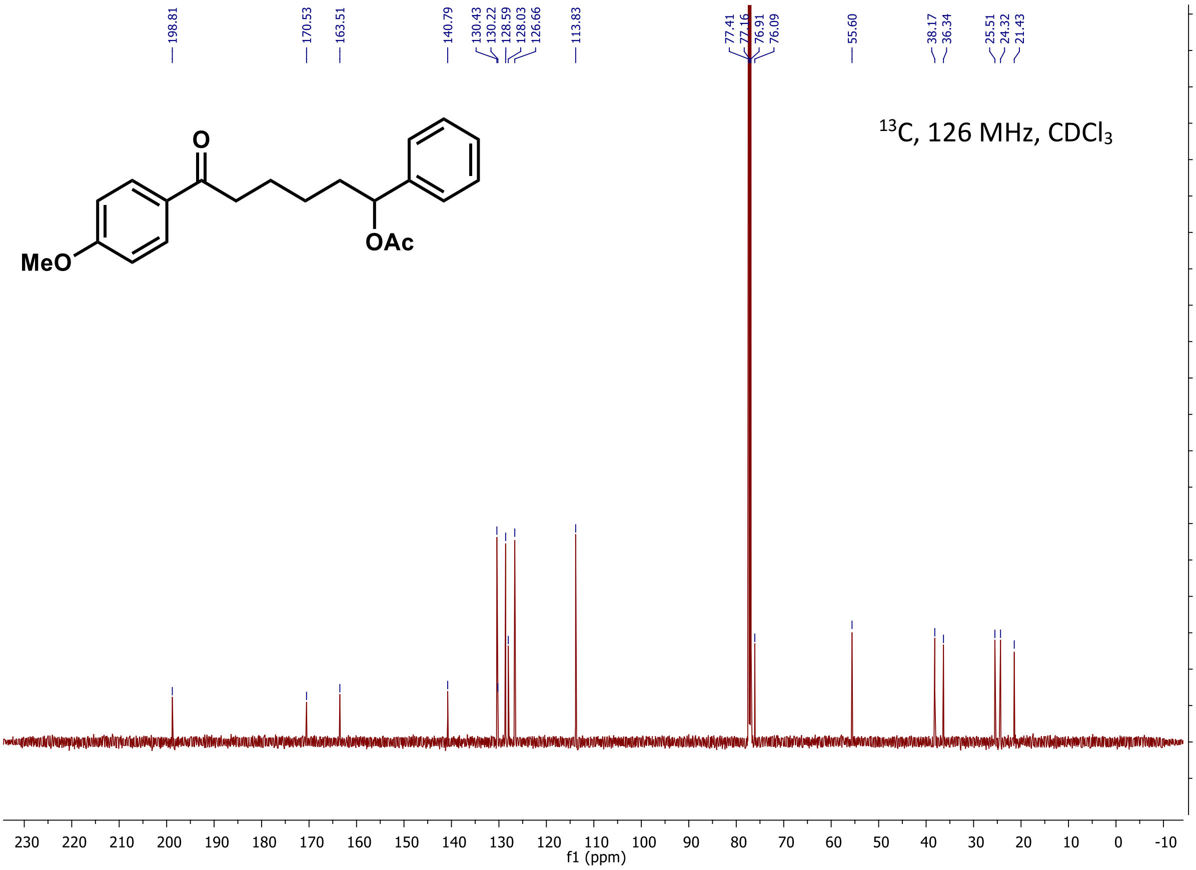

13C, 126 MHz, CDCl3
MeO
O
OAc
198.81
170.53
163.51
140.79
130.43
130.22
128.59
128.03
126.66
113.83
77.41
77.16
76.91
76.09
55.60
38.17
36.34
25.51
24.32
21.43
f1 (ppm)

(34)

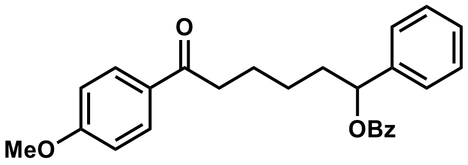

Prepared according to the General Procedure Y passing 2.0 *F* of charge using **1** (85.0 mg, 0.30 mmol, 1 equiv.), *n*-Bu_4NOBz (218 mg, 0.60 mmol, 2 equiv.), dichloromethane (5.8 mL) and Benzoic Acid (0.25 g). The yield was determined by crude ^{1}H NMR using 1,3,5-trimethylbenzene (42 µL, 1 equiv.) as an internal standard: 40%. Purification by flash column chromatography (eluent = 5-10% EtOAc in hexanes, silica gel) to afford product as a yellow oil (43.5 mg, 36% yield).

R_f = 0.29 (eluent = 20% EtOAc in hexanes); **ν_{max} / cm^{-1}** (thin film) 2980, 1701, 1673, 1600, 1508, 1450, 1251, 1171 ; **^{1}H NMR (500 MHz, $CDCl_3$)** δ_H: 1.36-1.47 (1H, m), 1.48-1.57 (1H, m), 1.78 (2H, pent, *J* 7.6 Hz), 1.90-2.04 (1H, m), 2.05-2.18 (1H, m), 2.83-2.95 (2H, m), 3.86 (3H, s), 6.00 (1H, dd, *J* 7.7, 6.0 Hz), 6.91 (2H, d, *J* 9.0 Hz), 7.27-7.31 (1H, m), 7.33-7.36 (2H, m), 7.39-7.46 (4H, m), 7.53-7.57 (1H, m), 7.91 (2H, d, *J* 9.0 Hz), 8.05-8.10 (2H, m); **^{13}C NMR (126 MHz, $CDCl_3$)** δ_C: 24.4, 25.6, 36.6, 38.2, 55.6, 76.7, 113.8, 126.6, 128.1, 128.5, 128.6, 129.8, 130.2, 130.4, 130.6, 133.1, 140.9, 163.5, 166.0, 198.8; **HRMS (ES$^+$)** [$C_{26}H_{26}O_4$] requires $[M+Na]^+$ 425.1729, found 425.1731 (+ 0.5 ppm).

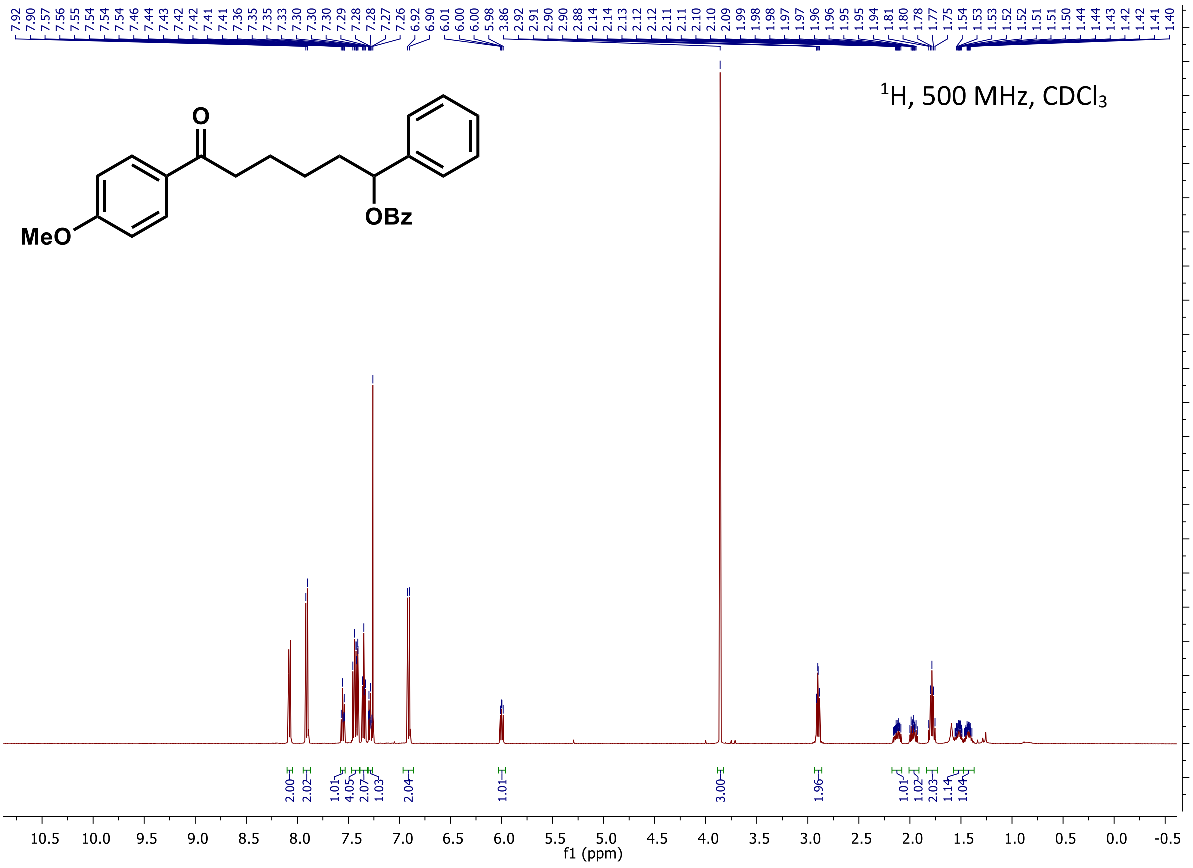

1H, 500 MHz, CDCl3
O
MeO
OBz
f1 (ppm)

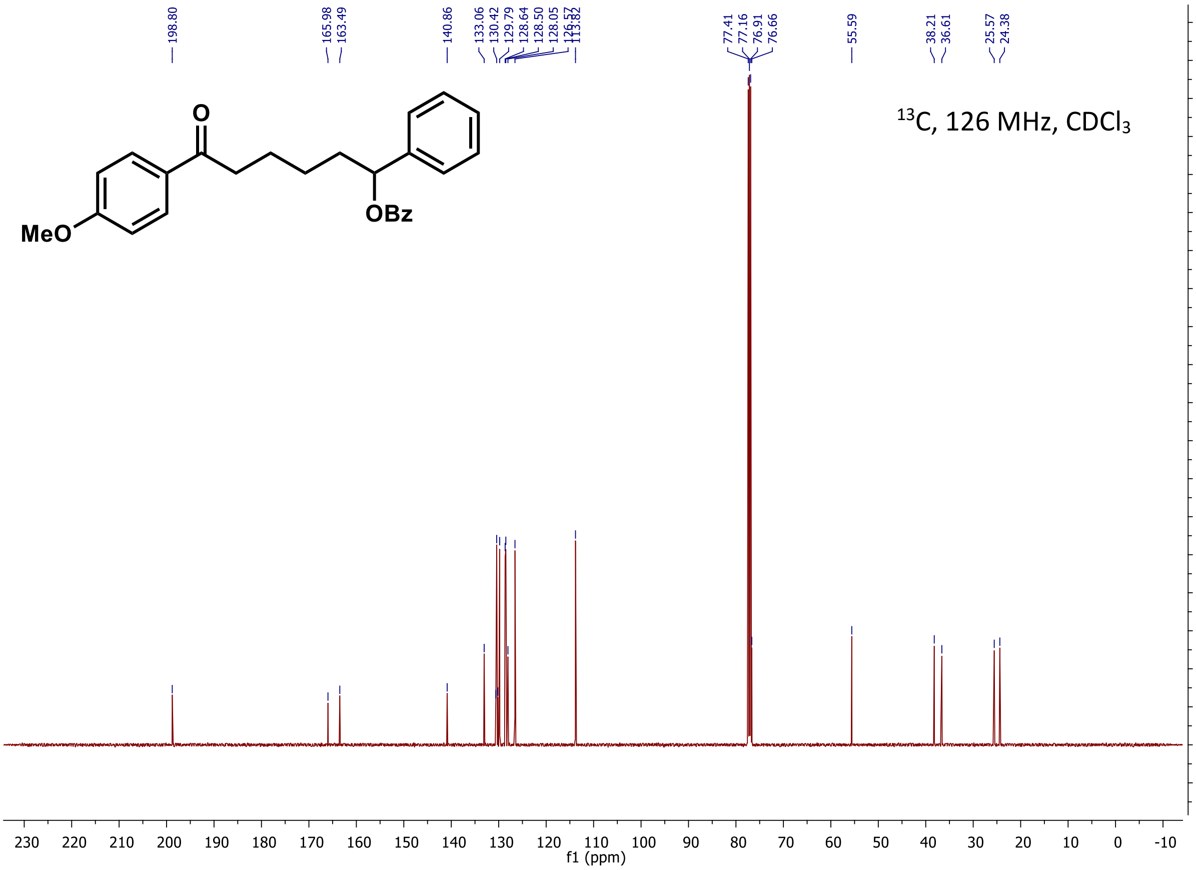

13C, 126 MHz, CDCl3
O
MeO
OBz
f1 (ppm)

(35)

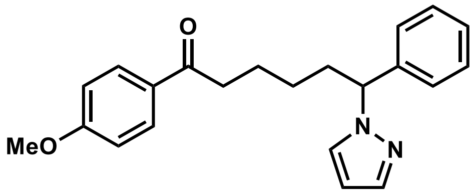

Prepared according to the General Procedure Y using **1** (85.0 mg, 0.30 mmol, 1 equiv.), *n*-Bu_4NPF_6 (232 mg, 0.60 mmol, 2 equiv.), 1*H*-pyrazole (40.5 mg, 0.60 mmol, 2 equiv.), dichloromethane (5.7 mL) and TFE (0.3 mL). The yield was determined by crude ^{1}H NMR using 1,3,5-trimethylbenzene (42 µL, 1 equiv.) as an internal standard: 54%. Purification by flash column chromatography (eluent = 30 to 50% EtOAc in hexanes, silica gel) to afford product as a brown oil (52.3 mg, 50% yield).

R_f = 0.29 (eluent =20% EtOAc in hexanes); **v_{max} / cm^{-1}** (thin film) 2935, 1662, 1600, 1390, 1260, 1165, 1028, 761, 700; **^{1}H NMR (500 MHz, $CDCl_3$)** δ_H: 1.29 - 1.46 (2H, m), 1.71-1.78 (2H, m), 1.88-1.93 (2H, m), 2.87-2.90 (2H, m), 3.86 (3H, s), 4.56-4.59 (1H, m), 6.35-6.36 (1H, dd, *J* 2.6, 1.6 Hz), 6.90-6.93 (2H, m), 7.21-7.25 (1H, m), 7.29-7.33 (2H, m), 7.35-7.37 (2H, m), 7.61 (1H, dd, *J* 1.5, 0.6 Hz), 7.90-7.92 (2H, m), 8.47-8.48 (1H, dd, *J* 2.6, 0.6 Hz); **^{13}C NMR (126 MHz, $CDCl_3$)** δ_C: 13.6, 24.5, 26.4 , 38.3, 39.7, 55.6, 63.7, 107.5, 113.8, 126.8, 128.6, 127.1, 130.2, 130.4, 141.1, 144.4, 163.5, 199.0; **HRMS (EI)** [$C_{22}H_{24}O_2N_2$] requires [M-H] 347.1760, Found 347.1750 (- 2.9 ppm).

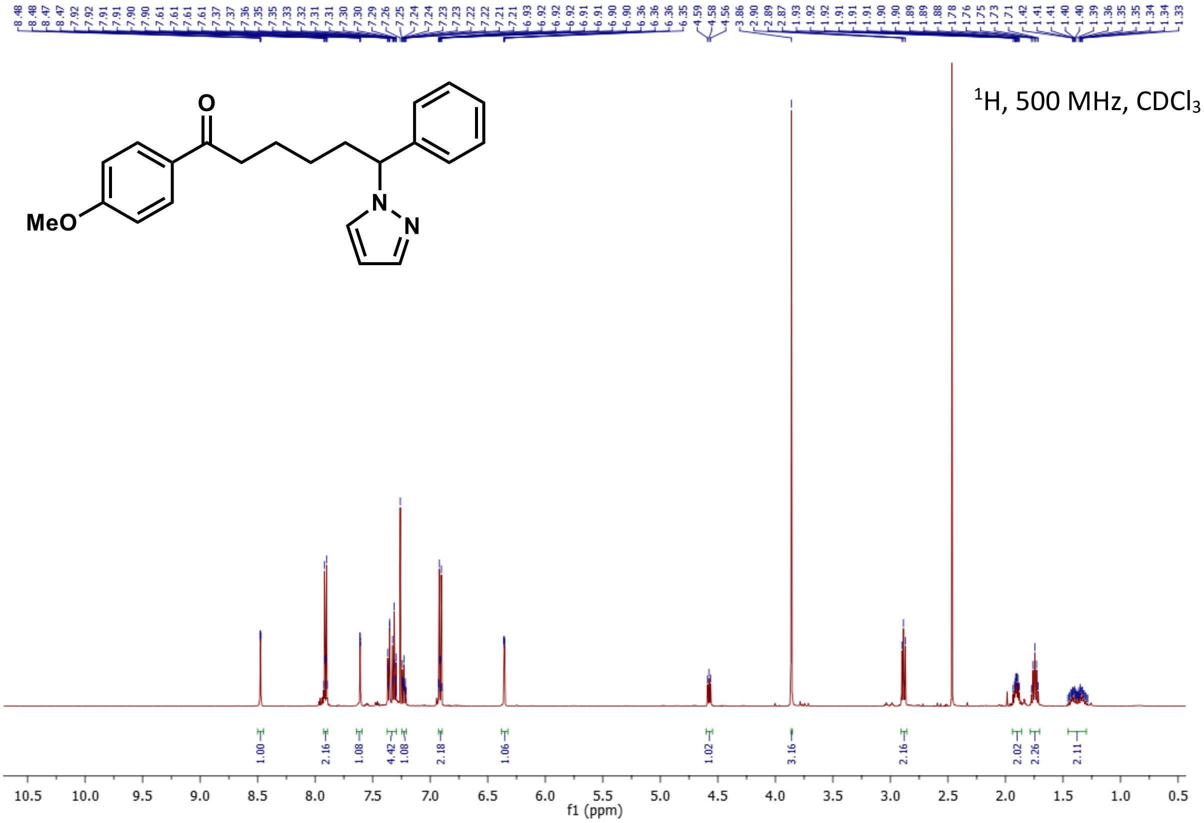

^{1}H, 500 MHz, $CDCl_3$
MeO
O
N
N
f1 (ppm)

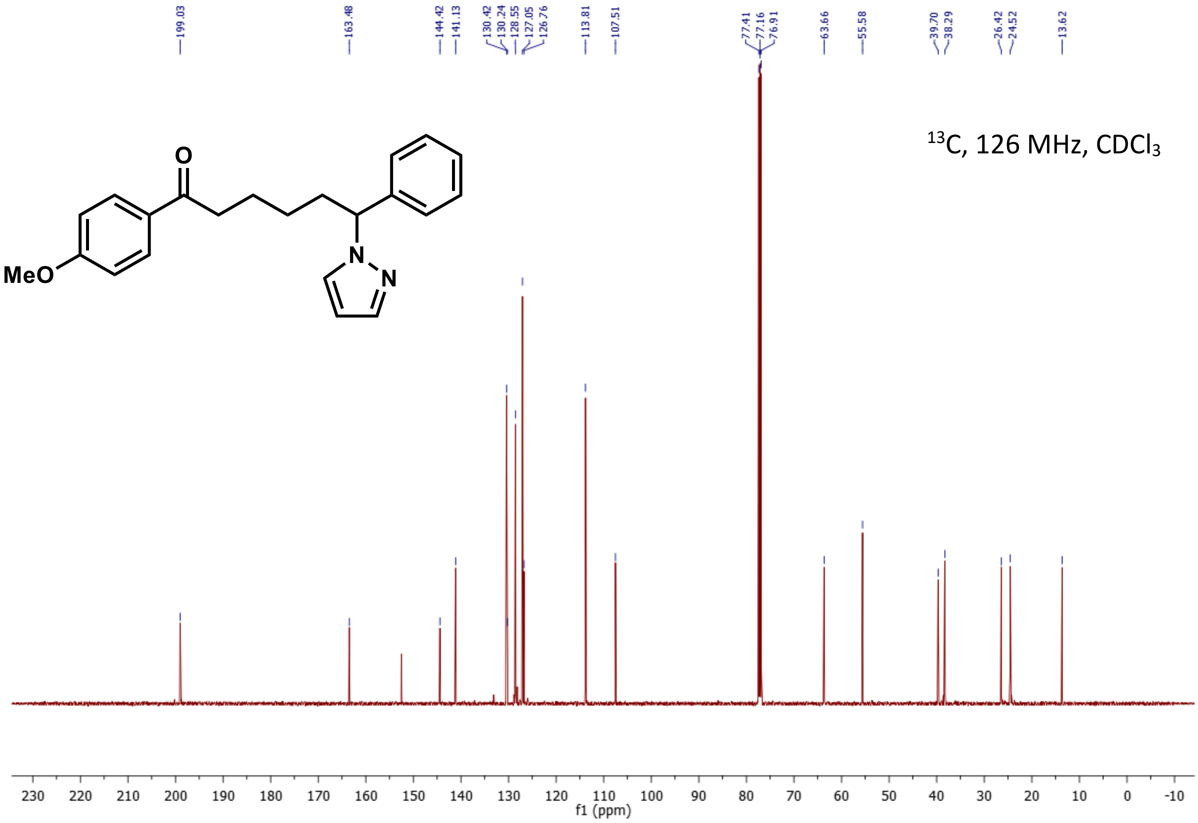

^{13}C, 126 MHz, $CDCl_3$
MeO
O
N
N
f1 (ppm)

(36)

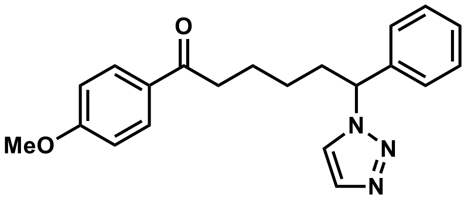

Prepared according to the General Procedure Y using **1** (85.0 mg, 0.30 mmol, 1 equiv.), *n*-Bu_4NPF_6 (232 mg, 0.60 mmol, 2 equiv.), 1*H*-1,2,3-triazole (41.4 mg, 0.60 mmol, 2 equiv.), dichloromethane (5.7 mL) and TFE (0.3 mL). The yield was determined by crude ^{1}H NMR using 1,3,5-trimethylbenzene (42 µL, 1 equiv.) as an internal standard: 53%. Product was formed as an 80:20 (N1:N2) mixture of regioisomers. Purification and isolation by silica gel chromatography proved challenging due to inseparable impurities.

HRMS (EI) [$C_{21}H_{23}O_2N_3$] requires [M-H] 348.1712, Found 348.1702 (- 2.9 ppm).

Resolved signals for major regioisomer:

^{1}H NMR (500 MHz, $CDCl_3$) δ_H: 7.63 (1H, d, *J* 1.2 Hz), 8.42 (1H, d, *J* 1.2 Hz)

Resolved signals for minor regioisomer:

^{1}H NMR (500 MHz, $CDCl_3$) δ_H: 7.72 (2H, m)

Resolved signals for mixture of regioisomers:

^{1}H NMR (500 MHz, $CDCl_3$) δ_H: 2.81-2.85 (2H, m), 4.57-4.62 (1H, m), 6.84-6.86 (2H, m), 7.82-7.83 (2H, m)

Regioisomer Identification:

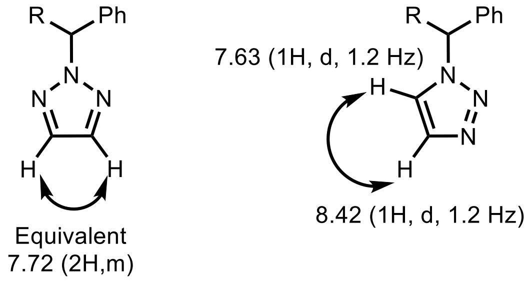

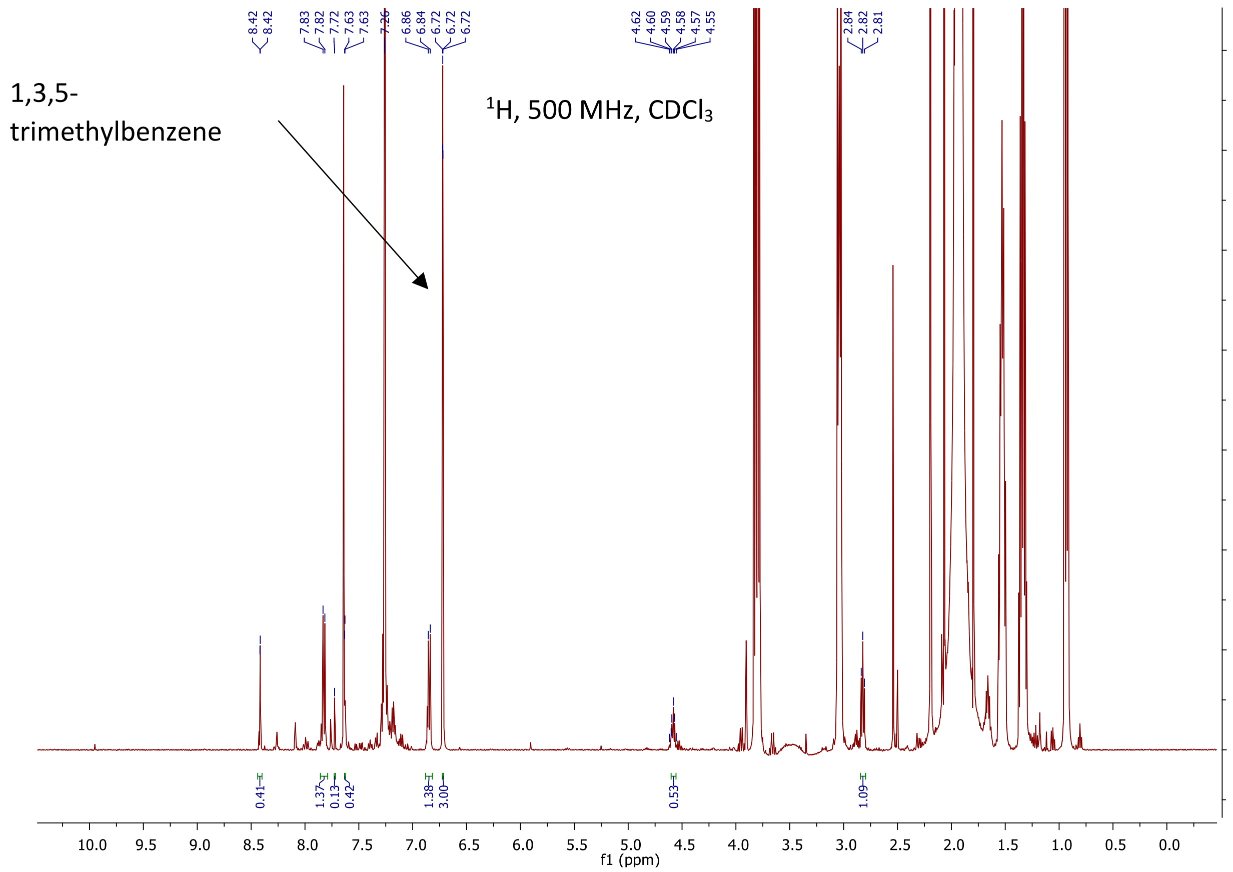
1,3,5-trimethylbenzene
^{1}H, 500 MHz, CDCl$_3$
f1 (ppm)

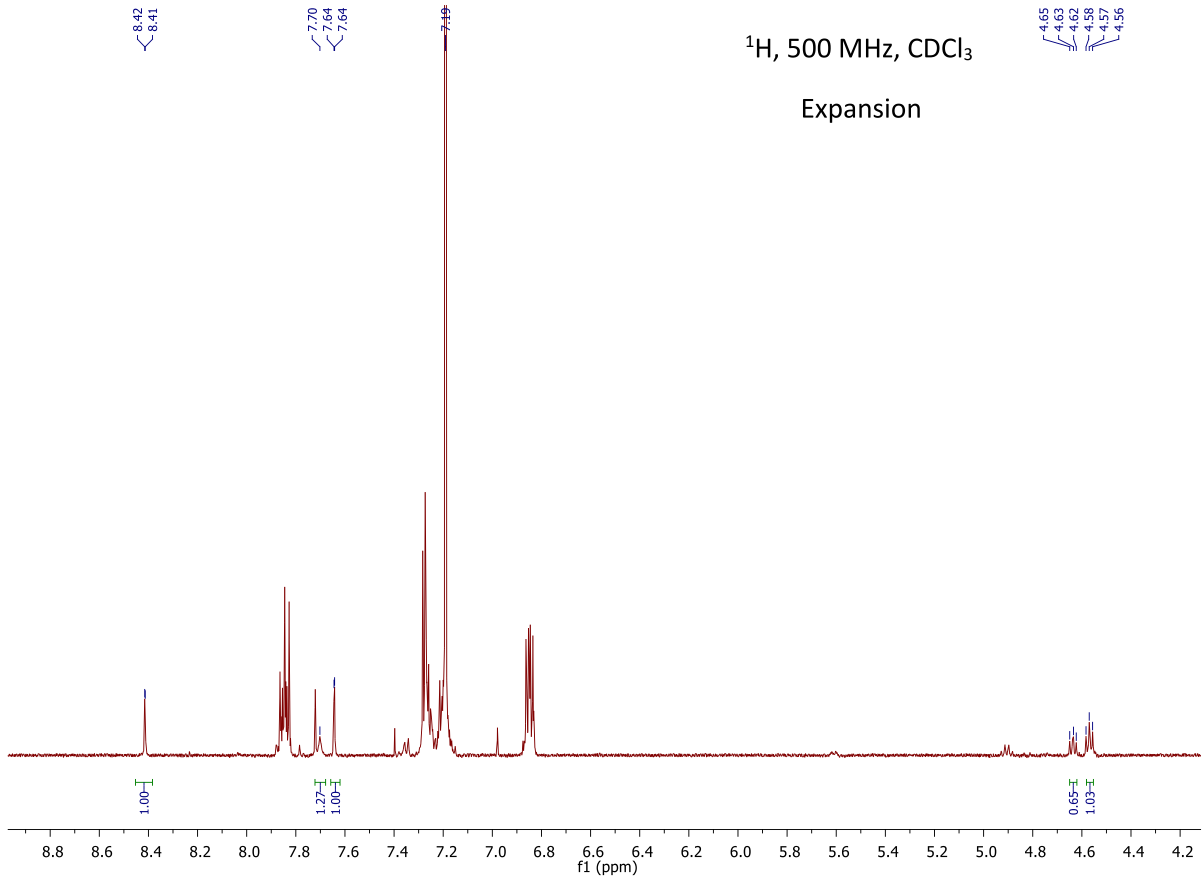
^{1}H, 500 MHz, CDCl$_3$
Expansion
f1 (ppm)

Mechanistic Studies:

(37)

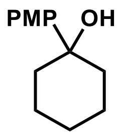

Prepared according to a literature procedure.[7]

(39)

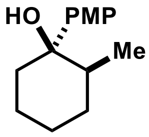

Prepared according to General Procedure A part B(1) using magnesium turnings (656 mg, 27 mmol), 4-bromoanisole (2.80 mL, 22.5 mmol), and 2- methylcyclohexanone (1.55 mL, 15 mmol). The crude residue was purified by flash column chromatography (5 to 10% EtOAc/Petrol, silica gel) to afford product as a colourless oil (2.15 g, 65% yield)

R_f = 0.27 (eluent = 10% EtOAc in hexanes); **^{1}H NMR (500 MHz, $CDCl_3$)** δ_H: 0.63 (3H, d, *J* 6.8 Hz), 1.32-1.52 (2H, m), 1.55-1.69 (3H, m), 1.69-1.75 (2H, m), 1.75-1.82 (1H, m), 1.84-1.95 (1H, m), 3.80 (3H, s), 6.87 (2H, d, *J* 9.0 Hz), 7.36 (2H, d, *J* 9.0 Hz); **^{13}C NMR (126 MHz, $CDCl_3$)** δ_C: 15.7, 22.3, 26.3, 30.6, 40.1, 41.5, 55.4, 75.6, 113.5, 125.9, 140.8, 158.1.

Consistent with literature data.[7]

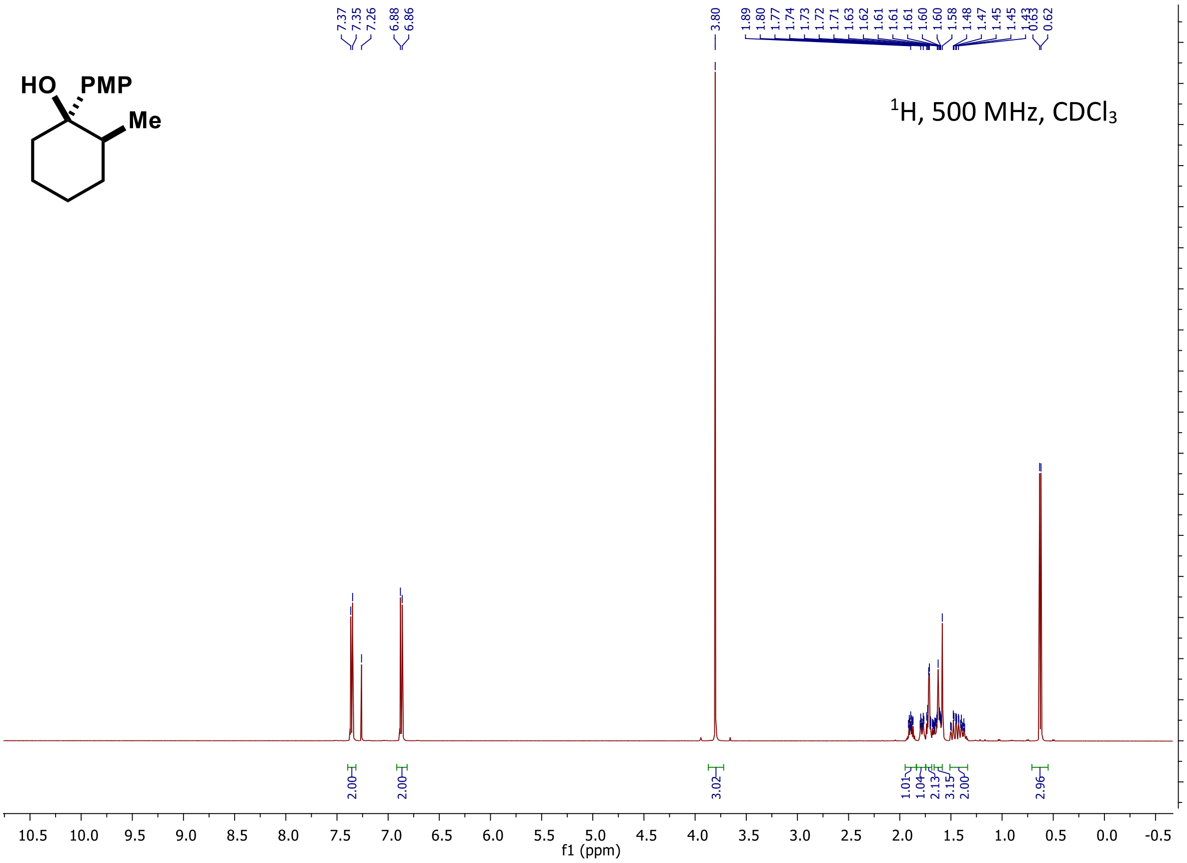
HO
PMP
Me
1H, 500 MHz, CDCl3
f1 (ppm)

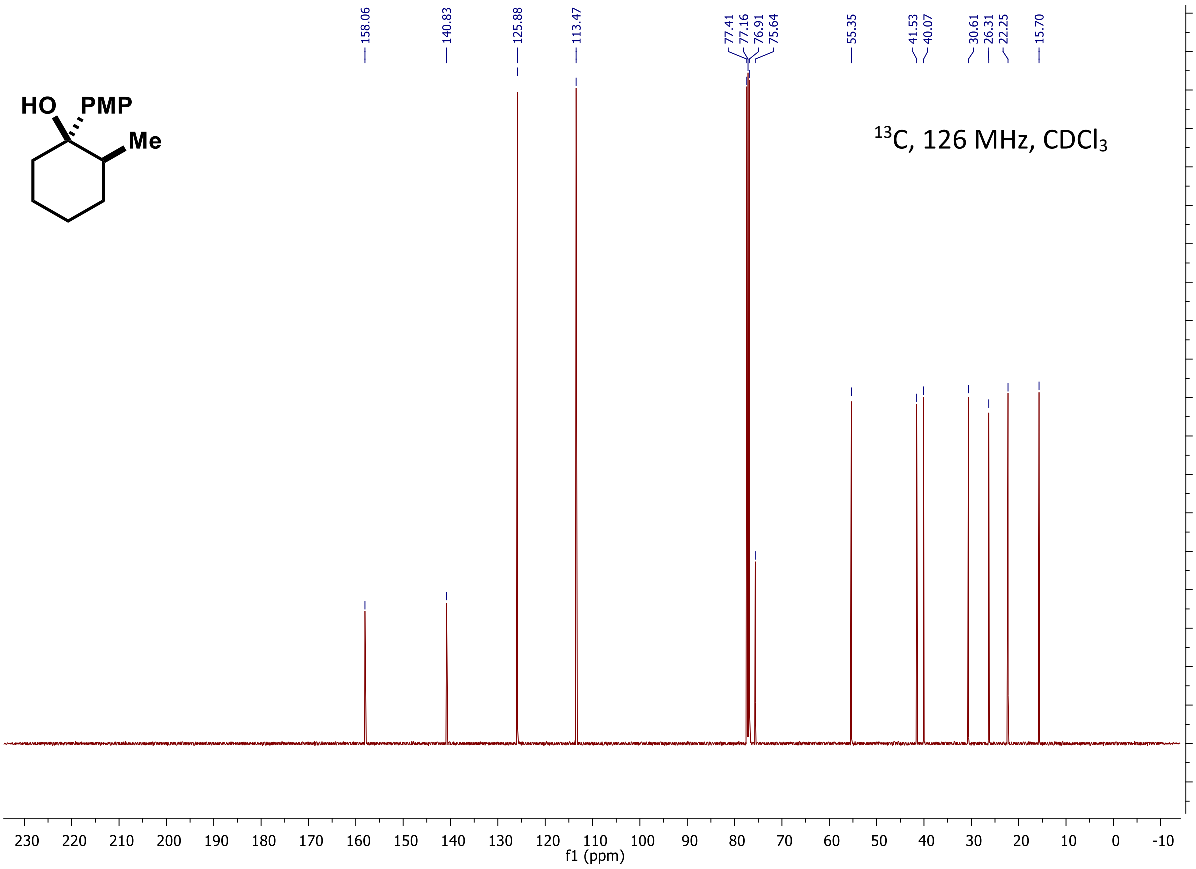
HO
PMP
Me
13C, 126 MHz, CDCl3
f1 (ppm)

(40)

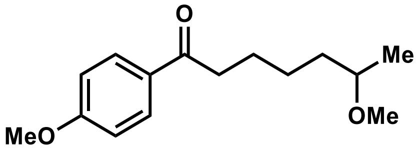

Prepared according to the General Procedure Y using 1-(4-methoxyphenyl)-2-methylcyclohexan-1-ol (66.0 mg, 0.30 mmol, 1 equiv.), *n*-Bu_4NPF_6 (232 mg, 0.60 mmol, 2 equiv.), dichloromethane (4.5 mL) and MeOH (1.5 mL). The yield was determined by crude ^{1}H NMR using 1,3,5-trimethylbenzene (42 µL, 1 equiv.) as an internal standard: 64% of ring opened aryl ketones. Purification and isolation by silica gel chromatography proved challenging due to inseparable impurities. Purification by flash column chromatography (eluent = 5 to 15% EtOAc in hexanes, silica gel) to afford product as a colourless oil (12.1 mg, 16% yield).

R_f = 0.24 (eluent = 20% EtOAc in hexanes); **ν_{max} / cm^{-1}** (thin film) 2932, 1678, 1599, 1258, 1169; **^{1}H NMR (500 MHz, $CDCl_3$)** δ_H: 1.13 (3H, d, *J* 6.1 Hz), 1.37-1.50 (3H, m), 1.55-1.62 (1H, m), 1.68-1.80 (2H, m), 2.89-2.95 (2H, m), 3.27-3.34 (4H, m), 3.87 (3H, s), 6.93 (2H, d, *J* 9.0 Hz), 7.94 (2H, d, *J* 9.0 Hz); **^{13}C NMR (126 MHz, $CDCl_3$)** δ_C: 19.2, 24.8, 25.4, 36.4, 38.4, 55.6, 56.1, 76.8, 113.8, 130.3, 130.5, 163.5, 199.2; **HRMS (ES$^+$)** [$C_{15}H_{22}O_3$] requires $[M+Na]^+$ 273.1467, found 273.1461 (- 2.2 ppm).

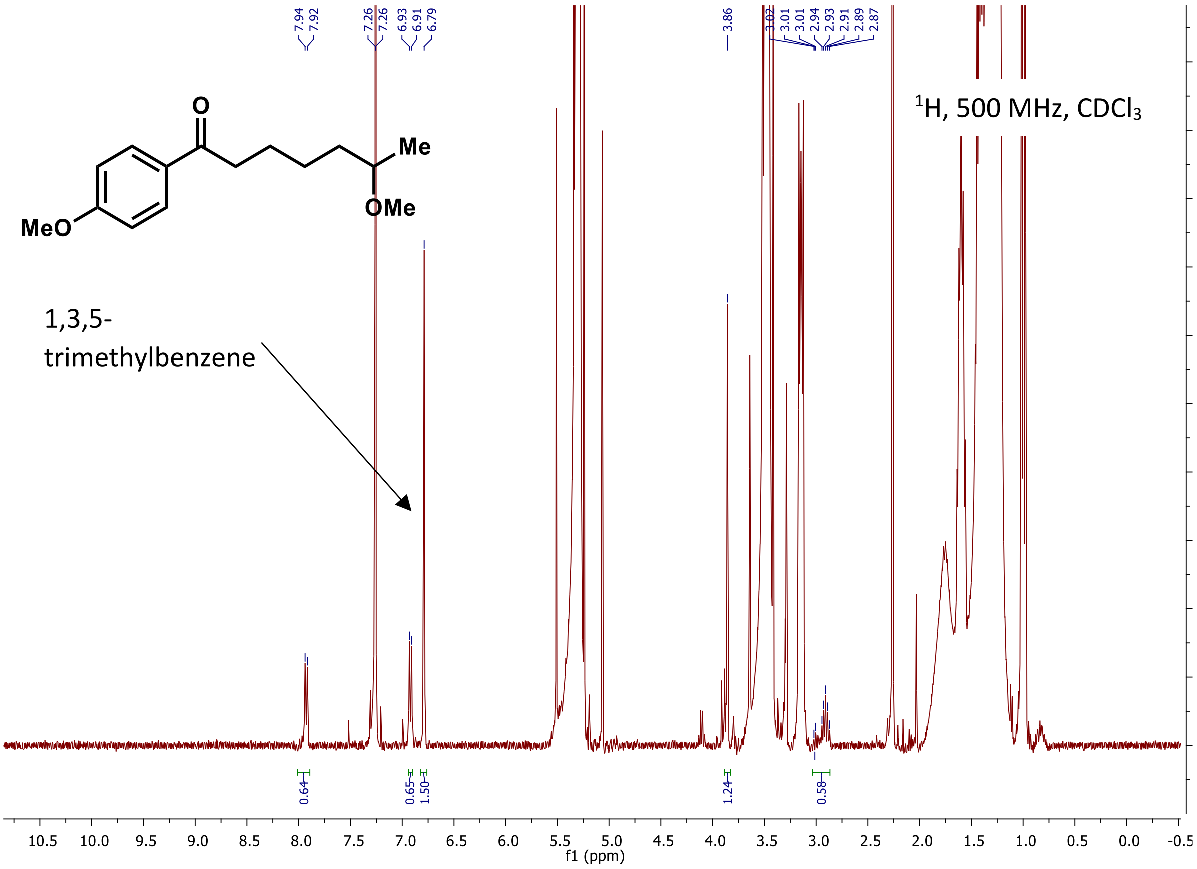
7.94
7.92
7.26
7.26
6.93
6.91
6.79
3.86
3.01
3.01
2.94
2.93
2.91
2.89
2.87
O
Me
OMe
MeO
¹H, 500 MHz, CDCl₃
1,3,5-trimethylbenzene
0.64
0.65
1.50
1.24
0.58
f1 (ppm)

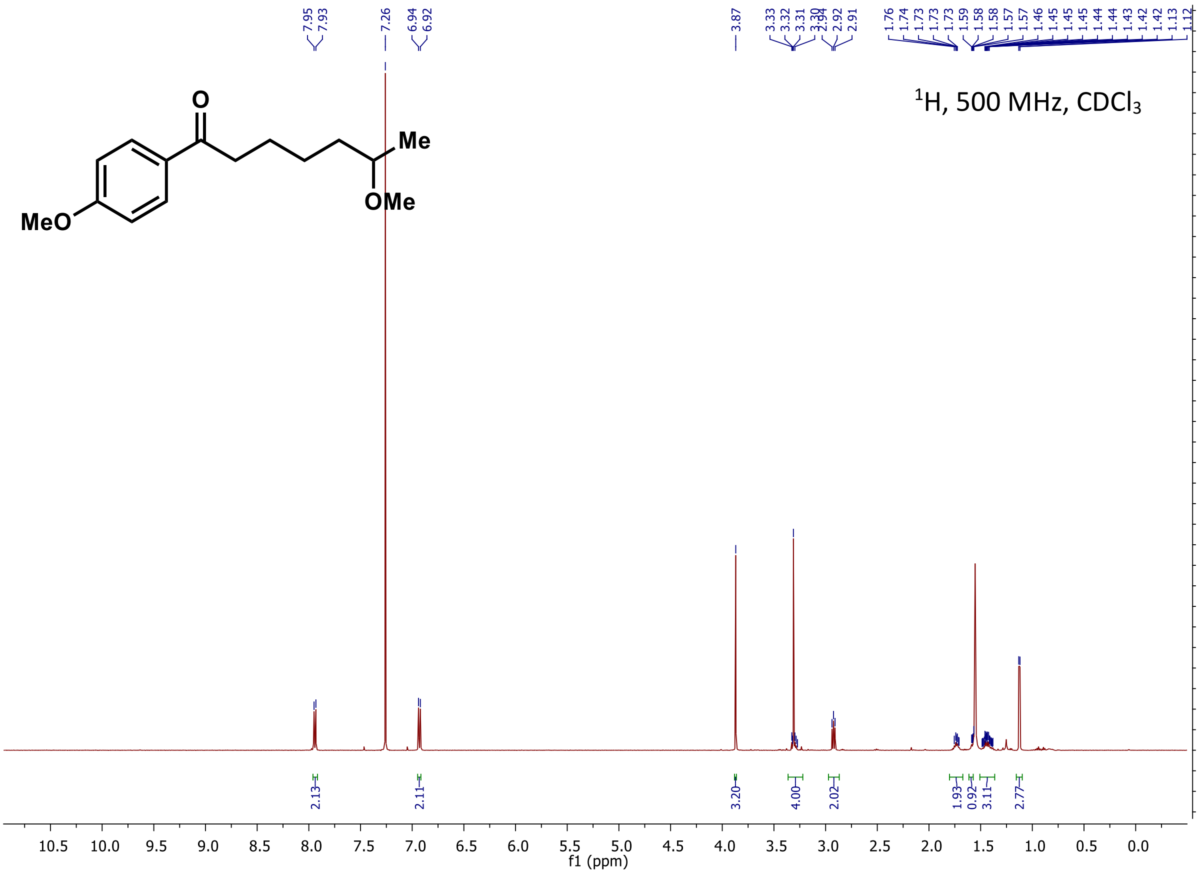
7.95
7.93
7.26
6.94
6.92
3.87
3.33
3.32
3.31
2.92
2.91
1.76
1.74
1.73
1.73
1.73
1.59
1.58
1.58
1.57
1.57
1.46
1.45
1.45
1.44
1.44
1.43
1.42
1.42
1.13
1.12
O
Me
OMe
MeO
¹H, 500 MHz, CDCl₃
2.13
2.11
3.20
4.00
2.02
1.93
0.92
3.11
2.77
f1 (ppm)

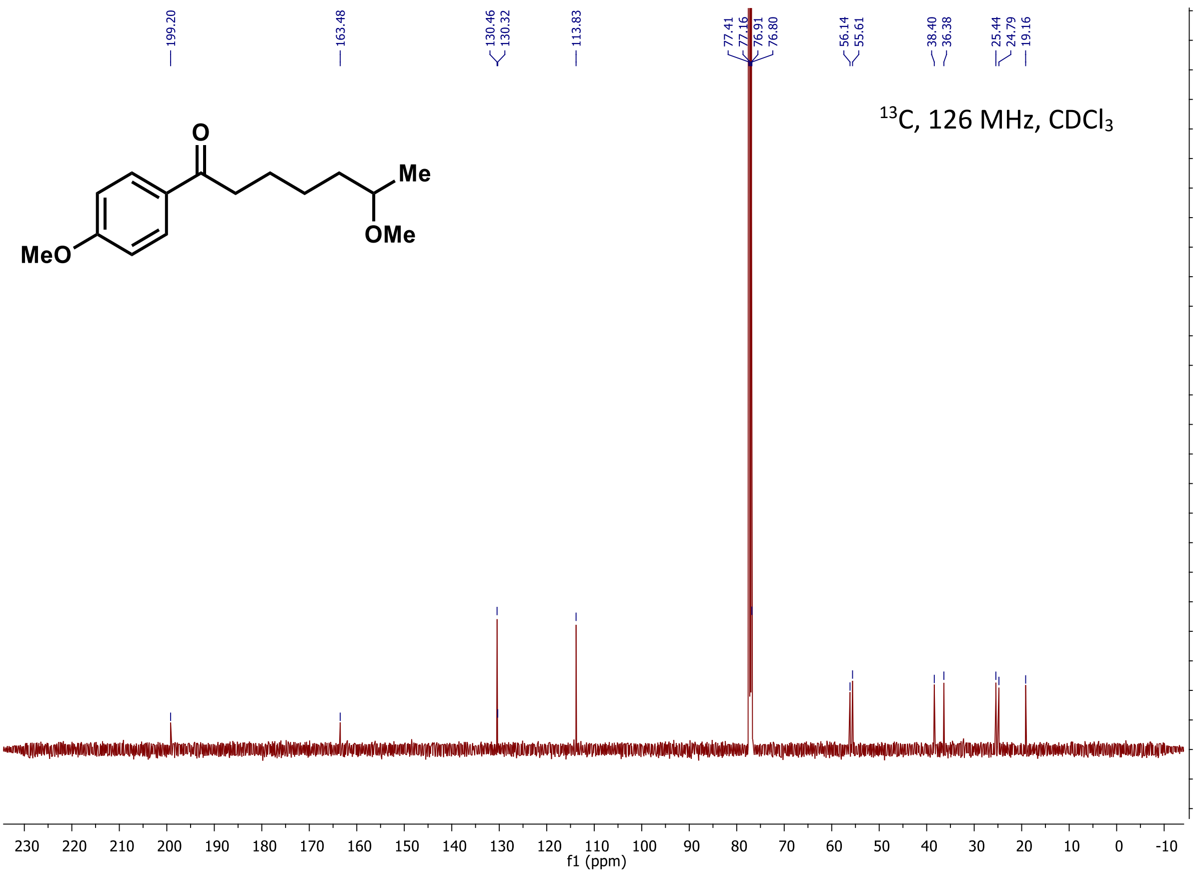

199.20
163.48
130.46
130.32
113.83
77.41
77.16
76.91
76.80
56.14
55.61
38.40
36.38
25.44
24.79
19.16
^{13}C, 126 MHz, CDCl$_3$
O
Me
MeO
OMe
230 220 210 200 190 180 170 160 150 140 130 120 110 100 90 80 70 60 50 40 30 20 10 0 -10
f1 (ppm)

(41)

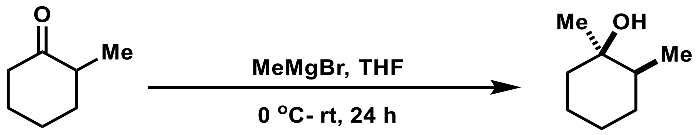

Prepared according to General Procedure B (1) using 2-methylcyclohexanone (2.24 g, 20 mmol), THF (40 mL) and methylmagnesium bromide (10 mL, 30 mmol, 3M in Et_2O). The crude residue was purified by flash column chromatography (eluent = 5 % EtOAc in hexanes, silica gel) to afford product as a colourless oil (734 mg, 29% yield).

R_f = 0.25 (eluent = 10% EtOAc in hexanes); **ν_{max} / cm^{-1}** (thin film) 3481-3423 (br), 2930; **^{1}H NMR (500 MHz, $CDCl_3$)** δ_H: 0.90 (3H, dd, *J* 6.6, 0.6 Hz), 1.16-1.19 (3H, m), 1.20-1.31 (2H, m), 1.31-1.48 (3H, m), 1.47-1.56 (2H, m), 1.56-1.71 (3H, m); **^{13}C NMR (126 MHz, $CDCl_3$)** δ_C: 15.3, 22.2, 26.1, 28.8, 30.8, 40.1, 40.4, 71.2; **HRMS (CI^+)** [$C_8H_{16}O$] requires $[M]^+$ 128.1196, found 128.1194 (-1.1 ppm).

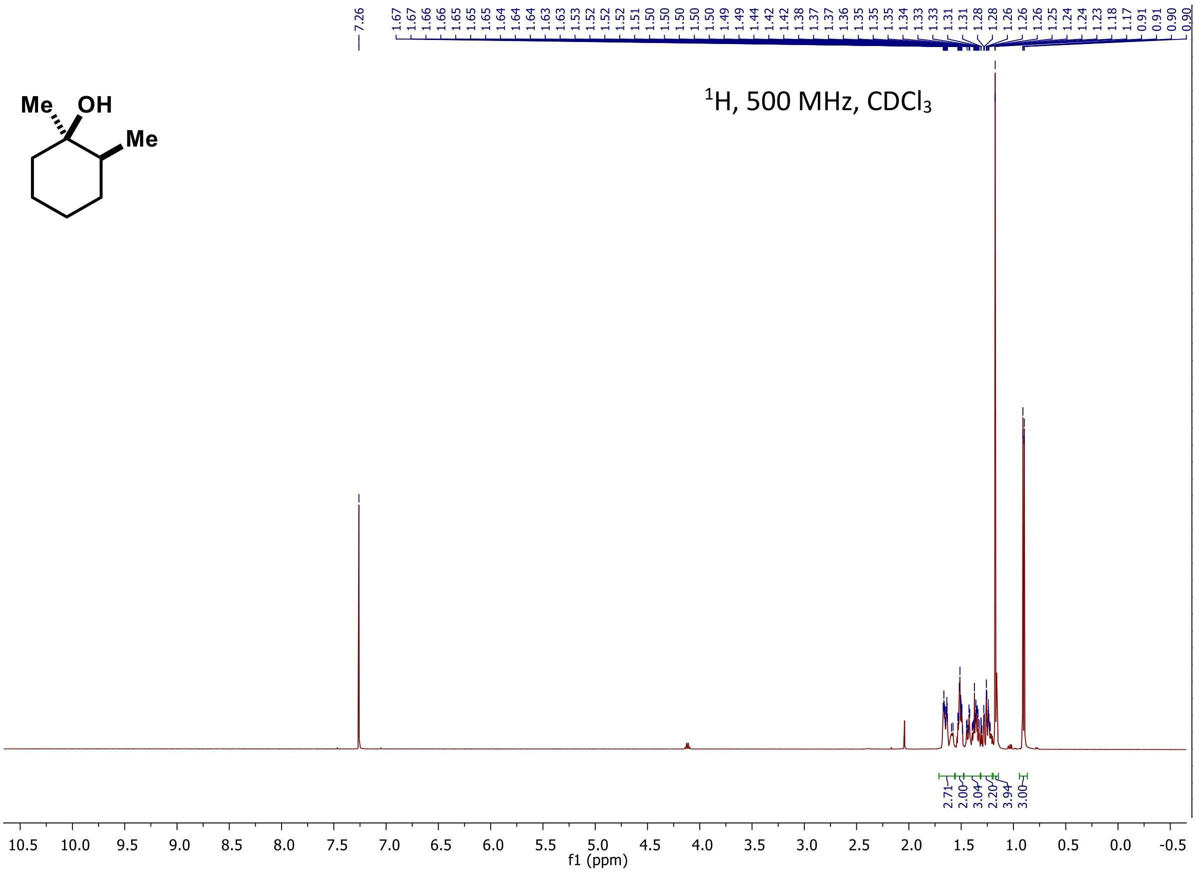

Me
OH
Me
^{1}H, 500 MHz, CDCl$_3$
f1 (ppm)

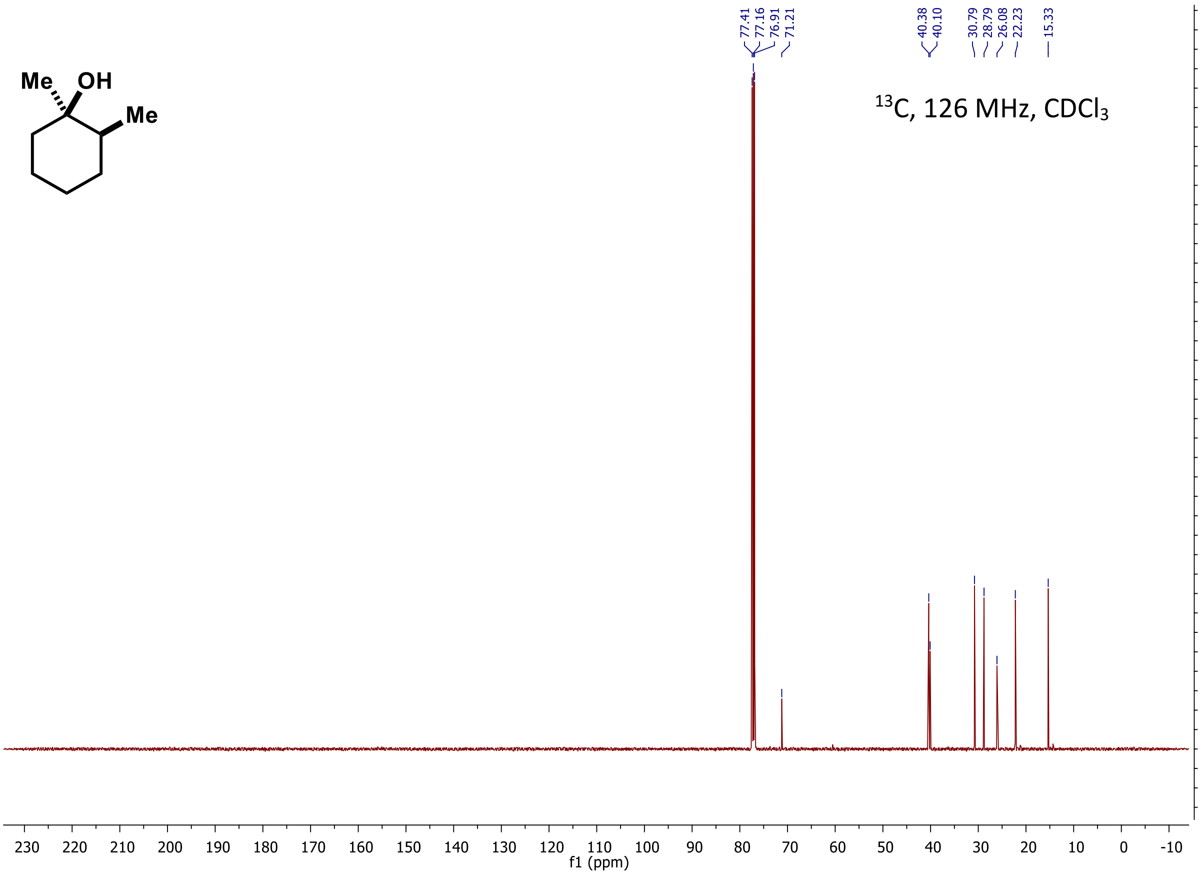

Me
OH
Me
^{13}C, 126 MHz, CDCl$_3$
77.41
77.16
76.91
71.21
40.38
40.10
30.79
28.79
26.08
22.23
15.33
f1 (ppm)

(43)

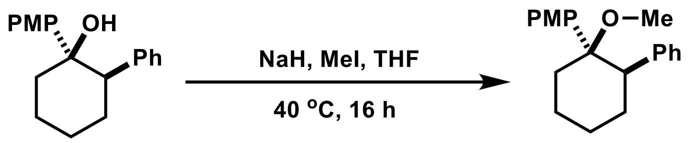

To a solution of 1-(4-methoxyphenyl)-2-phenylcyclohexan-1-ol (350 mg, 2 mmol, 1 equiv.) in THF (10 mL) at 0 °C was added sodium hydride (192 mg, 4.8 mmol, 2 equiv., 60 % in paraffin oil). The mixture was stirred at 0 °C for 15 minutes before methyl iodide (2.84 g, 20 mmol, 10 equiv.) was added and the mixture was warmed to room temperature, stirred for 3 h and then heated to 40 °C overnight. The mixture was then quenched with a saturated solution of ammonium chloride (5 mL) and extracted with EtOAc (3 x 10 mL). The organics were combined, dried over $MgSO_4$, filtered and concentrated *in vacuo* affording crude product as a yellow oil. The crude residue was purified by flash column chromatography (eluent = 10 to 20% EtOAc in hexanes, silica gel) to afford product as a colourless oil (90 mg, 15% yield).

R_f = 0.62 (eluent = 20% EtOAc in hexanes); **ν_{max} / cm^{-1}** (thin film) 2931, 1608, 1508, 1442, 1244, 1178; **^{1}H NMR (500 MHz, $CDCl_3$)** δ_H: 1.42-1.54 (1H, m), 1.62-1.70 (1H, m), 1.68-1.79 (2H, m), 1.88-1.97 (1H, m), 2.02-2.11 (1H, m), 2.17-2.23 (1H, m), 2.27-2.39 (1H, m), 2.50 (1H, dd, *J* 12.8, 3.4 Hz), 3.06 (3H, s), 3.72 (3H, s), 6.61 (2H, d, *J* 9.0 Hz), 6.77 (2H, d, *J* 9.0 Hz), 6.84-6.88 (2H, m), 7.01-7.09 (3H, m); **^{13}C NMR (126 MHz, $CDCl_3$)** δ_C: 21.7, 26.9, 29.3, 31.7, 49.6, 55.2, 56.9, 80.0, 112.7, 125.8, 127.1, 128.0, 130.0, 135.9, 143.0, 158.0; **HRMS (ES^+)** [$C_{20}H_{24}O_2$] requires $[M]^+$ 296.1776, found 296.1775 (-0.30 ppm).

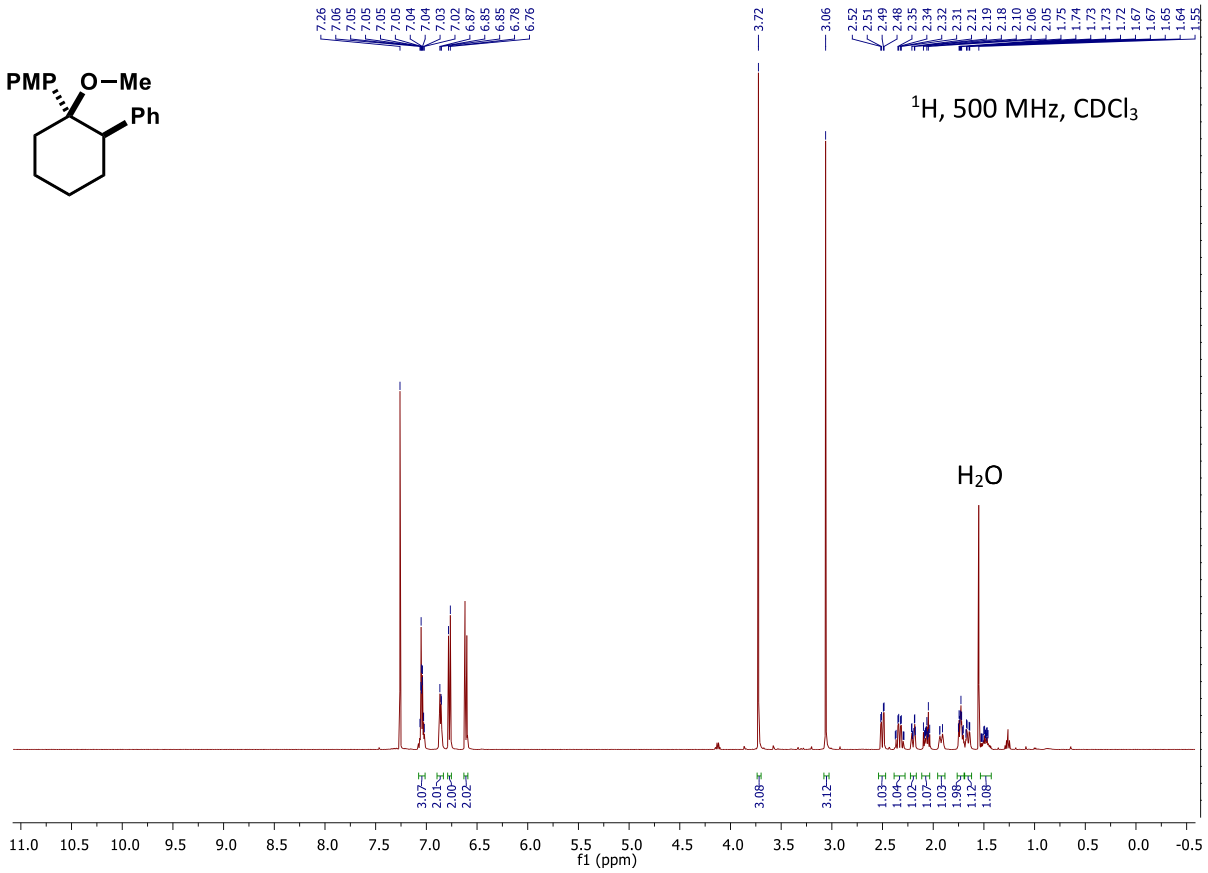
PMP
O−Me
Ph
1H, 500 MHz, CDCl3
H2O
f1 (ppm)

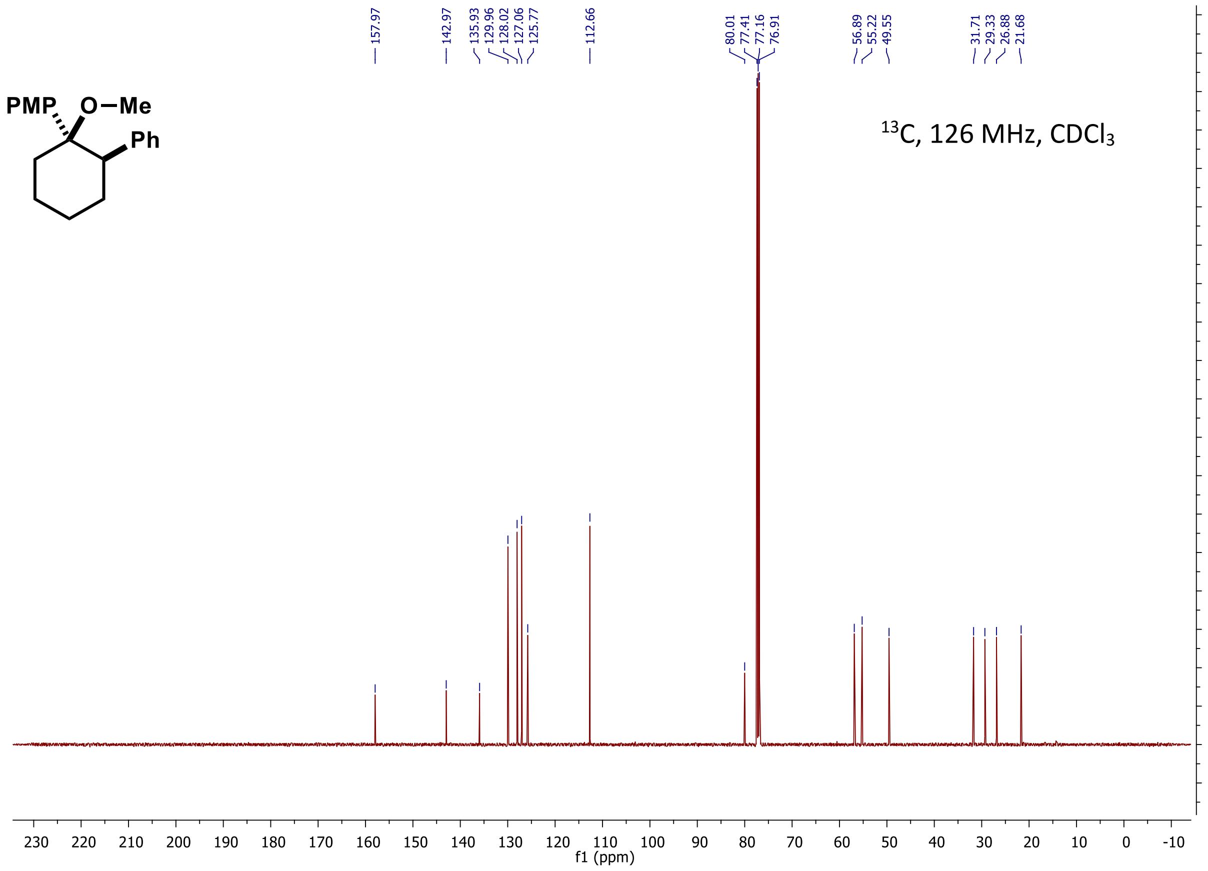
PMP
O−Me
Ph
13C, 126 MHz, CDCl3
f1 (ppm)

Electrochemical Flow Scale Up

General Information

The flow set-up used PFA tubing with a 0.79 ± 0.1 mm internal diameter and 1.58±0.1 mm outer diameter supplied by Polyflon. All flow fittings and connections were purchased from Kinesis (Gripper fitting nuts, part number: 002103; Adapters, part number: P-618; Omnilok type-p fitting ferrule, part number: 008FT16; Y-Connector, part number: P-512; Threaded union, part number: P-623. The syringe pumps used was the the Chemyx Fusion 100 syringe pump.

The power supply used was a Voltcraft LRP-1205 that supplied DC to the electrochemical system. The electrochemical flow cell was purchased from Cambridge Reactor Design, the Ammonite 8 (part number: 74660). The electrochemical flow cell consists of 1 carbon/PVDF electrode and one platinum electrode that are fixed either side of a FFKM gasket with a channel groove length of 1000 mm and an internal volume of 2.5 mL, of which 1 mL is exposed to the electrodes. The inlet and outlet fittings of the ammonite 8 cell were modified from 1/16" ID to accommodate 1/32" ID PFA tubing using Swagelok reducers, nuts and ferrules. The ammonite reactor was cell was dismantled for electrode cleaning every 2 passes. The graphite electrode was cleaned with acetone, water, acetone rinses and then the surface regenerated by rubbing with silica gel and cotton wool to remove surface contaminants. The platinum electrode was cleaned with acetone, water acetone rinses, left to dry and then burned with a blow torch.

The electrochemical flow system was used only in single pass electrolysis. Recirculating flow was not examined.

Gram Scale Flow Experimental

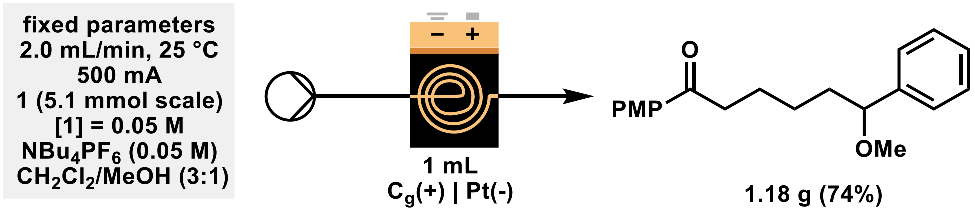

A 250 mL round-bottom flask was flame dried and charged with 1-(4-methoxyphenyl)-2-phenylcyclohexan-1-ol **1** (1.55 g, 5.5 mmol, 1 equiv.) and NBu_4PF_6 (2.13 g, 5.5 mmol, 0.05 M) and sealed with a Suba-seal. The reagent flask was evacuated on a Schlenk line and back filled with nitrogen gas for three cycles. Dry CH_2Cl_2 (83 mL) and MeOH (27 mL) were then added. After deoxygenating, (bubbling with nitrogen for 5 min) the mixture was drawn up into two 60 mL syringes (2 x 55 mL) and loaded onto a syringe pump.

The ammonite electrochemical flow reactor set up with a graphite anode and platinum cathode, was flushed with nitrogen gas for 5 min by connecting the inlet tubing directly to a dry nitrogen line. The reagent solution in 2 x 60 mL syringes were then connected to the flow set up via a T-piece. The syringe pumps were set to 1 mL/min, giving a combined total flow rate of 2 mL/min at the mixing-tee. The electrochemical set up was primed by pulling through 2 mL of reaction mixture to fill the volume of the flow reactor and tubing. The power supply was set (500 mA, constant current) and attached to the ammonite flow reactor. The power supply was switched on and syringe pumping was initiated, and the outlet of the flow system was set to waste for the first 3 mL (representing 1 whole flow path volume, inclusive of tubing, connectors and reactor) of reaction mixture to allow the flow system to be filled. The outlet stream of the flow system was then collected for 102 mL (representing 5.1 mmol of material processed) at an operating potential of 4.4-4.6 V. Mesitylene (357 μL, 0.5 equiv.) was added to the product mixture, stirred for 5 min and then the ^{1}H NMR spectrum was recorded to give a crude reaction yield of 78%.

The reaction mixture was then concentrated *in vacuo*. The crude residue was purified by flash column chromatography (eluent = 10 to 20% EtOAc in hexanes, silica gel) to yield pure product (1.18 g, 74% yield).

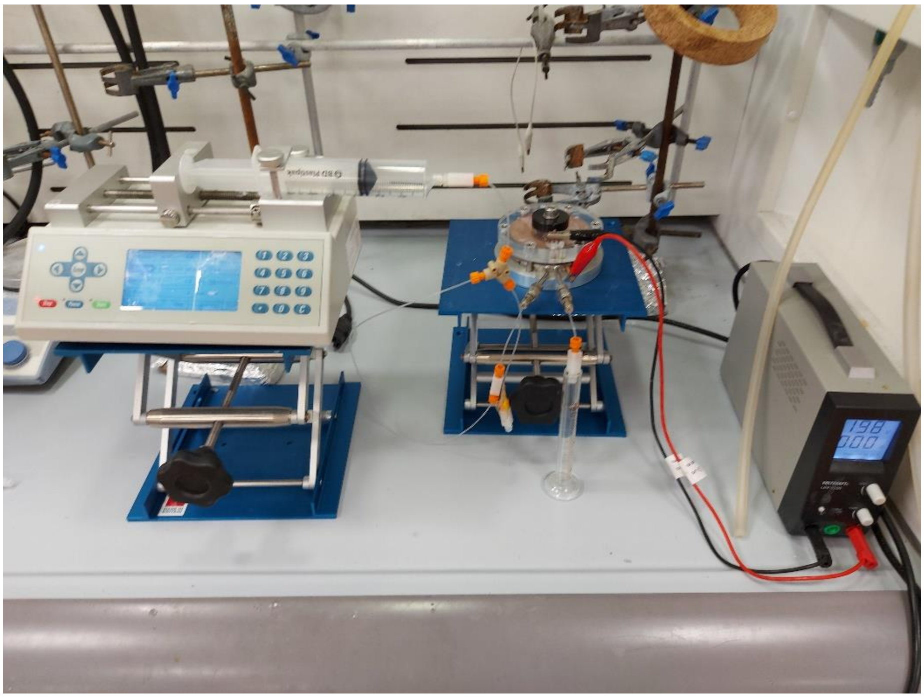

Electrochemical flow set up: Ammonite8 Reactor attached to a syringe pump and potentiostat

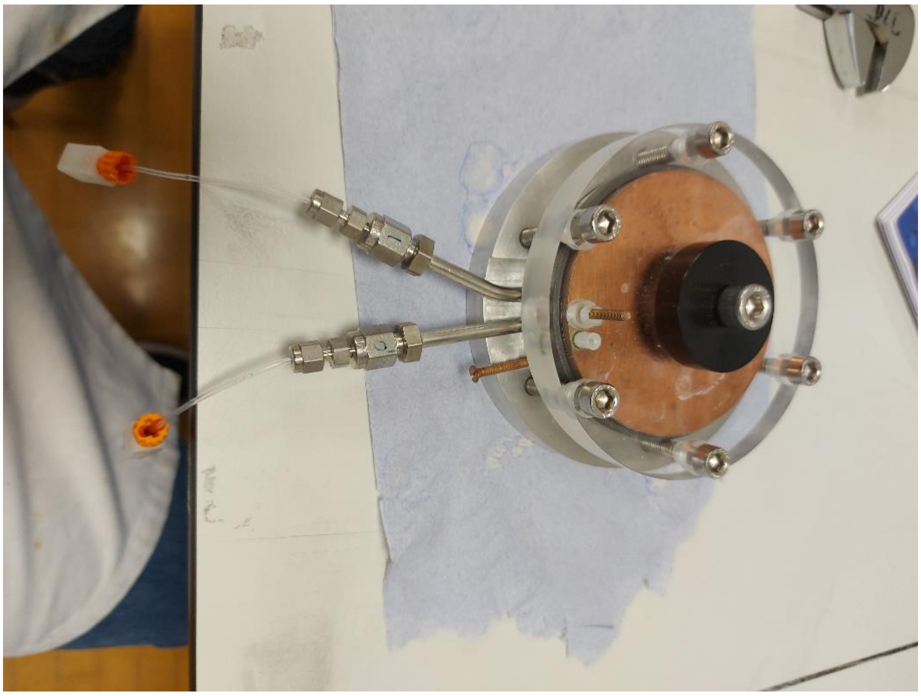

Constructed Ammonite8 Reactor

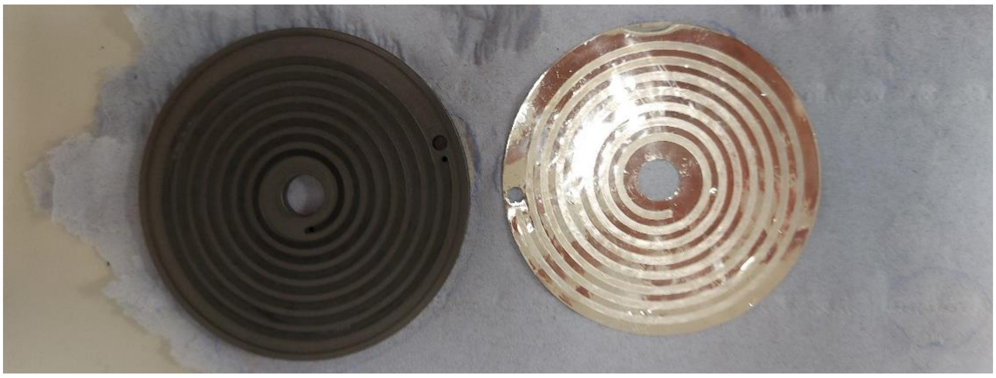

Prepared graphite (left) and platinum (right) electrodes

Proposed Mechanism through 1-aryl Ring Oxidation

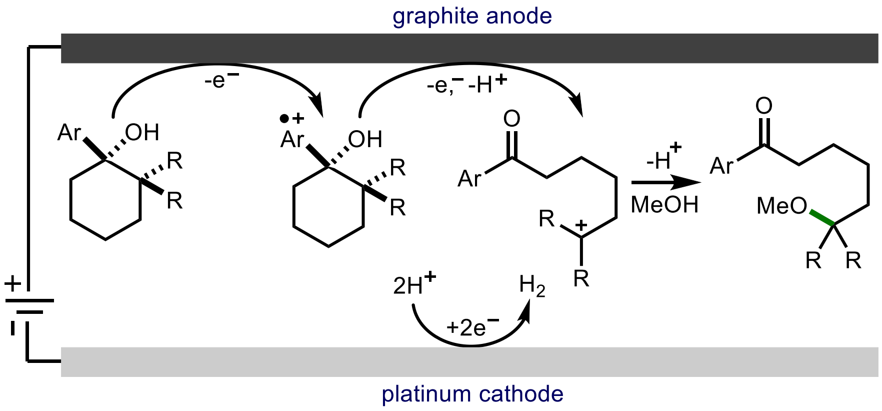

Cyclic Voltammetry Studies of Selected Substrates

Oxidative scans starting at 0.0 V.

(S1)

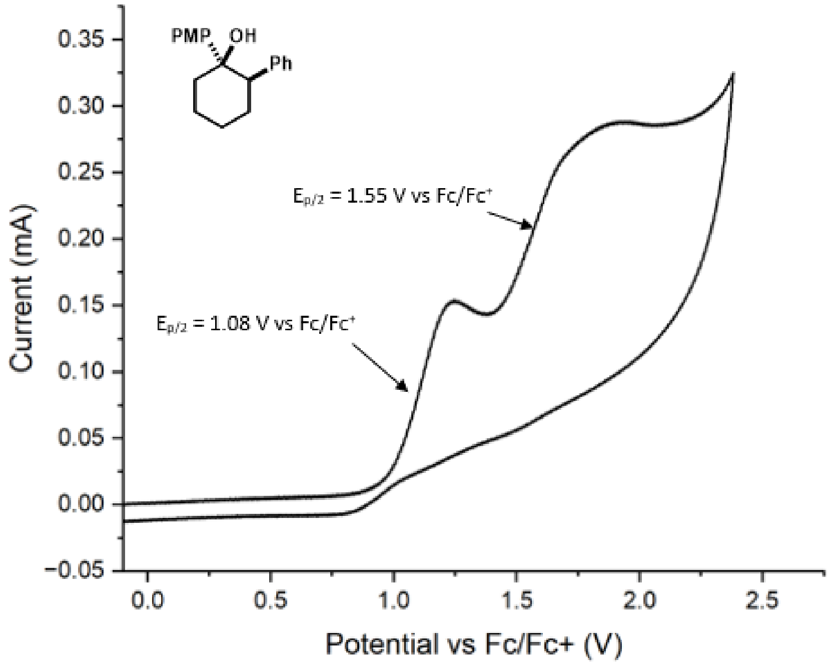

*CV (S1)-Cyclic Voltammogram of compound **S1** (5.0 mM in MeCN), NBu_4PF_6 (0.1 M), scan rate : 100 mV/s*

(S4)

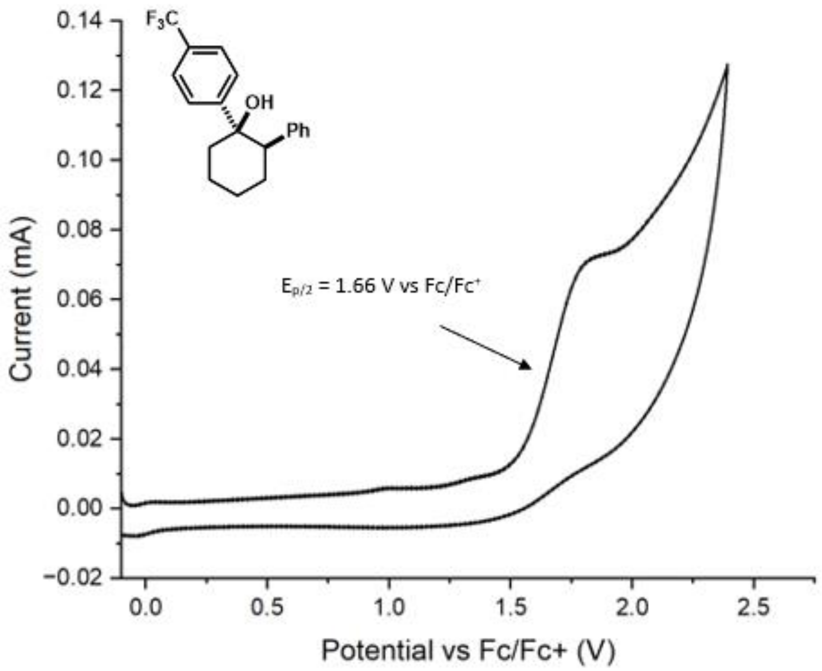

*CV (S4)-Cyclic Voltammogram of compound **S4** (5.0 mM in MeCN), NBu_4PF_6 (0.1 M), scan rate : 50 mV/s*

(S7)

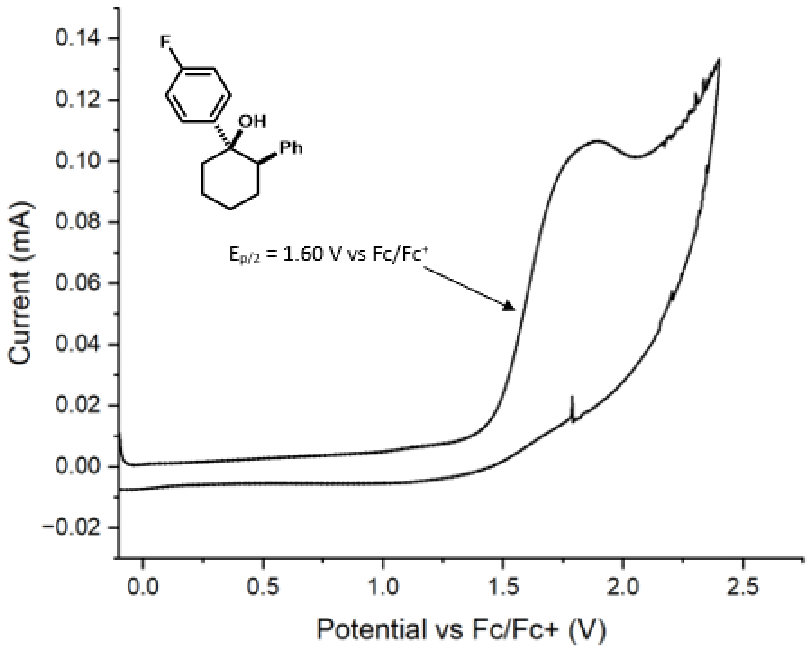

*CV (S7)-Cyclic Voltammogram of compound **S7** (5.0 mM in MeCN), NBu_4PF_6 (0.1 M), scan rate : 50 mV/s*

(S11)

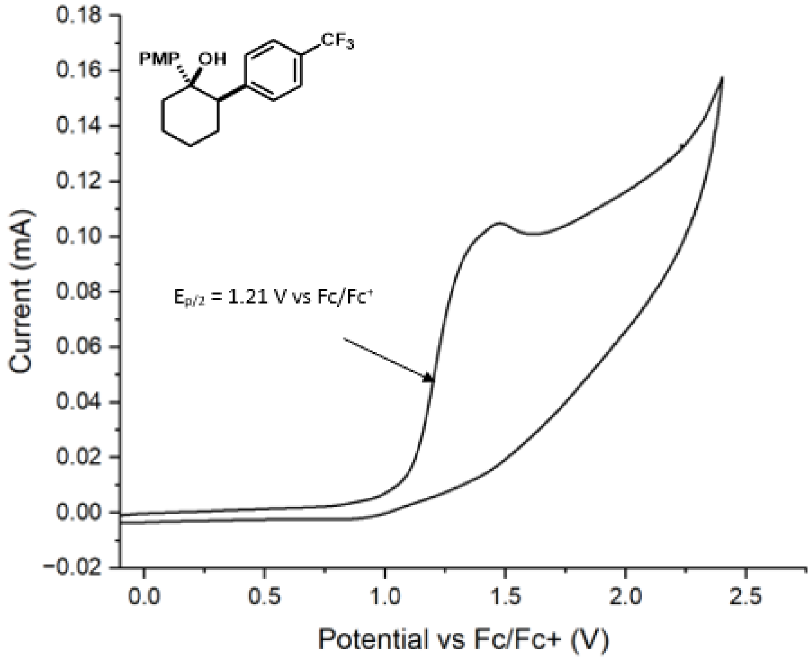

*CV (S11)-Cyclic Voltammogram of compound **S11** (5.0 mM in MeCN), NBu$_4$PF$_6$ (0.1 M), scan rate : 20 mV/s*

(S22)

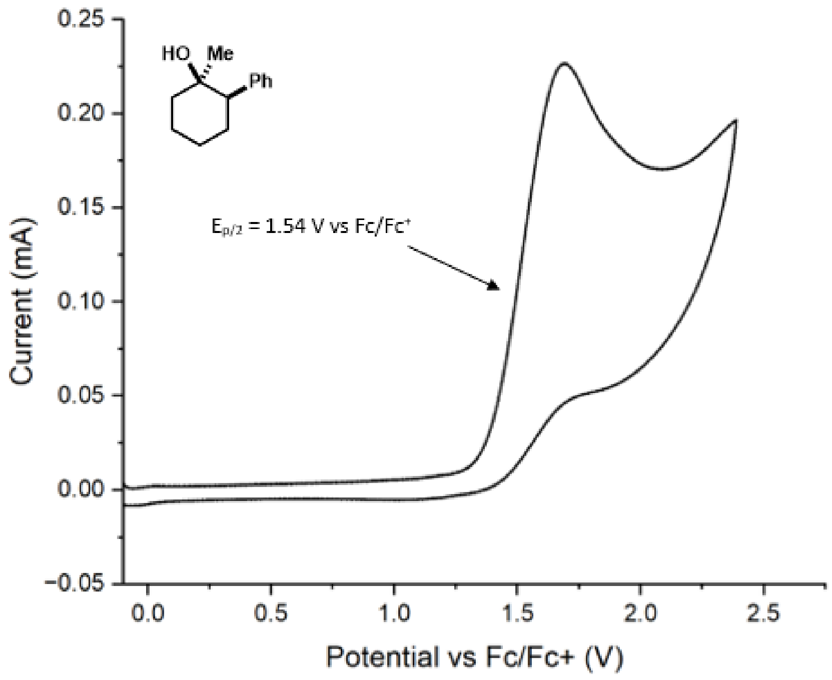

*CV (22)-Cyclic Voltammogram of compound **S22** (5.0 mM in MeCN), NBu$_4$PF$_6$ (0.1 M), scan rate : 100 mV/s*

(S27)

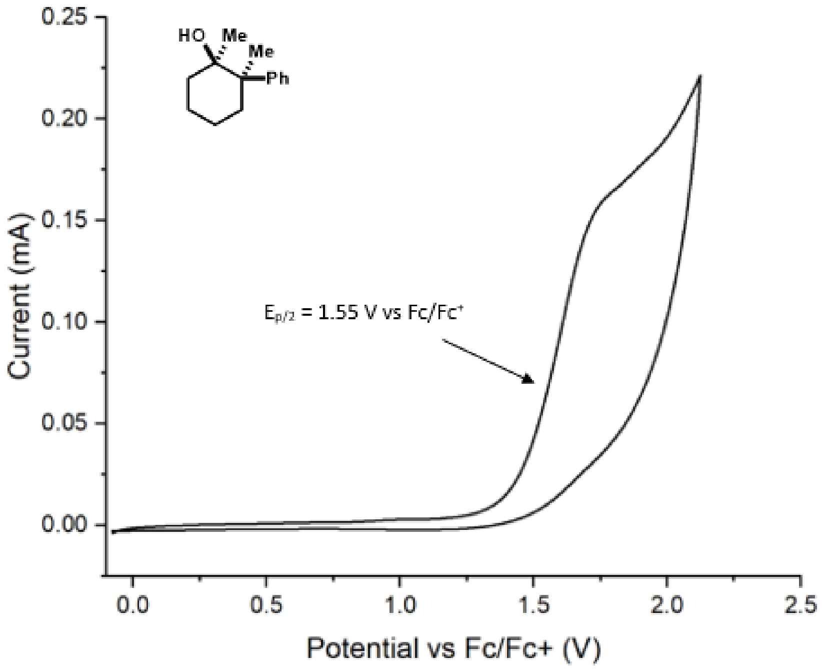

*CV (S27)-Cyclic Voltammogram of compound **S27** (10.0 mM in MeCN), NBu_4PF_6 (0.1 M), scan rate: 20 mV/s*

(S29)

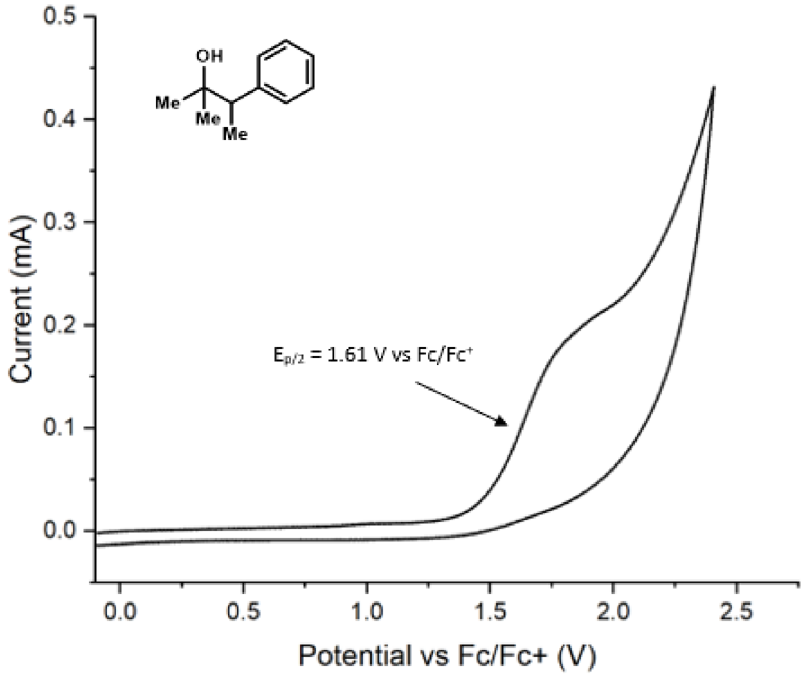

*CV (S29)-Cyclic Voltammogram of compound **S29** (10.0 mM in MeCN), NBu_4PF_6 (0.1 M), scan rate: 100 mV/s*

(S41)

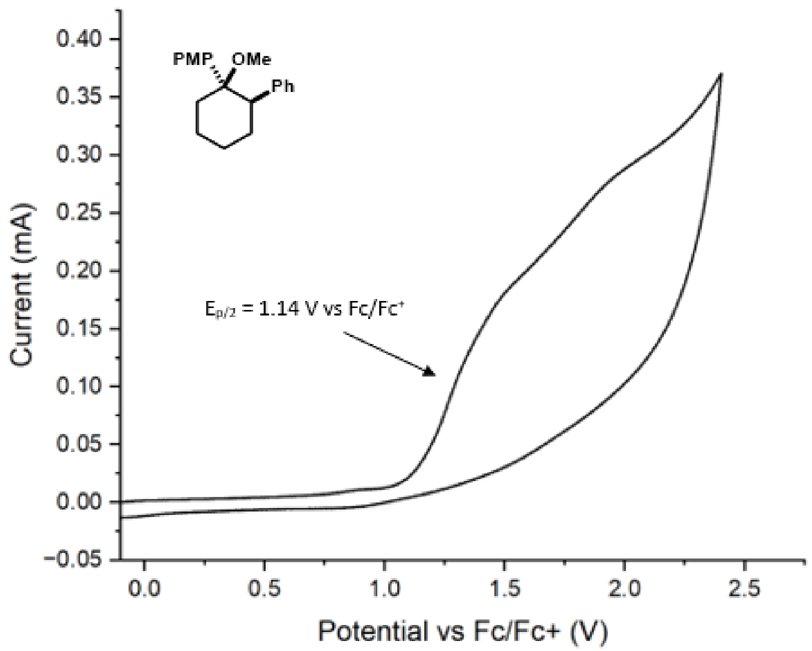

*CV (S41)-Cyclic Voltammogram of compound **S41** (5.0 mM in MeCN), NBu_4PF_6 (0.1 M), scan rate : 100 mV/s*

References

1) Hu, X.; Lichte, D.; Rodstein, I.; Weber, P.; Seitz, A.K.; Scherpf, T.; Gessner, V.H.; Gooßen, L.J. Ylide-Functionalized Phosphine (YPhos)–Palladium Catalysts: Selective Monoarylation of Alkyl Ketones with Aryl Chlorides. *Org. Lett.* **2019**, *21*, 7558–7562.
2) Allen, B. D. W.; Hareram, M. D.; Seastram, A. C.; McBride, T.; Wirth, T.; Browne, D. L.; Morrill, L. C. Manganese-Catalyzed Electrochemical Deconstructive Chlorination of Cycloalkanols via Alkoxy Radicals. *Org. Lett.* **2019**, *21*, 9241–9246.
3) Hirsch, J. A. Table of conformational energies. *Topics in Stereochemistry*. **1967**, *1*, 199-222
4) Berger, A. L.; Donabauer, K.; König, B. Photocatalytic carbanion generation from C–H bonds – reductant free Barbier/Grignard-type reactions. *Chem. Sci.* **2019**, *10*, 10991-10996.
5) Miller, J. L.; Zhou, L.; Liu, P.; Floreancig, P. E. Mechanism-Based Approach to Reagent Selection for Oxidative Carbon−Hydrogen Bond Cleavage Reactions. *Chem.Eur. J.* **2022**, *28*, e202103078.
6) Schneider, C.; Jackstell, R.; Maes, B. U. W.; Beller, M. Palladium-Catalyzed Alkoxycarbonylation of sec-Benzylic Ethers. *Eur. J. Org. Chem.* **2020**, *2020*, 932–936.
7) Hareram, M. D.; El Gehani, A. A. M. A.; Harnedy, J.; Seastram, A. C.; Jones, A. C.; Burns, M.; Wirth, T.; Browne, D. L.; Morrill, L. C. Electrochemical Deconstructive Functionalization of Cycloalkanols via Alkoxy Radicals Enabled by Proton -Coupled Electron Transfer. *Org. Lett.* **2022**, *24*, 3890 –3895.
